# Systemic downregulation of EV-associated MiRNAs following remote ischemic preconditioning

**DOI:** 10.1038/s41598-025-31356-9

**Published:** 2025-12-11

**Authors:** Marius Drysch, Alexander Fiedler, Sonja Verena Schmidt, Felix Reinkemeier, Flemming Puscz, Tabea Kurbacher, Ulrich H. Frey, Crista Ochsenfarth, Marcus Lehnhardt, Christoph Wallner, Alexander Sogorski

**Affiliations:** 1 Department of Plastic Surgery, BG University Hospital Bergmannsheil, Ruhr University Bochum, Bürkle-de-la-Camp Platz 1, 44789 Bochum, Germany; 2 Department of Gynecology and Obstetrics, St. Elisabeth-Hospital, Ruhr University Bochum, Bleichstraße 15, 44789 Bochum, Germany; 3 Department of Anesthesia, Intensive Care, Pain and Palliative Medicine, Ruhr-University Bochum, Marien Hospital Herne, 44625 Herne, Germany

**Keywords:** Extracellular vesicles, MiRNA, Ischemia-Reperfusion injury, Free flap surgery, Remote ischemic preconditioning, EV-miRNA profiling, GSEA, Biomarkers, Cardiology, Molecular biology

## Abstract

Remote ischemic preconditioning (RIPC) is a non-invasive strategy to protect tissues from ischemia-reperfusion injury (IRI), yet its systemic molecular mediators remain incompletely understood. Extracellular vesicles (EVs), particularly their microRNA (miRNA) cargo, have emerged as potential conveyors of RIPC-induced protection. In this translational study, we performed paired EV-miRNA profiling in five human subjects undergoing free flap surgery, with plasma collected before and 30 min after a standardized RIPC protocol. EVs were isolated, RNA was extracted and profiled using a 384-plex qPCR array. Differential expression was assessed using paired statistics and false discovery correction. Functional impact was inferred via validated target mapping, gene set enrichment analysis (GSEA), and miRNA-gene-pathway network modeling. RIPC induced a rapid, systemic shift in the circulating EV-miRNA profile. Principal component analysis revealed consistent directional changes post-RIPC. Four miRNAs, hsa-miR-505-3p (log2FC = -3.30, *p* = 0.025), hsa-miR-374a-5p (log2FC = -4.52, *p* = 0.028), hsa-miR-200a-3p (log2FC = -7.27, *p* = 0.044), and hsa-miR-181b-5p (log2FC = -3.94, *p* = 0.045), were significantly downregulated across the cohort. No miRNAs were consistently upregulated. GSEA of validated targets showed positive enrichment of stress-responsive and pro-survival pathways, including TNF-alpha signaling, TGF-beta, Notch, hypoxia, angiogenesis, and apoptosis. Network analysis identified miR-181b-5p and miR-374a-5p as central regulators affecting multiple interconnected pathways. These results suggest that RIPC elicits systemic protection not by introducing new molecular signals but by selectively removing suppressive EV-bound miRNAs. This may reflect a subtractive regulatory mechanism and enable rapid derepression of protective gene programs, offering a plausible explanation for the early benefits of RIPC. These findings should be validated in larger, multi-timepoint cohorts.

**Supplementary Information:**

The online version contains supplementary material available at 10.1038/s41598-025-31356-9.

## Introduction

Ischemia reperfusion injury (IRI) is a pathological process that drives tissue damage in acute conditions such as myocardial infarction, stroke, organ transplantation and free flap surgery, ultimately contributing to morbidity and mortality^1^. In these settings, timely reperfusion is essential to salvage ischemic tissue; however, paradoxically, the restoration of blood flow can itself exacerbate cell death and inflammation^2^. This phenomenon is called IRI. Despite advances in revascularization therapies, there are currently no broadly effective pharmacological interventions to prevent IRI, underscoring an urgent need for novel cytoprotective strategies^3^. One promising approach is remote ischemic preconditioning (RIPC), which involves applying brief, non-lethal cycles of ischemia and reperfusion to a limb to protect distant organs from subsequent IRI^4–6^. This simple, non-invasive procedure, often accomplished by inflating a blood-pressure cuff on the arm or leg for a few minutes at a time, utilizes an innate protective mechanism of the body. RIPC has demonstrated robust protective effects across a range of clinical settings, including reducing myocardial injury, preserving neurological function, and improving outcomes in major cardiovascular and surgical interventions^4,7–9^. However, the central mechanism by which RIPC confers systemic protection remains only partially understood and the biological pathways conveying the “preconditioning” signal from the limb to vital organs are not yet fully elucidated.

Current evidence suggests that the underlying mechanisms of RIPC involve a complex interplay of neural and humoral pathways^10–16^. While neural circuits, such as the vago-splenic axis, play a crucial role in signal transduction, the humoral pathway is characterized by the release of protective factors into the circulation. Among the leading candidates for these humoral mediators are extracellular vesicles (EVs) – nanoscale, membrane-bound vesicles released into the bloodstream^17,18^. Indeed, recent studies indicate that EVs released during RIPC are both necessary and sufficient for its remote benefits, as EVs isolated after a preconditioning stimulus have been shown to carry protective properties to distant cells^17,19^. EVs, which include exosomes (typically 30–150 nm in size) and slightly larger microvesicles, serve as intercellular messengers, carrying cargos of proteins, lipids, and nucleic acids between cells. Notably, they can circulate through the blood and deliver their cargo to specific target cells, thereby triggering signaling cascades in tissues far from their cell of origin. This has led to intense interest in EVs as the humoral factors of RIPC.

Among the diverse bioactive cargo carried by EVs, microRNAs (miRNAs) have emerged as particularly intriguing mediators of RIPC’s effects^18,20,21^. MiRNAs are small (~ 22 nucleotide) non-coding RNAs that regulate gene expression post-transcriptionally, typically by binding to target mRNAs and inhibiting their translation or stability^22^. These molecules have been identified in EVs and are remarkably stable in circulation, protected from enzymatic degradation within the vesicle lumen. It is well established that miRNAs can orchestrate cytoprotective or injury-response pathways in cells including pathways involved in apoptosis, inflammation, and metabolism^18,20,21,23^. RIPC has been hypothesized to provoke the release of specific EV-associated miRNAs into the bloodstream, which then home to vulnerable organs (e.g., heart or brain) and preemptively activate cytoprotective gene programs^19–21,24^. However, while a number of individual miRNAs have been postulated to play such roles, a comprehensive profile of the EV-bound miRNAs altered by RIPC is lacking. The present study addresses this gap by characterizing the circulating EV-miRNA signature before and after a standardized RIPC stimulus in humans. The aim of this study was to identify key miRNAs modulated by an RIPC stimulus by performing a direct, high-resolution comparative analysis of circulating EV-associated miRNA profiles immediately before vs. immediately after the RIPC intervention in human subjects undergoing free flap surgery. We hypothesized that RIPC would induce a rapid and significant alteration in circulating EV-miRNAs, resulting in the release of specific miRNAs that target pathways involved in cytoprotection.

## Materials and methods

### Study population and sample origin

The samples analyzed in this study were selected from the intervention (RIPC) arm of a larger clinical trial investigating the effects of RIPC on IRI in free flap surgery^25^. The present sub-study focuses on a representative cohort of five patients from this intervention group. Free flap reconstructions in this cohort were all performed because of a defect caused by cancer (e.g., breast reconstruction following mastectomy). All patients were cancer-free at the time of blood drawing and prior to their reconstructive surgery. RIPC was performed using a tourniquet applied to the patient’s arm, inflated to 250 mmHg for three cycles of ten-minute occlusion followed by ten-minute reperfusion. For these patients, plasma samples were collected at two time points: immediately before the RIPC procedure (pre-RIPC) and within 30 min post-intervention (post-RIPC). Blood was collected from the contralateral arm using a 14G peripheral intravenous access established without tourniquet application. Baseline blood sample collection, the RIPC maneuver, and the post-intervention blood sample collection were completed prior to the induction of anesthesia. Patient characteristics can be found in Table 1.

**Table 1:** Overview of patient Characteristics. Demographic information (pseudonym, age, gender, height, weight) and the underlying condition necessitating free flap reconstruction are presented for each of the five study patients.

Patient	Age	Gender	Height (cm)	Weight (kg)	Underlying condition
91	59	Male	176	86	Renal Cell Carcinoma
269	40	Female	167	78	Breast Cancer
277	44	Female	167	68	Breast Cancer
312	75	Female	155	90	Angiosarcoma
318	50	Female	168	73	Breast Cancer

### Ethics declarations

This study was conducted in accordance with the principles of the Declaration of Helsinki and was approved by the Institutional Ethics Committee of Ruhr University Bochum (Approval Number: 19–6752, 08 January 2020). All participants gave written informed consent prior to enrolment. In line with the General Data Protection Regulation (GDPR) and institutional policies, all samples were anonymized before analysis. The present sub-study analyzed only samples obtained from participants in the RIPC arms of the parent randomized trial.

### EV Isolation, MiRNA Extraction, and cDNA synthesis

Extracellular vesicles (EVs) were isolated from EDTA plasma using the ExoQuick (EQ) method as previously described^26^. Briefly, EVs were precipitated from 500 µl EDTA plasma using ExoQuick Exosome Precipitation (System Biosciences, Palo Alto, USA) as recommended by the manufacturer with minor modifications. After precipitation of the EVs from the plasma, we carried out a secondary purification step. Using PD Spin Traps G25 (Cytiva, Marlborough, USA) the EVs were filtered through sepharose to remove any remaining EQ from the extracted EVs. EV isolation efficiency and marker profile (e.g., Western Blot for Alix, CD63) using this specific ExoQuick protocol have been published by our group previously^26,27^. An aliquot of the isolated EVs was used for quantification and size determination via Nanoparticle Tracking Analysis (NTA) with a ZetaView^®^ (Particle Metrix, GmbH, Meerbusch, Germany) instrument as previously described. In short, the particle concentration was measured after concentration correction to 50–200 particles/position. We included five cycles and 11 positions. The “region of interest” (ROI) was set to 50–150 nm. NTA quantification data comparing pre- and post-RIPC samples is provided in Supple Figure S1 and Supplementary Table S1. For miRNA analysis, the remaining EV fraction was treated with RNase A (Thermo Fisher Scientific, Waltham, MA, USA) to remove miRNA or any other extra-vesicular RNA, ensuring that only intra-vesicular cargo was analyzed. Subsequent microRNA extraction was performed with the mirVANA™ miRNA Isolation Kit (Invitrogen, Thermo Fisher Scientific, Waltham, MA, USA) and the addition of a synthetic cel-mir-39-3p spike-in control (Invitrogen, Thermo Fisher Scientific, Waltham, MA, USA) to monitor extraction efficiency. The extracted miRNAs were stored at −80 °C until they were reverse-transcribed into cDNA using the TaqMan™ Advanced miRNA-cDNA Synthesis Kit according to the manufacturer’s instructions with a slight modification to the number of cycles in the mirAMP amplification step.

### MiRNA expression profiling and data processing

miRNA expression was profiled using quantitative real-time PCR and the Human Advanced miRNA Array Card A (Thermo Fisher Scientific, Waltham, MA, USA) according to the manufacturer’s instructions, without modification. Data acquisition was performed on a QuantStudio 7 Flex Real-Time PCR System. Raw amplification data (CT values) were processed using a custom Python pipeline. CT values reported as “undetermined” were conservatively imputed with a value of 40 to represent the limit of detection. A quality control filtering step was then applied: miRNAs were retained for analysis only if they were detected (CT < 40) in at least two of the five samples in either the pre-RIPC or post-RIPC group. This filtering removes uninformative assays while retaining miRNAs that may be specifically activated or repressed by the I/R event. For the retained miRNAs, CT values were normalized to the *cel-mir-39* spike-in control to compute ΔCT values (ΔCT = CTmiRNA​−CTspike − in​). The final log2-transformed relative expression values were calculated as − ΔCT and used for all downstream statistical analyses.

### Paired differential expression analysis

To identify miRNAs that were differentially expressed following RIPC, a paired analysis of the log2-transformed miRNA expression profiles was performed. For each miRNA, a paired, two-tailed Student’s t-test was used to compare the pre- and post-RIPC expression values within each of the five patients. The log2 fold change (log2FC) was calculated as the difference between the mean post-RIPC expression and the mean pre-RIPC expression. To account for multiple hypothesis testing, the resulting p-values were adjusted using the Benjamini-Hochberg method to generate a False Discovery Rate (FDR) q-value^28^. A miRNA was considered statistically significant if its FDR q-value was less than 0.2. To further increase confidence, “consistently regulated” miRNAs were identified as those that changed expression in the same direction (up or down) in at least three of the five patients.

### Bioinformatic pathway enrichment analysis

To infer the biological impact of the observed miRNA changes, we performed Gene Set Enrichment Analysis (GSEA). This was achieved by first translating the list of differentially expressed miRNAs into a ranked list of their validated gene targets. Raw miRNA identifiers were systematically cleaned and normalized to ensure compatibility with annotation databases. The normalized identifiers were mapped to their experimentally validated gene targets using the miRTarBase database, accessed via the gseapy (v1.1.0) Python library^29^. A ranked list of all unique target genes was generated. The ranking metric for each gene was set to the negative log2FC of its most strongly regulated targeting miRNA, reflecting the canonical inverse relationship between miRNA and target gene expression. Preranked GSEA was performed on this gene list using gseapy.prerank with 1,000 permutations. Enrichment was tested against the Human MSigDB Hallmark gene set^30,31^. A pathway was considered significantly enriched at an FDR q-value < 0.2. Results were visualized using dot plots and volcano plots.

### Network visualization

To synthesize these findings, a miRNA-gene-pathway interaction network was constructed. The network included miRNAs with a nominal p-value < 0.05, Hallmark pathways with an FDR q-value < 0.2, and the genes that were both validated targets of the included miRNAs and members of a leading-edge enrichment subset. The network was visualized using the networkx library with a Kamada-Kawai layout to illustrate the relationships between these components^32^.

## Results

### A distinct EV-miRNA signature is induced by remote ischemic preconditioning

**Fig. 1:**
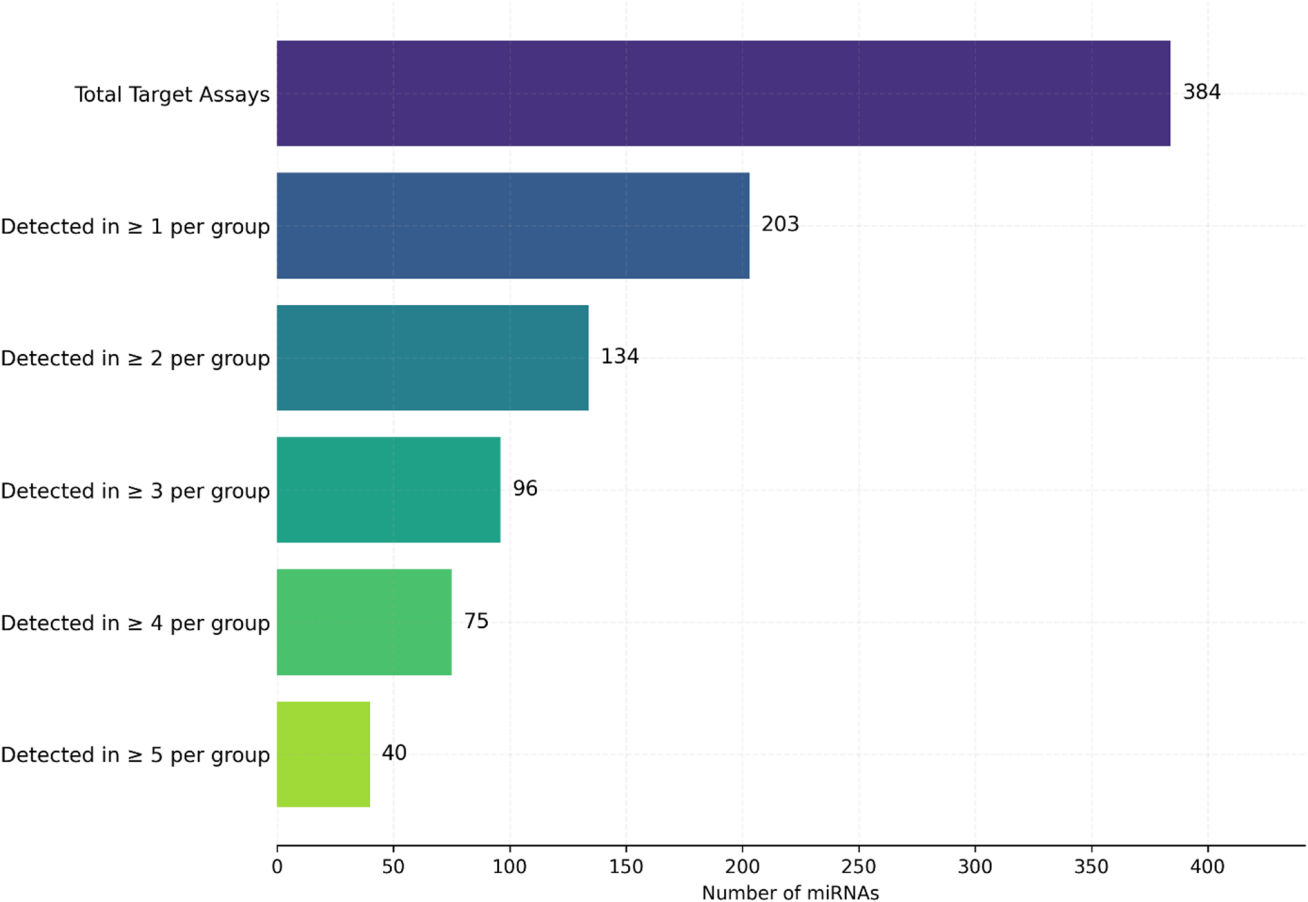
miRNA Data Filtering Cascade. The bar chart illustrates the number of miRNAs retained at each stage of the data filtering process. Starting from the 384 target miRNA assays on the array, 203 were detected in at least one sample in either the pre- or post-RIPC group. The final set of 134 miRNAs used for differential expression analysis was obtained by requiring detection in at least two samples in either group.

To characterize the circulating EV-miRNA profile in response to RIPC, we performed high-throughput qPCR analysis on plasma samples from five patients taken before and after the intervention. Our data processing and filtering cascade is summarized in Fig. 1. Of the 384 target miRNA assays on the array, 203 were robustly detected in at least one sample in either the pre- or post-RIPC group. Applying our pre-defined filtering criterion of retaining miRNAs detected in at least two samples per group resulted in a final set of 134 high-confidence miRNAs that were used for subsequent analyses.

### RIPC induces a systemic and asymmetric change in the Circulating EV-miRNome

**Fig. 2:**
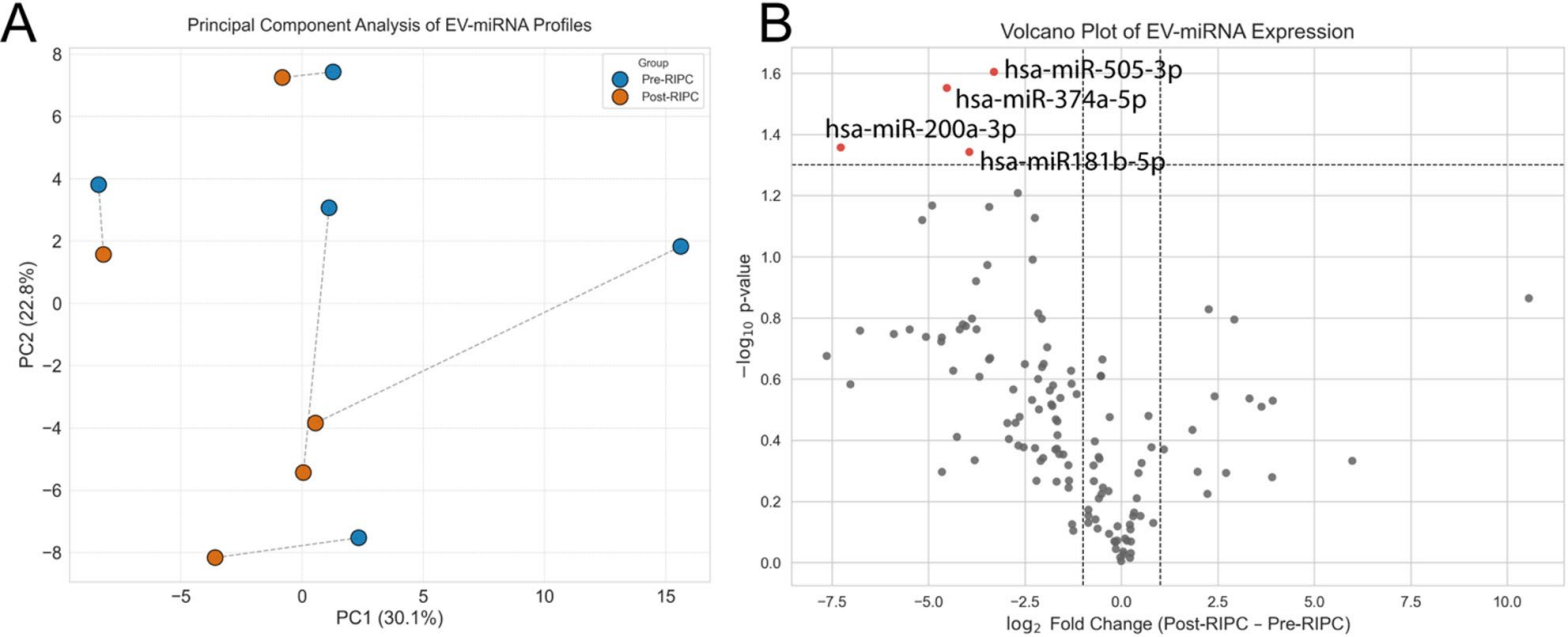
Global miRNA Profile Changes Following RIPC.** (A)** Principal Component Analysis of the 134 miRNA profiles. Each point represents a sample, with lines connecting the pre- and post-RIPC samples for each patient. The plot shows a consistent directional shift post-intervention. **(B)** Volcano plot illustrating the log2 fold change and statistical significance (-log10 p-value) for each miRNA. The four significantly downregulated miRNAs (*p* < 0.05) are highlighted in red.

To assess the global impact of the RIPC procedure on this miRNA profile, we first performed a Principal Component Analysis (PCA). The PCA plot revealed a clear and consistent trend, with post-RIPC samples shifting away from their pre-RIPC counterparts for each patient, indicating a distinct, systemic effect of the intervention on the circulating miRNome (Fig. 2A). To identify the specific miRNAs driving this change, we conducted a paired differential expression analysis. The volcano plot visualizes both the statistical significance and the magnitude of change for all 134 miRNAs (Fig. 2B). Several miRNAs clustered around a log2 fold change of zero, with no significant change in expression. However, a clear asymmetric response was observed: four miRNAs were significantly downregulated (*p* < 0.05), all with a strong magnitude of change (log2FC < −2.5), while no miRNAs were found to be significantly upregulated at this threshold. This suggests that the primary response to RIPC is a consistent downregulation of specific miRNAs.

### Top candidate MiRNAs exhibit a uniform downregulation pattern

**Fig. 3:**
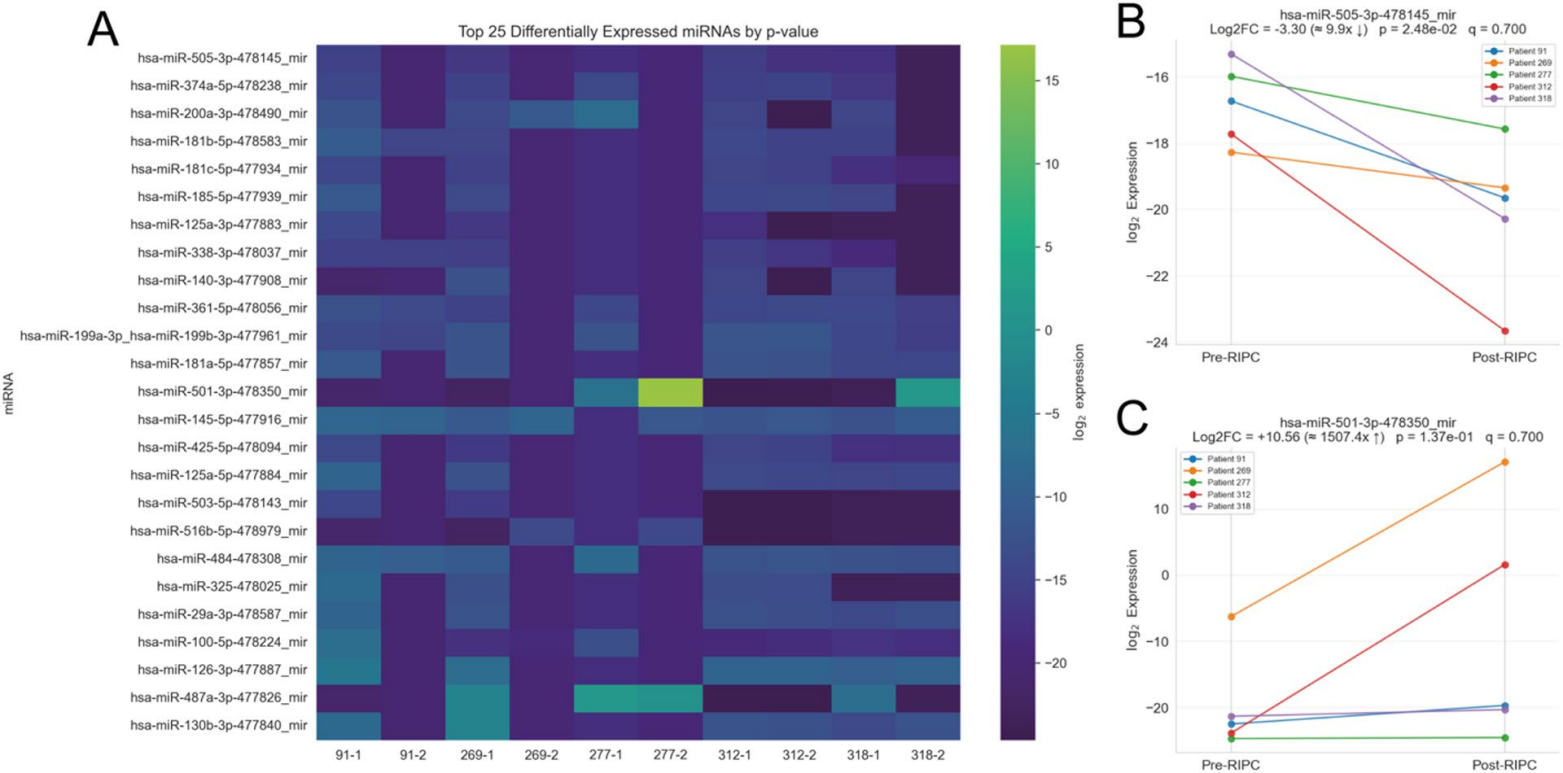
Characterization of Top Differentially Expressed miRNAs.** (A)** Heatmap of the top 25 miRNAs ranked by p-value. The columns represent individual samples, and rows represent miRNAs. The clear separation between pre- and post-RIPC samples is evident. **(B)** Variability plot for the top significant candidate, hsa-miR-505-3p, showing a consistent decrease in expression across all five patients. **(C)** Variability plot for hsa-miR-501-3p, illustrating an outlier upregulation pattern driven by a strong response in only two patients.

To further characterize the RIPC-induced EV-miRNA signature, we visualized the top 25 differentially expressed miRNAs (ranked by p-value) using a heatmap (Fig. 3A). This analysis revealed a clear separation between pre- and post-RIPC samples, primarily driven by a prominent cluster of downregulated miRNAs. Examination of individual miRNA changes across all five patients demonstrated a highly uniform downregulation pattern for the most significantly altered candidates. The top four (down-)regulated miRNAs were hsa-miR-505-3p (log2FC = −3.30, *p* = 0.025), hsa-miR-374a-5p (log2FC = −4.52, *p* = 0.028), hsa-miR-200a-3p (log2FC = −7.27, *p* = 0.044), and hsa-miR-181b-5p (log2FC = −3.94, *p* = 0.045), all of which decreased consistently in patients following RIPC (Fig. 3B; Supplementary Figure S2). In contrast, several miRNAs with a positive overall fold change, such as hsa-miR-501-3p (log2FC = 10.56, *p* = 0.137), exhibited heterogeneous expression profiles. These upregulation patterns were typically driven by one or two outlier patients, while the remaining individuals showed minimal change (Fig. 3C). This asymmetry was further supported by a consistency analysis across patients: miRNAs downregulated in ≥ 3 of 5 individuals (Supplementary Table S3) displayed substantially lower p-values compared to those in the upregulated group (Supplementary Table S4).

### Functional analysis predicts modulation of key protective and signaling pathways

**Fig. 4:**
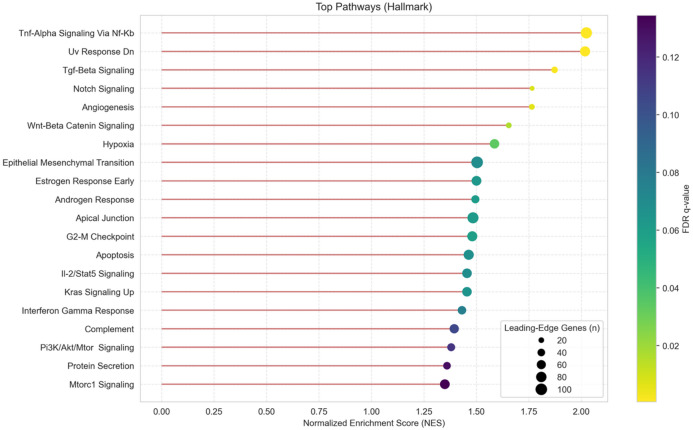
Functional Enrichment of the RIPC-Induced miRNA Signature. Dot plot of Gene Set Enrichment Analysis (GSEA) results against the Hallmark pathway database. The x-axis shows the Normalized Enrichment Score (NES). The size of each dot corresponds to the number of leading-edge genes in the pathway, and the color represents the FDR q-value. All significantly enriched pathways (FDR < 0.2) show a positive NES, indicating that their member genes are targets of the downregulated miRNAs.

To elucidate the potential biological function of the observed miRNA signature, we performed Gene Set Enrichment Analysis (GSEA). Given the canonical inverse relationship between miRNAs and their targets, a ranked list of validated target genes was generated based on the negative log2 fold change of their targeting miRNAs, using a comprehensive miRNA-target interaction map derived from the miRTarBase database (Supplementary Table S5). This analysis, focused on the MSigDB Hallmark collection, revealed a significant enrichment of several pathways critical to cellular signaling and stress responses (Fig. 4; Supplementary Figure S3).

Notably, the analysis showed a strong and consistent positive enrichment for pathways central to inflammation, cell fate, and tissue remodeling. The top five enriched pathways were TNF-alpha Signaling Via NF-kB (NES = 2.02, FDR q-val = 0.001), UV Response Down (NES = 2.02, FDR q-val = 0.0005), TGF-beta Signaling (NES = 1.87, FDR q-val = 0.002), Notch Signaling (NES = 1.77, FDR q-val = 0.008), and Angiogenesis (NES = 1.76, FDR q-val = 0.006). The uniformly positive Normalized Enrichment Scores (NES) indicate that the leading-edge genes of these pathways are concentrated among those whose targeting miRNAs were downregulated by RIPC. This suggests a functional derepression of these programs, consistent with a cellular “priming” mechanism. Furthermore, pathways directly relevant to ischemia-reperfusion injury, such as Hypoxia (NES = 1.69, FDR q-val = 0.003) and Apoptosis (NES = 1.52, FDR q-val = 0.005), were also significantly enriched. The coordinated regulation of these programs implies that the systemic EV-miRNA signal may prepare distant tissues for subsequent ischemic stress by modulating fundamental inflammatory, cell-survival and tissue-remodeling cascades.

### Network analysis reveals a coordinated regulatory hub

**Fig. 5:**
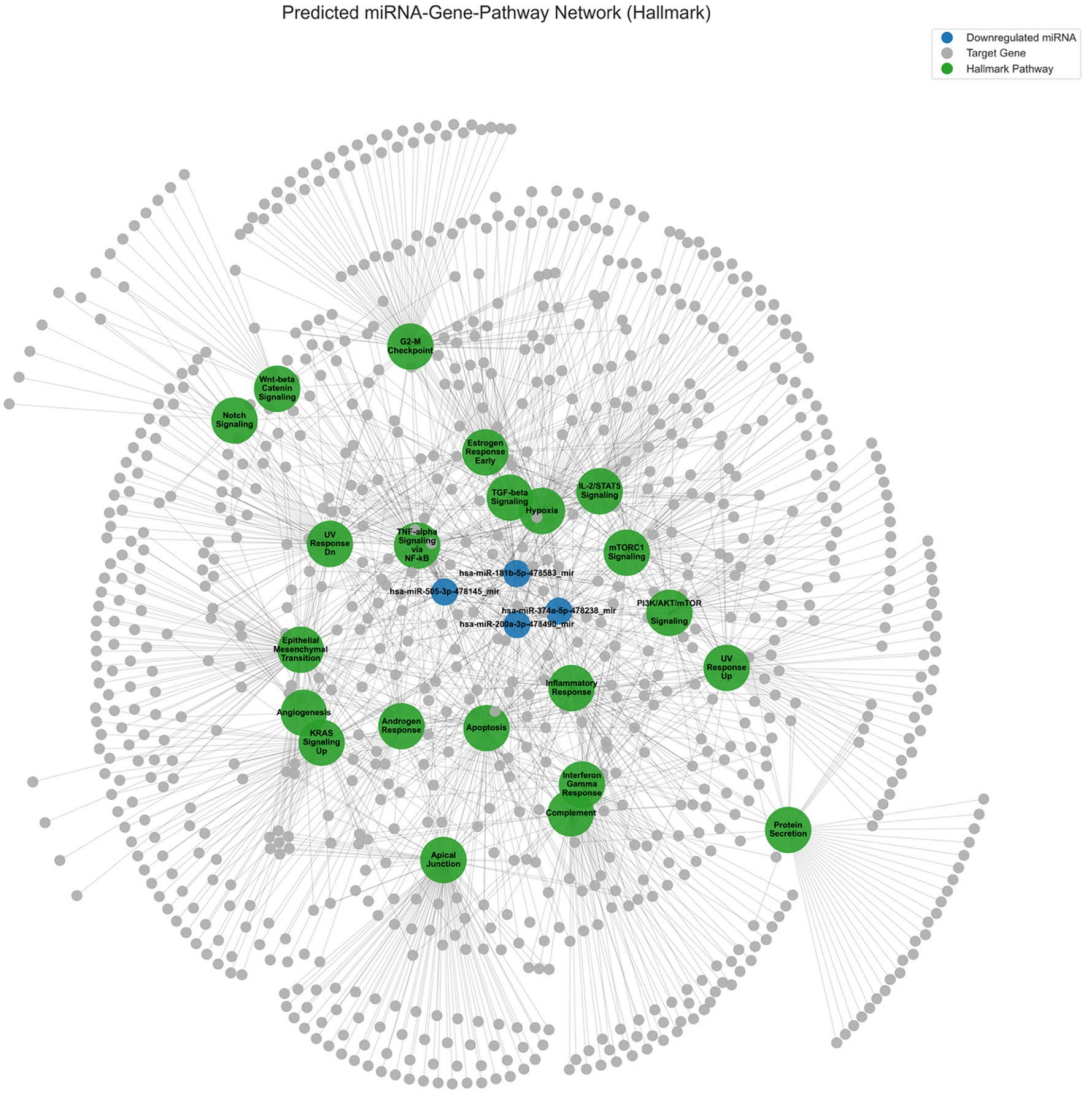
Predicted miRNA-Gene-Pathway Interaction Network. The network visualizes the connections between significantly downregulated miRNAs (*p* < 0.05, blue nodes), their validated gene targets that are part of a leading-edge subset (grey nodes), and the significantly enriched Hallmark pathways (FDR < 0.2, green nodes). The layout highlights the central role of the downregulated miRNAs in regulating a large number of genes across multiple protective pathways.

To synthesize our pathway enrichment and target prediction results into an integrative framework, we constructed a miRNA-gene-pathway interaction network using the Hallmark gene sets (Fig. 5). This network connects the four significantly downregulated EV-miRNAs (*p* < 0.05) to their predicted target genes within the leading-edge subsets of enriched pathways. The resulting topology reveals a highly coordinated regulatory hub in which a small set of miRNAs exerts broad influence across multiple interrelated stress-response pathways. Within this network, hsa-miR-181b-5p and hsa-miR-374a-5p emerge as central regulators, targeting 68 and 53 genes respectively, and dominating the interaction space compared to hsa-miR-200a-3p (35 genes) and hsa-miR-505-3p (25 genes). Notably, many of these target genes are not limited to a single function; they are shared components across several critical Hallmark pathways, including TNF-alpha/NF-κB signaling, hypoxia response, TGF-beta signaling, and apoptosis. This high degree of interconnectivity suggests a concerted, systems-level response, where RIPC-triggered EV-miRNA modulation reprograms stress-adaptive gene networks across tissues. Furthermore, the central positioning of these miRNAs suggests a model in which systemic EV-miRNA downregulation coordinates tissue-wide priming for ischemic tolerance. By targeting genes with such pleiotropic effects, the miRNA response to RIPC appears to induce a multi-layered protective phenotype that enhances resilience against subsequent IRI.

## Discussion

In this study, we demonstrate that a brief RIPC stimulus in humans provokes a rapid, systemic shift in the circulating EV-associated microRNA profile. Notably, this shift is defined by a coordinated downregulation of a small subset of miRNAs, with no miRNAs showing consistent or significant upregulation. All five patients exhibited a similar directional change post-RIPC, as evidenced by principal component analysis and paired comparisons. Four miRNAs (hsa-miR-181b-5p, hsa-miR-374a-5p, hsa-miR-200a-3p, and hsa-miR-505-3p) were significantly decreased in EVs after RIPC, with consistent trends across the cohort. In contrast, miRNAs with nominal positive fold-changes varied considerably between individuals and did not reach significance. This asymmetric pattern suggests that the primary early response to RIPC is not the addition of new protective signals, but the selective removal of key regulatory miRNAs from the EV compartment, potentially to relieve suppression of downstream stress-adaptive pathways.

These findings both complement and differ from earlier work on RIPC-induced molecular effects^18,20,33,34^. Several preclinical studies have described upregulation of specific cardioprotective miRNAs in EVs following RIPC, such as miR-24^18^, miR-126a-3p^35^, and miR-144^20^, which contribute to anti-apoptotic signaling or kinase activation in target tissues. In human cardiac surgery settings, RIPC has been associated with increased EV concentrations and altered EV miRNA content, including elevation of canonical cytoprotective miRNAs like miR-21^21^. However, our study did not identify consistent upregulation of any of these miRNAs in the acute phase following RIPC. Instead, the dominant response was a uniform reduction in a small set of EV-bound miRNAs. These differences likely reflect variations in timing and physiology. Our study captured the acute humoral response within 30 min of RIPC, whereas many prior findings pertain to later stages of conditioning or require subsequent ischemic insults to manifest fully^36,37^. It is therefore plausible that the early systemic response to RIPC in humans relies more on the withdrawal of inhibitory miRNAs than on the deployment of new effectors.

The striking downregulation of these miRNAs suggests that RIPC initiates a systemic release of constraints on multiple stress-responsive and pro-survival pathways. Gene set enrichment analysis revealed that the validated targets of these miRNAs cluster within Hallmark pathways involved in inflammation, cellular adaptation, and tissue remodeling, including TNFα and NF-κB signaling, TGF-β and Notch signaling, angiogenesis, hypoxia responses, and apoptosis regulation. These pathways exhibited positively skewed enrichment scores, indicating predicted upregulation of their member genes following miRNA downregulation. In functional terms, this suggests that RIPC removes miRNA-mediated repression from critical survival and repair pathways, transiently priming endothelial, immune, and parenchymal compartments for future ischemic stress.

The individual miRNAs reinforce this mechanistic interpretation: miR-181b-5p is a known repressor of NF-κB signaling through direct inhibition of importin-α3, a nuclear transport protein essential for NF-κB translocation^38,39^. Its reduction is expected to facilitate controlled NF-κB activation, a recognized early component of ischemic preconditioning. Similarly, miR-374a-5p is a known suppressor of chemokines such as MCP-1 and cytokines including IL-6 and TNF-α^40,41^. Its downregulation may allow a transient rise in inflammatory signaling that contributes to immune system priming. MiR-200a-3p targets components of the TGF-β/Smad and Notch pathways; its reduction is predicted to enhance anti-apoptotic and regenerative signaling^42–44^. Finally, miR-505-3p acts as a negative regulator of inflammation through pathways involving HMGB1 and Uromodulin^45–47^. Lowering miR-505-3p levels may permit a controlled increase in pro-inflammatory signaling that initiates protective cascades, such as M2 macrophage recruitment and ischemic tolerance.

Our findings support a framework in which RIPC induces a rapid, coherent reprogramming of the circulating EV miRNA landscape. Rather than broadcasting a single protective signal, RIPC modifies the composition of circulating EVs in a targeted manner, removing specific regulatory miRNAs that collectively govern survival, hypoxia, and immune-related gene networks. This mechanism may complement other forms of RIPC signal transmission, including neural and cytokine-mediated pathways, but is uniquely well-suited for targeted intercellular communication. EVs can home to specific organs and deliver cargo directly into recipient cells, and the absence of particular miRNAs from these vesicles may act as a functional cue^48^. Our network analysis further highlights miR-181b-5p and miR-374a-5p as central regulatory nodes with broad influence over multiple interconnected pathways. These miRNA changes likely contribute to the systems-level shift that prepares tissues for future ischemic insult. That such changes are detectable within the short time frame investigated in this study underscores the speed and coordination of this protective signaling mechanism.

This study has several limitations. First, the small sample size (*n* = 5) and the specific demographic distribution (predominance of female patients with prior breast cancer) limits generalizability, though the paired design provides internal consistency. These factors caution against extrapolating the findings to other populations without further validation. Second, our analysis was limited to a single early time point; dynamic changes occurring before or after 30 min may have been missed. Future studies should incorporate detailed time-course analyses to capture transient or delayed miRNA responses. Third, we relied on a predefined panel of 384 miRNAs using qPCR-based profiling. Unbiased small RNA sequencing may identify additional RIPC-responsive species, including rare or unannotated miRNAs. Fourth, we could not determine the cellular origin of the EV-bound miRNAs. RIPC likely affects multiple donor cell types, including endothelium, circulating immune cells, and limb muscle, and further studies using EV surface markers or tissue-specific profiling are warranted. Lastly, our study was observational in nature. Although bioinformatic analysis predicts derepression of protective pathways, these in silico predictions are hypothetical. Functional experiments are needed to establish causality and confirm that removal of these miRNAs contributes directly to ischemic protection.

Future research should aim to validate these findings in larger, independent cohorts with appropriate control groups. Time-course profiling of EV-miRNA content will be essential to map the kinetics of the response and identify optimal therapeutic windows. Functional studies using gain- and loss-of-function approaches are required to determine whether specific miRNAs mediate RIPC-induced protection. Investigating the upstream cues that lead to miRNA downregulation in EVs such as shear stress, hypoxia, or adrenergic signaling may provide further insight into how RIPC modifies vesicle content. From a translational perspective, targeted inhibition of the downregulated miRNAs using antagomirs could potentially mimic the effects of RIPC without requiring ischemic stimuli. Such a strategy would need to be carefully evaluated for safety and specificity but may open new options for prophylactic organ protection in high-risk surgical or ischemic settings.

Taken together, this study provides novel evidence that RIPC in humans rapidly alters the circulating EV miRNA landscape through the selective downregulation of key regulatory miRNAs. These changes appear to relieve repression on stress-responsive pathways, enabling broad activation of pro-survival, inflammatory, angiogenic, and anti-apoptotic gene networks. Rather than introducing new signals, RIPC may act primarily by withdrawing suppressive ones. This subtractive molecular editing, detectable within minutes of the stimulus, offers a compelling mechanistic explanation for the systemic benefits of RIPC. We emphasize, however, that given the sample size and statistical design, these results are exploratory in nature and should be viewed as hypothesis-generating. Nevertheless, our findings suggest that EV-associated miRNAs are not only markers of conditioning but may also serve as functional mediators, with potential implications for biomarker development and therapeutic innovation in IRI.

## Supplementary Information

Below is the link to the electronic supplementary material.

Supplementary Material 1

Supplementary Material 2

## Data Availability

All processed data supporting the findings of this study are included in this published article and its supplementary files. The raw qPCR data and code used for analysis are available from the corresponding author upon reasonable request.
