# Supplementary material for: Systemic downregulation of EV-associated MiRNAs following remote ischemic preconditioning
Source: Sci Rep. 2025 Dec 11;15:43657. doi: 10.1038/s41598-025-31356-9 (PMC12701078; doi:10.1038/s41598-025-31356-9)
Supplement: Supplementary file 1 — Supplementary Material 1 [file 41598_2025_31356_MOESM1_ESM.pdf]

Supplementary Tables for *Systemic Downregulation of EV-Associated miRNAs Following Remote Ischemic Preconditioning*

Marius Drysch,[1] Alexander Fiedler[1], Sonja Verena Schmidt,[1] Felix Reinkemeier,[1] Flemming Puscz[1], Tabea Kurbacher[2], Ulrich Frey[3], Crista Ochsenfarth[3], Marcus Lehnhardt,[1] Christoph Wallner,[1#] Alexander Sogorski[1#]

[1] Department of Plastic Surgery, BG University Hospital Bergmannsheil, Ruhr University Bochum, Bürkle-de-la-Camp Platz 1, 44789 Bochum, Germany

[2] Department of Gynecology and Obstetrics, St. Elisabeth-Hospital, Ruhr University Bochum, Bleichstraße 15, 44789 Bochum, Germany

[3] Department of Anesthesia, Intensive Care, Pain and Palliative Medicine, Ruhr-University Bochum, Marien Hospital Herne, 44625, Herne, Germany

# These authors contributed equally

Corresponding author:
Marius Drysch, MD, MHBA
Bürkle-de-la-Camp-Platz 1
44789 Bochum, Germany
+49 234 302 0
Email: marius.drysch@rub.de

Keywords: Extracellular vesicles, miRNA, Ischemia-Reperfusion Injury, Free Flap surgery, Remote Ischemic Preconditioning, EV-miRNA profiling, GSEA

Sample Name	Conc (p./mL)	Part in ROI 50-150nm	Dilution	Conc (p./mL)	To	Peak ø (nm)
91.1	3,20E+07	2,80E+07	20000	6,40E+11	5,60E+11	100,2
91.2	4,60E+07	3,80E+07	20000	9,20E+11	7,60E+11	107,4
269.1	6,50E+07	5,50E+07	5000	3,25E+11	2,75E+11	102,6
269.2	3,80E+07	3,10E+07	10000	3,80E+11	3,10E+11	89,2
277.1	4,20E+07	3,30E+07	20000	8,40E+11	6,60E+11	115,6
277.2	3,40E+07	2,20E+07	40000	1,36E+12	8,80E+11	122,9
312.1	5,20E+07	3,80E+07	2500	1,30E+11	9,50E+10	110,8
312.2	6,20E+07	4,20E+07	2500	1,55E+11	1,05E+11	115,3
318.1	7,60E+07	5,70E+07	2500	1,90E+11	1,43E+11	110,6
318.2	5,70E+07	4,70E+07	5000	2,85E+11	2,35E+11	104,8

Supplementary Table S1. Nanoparticle Tracking Analysis (NTA) Quantification Data. Raw NTA data for EVs isolated from the plasma of five patients. Samples were collected immediately before (indicated by sample suffix ".1") and 30 minutes after (indicated by sample suffix ".2") RIPC. The table displays the raw particle concentration (Conc) and the number of particles detected within the 50–150 nm region of interest (Part in ROI), alongside the dilution factor applied. The final columns report the total dilution-corrected particle concentration and the concentration of particles specifically within the 50–150 nm size range (To), followed by the peak particle diameter (Peak ø) in nanometers.

miRNA	p_value	log2FC	FC_linear	q_value_FDR
hsa-miR-505-3p-478145_mir	0.025	-3.302	-9.861	0.700
hsa-miR-374a-5p-478238_mir	0.028	-4.523	-22.995	0.700
hsa-miR-200a-3p-478490_mir	0.044	-7.272	-154.604	0.700
hsa-miR-181b-5p-478583_mir	0.045	-3.940	-15.350	0.700
hsa-miR-181c-5p-477934_mir	0.062	-2.684	-6.424	0.700
hsa-miR-185-5p-477939_mir	0.068	-4.904	-29.941	0.700
hsa-miR-125a-3p-477883_mir	0.069	-3.425	-10.744	0.700
hsa-miR-338-3p-478037_mir	0.075	-2.242	-4.731	0.700
hsa-miR-140-3p-477908_mir	0.076	-5.160	-35.763	0.700
hsa-miR-361-5p-478056_mir	0.102	-2.299	-4.921	0.700
hsa-miR-199a-3p_hsa-miR-199b-3p-477961_mir	0.106	-3.473	-11.101	0.700
hsa-miR-181a-5p-477857_mir	0.120	-3.766	-13.607	0.700
hsa-miR-501-3p-478350_mir	0.137	10.558	1507.435	0.700
hsa-miR-145-5p-477916_mir	0.149	2.263	4.798	0.700
hsa-miR-425-5p-478094_mir	0.153	-2.155	-4.453	0.700
hsa-miR-125a-5p-477884_mir	0.159	-3.872	-14.643	0.700
hsa-miR-503-5p-478143_mir	0.159	-2.065	-4.185	0.700
hsa-miR-516b-5p-478979_mir	0.160	2.925	7.595	0.700
hsa-miR-484-478308_mir	0.166	-4.108	-17.248	0.700
hsa-miR-325-478025_mir	0.169	-4.030	-16.337	0.700
hsa-miR-29a-3p-478587_mir	0.173	-3.754	-13.495	0.700
hsa-miR-100-5p-478224_mir	0.173	-4.189	-18.239	0.700
hsa-miR-126-3p-477887_mir	0.173	-5.487	-44.842	0.700
hsa-miR-487a-3p-477826_mir	0.174	-6.772	-109.325	0.700
hsa-miR-130b-3p-477840_mir	0.179	-5.897	-59.585	0.700
hsa-miR-30b-5p-478007_mir	0.183	-5.063	-33.425	0.700
hsa-miR-146a-5p-478399_mir	0.184	-4.654	-25.179	0.700
hsa-miR-142-3p-477910_mir	0.189	-4.668	-25.418	0.700
hsa-miR-155-5p-477927_mir	0.198	-1.922	-3.789	0.700
hsa-miR-190a-5p-478358_mir	0.211	-7.636	-198.943	0.700
hsa-let-7a-5p-478575_mir	0.214	-3.399	-10.546	0.700
hsa-miR-27a-3p-478384_mir	0.217	-3.430	-10.775	0.700
hsa-miR-23a-3p-478532_mir	0.217	-0.493	-1.407	0.700
hsa-let-7d-5p-478439_mir	0.224	-2.014	-4.038	0.700
hsa-miR-27b-3p-478270_mir	0.224	-2.501	-5.662	0.700
hsa-miR-652-3p-478189_mir	0.229	-2.058	-4.165	0.700
hsa-miR-10a-5p-479241_mir	0.236	-4.357	-20.499	0.700
hsa-miR-224-5p-477986_mir	0.236	-1.303	-2.468	0.700

hsa-miR-16-5p-477860_mir	0.245	-0.531	-1.445	0.700
hsa-miR-16-5p-477860_mir	0.245	-0.531	-1.445	0.700
hsa-miR-16-5p-477860_mir	0.245	-0.531	-1.445	0.700
hsa-miR-16-5p-477860_mir	0.245	-0.531	-1.445	0.700
hsa-miR-16-5p-477860_mir	0.245	-0.531	-1.445	0.700
hsa-miR-139-5p-478312_mir	0.247	-3.678	-12.802	0.700
hsa-miR-101-3p-477863_mir	0.251	-2.160	-4.468	0.700
hsa-miR-153-3p-477922_mir	0.260	-1.292	-2.449	0.700
hsa-miR-135a-5p-478581_mir	0.261	-7.021	-129.889	0.700
hsa-miR-148a-3p-477814_mir	0.264	-1.771	-3.412	0.700
hsa-miR-494-3p-478135_mir	0.272	-2.801	-6.970	0.700
hsa-miR-107-478254_mir	0.274	-1.855	-3.618	0.700
hsa-miR-17-5p-478447_mir	0.281	-1.159	-2.233	0.700
hsa-miR-376c-3p-478459_mir	0.286	2.416	5.335	0.700
hsa-miR-24-3p-477992_mir	0.290	-1.580	-2.990	0.700
hsa-let-7f-5p-478578_mir	0.291	3.319	9.977	0.700
hsa-miR-134-5p-477901_mir	0.294	-2.314	-4.974	0.700
hsa-miR-363-3p-478060_mir	0.296	3.921	15.149	0.700
hsa-miR-148b-3p-477824_mir	0.304	-1.815	-3.519	0.700
hsa-miR-222-3p-477982_mir	0.308	-1.787	-3.451	0.700
hsa-miR-490-3p-478131_mir	0.309	3.630	12.376	0.700
hsa-miR-193a-5p-477954_mir	0.316	-2.137	-4.398	0.700
hsa-miR-335-5p-478324_mir	0.331	0.702	1.626	0.700
hsa-miR-19b-3p-478264_mir	0.334	-2.638	-6.226	0.700
hsa-miR-28-5p-478000_mir	0.335	-0.303	-1.234	0.700
hsa-miR-10b-5p-478494_mir	0.340	-1.698	-3.245	0.700
hsa-miR-500a-5p-478309_mir	0.345	-1.660	-3.161	0.700
hsa-miR-150-5p-477918_mir	0.349	-2.744	-6.698	0.700
hsa-miR-380-3p-477854_mir	0.350	-2.955	-7.754	0.700
hsa-miR-885-5p-478207_mir	0.368	1.841	3.583	0.717
hsa-miR-103a-3p-478253_mir	0.383	-1.657	-3.154	0.717
hsa-miR-133a-3p-478511_mir	0.388	-4.262	-19.185	0.717
hsa-miR-1-3p-477820_mir	0.394	-2.915	-7.543	0.717
hsa-miR-92b-3p-477823_mir	0.402	-0.689	-1.612	0.717
hsa-miR-21-5p-477975_mir	0.414	-2.669	-6.358	0.717
hsa-miR-130a-3p-477851_mir	0.420	0.779	1.716	0.717
hsa-miR-26b-5p-478418_mir	0.420	-2.534	-5.794	0.717
hsa-let-7e-5p-478579_mir	0.422	-2.238	-4.718	0.717
hsa-miR-19a-3p-479228_mir	0.424	-1.672	-3.187	0.717
hsa-miR-25-3p-477994_mir	0.427	1.101	2.145	0.717

hsa-miR-125b-5p-477885_mir	0.427	-1.709	-3.270	0.717
hsa-let-7i-5p-478375_mir	0.442	-1.611	-3.054	0.717
hsa-let-7g-5p-478580_mir	0.443	-1.506	-2.841	0.717
hsa-miR-92a-3p-477827_mir	0.452	-0.589	-1.505	0.717
hsa-miR-18a-5p-478551_mir	0.455	-2.029	-4.081	0.717
hsa-miR-376a-3p-478240_mir	0.458	-0.569	-1.484	0.717
hsa-miR-326-478027_mir	0.463	-3.802	-13.950	0.717
hsa-miR-433-3p-478102_mir	0.465	5.982	63.199	0.717
hsa-miR-342-3p-478043_mir	0.465	-2.093	-4.265	0.717
hsa-miR-32-5p-478026_mir	0.473	0.523	1.437	0.717
hsa-miR-214-3p-477974_mir	0.480	-1.374	-2.593	0.717
hsa-miR-499a-5p-478139_mir	0.481	-0.721	-1.649	0.717
hsa-miR-29b-3p-478369_mir	0.505	1.975	3.930	0.726
hsa-miR-199a-5p-478231_mir	0.505	-4.650	-25.112	0.726
hsa-miR-502-3p-478348_mir	0.509	2.712	6.554	0.726
hsa-miR-518e-3p-479408_mir	0.510	0.443	1.359	0.726
hsa-miR-744-5p-478200_mir	0.526	3.905	14.985	0.736
hsa-miR-339-5p-478040_mir	0.540	-1.358	-2.564	0.736
hsa-miR-15b-5p-478313_mir	0.540	-2.200	-4.594	0.736
hsa-miR-127-3p-477889_mir	0.542	-0.714	-1.640	0.736
hsa-miR-423-3p-478327_mir	0.544	-1.679	-3.202	0.736
hsa-miR-320a-478594_mir	0.569	-0.479	-1.394	0.755
hsa-miR-194-5p-477956_mir	0.569	-1.369	-2.582	0.755
hsa-miR-451a-478107_mir	0.584	-0.333	-1.260	0.767
hsa-miR-31-5p-478015_mir	0.596	2.230	4.690	0.770
hsa-miR-374b-5p-478389_mir	0.597	-0.518	-1.432	0.770
hsa-miR-26a-5p-477995_mir	0.616	0.398	1.318	0.780
hsa-miR-653-5p-479134_mir	0.617	-0.585	-1.500	0.780
hsa-miR-93-5p-478210_mir	0.672	-0.855	-1.809	0.842
hsa-miR-186-5p-477940_mir	0.686	0.330	1.257	0.852
hsa-miR-99b-5p-478343_mir	0.702	-0.858	-1.813	0.852
hsa-miR-128-3p-477892_mir	0.705	0.494	1.408	0.852
hsa-miR-452-5p-478109_mir	0.706	0.305	1.235	0.852
hsa-miR-324-5p-478024_mir	0.723	-0.673	-1.594	0.865
hsa-miR-20a-5p-478586_mir	0.741	-0.858	-1.812	0.868
hsa-miR-660-5p-478192_mir	0.742	0.827	1.774	0.868
hsa-miR-424-5p-478092_mir	0.750	-1.278	-2.424	0.868
hsa-miR-448-478105_mir	0.752	0.220	1.165	0.868
hsa-miR-223-3p-477983_mir	0.761	-0.100	-1.072	0.872
hsa-let-7b-5p-478576_mir	0.775	-0.617	-1.534	0.877

hsa-miR-382-5p-478078_mir	0.779	0.235	1.177	0.877
hsa-miR-210-3p-477970_mir	0.787	-1.247	-2.373	0.879
hsa-miR-323a-3p-477853_mir	0.805	-0.320	-1.248	0.892
hsa-miR-486-5p-478128_mir	0.835	0.096	1.068	0.906
hsa-miR-15a-5p-477858_mir	0.847	-0.111	-1.080	0.906
hsa-miR-495-3p-478136_mir	0.849	0.146	1.107	0.906
hsa-miR-485-3p-478125_mir	0.852	-0.182	-1.134	0.906
hsa-miR-429-477849_mir	0.854	0.247	1.187	0.906
hsa-miR-576-3p-478164_mir	0.859	-0.152	-1.111	0.906
hsa-miR-2110-477971_mir	0.903	-0.142	-1.104	0.946
hsa-miR-423-5p-478090_mir	0.921	0.049	1.034	0.957
hsa-miR-629-5p-478183_mir	0.932	0.244	1.184	0.960
hsa-miR-191-5p-477952_mir	0.940	0.060	1.042	0.962
hsa-miR-221-3p-477981_mir	0.961	-0.029	-1.020	0.973
hsa-miR-483-5p-478432_mir	0.966	0.223	1.167	0.973
hsa-miR-22-3p-477985_mir	0.989	-0.007	-1.005	0.989

Supplementary Table S2: Differential expression results for all 134 relevant miRNAs. The table lists all miRNAs that passed the filtering criteria and were used in the differential expression analysis. Columns include miRNA identifier, nominal p-value, log2 fold change (log2FC), and the False Discovery Rate (FDR) adjusted q-value. The linear fold change (FC_linear) is reported to provide an intuitive measure of change magnitude: for upregulated miRNAs (positive log2FC), it represents the standard fold change (2^{log2FC}); for downregulated miRNAs (negative log2FC), it is represented as the negative reciprocal ($-2^{|log2FC|}$) to make the magnitude comparable to upregulation.

miRNA	p_value	log2FC	FC_linear	q_value_FDR
hsa-miR-374a-5p-478238_mir	0.028	-4.523	-22.995	0.700
hsa-miR-200a-3p-478490_mir	0.044	-7.272	-154.604	0.700
hsa-miR-181b-5p-478583_mir	0.045	-3.94	-15.35	0.700
hsa-miR-185-5p-477939_mir	0.068	-4.904	-29.941	0.700
hsa-miR-140-3p-477908_mir	0.076	-5.16	-35.763	0.700
hsa-miR-199a-3p_hsa-miR-199b-3p-477961_mir	0.106	-3.473	-11.101	0.700
hsa-miR-181a-5p-477857_mir	0.12	-3.766	-13.607	0.700
hsa-miR-125a-5p-477884_mir	0.159	-3.872	-14.643	0.700
hsa-miR-484-478308_mir	0.166	-4.108	-17.248	0.700
hsa-miR-325-478025_mir	0.169	-4.03	-16.337	0.700
hsa-miR-126-3p-477887_mir	0.173	-5.487	-44.842	0.700
hsa-miR-100-5p-478224_mir	0.173	-4.189	-18.239	0.700
hsa-miR-29a-3p-478587_mir	0.173	-3.754	-13.495	0.700
hsa-miR-487a-3p-477826_mir	0.174	-6.772	-109.325	0.700
hsa-miR-130b-3p-477840_mir	0.179	-5.897	-59.585	0.700
hsa-miR-30b-5p-478007_mir	0.183	-5.063	-33.425	0.700
hsa-miR-146a-5p-478399_mir	0.184	-4.654	-25.179	0.700
hsa-miR-142-3p-477910_mir	0.189	-4.668	-25.418	0.700
hsa-miR-190a-5p-478358_mir	0.211	-7.636	-198.943	0.700
hsa-miR-10a-5p-479241_mir	0.236	-4.357	-20.499	0.700
hsa-miR-139-5p-478312_mir	0.247	-3.678	-12.802	0.700
hsa-miR-135a-5p-478581_mir	0.261	-7.021	-129.889	0.700
hsa-miR-133a-3p-478511_mir	0.388	-4.262	-19.185	0.717
hsa-miR-326-478027_mir	0.463	-3.802	-13.95	0.717
hsa-miR-199a-5p-478231_mir	0.505	-4.65	-25.112	0.726

Supplementary Table S3: Top consistently downregulated miRNAs. The table lists the top 25 miRNAs that were consistently downregulated in at least 3 of the 5 patients, selected based on the magnitude of their log2 fold change. The list is presented sorted by ascending p-value to highlight the most statistically significant candidates within this group. Columns include miRNA identifier, nominal p-value, log2 fold change (log2FC), and the False Discovery Rate (FDR) adjusted q-value. The linear fold change (FC_linear) is reported to provide an intuitive measure of change magnitude: for upregulated miRNAs (positive log2FC), it represents the standard fold change (2^{log2FC}); for downregulated miRNAs (negative log2FC), it is represented as the negative reciprocal ($-2^{|\text{log2FC}|}$) to make the magnitude comparable to upregulation.

miRNA	p_value	log2FC	FC_linear	q_value_FDR
hsa-miR-501-3p-478350_mir	0.137	10.558	1507.435	0.700
hsa-miR-145-5p-477916_mir	0.149	2.263	4.798	0.700
hsa-miR-516b-5p-478979_mir	0.160	2.925	7.595	0.700
hsa-miR-376c-3p-478459_mir	0.286	2.416	5.335	0.700
hsa-let-7f-5p-478578_mir	0.291	3.319	9.977	0.700
hsa-miR-490-3p-478131_mir	0.309	3.630	12.376	0.700
hsa-miR-335-5p-478324_mir	0.331	0.702	1.626	0.700
hsa-miR-885-5p-478207_mir	0.368	1.841	3.583	0.717
hsa-miR-130a-3p-477851_mir	0.420	0.779	1.716	0.717
hsa-miR-25-3p-477994_mir	0.427	1.101	2.145	0.717
hsa-miR-433-3p-478102_mir	0.465	5.982	63.199	0.717
hsa-miR-32-5p-478026_mir	0.473	0.523	1.437	0.717
hsa-miR-29b-3p-478369_mir	0.505	1.975	3.930	0.726
hsa-miR-502-3p-478348_mir	0.509	2.712	6.554	0.726
hsa-miR-518e-3p-479408_mir	0.510	0.443	1.359	0.726
hsa-miR-744-5p-478200_mir	0.526	3.905	14.985	0.736
hsa-miR-31-5p-478015_mir	0.596	2.230	4.690	0.770
hsa-miR-26a-5p-477995_mir	0.616	0.398	1.318	0.780
hsa-miR-128-3p-477892_mir	0.705	0.494	1.408	0.852
hsa-miR-448-478105_mir	0.752	0.220	1.165	0.868
hsa-miR-486-5p-478128_mir	0.835	0.096	1.068	0.906
hsa-miR-495-3p-478136_mir	0.849	0.146	1.107	0.906
hsa-miR-429-477849_mir	0.854	0.247	1.187	0.906
hsa-miR-629-5p-478183_mir	0.932	0.244	1.184	0.960
hsa-miR-483-5p-478432_mir	0.966	0.223	1.167	0.973

Supplementary Table S4: Top consistently upregulated miRNAs. The table lists the top 25 miRNAs that were consistently upregulated in at least 3 of the 5 patients, selected based on the magnitude of their log2 fold change. The list is presented sorted by ascending p-value to highlight the most statistically significant candidates within this group. Columns include miRNA identifier, nominal p-value, log2 fold change (log2FC), and the False Discovery Rate (FDR) adjusted q-value.

miRNA	miRNA _log2FC	miRNA_ p_value	Target_Genes
hsa-miR-505-3p-478145_mir	-3.302	0.025	ACAP2;ACER2;ALYREF;AMFR;ANKRD13B;ANKRD13D;ASB1;ATG2B;BACE1;BAHD1;BEND4;BICD2;BMP2;BMS1;BTAF1;BTRC;C15ORF39;C5ORF64;CANX;CASTOR2;CBWD1;CBWD5;CCND2;CCT7;CD226;CD47;CDC5L;CHEK1;CLUH;COL4A1;CSE1L;CSF1;CSNK1A1;CTSZ;CWC25;DDX3X;DDX6;DGKH;DHX9;DIP2C;DMRT2;DNAJB9;DROSHA;EBNA1BP2;EDEM3;EEF1A1;EIF1AX;EIF6;ELF4;ELK3;EPHA4;FAHD2A;FAM160B1;FAM89A;FANCD2;FANCI;FBXL16;FBXO33;FIS1;FNBP1L;FUT10;FUT11;FZD5;GAS1;GGCX;GID4;GIGYF2;GJB7;GJD3;GNL3L;GPLD1;GRAMD4;GRK3;H3F3B;HDLBP;HIST1H4D;HIST2H4B;HMGN4;HNRNPA3;HNRNPF;HOXA9;HSPA1B;ID4;IGF1;IL20RB;INTU;IRS4;JARID2;KBTBD6;KCNH1;KCNMB4;KDM4A;KLHDC4;LGALS8;LIMS1;LSM4;LTBP4;MAML3;MAP1S;MAZ;MCFD2;MDC1;MICALCL;MKI67;MORF4L2;MTA2;MYBL2;MYBPC1;MYH10;MYO19;NABP2;NBPF8;NCK2;NDRG1;NEGR1;NPTX1;NUCKS1;NUDT16;OGFR;OPN5;ORC2;OSBP;OTUD5;PAICS;PDE4DIP;PET117;PISD;PJA2;PLEKHG3;PNMA2;POLG;POLR2D;PPP1CB;PPP1R13B;PRC1;PRDM10;PRICKLE2;PRKCA;PRRC2B;PSMD10;PTCD1;PTPRJ;PUS7;QSER1;RBM28;REST;RGS2;RHOH;RIMS4;RPL9;RPLP0;RPS15;RRP1B;RSRC1;RTL8C;RUFY3;SALL1;SCD;SEC13;SERBP1;SLC25A12;SLC5A12;SMAD2;SNAP29;SOCS5;SOD2;SOGA3;SOX11;SPINT2;SRSF1;SRXN1;STAMBP;SUB1;SYT4;TAF9;TCF7L2;TET1;TGFA;TGFBR2;THADA;THOP1;TLE3;TLK1;TM9SF1;TMEM132B;TMEM185A;TMEM37;TMOD3;TNFAIP3;TNPO1;TNRC6C;TP53BP1;TPBG;TPD52;TPRG1L;TREM1;TRIM28;TRIM4;TSC22D3;TUBB;TUBB2A;TUBB3;TXNRD1;TYW5;UBE2D3;UBE2O;UQCRFS1;UTRN;VBP1;VMP1;XIAP;XRCC6;YTHDC2;YTHDF1;ZBTB18;ZBTB9;ZFP64;ZNF208;ZNF445;ZNF511;ZNF652;ZNF845
hsa-miR-374a-5p-478238_mir	-4.523	0.028	ABCF1;ABCG2;ABHD18;ACSL1;ACSL4;ACSM2A;ACSM2B;ACTN4;ACVR2B;ACYP1;ADD2;ADD3;AFMID;AGL;AHSA2;ANGPTL3;ANKFY1;ANKRD12;ANKRD13C;APOL6;ARHGAP6;ARID2;ATM;ATP5L;ATP6V1G1;ATXN1;AVPR1A;BACH2;BHLHE40;BOLA3;BTBD3;BTBD7;C5ORF51;C8ORF33;C9ORF170;CA8;CAMSAP2;CASP3;CBX4;CCDC80;CCND1;CDKN2AIPNL;CEBPB;CENPK;CENPQ;CLIC4;CNTNAP5;CPEB4;CREB3L2;CTDSPL2;CXCL5;CYP4F11;DBT;DDX52;DDX55;DICER1;DLC1;DPP8;DPY19L1;DSN1;DUSP8;EIF2S3;EMC7;EP300;EPDR1;EPS15;ETNK1;FAM126A;FAM135A;FAM229B;FAT3;FKBP1A;FMR1;FOXC1;FXN;FZD5;FZD6;GABARAPL2;GABRG2;GADD45A;GALNT7;GIMAP4;GK5;GNL3;GNPDA2;GPR158;GSK3B;GSKIP;HECTD1;HHIP;HIST1H3B;HMGB2;HOXA11;HSBP1;HSPA13;IKZF2;JMJD1C;KCNC4;KIAA1468;KLHL15;KLHL9;KLRC3;KMT2A;KYAT3;L1CAM;LAMP3;LAMTOR3;LARP1;LDHA;LHFPL2;LIN28B;LIN7C;LMAN2;LMLN;LNPK;LRP8;LUZP2;MAPK7;MB21D2;MBNL2;MCM8;MFAP5;MIB1;MIER3;MIS18A;MLX;MPP5;MRPL18;MTF2;MYBPC1;MYLIP;NBPF11;NCOA6;NELL2;NF1;NFAT5;NFE2L2;NHLRC3;NLN;NNT;NPLOC4;NPM3;NR2C2;NR3C1;NSD1;NUFIP2;NUS1;OCRL;PANK3;PARD6B;PARP15;PBLD;PCNX1;PDE12;PDE4B;PELI1;PHACTR2;PIGW;PITPNC1;PITX2;PLAG1;PLEKHA2;PMEPA1;POLR1B;POLR3G;PRDM1;PRDX3;PRKCD;PRKX;PTAR1;PTBP1;PTMA;RAB21;RAB32;RAB5B;RAB8B;RAC1;RAI1;RALGDS;RANBP6;RAP1B;RBM26;RBMXL1;RBSN;RC3H1;RGS1;RIPOR2;RORA;RPL24;RPL38;SCAF4;SCOC;SDHD;SEC23B;SEC24A;SELENOI;SERBP1;SESN3;SESTD1;SETD5;SFT2D2;SHISA9;SHOC2;SIK3;SIM1;SKI;SLC16A1;SLC25A24;SLC25A27;SLC38A1;SLC9A4;SNRNP27;SNRPA1;SOCS6;SP1;SPC25;SRCIN1;SRSF7;SSX2IP;ST3GAL5;STAU1;STX16;STXBP2;SVIP;SYNGR2;SYP;TACC1;TAGAP;TET3;TFDP1;TFEC;TMEM241;TMEM245;TMTC1;TNFAIP3;TNFSF9;TNPO1;TNRC6A;TOP1;TOR2A;TPR;TRAF6;TRIM2;TRIM35;TRMT112;TRPS1;TRUB1;TTC8;UBE2G2;UBE3A;UBE4A;USP31;USP8;UST;VEGFA;VGLL2;WIF1;WNT5A;WWTR1;XKR4;XKR9;YEATS4;YME1L1;YOD1;YTHDF1;ZBED3;ZBTB7A;ZCCHC2;ZCCHC9;ZDHHC5;ZER1;ZFP37;ZNF148;ZNF217;ZNF260;ZNF383;ZNF384;ZNF443;ZNF566;ZNF681;ZNF99;ZSWIM6
hsa-miR-200a-3p-478490_mir	-7.272	0.044	A1BG;ADNP2;ANGPTL7;APOOL;AREG;ARL5B;ATRX;ATXN7L1;BAP1;BICD2;BICRAL;BRD3;C16ORF58;C18ORF25;CCDC18;CCDC71L;CCNE2;CDK6;CDV3;CELF1;CHML;CLDND1;COX6B1;CTNNB1;CYP1B1;DEK;DICER1;DLC1;DLX5;DNAJC28;DNMT1;DPY19L1;EGFR;ELAVL2;ELMO2;EPHA2;EPHA7;ERBIN;ERO1A;EZH2;FOXA2;FOXG1;FUT11;G3BP2;GATA6;GDAP1;GEMIN2;GJA1;GNA13;GRB2;H2AFZ;HFE;HGF;HMGB1;HNRNPAB;HNRNPF;HOXB5;HSPA4;IPO5;IRF2BPL;KEAP1;KIAA1147;KIAA1549;KIAA1549L;KLF11;KLF12;KLHL20;LHX1;LPP;MACC1;MALAT1;MALT1;MAPK14;MARCH6;MCL1;MDM4;MED13;MFF;MNX1;MYB;MYH10;NDST4;OGT;PCDH8;PDCD4;PEX11A;PGAM4;PGK1;PHB2;PIGW;POLR3F;PPP1R15B;PRELID2;PRKAA2;PSMD11;PSMD2;PTEN;PTPRD;QKI;RAB30;RAB8B;RAP2C;RASSF2;RBM28;RIN2;RPL12;SCD5;SEPT7;SEPT8;SHC1;SLC35D1;SMAD2;SMAD3;SPAG9;SPRYD4;SRF;STAT3;STXBP2;TCF7L1;TFAM;TFRC;TGFB2;THRB;TMOD3;TNFRSF10B;TNKS2;TP53;TP73-AS1;TRAM1;TRAPPC2B;TRMT112;TROVE2;UBASH3B;UQCRFS1;USP53;VAC14;VCAM1;WASF3;WDR37;XPOT;YAP1;YME1L1;YRDC;ZBTB34;ZEB1;ZEB2;ZFPM2;ZMAT3;ZNF292;ZNF621;ZNF805
hsa-miR-181b-5p-478583_mir	-3.940	0.045	ACYP1;ADAM17;ADCY9;AFF4;ALDH9A1;AP3M2;AP5M1;APOL6;ARF6;ARHGAP35;ARRB2;ARRDC3;ARSJ;ASB1;ASB13;ATG2B;ATM;ATP2B1;ATP8B1;ATXN7;BAZ2A;BCL2;BCL2L11;BLOC1S2;BMPR2;BPTF;BRINP2;C17ORF100;C20ORF173;C2ORF69;C5ORF34;CADPS2;CAPRIN2;CARD10;CARM1;CAT;CBX4;CBX7;CCDC88C;CCL22;CC

			NG1;CCNK;CCNQ;CDX2;CENPO;CHCHD7;CHD9;CHMP2B;CIP2A;CLCC1;CNOT9;CPEB4;CPOX;CREB1;CSNK1A1;CUL5;CYLD;DAZAP2;DCAF4;DCBLD2;DDHD1;DDIT4;DDX3X;DDX52;DIDO1;DNAJB11;DRAM1;DTD2;DUSP5;DYNC1LI2;E2F1;EED;EFCAB14;EIF3C;EIF4E2;EIF5A;ELK4;ELMSAN1;ELN;EMSY;EN2;EP300;EPS15;EPS8;ETS1;FAM13A;FAM160A2;FAM192A;FAM3C;FAM96A;FBL;FBXO11;FGFR1;FKBP14;FKBP1A;FKBP1C;FNDC3B;FOS;FOXL1;FSD1L;FUS;G3BP2;GANAB;GATA6;GIGYF1;GJB7;GK5;GNS;GOLGA1;GOLGA8B;GOT1;GRIA2;GRK2;GSKIP;GTPBP3;H1F0;HECW2;HEPHL1;HFM1;HIGD2A;HIST1H1E;HIST1H3D;HIST2H4B;HK2;HMGA2;HMGB1;HNRNPH1;HSP90B1;HSPA1B;HUWE1;ID4;IFNGR1;IGF1R;IL1A;ILF2;INCENP;INO80B;INO80D;INSL3;IPO5;IQCG;IRAK1BP1;KAT2B;KDM5A;KIAA1551;KIF2C;KIF3B;KLHL15;KLHL24;KLHL7;KMT2E;KPNA1;KPNA2;KPNA4;KPNB1;KRBOX4;KRI1;LAPTM4B;LATS2;LBR;LCLAT1;LDLR;LMAN1;LMCD1;LONRF1;LPCAT1;LRRC8D;MAN1A2;MAP2K1;MAP3K10;MAP3K3;MCL1;MED14;MEG3;MEPCE;MIGA2;MPP5;MRPL34;MRPS12;MRPS14;MRPS23;MT-ATP6;MTOR;MTUS1;MTX3;MYO1C;NAA50;NCAPG;NCOA3;NCOA7;NDC1;NDE1;NEK3;NFIA;NHLRC3;NIN;NLK;NMT2;NPM3;NR2F1;NR6A1;NRBP1;NRDC;NSD2;NUDT19;OSBPL3;OSBPL8;OTUD1;PAPD5;PBRM1;PBX3;PDCD10;PDCD4;PDHX;PDIA6;PDK3;PEBP1;PER2;PGAP1;PHC3;PHOX2B;PLAG1;PLPP3;PMAIP1;PMEPA1;PNKD;PNRC2;PPP2R5E;PRAMEF11;PRAMEF15;PRAMEF26;PRAMEF4;PRAMEF9;PRKCD;PRRC2C;PTBP3;PTEN;PTPDC1;PURB;PWP2;RAB2B;RAN;RAP1B;RAP1GAP2;RASSF1;RBM25;RBMX;RCOR1;RGS16;RHOG;RLIM;RNF187;RNF2;RNF6;RNMT;RPL13A;RPL18A;RPRD1A;RPS6KA3;RPS7;RPS8;RSBN1L;RSF1;RTFDC1;SCAMP2;SCAP;SCD;SCN8A;SEC24C;SEPT2;SHOC2;SIPA1L1;SIRT1;SIX2;SLC10A7;SLC19A2;SLC25A25;SLC25A37;SLC35G2;SLC35G3;SLC38A2;SLC7A1;SMCR8;SORT1;SPIRE1;SPP1;SPTLC3;SRGN;SRRM2;SSX2IP;STX2;SUV39H2;TAF5L;TBC1D13;TBC1D7;TBL1XR1;TCL1A;TEF;TFRC;THOC3;TIMP3;TLDC1;TMCC1;TMED7;TMEM189;TMEM30A;TMEM94;TMF1;TNFRSF11B;TNFSF11;TNPO1;TNRC6B;TOPBP1;TRRAP;TSG101;TTPAL;TUBB;TUBB2A;UBB;ULK1;UNC5B;USP10;USP24;VCAM1;VSNL1;WASHC5;WDFY3;WDR72;XIAP;XPNPEP3;XPO6;XRCC6;ZADH2;ZBTB20;ZBTB33;ZBTB4;ZC3HAV1L;ZDHHC15;ZFAND6;ZFHX3;ZFP36L1;ZFP36L2;ZFP69B;ZNF107;ZNF121;ZNF136;ZNF23;ZNF253;ZNF266;ZNF268;ZNF415;ZNF439;ZNF440;ZNF449;ZNF487;ZNF503;ZNF526;ZNF556;ZNF597;ZNF616;ZNF664;ZNF667;ZNF669;ZNF699;ZNF747;ZNF780B;ZNF781;ZNF788;ZNF791;ZNF829;ZNF83;ZNF844;ZNF846;ZXDC
hsa-miR-181c-5p-477934_mir	-2.684	0.062	ACYP1;ADAM17;ADCY9;AFF4;ALDH9A1;AP3M2;AP5M1;APOL6;ARF6;ARRB2;ARRDC3;ARSJ;ASB1;ATG2B;ATM;ATP2B1;ATP8B1;ATXN7;BAZ2A;BCL2;BLOC1S2;BMPR2;BTBD3;C2ORF69;CADPS2;CAPRIN2;CARM1;CBX4;CCDC88C;CCL22;CCNG1;CCNK;CCNQ;CDX2;CENPO;CHCHD7;CHD9;CHMP2B;CLCC1;CPEB4;CPOX;CSNK1A1;CUL5;DAZAP2;DCAF4;DCBLD2;DDIT4;DDX3X;DDX52;DPYSL2;DRAM1;DUSP5;DYNC1LI2;EED;EFCAB14;ELK4;ELMSAN1;EMSY;EN2;EPS15;EPS8;ETS1;FAM13A;FAM160A2;FAM192A;FAM3C;FAM96A;FBXO11;FKBP14;FKBP1A;FKBP1C;FNDC3B;FOXL1;FSD1L;G3BP2;GATA6;GIGYF1;GJB7;GK5;GNS;GOLGA1;GOLGA8B;GOT1;GRK2;GSKIP;GTPBP3;H1F0;HECW2;HEPHL1;HFM1;HIGD2A;HIST1H3D;HMGA2;HNRNPH1;HSP90B1;HSPA1B;ID4;IL1A;IL2;IL7;INCENP;INO80D;INSL3;IPO5;IQCG;IRAK1BP1;ISCA1;KDM5A;KIAA1551;KIF2C;KIF3B;KIT;KLF6;KLHL15;KLHL24;KMT2E;KPNA1;KRAS;KRBOX4;LAPTM4B;LATS2;LBR;LCLAT1;LDLR;LMAN1;LONRF1;LPCAT1;LRRC8D;MAN1A2;MAP3K3;MECP2;MIGA2;MPP5;MRPL34;MRPS14;MST1;MTUS1;MTX3;NAA50;NCAPG;NCOA3;NCOA7;NDRG2;NHLRC3;NIN;NLK;NMT2;NOTCH2;NOTCH4;NPM3;NR6A1;NSD2;NUDT19;OSBPL3;OTUD1;PAPD5;PBRM1;PDIA6;PDK3;PEBP1;PER2;PGAP1;PHC3;PHOX2B;PLPP3;PMAIP1;PMEPA1;PNKD;PNRC2;PPP2R5E;PRAMEF11;PRAMEF15;PRAMEF26;PRAMEF4;PRAMEF9;PRAP1;PRKCD;PRRC2C;PTBP3;PTEN;PTPDC1;PURB;RAB2B;RAN;RAP1B;RBM25;RCOR1;RGS16;RHOG;RLIM;RNF187;RNF6;RNMT;RPL13A;RPS6KA3;RSBN1L;RSF1;SAV1;SCAMP2;SCD;SCN8A;SEPT2;SGMS1;SHOC2;SIPA1L1;SIRT1;SLC10A7;SLC19A2;SLC25A25;SLC25A37;SLC35G2;SLC35G3;SLC38A2;SLC7A1;SMCR8;SORT1;SPIRE1;SPTLC3;SRGN;SSX2IP;ST8SIA4;STX2;SUV39H2;TBC1D13;TBC1D7;TBL1XR1;TEF;TFRC;TGFBR1;TGFBR2;TGFBRAP1;TIAL1;TLDC1;TMCC1;TMEM30A;TMEM94;TMF1;TNPO1;TNRC6B;TOPBP1;TRIM2;TSG101;TTPAL;TUB;TUBB;TUBB2A;UBR7;ULK1;UNC5B;VCAM1;WASHC5;WDFY3;WDR72;XPNPEP3;ZADH2;ZBTB33;ZBTB4;ZC3HAV1L;ZDHHC15;ZFAND6;ZFP36L1;ZFP36L2;ZFP69B;ZNF107;ZNF121;ZNF136;ZNF23;ZNF253;ZNF266;ZNF268;ZNF415;ZNF439;ZNF440;ZNF449;ZNF487;ZNF556;ZNF597;ZNF616;ZNF664;ZNF667;ZNF669;ZNF699;ZNF780B;ZNF781;ZNF788;ZNF791;ZNF829;ZNF83;ZNF844;ZNF846
hsa-miR-185-5p-477939_mir	-4.904	0.068	AADACL3;ABCC5;ABCG4;ACTN4;ADD2;AFF2;AGO1;AGO2;AHCYL2;AKR1B1;AKT1;AKT1S1;ALDH9A1;ALOX12;AMOTL2;ANKHD1;ANKRD45;AP3M1;APEX1;AQP3;ARC;ARHGAP39;ARHGEF6;ARID1A;ARID5B;ARIH1;ARL16;ARL9;ASH1L;ATAD3C;ATP5G2;ATP6AP1;ATR;ATXN1L;B4GALT1;B4GALT5;BACH1;BASP1;BHLHA15;BNC2;BTBD3;BTBD9;BTG2;C10ORF105;C11ORF74;C16ORF70;C6ORF106;CACHD1;CADM1;CALM3;CAMK2D;CAMK4;CAMKK2;CANX;CAPNS1;CAPZB;CASKIN1;CASP14;CBX6;CCDC86;CCDC88A;CCND2;CCNE1;CD180;CDC37;CDC42;CDK14;CDK6;CEBPB;CELF1;CENPU;CGN;CHRDL1;CHST11;CNOT6L;CNTNAP2;COCH;COL1A1;COPB1;CORO2B;CREB3L2;CSF1;CSNK1E;CTTN;CYFIP1;CYP2W1;DAZAP1;DCUN1D3;DEN

			ND2A;DIAPH1;DLG5;DLGAP4;DLX6;DNAJC10;DNAJC8;DNMT1;DPF1;DUSP4;DUSP7;E2F6;EDAR;EEF1A1;EEF2;EFNA1;EIF4H;EIF5AL1;ELAVL3;ELOVL1;EMC10;ENPP5;ENTPD7;EPAS1;EPHB2;EPS15L1;EZH2;FAM103A1;FAM210A;FAM228A;FAM241A;FAM53C;FAM83G;FASTK;FGFR1;FOSL1;FRMPD4;FZD7;G3BP1;GATA6;GCN1;GDF5OS;GFPT1;GFRAL;GIPC1;GLB1L3;GLI4;GLUD1;GLUL;GOLGA2P5;GPR107;GPR108;GPR137C;GRWD1;GSTA4;GXYLT2;HAAO;HACE1;HDX;HHIP;HIST3H2BB;HMGA1;HMGA2;HNRNPA0;HOXB6;HOXC6;HSD11B1L;HSP90AA1;IBTK;IFFO2;IGDCC4;IGF1R;IGFBP5;IKBKG;IL10RA;INCENP;IP6K1;IQSEC2;ISCU;ITM2C;KAT7;KBTBD3;KDM2A;KHSRP;KIAA0368;KIF6;KITLG;KLF16;LASP1;LDHB;LENG8;LNPEP;LPCAT3;LRP3;LUZP1;LY6H;MARS;MATR3;MCM10;MCRIP1;MDC1;MDM2;MED17;METTL22;MEX3A;MICAL2;MIEF1;MLF2;MORC4;MPV17;MRAS;MRE11;MRPL34;MRPS21;MSANTD1;MSL3;MYBL1;MYC;MYH14;MZB1;NAB2;NACC1;NEUROD1;NFATC3;NFE2L1;NRG4;NTRK3;OAS2;ONECUT3;PABPC1L2A;PABPC1L2B;PBX2;PDE4A;PDPK1;PDZD4;PFKFB3;PHACTR2;PHLDA2;PKD1;PKM;PKNOX2;PLAC8;PLAGL2;PLCD1;PLD3;PLEKHB2;PLPP3;POGK;POTED;POU2F1;PPP1CC;PPP2R1B;PPP2R5D;PRELP;PRRT2;PSME3;PTK7;PTMA;PTPA;RAB1B;RAB32;RAB40C;RAB5B;RAE1;RALBP1;RANBP10;RAPGEF1;RAPH1;RBM38;RBPJL;RHOA;RPP25;SAMD4B;SBK1;SCARB1;SCD;SELENON;SET;SETD1B;SF1;SFMBT2;SHISA9;SIGLEC10;SIX1;SLC25A28;SLC26A9;SLC29A2;SLC30A7;SLC31A1;SLC38A1;SLC6A17;SLC7A8;SLC8A1;SLC9A1;SLITRK5;SMAD7;SMG6;SMG7;SOCS4;SOD2;SPATA2;SRCAP;SRD5A3;SREBF1;SREBF2;ST6GAL2;STIM1;STK35;STK38;STX6;SUGT1;SUOX;SUPT4H1;SYT2;SYT7;TAGLN2;TAOK1;TBC1D13;TERB2;TEX261;TFDP1;TG;TGFB1;TGFBR3L;TIGD5;TIGIT;TLE3;TM9SF1;TM9SF4;TMEM109;TMEM160;TMEM184A;TMEM189;TMEM189-UBE2V1;TMEM214;TMEM86A;TMEM9;TMOD3;TMPRSS5;TNRC6A;TP53;TRIM29;TRRAP;TSPYL4;TUBB;TXLNG;UBA52;UBE2V1;UBR4;UHMK1;UMPS;USB1;USF3;VEGFA;VPS39;WDR77;WDTC1;WNT5B;WSB2;XPO6;ZADH2;ZBTB44;ZC3H11A;ZC3H12B;ZFYVE27;ZNF251;ZNF385A;ZNF444;ZNF512B;ZNF561;ZNF606
hsa-miR-125a-3p-477883_mir	-3.425	0.069	ABHD15;ABHD18;ADAT1;ADO;AGO2;AGO3;AK2;ALDOA;ALKBH5;ANKRD33B;ANKRD62;ANKS1A;APOH;AREL1;ARFGEF1;ARHGEF39;ARPC2;ARRB1;ARRB2;ASB8;ATCAY;ATP6V1B2;B4GALT1;BLCAP;BORCS5;BRCA1;BSCL2;BTF3L4;C14ORF144;C15ORF40;C1ORF43;C22ORF39;C2ORF72;CACNB2;CAPNS1;CASTOR2;CCR4;CDH6;CDK4;CECR2;CENPBD1;CFL2;CHCHD5;CLUAP1;CMBL;COL18A1;COL5A1;COLGALT1;COPS7B;CRKL;CTDNEP1;CWF19L1;CXORF38;CYB5D1;CYP20A1;CYTIP;DCUN1D2;DDX19B;DEGS1;DHTKD1;DLEU1;DPM2;DPYSL2;DPYSL5;E2F8;EIF2A;ELK1;EMP2;ENAH;EZH2;F2RL3;FAM131B;FAXC;FBLIM1;FBXL2;FFAR4;FLVCR1;FOXC1;FPR2;FXN;FXR1;FYN;GATAD1;GBA2;GIT1;GLTP;GLUL;GNAS;GNE;GPC4;GPHA2;GRIN2A;HCAR1;HES2;HGS;HIF1AN;HILPDA;HMGA1;HPCAL1;HSPA4;HSPA4L;HSPE1-MOB4;HUNK;IFNE;IGF2BP2;IGFBP5;IL23R;IL6;IP6K2;IRF1;IRF4;ITGAL;ITPRIPL2;IVD;KCNN1;KCNN3;KCTD5;KDM6B;KIF1C;KLB;LACTB;LASP1;LCE1A;LFNG;LGALS3BP;LIAS;LIPG;LMBR1L;MANEAL;MAPK14;MAST3;MELK;MFSD11;MFSD2A;MLLT10;MMAB;MOB4;MPEG1;MRPS10;MSANTD3;MTA1;MTAP;MTFR1;MTHFR;MUL1;MYC;MYOZ3;NAA50;NARS;NAV1;NECAB3;NFIC;NHLRC3;NOA1;NPHP1;NPHS1;NPTXR;NRG1;NT5C2;NUFIP2;NUP153;NUP155;NUP62;NWD1;OMD;OPA3;OSBPL10;OSTF1;P2RX7;P3H1;PACS2;PAG1;PALM2;PAX5;PBOV1;PDE4C;PDE6A;PDE7A;PFAS;PFKFB2;PGK1;PHLDA3;PIGG;PIGO;PIGP;PIGS;PIK3C2A;PISD;PLSCR1;PNMA8B;POLL;POLM;POLR3A;PPM1H;PRDM1;PRIM1;PRRT2;PRY;PRY2;PTPN3;RANGAP1;RBM27;RHOA;RNF19B;RPL13A;RPL30;RPL37;RPS6;RRAD;RTL10;RTN2;RUNDC1;S1PR3;SAR1B;SCIMP;SDK1;SETX;SGK1;SGTB;SHANK3;SIT1;SKI;SLC15A2;SLC1A5;SLC38A9;SMDT1;SMIM14;SMURF2;SMYD5;SNX2;SPATA5;SPIRE2;SS18L1;ST6GAL2;STAC2;STK4;STOML1;SUGT1;SUMO1;SWSAP1;SYNGR2;SYNJ2BP;TAOK1;TBC1D13;TERF2;THBS1;TIMM50;TIMM8A;TIRAP;TMCO1;TMEM132B;TMEM154;TMEM37;TMOD2;TMOD3;TOB2;TOR1B;TPM2;TRIM72;TRIP11;TRPV2;TSPYL1;TTC31;TTC4;TTC8;TTLL12;TXNDC16;VEGFA;VMAC;VPS36;WAC;WDR12;WDR31;WDR41;WDR5B;WDR77;WFDC6;WNT3;WNT7B;WSB1;XBP1;YPEL1;YWHAG;ZBTB43;ZBTB8B;ZC3H10;ZC3H7B;ZCCHC8;ZDHHC6;ZFP69B;ZIC5;ZKSCAN1;ZNF321P;ZNF333;ZNF37A;ZNF43;ZNF430;ZNF439;ZNF517;ZNF519;ZNF576;ZNF614;ZNF641;ZNF665;ZNF699;ZNF708;ZNF724;ZNF737;ZNF74;ZSWIM4
hsa-miR-338-3p-478037_mir	-2.242	0.075	ACOT9;ACVR1;ADAM17;ADCY7;ALG9;ALOX5AP;ALPK3;ANAPC13;ARHGEF28;ARL4C;ATAT1;B3GAT2;BEST3;BTBD9;C15ORF52;C1QTNF6;C2ORF71;C8A;C9ORF78;CAPN5;CATSPER4;CCDC14;CCND1;CDC42SE1;CDH2;DAB2IP;DHX8;DNAJB2;DSEL;EIF4EBP2;EMC2;FAM131B;FJX1;FOS;FUT4;GATA6;GPR132;GPR146;GPRC5A;GRIK3;GRK2;HES4;HIF1A;HIST1H2BJ;ID3;IRS2;ISPD;ITGB3;KIAA0513;KIAA0895;KIAA1210;KIFC1;LCOR;LMNB2;LRIG2;LRRC47;MACC1;MAP1A;MID1;MIER1;MMP2;MMP9;MNX1;MORF4L1;MRPL45;MRPS23;MSN;MTMR12;MVK;MYH9;MYLK4;NCS1;NLRP9;NOVA1;NRP1;NUP43;ORC4;PCDH7;PFAS;PHF3;PHF7;PKLR;PKM;PLA2G2C;PLA2G4B;PLEKHA1;PLXNA2;POLR2J3;PREX2;RFTN2;RLIM;RNF217;RNF8;RPL35A;RPL41;RPP25;RSRC1;RUNX2;SALL1;SCARA3;SCIMP;SCML2;SDHAF2;SGTB;SHROOM3;SLC25A34;SLC7A5;SLC7A7;SLFN12L;SMO;SOX4;SSR3;SSX2IP;STK25;TAPBP;TAZ;TENM3;TET3;TMEM199;TMEM74B;TOM1;TRADD;TRAF7;UBE2Q1;UPK3BL1;VHL

			L;VPS52;WDR55;WNT9A;XRCC2;ZBTB18;ZBTB39;ZBTB7B;ZDHHC18;ZEB2;ZFP91;ZKSCAN3;ZNF208;ZNF566;ZNF582;ZNF626;ZWINT
hsa-miR-140-3p-477908_mir	-5.160	0.076	ABL2;ACER2;ACVR2B;ADARB1;ADGRL1;AHCY;AIFM2;AMFR;ANKRD12;AP2M1;AP3S2;AP5Z1;ARID5B;ATP6AP2;ATP8A1;ATP8B1;ATXN1;BCAT1;BCL2L2-PABPN1;BOD1;BRMS1L;BTG2;C11ORF74;C15ORF38-AP3S2;C17ORF51;C3ORF52;CABLES1;CADM1;CAPN1;CAPN15;CAPZA1;CASP10;CBX6;CBX8;CCDC198;CD38;CD3EAP;CDC25A;CDCA4;CDCA8;CDK6;CHRDL1;CLDN7;CLDND1;CLIP1;CNN2;COL4A1;COMMD2;CORO7;CRY2;CTDSPL2;CXCL6;DAZAP2;DDA1;DDAH1;DDI2;DNAJC9;DRAXIN;EBPL;EMC8;EPB41;EPRS;ERAP2;ERCC1;ERRFI1;ESF1;ESR2;EVX2;FAM213B;FAM3D;FAM46B;FANCA;FAU;FDFT1;FN1;FOXK1;FPGS;FSTL3;FURIN;GAPVD1;GCNT3;GDF15;GDNF;GFPT1;GGT7;GJB7;GLMP;GPC1;GPR180;GPRIN2;GRAMD4;GUK1;HEATR5B;HMGN1;HMGN2;HNRNPA2B1;IBTK;IER3IP1;IL17REL;INTS14;IPPK;ITGA10;ITGA6;KCNMB1;KIAA0040;KIAA0907;KIF21A;KIF5A;KLF9;KSR1;LAMP2;LRPAP1;LRRC20;LYRM2;MAMLD1;MAP10;MAP3K3;MAPK8;MARC1;MARCKSL1;MAX;METRNL;MICB;MKKS;MKNK2;MLXIP;MOBP;MTDH;MTRNR2L5;MYL12A;MYO6;NANOS1;NCKAP1L;NEBL;NFASC;NFE2L1;NFYA;NRIP1;NUDT21;NUFIP2;NUP50;NWD1;ORMDL3;PABPN1;PALD1;PCYOX1;PDS5A;PITPNB;PITPNC1;PLCL1;POM121;PPP1R15A;PSAT1;PSMG1;PXDC1;RAB31;RACGAP1;RPL22;RPRD1A;RPS24;SCD5;SENP3;SERINC3;SERTAD4;SETD5;SHANK3;SKI;SLC16A10;SLC1A4;SLC23A1;SLC24A4;SLC2A5;SLC30A3;SLC39A10;SLC7A5;SNTB2;SPATA13;SS18;STC2;STUM;SUZ12;TADA3;TBC1D15;TBRG1;THRA;TMBIM6;TMED10;TMED7-TICAM2;TMEM109;TMEM133;TMEM189;TMEM189-UBE2V1;TMEM236;TMEM78;TNPO1;TNPO2;TPI1;TRIM31;TRPC4;TTC39C;TTLL12;TUBA1B;UBE2C;UBE2V1;UBTF;UCK2;USP14;USP46;VASH1;VKORC1L1;WDR26;WEE1;XPOT;ZBTB21;ZBTB22;ZBTB40;ZDHHC23;ZER1;ZNF207;ZNF423;ZNF431;ZNF460;ZNF490;ZNF518B
hsa-miR-361-5p-478056_mir	-2.299	0.102	AAR2;ABI2;ACLY;ACTG1;AFF1;AKR1B1;AKT3;ARCN1;ARF4;ASH1L;ATP6V1A;BACH1;BLMH;BSG;C8ORF46;CALCOCO2;CCDC58;CD46;CDC123;CDK2;CEBPG;CENPN;CENPQ;CHORDC1;CNOT2;COPS7A;CPOX;CXCR6;CYCS;DCAF4L1;DCTN1;DDX23;DEFB118;DEPDC1B;DOCK6;DTWD1;DUSP3;EAPP;ECD;EDC3;EGFL7;EHD2;EIF3C;EIF4A1;ENTPD1;EPGN;EPHA4;ERAL1;ERP44;FGFR1OP2;G3BP2;GPC6;GRK6;GSPT1;GTF2E1;HNRNPA2B1;HSF2;HSP90AA1;HSPA1B;HSPA4L;HUWE1;JKAMP;KCNJ3;KIAA1549L;KLHL15;KLHL28;KYNU;LHFPL3;LNPEP;MAGEF1;MAP3K9;MDC1;MDM4;MGARP;MSANTD4;MT-CO1;MTCH1;MTPAP;NAPG;NCEH1;NCOA6;NOP2;OGDH;PDE3A;PHACTR4;PIP4K2C;PLCL1;POMGNT2;PPA1;PPIL1;PRKCSH;PROSER2;PRPF8;PSMD2;PTCD3;QTRT2;RAB8B;RAC1;RAD23B;RAE1;RALGAPA1;RANBP17;RAVER1;RDH11;RNF141;RNF4;RPL28;RPS2;RPUSD4;SCARF1;SEMA6D;SETD1A;SGK1;SLC25A5;SLC30A5;SLPI;SMAD2;SMG1;SND1;SON;SRSF3;STAT6;SUPT16H;TEDC1;TFIP11;TMEM18;TMEM185B;TMEM246;TMEM62;TNRC6A;TP53BP1;TSR1;TWIST1;U2SURP;UCK2;USP22;VBP1;VCP;VCPIP1;VEGFA;VEGFB;WBP2;WDR36;WIPF2;WWTR1;XRCC5;YOD1;ZBTB10;ZBTB18;ZBTB33;ZBTB7A;ZC3H12C;ZMAT3;ZMYND11;ZNF154;ZNF207;ZNF460;ZNF554;ZNF776;ZNF92
hsa-miR-181a-5p-477857_mir	-3.766	0.120	ABCG2;ACAN;ACOT12;ACYP1;ADAM17;ADCY9;AFF4;AFTPH;AHR;AKAP12;ALDH1A1;ALDH9A1;AMMECR1;ANKRD1;ANKRD13C;AP1M1;AP3M2;AP5M1;APOL6;ARF6;ARHGAP12;ARL6IP6;ARRB2;ARRDC3;ARSJ;ASB1;ATF7IP2;ATG10;ATG2B;ATG5;ATM;ATP2B1;ATP6V0E1;ATP8A1;ATP8B1;ATXN7;BAG2;BAX;BAZ2A;BCL2;BCL2L11;BLOC1S2;BMP3;BMPR2;BPGM;BRCA1;BRMS1L;BTBD3;C12ORF29;C1ORF109;C1QTNF9;C2ORF69;C8A;CADPS2;CAPRIN2;CARM1;CBX4;CCDC6;CCDC88C;CCL22;CCNG1;CCNK;CCNQ;CD4;CD46;CDH13;CDKN1A;CDKN1B;CDX2;CEBPA;CENPO;CFI;CHCHD7;CHD9;CHL1;CHMP2B;CHRFAM7A;CLCC1;CLUAP1;COL16A1;COL27A1;COPS2;CPEB4;CPOX;CSNK1A1;CST5;CTDSPL;CTNNB1;CUL5;DAZAP2;DCAF4;DCBLD2;DCST1;DDIT4;DDX27;DDX3X;DDX52;DNAJC7;DRAM1;DSCR8;DUSP5;DUSP6;DYNC1LI2;E2F5;EED;EFCAB14;EGR1;ELK4;ELMSAN1;EMSY;EN2;ENAH;EPHA5;EPS15;EPS8;EREG;ETS1;EYA4;FAM13A;FAM160A2;FAM192A;FAM222B;FAM3C;FAM47B;FAM96A;FAT1;FBXO11;FBXO28;FBXO33;FBXO34;FKBP10;FKBP14;FKBP1A;FKBP1C;FKBP7;FNDC3B;FOS;FOXL1;FRA10AC1;FSD1L;FSIP1;FXYD6;G3BP2;GADD45G;GANAB;GATA6;GATAD2B;GCNT1;GIGYF1;GJB7;GK5;GNAI3;GNS;GOLGA1;GOLGA8B;GOT1;GPD1L;GPR137B;GPR78;GPR83;GPRIN3;GRK2;GSKIP;GSTM2;GTPBP3;H1F0;H2AFY;H3F3B;HACD3;HDAC6;HECW2;HEPHL1;HERC3;HEY2;HFM1;HIGD2A;HIPK2;HIST1H3D;HMGA2;HMGB2;HNRNPAB;HNRNPH1;HOOK3;HRAS;HSD17B3;HSP90B1;HSPA13;HSPA1B;HUWE1;ID4;IDS;IFNG;IL1A;INCENP;INO80D;INPP4B;INSL3;IPO5;IQCG;IRAK1BP1;JCAD;KAT2B;KBTBD3;KCTD2;KCTD3;KDM5A;KIAA0100;KIAA1551;KIF2C;KIF3B;KLF6;KLHL15;KLHL24;KLHL42;KLRC4;KMT2E;KPNA1;KRAS;KRBOX4;LAMA3;LAPTM4B;LBR;LCLAT1;LDLR;LFNG;LGALSL;LMAN1;LONRF1;LPCAT1;LPGAT1;LRRC17;LRRC8D;LRRN3;LYSMD3;MADD;MAN1A2;MAP2K1;MAP3K10;MAP3K3;MAP4K4;MAPK1;MAPK1IP1L;MAZ;MCL1;MEG3;METAP1;MGAT5;MIGA2;MMP14;MOB1A;MOB1B;MOB3B;MOSPD1;MPP5;MRPL34;MRPS14;MT-ND2;MTCL1;MTMR12;MTMR3;MTUS1;MTX3;NAA50;NCAPG;NCOA3;NCOA7;NFY

			B;NHLRC3;NIN;NKX3-2;NLK;NMRK2;NMT2;NOL4;NOTCH1;NOTCH2;NPM3;NR6A1;NRAS;NRP1;NSD2;NUDT12;NUDT19;NUP58;OCA2;OFCC1;OR11A1;OSBPL3;OTUD1;OTX2;PABPC1;PAPD5;PBRM1;PBX3;PCDHA1;PCDHA10;PCDHA11;PCDHA12;PCDHA13;PCDHA2;PCDHA3;PCDHA4;PCDHA5;PCDHA6;PCDHA7;PCDHA8;PCDHAC1;PCDHAC2;PCDHB6;PCDHB8;PCLAF;PDAP1;PDGFRA;PDIA6;PDK3;PEBP1;PER2;PFKFB2;PGAP1;PGD;PGR;PHACTR2;PHACTR4;PHC3;PHLDA1;PHLPP2;PHOX2A;PHOX2B;PHPT1;PITPNB;PLA2G4C;PLAG1;PLCL2;PLPBP;PLPP3;PLXDC2;PMAIP1;PMEPA1;PNKD;PNRC2;PPP1R9A;PPP2R5E;PPP3CA;PRAMEF11;PRAMEF15;PRAMEF26;PRAMEF4;PRAMEF9;PRAP1;PRDX3;PRKCD;PRKN;PRLR;PROX1;PRR4;PRRC2B;PRRC2C;PTBP3;PTEN;PTGS2;PTPDC1;PTPN11;PTPN22;PTPRZ1;PUM1;PURB;RAB2B;RALA;RAN;RAP1B;RASSF1;RASSF6;RBM25;RCOR1;RGS16;RGS5;RHOG;RLIM;RNF187;RNF2;RNF34;RNF6;RNMT;ROPN1L;RPL13A;RPL14;RPS6KA3;RPS8;RSBN1L;RSF1;RTEL1-TNFRSF6B;RUNX1;S100A1;SAMHD1;SASH1;SCAMP2;SCD;SCN8A;SEPT2;SH3BGRL;SHOC2;SIK2;SIPA1L1;SIRT1;SIX6;SLC10A7;SLC19A2;SLC25A25;SLC25A37;SLC35B4;SLC35G2;SLC35G3;SLC37A3;SLC38A2;SLC7A1;SLC7A11;SLCO2A1;SMAD2;SMCHD1;SMCR8;SMG1;SNAI2;SORT1;SOX5;SPIRE1;SPRY2;SPTLC3;SRGAP1;SRGN;SRPK2;SRSF7;SSX2IP;STAG2;STAT3;STX2;SUV39H2;TAAR6;TAB2;TAB3;TAF15;TAF2;TBC1D13;TBC1D7;TBL1XR1;TBX4;TCF21;TCF4;TEAD4;TEF;TERT;TFRC;TGFBR1;TGFBR3;TGFBRAP1;TGIF2;TIAL1;TIMP1;TLDC1;TM9SF3;TMCC1;TMED4;TMEM132B;TMEM14A;TMEM192;TMEM30A;TMEM45A;TMEM64;TMEM94;TMF1;TMPRSS11A;TNFRSF11B;TNIP1;TNPO1;TNRC6B;TOPBP1;TRIM2;TSG101;TSHR;TTPAL;TUBB;TUBB2A;TUSC1;TUSC3;TWIST1;UBA2;UBL3;UCHL1;UGT3A1;ULK1;UNC5B;USP28;VCAM1;VEGFA;WASHC5;WDFY3;WDR72;WIF1;WNT16;WNT2;WNT3A;XIAP;XPNPEP3;YOD1;ZADH2;ZBTB33;ZBTB4;ZC3HAV1L;ZDHHC15;ZEB2;ZFAND6;ZFP36L1;ZFP36L2;ZFP69B;ZNF107;ZNF12;ZNF121;ZNF136;ZNF148;ZNF23;ZNF25;ZNF253;ZNF266;ZNF268;ZNF35;ZNF350;ZNF415;ZNF426;ZNF439;ZNF440;ZNF445;ZNF449;ZNF487;ZNF556;ZNF558;ZNF562;ZNF594;ZNF597;ZNF616;ZNF652;ZNF664;ZNF667;ZNF669;ZNF699;ZNF763;ZNF780B;ZNF781;ZNF788;ZNF791;ZNF829;ZNF83;ZNF844;ZNF846
hsa-miR-501-3p-478350_mir	10.558	0.137	AGBL5;B2M;C8ORF33;CA12;CDC27;CDK6;COL23A1;CRK;CSRP1;CYP4F11;DENND4B;EFCAB11;ELAVL2;EMP2;ENTHD1;FAM219B;FCN2;FDXR;FRS2;FSCN1;GTPBP2;IBA57;IL21R;KCNJ6;KMO;LINC00632;LINC00955;LRRC58;LYSMD3;MANBAL;MCM2;MCM4;MRRF;MSI1;MTHFD2;NAA30;NCAPG2;NLGN4X;NPTXR;OIP5;PEG10;PLCB1;PLEKHB2;PSMG1;RBM3;RPL4;SESN1;SIGLEC15;SLC2A12;SMIM13;SNRPD3;SOCS4;SOD2;SSTR1;STARD7;TBL1XR1;TLCD2;TMEM135;TNIP3;TOMM40L;TRDN;TRIM72;UBE2H;UGT2B10;WDR82P1;WSB1;ZBTB43;ZC3H12C;ZNF460
hsa-miR-145-5p-477916_mir	2.263	0.149	AAED1;ABCC1;ABHD17C;ABRACL;ACTB;ADAM17;ADD3;AGPS;AGTRAP;AKR1B10;ALDH3A1;ALG9;ALPPL2;ANGPT2;ANKRD28;AP1G1;APH1A;AQR;ARF6;ARL6IP5;BCLAF3;BNIP3;BRAF;BTG1;C11ORF65;C1ORF27;CAMK1D;CBFB;CCDC43;CCDC80;CCDC85C;CD28;CD40;CD44;CDH2;CDK4;CDK6;CDKN1A;CEP19;CFTR;CLINT1;CLSTN2;COL5A1;CPEB4;CRAMP1;CRNDE;CRYBG1;CSRNP3;CTGF;CTNNBIP1;CTNND1;CYP2C19;DDC;DDI2;DDX17;DDX6;DEK;DFFA;DMXL1;DNAJC28;DTD1;DUSP6;E2F3;EGFR;EID2B;EIF4E;EPAS1;ERBB4;ERG;ESR1;ETS1;F11R;FAM3C;FAM45A;FLI1;FSCN1;FXN;FZD6;FZD7;GMFB;GOLM1;HBEGF;HDAC11;HDAC2;HIF1A;HIST1H2AH;HIST1H2BF;HLTF;HMGA2;HOXA9;IFNB1;IGF1R;IGFBP5;ILK;IMMP2L;IRS1;IRS2;ITGB8;IVNS1ABP;JADE1;JAG1;KIF21A;KLF4;KLF5;KREMEN1;KRT7;LMNB2;LYPLA2;MAP1LC3B;MAP2K4;MAP2K6;MAP3K11;MAP3K3;MAP4K2;MCM2;MDM2;MEST;MIXL1;MMP1;MMP12;MMP14;MSH3;MTDH;MTMR14;MUC1;MUC19;MUC4;MYC;MYO5A;MYO6;MYOCD;MYRF;NAIP;NANOG;NDRG2;NDUFA4;NDUFS2;NEDD9;NFATC1;NIPSNAP1;NR1D2;NRAS;NUDT1;NUFIP2;NUP43;ORC4;P4HA1;PADI1;PAK4;PANK1;PARP8;PDGFD;PHACTR2;PIGF;PLAGL2;PLEKHM1;PNMA3;PODXL;POU5F1;PPM1D;PPP3CA;PRDM2;PSAT1;PTP4A2;PXN;RAB3IP;RBM18;REL;ROBO2;ROCK1;RPA1;RPS6KA3;RPS6KB1;RRAGC;RREB1;RTKN;SAMD5;SENP1;SERINC5;SERPINE1;SESN2;SET;SLC16A10;SLC16A5;SLC22A9;SLC26A2;SMAD2;SMAD3;SMAD4;SMAD5;SMIM17;SNTB1;SNX24;SOCS4;SOX11;SOX2;SOX9;SP1;SP7;SPTBN1;SPTLC1;SRGAP1;SRPX2;STAT1;SWAP70;TGFB2;TGFBI;TGFBR2;THSD7A;TIRAP;TMEM9B;TMOD3;TNFSF13;TNR;TPM3;TPRG1;TSPAN6;TUG1;UBR7;UBXN2A;UNC5D;UTP15;VEGFA;VGLL4;VPS51;WASHC2C;WSB1;YES1;ZBTB25;ZFAND3;ZFYVE9;ZNF100;ZNF426;ZNF445;ZNF451;ZNF660;ZNF678;ZNF772
hsa-miR-425-5p-478094_mir	-2.155	0.153	ACACB;AFF4;AP3D1;ARIH1;ASCC3;ATP5G3;BAZ2A;BCOR;BEX4;BHLHB9;BIRC5;BTK;CAND1;CCDC144NL;CCND1;CDC25B;CELF2;CERS6;CKS1B;CLU;COPS7B;COX6B1;CREBZF;CRKL;CSNK1G1;CYB561;DDX6;DENR;DHCR7;DICER1;DPYSL2;E2F3;EIF3C;EIF3G;EOGT;EPM2AIP1;EXOG;FAM114A1;FAM122A;FAM122B;FAM196B;FAM89A;FGFR3;FOXJ3;FOXK2;FOXN3;FRY;GAN;GNB5;GSK3B;HDGF;HSP90AA1;IGSF1;INPP5E;KAT6A;KIFAP3;LCOR;LDHA;LDHD;LRP11;LRRC40;MAFB;MAP2K6;MAP3K5;MDM2;MED22;MED4;MET;MIDN;MYPN;MZT1;NADK;NPNT;NRAS;OCRL;ORMDL2;PDCD10;PDZD8;PIP4K2A;PLOD2;PLSCR4;PLXNA3;PNMA8A;PPAT;PPP1R15B;PPP2CB;PPP4R3A;PPRC1;PRR13;PSMD8;PTEN;QKI;RAB31;RABL6;RBM8A;RNF168;RPL7L1;RPS21;RPS28;RRM2;RUFY2;RUSC1;SDHAF2;SFT2D1;SH3PXD2A;SHCBP1;SHROOM1;SIGLEC14;SLC16A1;SLC25A3;SMAD2;SMARCD1;SNRNP200;SOD2;SPP

			L2A;SPRED1;SSX2;SSX2B;STMN1;SYNCRIP;TACC3;TAOK1;THOP1;THRB;TNFRSF10B;TTC39B;TXN2;USMG5;VPS39;WASF2;WDR6;WRB;YARS;YRDC;ZBTB10;ZBTB21;ZBTB34;ZC3H11A;ZHX3;ZNF148;ZNF18;ZNF417;ZNF700;ZNF805
hsa-miR-125a-5p-477884_mir	-3.872	0.159	ABL2;ADAM9;AGTRAP;AKT1;ANAPC16;ANKRD33B;ARHGAP12;ARID3A;ARID3B;ARIH2;ARPC5;ASB16;ATL2;ATP5G2;ATP5J2;ATXN1;ATXN7L3;B4GALT1;BACE2;BAK1;BCL2;BCL2L12;BTG2;C17ORF80;C1D;C2ORF15;CASC4;CASP2;CCL5;CD244;CD34;CDKN1A;CENPP;CGNL1;CLCN7;CLDN12;CLEC5A;COIL;COL4A1;COPZ1;CORO1C;CREB5;CRK;CSNK2A1;DHX33;DIP2A;DLL4;DLST;DOCK3;DUSP3;DYRK2;E2F3;E2F7;EDN1;EEF1A1;EGFR;EHD1;EIF1AD;EIF4E;EIF4EBP1;ELAVL1;ELOB;EMID1;ENO1;ERBB2;ERBB3;ERMP1;ESRRA;ETNK2;FAM129B;FAM174B;FBXL18;FBXO10;FERMT1;G2E3;GALNT14;GGCT;GJB7;GOLGA1;GOLGA8A;GOLGA8B;GPAT4;GRHL1;GSS;GUCD1;HAX1;HDAC4;HDAC5;HID1;HK2;HMGCLL1;HOXC4;HOXD1;HRH4;IFNG;IFRD2;IL1RN;IP6K1;IPPK;IRF4;JAK2;KIAA0232;KIF6;KLF13;KRTAP5-9;LAMB1;LBX2;LFNG;LIF;LIFR;LIN28A;LIN28B;LINS1;LONRF2;LSM4;LYPLA1;MACF1;MAP2K7;MAP3K1;MAPK14;MAPK8;MASP1;MAT2A;MCL1;MEG3;MEGF9;MFSD9;MKNK2;MMP11;MON1B;MORC2;MRPL50;MTOR;MTUS1;MYC;MYCBP2;NAIF1;NBPF11;NCKIPSD;NDEL1;NDUFB6;NEGR1;NF2;NIN;NME2;NNT;NOP16;NPM1;NR1D2;NTRK3;NUP37;OGT;OMA1;OPRL1;OSBPL9;OSTM1;OTUB2;PANK1;PANX1;PARD6B;PC;PCDHGB2;PCTP;PDCL3;PDPK1;PEG10;PFKM;PGM3;PIK3CG;PLA2G4F;PLEKHG5;PLS3;PLXDC1;PPP1R37;PPP2CA;PPP2R5E;PRC1;PRDM1;PREPL;PTBP1;PTGS1;PUM2;RAB4A;RAF1;RALBP1;RARA;RBAK;RBM17;REST;RIT1;RPL35A;RPL9;RPS2;RRP1B;RSG1;RTN2;SCARB2;SEC14L3;SELENOW;SEMA4B;SEMA4C;SGPL1;SH3BP5L;SIRT7;SIX1;SLC1A5;SLC35G1;SLFN11;SMAD2;SMAD4;SMC2;SNX4;SP1;SPATA5;SPHAR;SPRTN;SRGAP2;STAT2;STAT3;STOX2;TACC2;TAZ;TBC1D1;TDG;TEF;TFRC;THRAP3;TMEM101;TMEM136;TMEM59;TMEM63C;TNFAIP3;TNFRSF10B;TNPO2;TNPO3;TOR1AIP2;TOR2A;TOR4A;TP53;TP53INP1;TPI1;TPM4;TRAF6;TRMT2A;TRPS1;TTC30B;UBE2G2;UBE2L3;UBR7;UNC45A;VEGFA;VPS51;WNK3;XPO1;YES1;YIPF4;YOD1;ZBTB10;ZBTB7A;ZDHHC9;ZEB2;ZFAND1;ZMYND19;ZNF177;ZNF385A;ZNF776;ZSWIM6;ZZEF1
hsa-miR-503-5p-478143_mir	-2.065	0.159	ACTR2;ACTR3B;AGO1;AHSA1;AKT3;ANK3;ANLN;AP2B1;ARHGEF19;ASCC1;ASH1L;ATAD5;ATF6;ATP5G3;ATP6V1B2;B4GALT5;BCL2;BCL2L12;BTN3A3;BZW1;C1ORF21;CA8;CACUL1;CANX;CAPZA2;CASK;CBX2;CBX4;CCDC83;CCND1;CCND2;CCND3;CCNE1;CCNE2;CCNF;CCNG2;CCNT1;CD2AP;CD40;CDC14A;CDC25A;CDC37L1;CDC42SE2;CDCA4;CDK12;CDK17;CDKN1A;CERK;CHAC1;CHEK1;CLTC;CMTM4;CNKSR3;CNNM2;COL1A1;CREBL2;CREBRF;CRK;CTDSPL;CTSA;CTSD;CUL3;CUZD1;CYP26B1;DCAF10;DCAF17;DCLRE1A;DCTN5;DDHD2;DDX3X;DECR1;DHFR;DLGAP3;DLST;DMTF1;DNAH17;DNAJC10;DYNLL2;E2F3;EFNB2;EIF4H;ELMSAN1;EPOR;EXT1;FAM229B;FANCA;FARSA;FBXL18;FBXW7;FGF2;FGF8;FGFR1;FOXK1;GLP2R;GNAT1;GPR27;GREM2;GSG1;HCFC2;HDGF;HEXIM1;HNRNPA1;HNRNPA1L2;HNRNPK;HSPA8;HSPE1-MOB4;IGF1R;IKBKB;INSL6;IVD;JARID2;KANK1;KIAA0895L;KIAA1456;KIAA1671;KIF23;KIF5B;KPNA3;L2HGDH;LANCL1;LARP1;LIX1L;LRWD1;LURAP1L;MAP3K7;MAP4K2;MCM7;MOB4;MRGBP;MRPL10;MRPS23;MT1E;MTMR3;MTPN;MYB;MYO5A;N4BP1;NAPG;NDUFA4P1;NNT;NPLOC4;NR6A1;NT5DC3;NUCKS1;NUFIP2;NUP50;OCRL;ODF2L;ORC4;OSCAR;PAPOLG;PHKA1;PI4K2B;PIK3R1;PIK3R4;PISD;PLAG1;PLEKHA1;PNPLA6;PNRC2;PPM1A;PPP1R11;PPP2R5C;PRDM4;PRKAR2A;PRSS21;PSAT1;PTPRD;RAB3IP;RALGAPB;RAPH1;RCC1L;RECK;REL;RFK;RNF149;RNF41;RPL15;RPL18A;RPL23;RPS5;RPS6KA3;RS1;RTN4;SALL1;SBF1;SBNO1;SCAMP4;SEC16A;SEC24A;SEC61A1;SESTD1;SETD1B;SF3B3;SIAH2;SKI;SLC25A12;SLC2A3;SLC33A1;SMAD2;SMAD7;SNRPB2;SNTB2;SOCS5;SPNS1;SPRED1;SREK1;SRPRA;SZRD1;TAOK1;TFAP2A;TLK1;TLL1;TMEM100;TMEM245;TNFRSF11A;TOMM20;TRAK1;TRAPPC10;TRIM35;TSC22D2;TUT1;TXNDC5;UBFD1;UBN2;UBR3;UGT2B4;UHMK1;USP48;USP53;VEGFA;VOPP1;WEE1;WIPI2;WNK3;XKR7;YRDC;YTHDC1;ZBTB34;ZBTB44;ZFHX4;ZMAT3;ZNF282;ZNF367;ZNF449;ZNF620;ZNF622;ZNF691;ZNF704;ZNRF2;ZNRF3
hsa-miR-516b-5p-478979_mir	2.925	0.160	ADAMTS5;AGTR2;ANXA6;B3GNT2;BSG;C1ORF109;CANX;CCDC140;CCDC158;CCND1;CD226;CD274;CLCN3;CLDND1;CNOT6;CORO2A;DCAF4;DPY19L3;EHD3;ELK4;FSD2;GNAS;GXYLT2;GYS1;HRK;IL6ST;KBTBD13;KLHL15;KLHL23;KPNA6;LCE1A;LRIG3;LRRC32;LUZP1;METTL8;MT1A;MYO18A;MYRF;NACC2;NASP;NCBP3;NCLN;NDST1;NFIX;NUP133;OCA2;PATZ1;PIGS;PLCG1;PLD5;PPP1R11;PPP1R13L;PRRX1;RAB34;RBM23;RBM47;RBMS3;RSL1D1;RTL8A;SLF1;SMIM12;SMPD1;STARD13;TBL2;TIAF1;TMEM151A;TMEM86B;TMPRSS4;TMTC3;TRIB3;TRIM65;UBBP4;VAMP3;WTAP;YOD1;YY1;ZC3H4;ZFP36;ZFP62;ZNF326;ZNF354B;ZNF850;ZNFX1;ZSWIM4
hsa-miR-29a-3p-478587_mir	-3.754	0.173	ABCE1;ABL1;ADAM12;ADAMTS9;AGAP1;AGO1;AHR;AKT2;AKT3;ALDH5A1;AMER1;AMFR;ASXL2;ATG9A;BACE1;BBC3;BCAP31;BCL2;BCL7A;BMT2;BTG2;C1QTNF6;C21ORF91;C4ORF26;CACNA1C;CALCR;CALM3;CAND1;CASP8;CBX2;CBX6;CCDC117;CCDC14;CCNA2;CCND1;CCND2;CCNT2;CCT4;CD276;CD93;CDC23;CDC42;CDC42SE1;CDC7;CDK2;CDK4;CDK6;CEACAM6;CLDN1;CNBP;COL10A1;COL1A2;COL3A1;COL4A1;COL4A2;COL5A2;COLEC10;COMMD2;COX7A2L;CPEB3;CPEB4;CRKL;CRYBG1;CSRNP2;CTC1;CTNNBIP1;CYP2C19;DAG1;DDX6;DENND6A;DIABLO;DIC

			ER1;DKK1;DNMT1;DNMT3A;DNMT3B;DOT1L;DSC2;DUSP2;DYNLT1;EDC3;EIF3E;ELMSAN1;ELN;EMP1;ENPP2;ENTPD1;EPHX2;EREG;FAM102B;FAM109B;FAM133A;FAM193A;FAM53C;FAM71F2;FBN1;FBRS;FEM1B;FGA;FGB;FGG;FJX1;FMNL3;FOS;FOXO3;FRK;FSCN1;FSTL1;GAREM2;GAS2L3;GLDN;GLRX3;GLUL;GOLGA7;GPR85;GSK3B;GTDC1;HBP1;HDGF;HECW1;HMGCR;HNRNPM;HUWE1;ID3;IFNAR1;IFRD1;IGF1;IMPDH1;INSIG1;ISG20L2;ITGA11;ITGA6;ITGB1;ITIH5;KCTD15;KDM5B;KDM6B;KEAP1;KLF4;KLHDC3;KMT5C;KREMEN2;LAMA2;LAMC2;LIMS1;LOX;LPL;MAPKBP1;MAZ;MCL1;MDM2;METTL15;MMP2;MORF4L1;MORF4L2;MRM3;MUC1;MXD1;MYC;MYCN;NAA40;NASP;NAV3;NEDD9;NFIA;NKIRAS2;NMI;OAZ1;OTUD4;P3H1;PAN2;PDGFRB;PER1;PHACTR2;PIGS;PIK3R1;PLAG1;POLD3;PPM1D;PPP1R13B;PPT1;PPY;PRY;PRY2;PTEN;PTP4A1;PXDN;QKI;R3HDM4;RAB11FIP1;RAB40C;RAET1L;RAN;RASGRP1;RCC1L;REL;REST;RET;RHBDD1;RIOK3;RNASEL;RNF138;ROBO1;RPL22;RPS4X;RPS6KA3;RRAGC;RTL6;S100B;SAPCD2;SARS;SEC31A;SERPINB9;SERPINH1;SETDB1;SFRP2;SGK1;SH3GLB1;SLC16A1;SLC22A7;SLC29A2;SLC2A14;SLC30A10;SLC7A5P2;SNX24;SP2;SPARC;SPRTN;SRGAP2;SURF2;SYNCRIP;TDG;TESPA1;TET1;TET2;TET3;TFEB;TGFB3;TMEM237;TMTC3;TNFAIP3;TNRC18;TRAF4;TRAM2;TRIM63;TRIM68;TRIM72;TUBB2A;UBE2Q1;ULBP2;VDAC1;VEGFA;VHL;WDR26;WWTR1;YAE1D1;YY2;ZBTB34;ZBTB5;ZFP36;ZFP91;ZFPM1;ZNF286A;ZNF850
hsa-miR-100-5p-478224_mir	-4.189	0.173	ABLIM1;ACKR3;ACSL3;ACTB;ACTR1B;AGPAT3;AKT1;ALDH9A1;ALG3;ANAPC1;ANAPC11;AP3M1;APBA3;APEX1;ARPP19;ATAT1;ATM;ATP1A2;ATP1B3;ATP2A2;ATP5A1;ATP5L;ATP6AP1L;BAZ1B;BCL10;BECN1;BMPR2;C21ORF91;C9ORF66;CAMKK2;CAPNS1;CBX3;CCNG1;CCNYL1;CCZ1B;CDC73;COL4A1;COX5A;CREBBP;CTC1;CTDSPL;CTDSPL2;CUL1;CUL2;CUL5;CYR61;DAZAP2;DCAF6;DCBLD1;DDAH1;DDX21;DEAF1;DNMT1;DOLK;DPY19L4;EDEM1;EEF1A1;EGR2;EIF5A;EIF5AL1;ESR1;EWSR1;FAM221A;FARSB;FBXL15;FGFR3;FKBP5;FLT1;FOXN2;FOXP1;FXN;GDE1;GFOD1;GNA13;GNG5;GOSR1;GRB2;GRHL1;GTF2H1;GTPBP1;H2AFV;H2AFY;H3F3A;HACD3;HIST1H4C;HIST2H2AA3;HIST2H4B;HMGB1;HMGN1;HOXA1;HS3ST2;HSPA14;IARS;ID1;IFIT3;IGF1R;IGF2;INSIG1;ITM2B;JPH1;KANK1;KATNBL1;KBTBD8;KDELR2;KLHL15;KPNA2;LDHB;LIN28B;LIX1L;LRRC41;MAFG;MAP4;MAP7D1;MAPK1IP1L;MAPK6;MARC1;MBIP;MED12;MITD1;MKLN1;MMP13;MORF4L2;MRPS33;MT-CO2;MTO1;MTOR;N4BP2;NCOR2;NDE1;NDUFA2;NDUFA9;NDUFC2;NFIA;NIP7;NLRP3;NOP2;NSF;NTAN1;NUDCD2;ODC1;ORC2;ORC5;ORMDL1;OXA1L;OXCT1;PAFAH1B1;PANK3;PAPSS1;PATZ1;PCBP1;PCBP2;PFN2;PIKFYVE;PLK1;PNRC1;PPP1CB;PRDX1;PRELID3B;PRRC2C;PSMA2;PSMA5;PSMG2;PTP4A1;PTPN9;QKI;QSER1;RAB33B;RAB5C;RAD51C;RAP1B;RAP2C;RARS;RB1;RCN2;RHPN2;RIOK2;RLIM;RNF144B;RPL10;RPL10A;RPL14;RPL15;RPL19;RPL21;RPL26;RPL31;RPL36A;RPL39L;RPL5;RPL7;RPL7A;RPL7L1;RPLP1;RPS15;RPS15A;RPS27;RPS8;RRM2;S100A10;SAP30L;SAT1;SEPSECS;SEPT10;SERBP1;SET;SF3B3;SGTB;SLC17A8;SMAD7;SMARCA5;SMCHD1;SMG1;SMPDL3B;SNRNP27;SNX4;SOGA1;SRD5A3;SREK1IP1;TAPBP;TCP1;TERF2IP;TEX264;THAP2;TM9SF3;TMED4;TMEM30A;TRIB1;TSC22D1;UBA2;UBB;UBE2E3;UBN2;UBQLN1;UBXN2B;UNC50;USP1;UTP4;VCP;VPS45;WDR4;WTIP;XRCC5;XRCC6;YBX3;YTHDC1;YWHAE;ZBTB7A;ZNF215;ZNF511;ZNRF2
hsa-miR-126-3p-477887_mir	-5.487	0.173	ACVR2B;ADAM9;ADGRE5;ADM;AKT1;AKT2;BCL2;CADM1;CCNE2;CDKN1B;CRK;CRKL;CXCL12;CXCR4;DNMT1;E2F1;EGFL7;EVI5;EZH2;FOXO3;GRIN2B;HOXA9;IGFBP2;IRS1;KCNJ1;KRAS;L2HGDH;LRP6;MERTK;MMP7;NFKBIA;PGR;PIK3CG;PIK3R1;PIK3R2;PITPNC1;PLAGL2;PLK2;PTPN7;RBMX;RGS3;RHOU;ROCK1;SIRT1;SLC39A6;SLC41A2;SLC45A3;SLC7A5;SOX2;SPRED1;SZRD1;TCF4;TEK;TOM1;TWF1;TWF2;VCAM1;VEGFA
hsa-miR-487a-3p-477826_mir	-6.772	0.174	ABCG2;ACTR2;ACTR3B;ACTR3C;AKIP1;ATP11C;BAZ2B;BRD3;CAND1;CHAF1B;CLEC12A;CLTC;CPEB4;EGR1;FAM217B;FIGN;FOCAD;GID4;HPS3;INTS3;ITGB8;KANSL1;KCNK5;KPNA3;LIN52;MAGI2;MAP1B;MAPK1;MCMBP;MMP16;MRI1;MYCBP2;NGDN;PANK3;PIK3R1;PLEKHA6;PLXNA3;PMEPA1;PRKAA1;PTPRS;RAB3B;RORA;RPS4X;SAMD8;SMIM13;SPRED2;SYNGAP1;SYPL1;TADA2A;TDRD1;TRIM66;TULP4;ZDHHC2;ZMYM1
hsa-miR-130b-3p-477840_mir	-5.897	0.179	ABCD2;ABCG8;ACBD5;ACP6;ACSL4;ACVR1;ADAR;ADARB1;ADARB2;ADM2;AGO1;AGO3;AHR;AKAP11;AKIRIN2;ANKFY1;ANKRD29;ANKRD50;ANKRD52;ANKRD9;ARCN1;ARHGAP1;ARHGAP12;ARHGEF26;ARID4B;ARL1;ARL17B;ARL6IP1;ARRDC3;ARSA;ASB16;ASB6;ASF1A;ATE1;ATF7IP;ATMIN;ATP6V0D1;ATP6V0E1;ATP6V1B2;ATP6V1C1;BAHD1;BLCAP;BMP3;BMT2;BNIP2;BRWD1;BTBD10;BTBD3;BTF3L4;BTG1;BTG3;C11ORF57;C12ORF65;C16ORF45;C16ORF70;C3ORF18;C3ORF38;C4ORF36;CALM2;CAMSAP2;CAPRIN2;CAPZB;CASP2;CBFB;CBY1;CCDC137;CCDC6;CCNA2;CCND2;CCR6;CCT6A;CD164;CD2AP;CDADC1;CDCA4;CDK19;CDK2AP2;CDK4;CEP170;CEP55;CERCAM;CFL2;CHEK2;CHERP;CHIC1;CINP;CKAP5;CLCN3;CLEC12B;CLIC4;CLIP1;CMPK1;CNOT1;CNOT4;CNOT6;CNTN1;COX10;COX20;CPPED1;CRY2;CSF1;CSNK1G1;CSNK2A1;CUL3;CXORF21;CYB5D1;CYLD;CYP20A1;CYP2C9;DAD1;DAPK1;DCAF8;DCBLD2;DCUN1D3;DDX5;DDX6;DEPDC1;DICER1;DLC1;DLG5;DLL1;DPYSL2;DSTYK;DTX4;DUSP18;DUSP8;DYNC1LI2;DYNLL2;E2F1;EDN1;EFCAB14;EGLN3;EGR2;ELL2;ENPP4;ENPP5;EOGT;EPC1;EPHA4;ERBB2;ERBIN;EREG;ESR1;EZH1;F2RL1;FAM114A1;FAM120AOS;FAM129A;FAM210A;FAM217B;FAT3;FBXL5;FBXO28;FGFR1OP;FIG4;FKTN;FLYWCH2;FMR1;FOXJ3;FOXQ1;FUT11;FXR1;FYCO1

			;FZD6;G6PC;GALNT4;GAS1;GFOD1;GLE1;GMFB;GNB5;GNPTAB;GOPC;GP2;GPATCH2;GPATCH8;GPR137C;GPR161;GPR75;GPR82;GPRC5A;GRAMD1A;GRB10;GRSF1;HABP4;HADHB;HARS;HBP1;HCCS;HEG1;HHEX;HIF1AN;HIVEP2;HNRNPUL1;HOXA5;HOXB3;HOXC8;HOXD11;HPRT1;HSP90B1;HSPA8;ICMT;IER3IP1;IFITM1;IFNLR1;IGF1;IGF2R;IGFBP5;IKZF4;IL23R;IPMK;IRF1;ITGB1;JARID2;JMY;KANSL1;KATNAL1;KBTBD2;KBTBD6;KCNB1;KCTD10;KCTD20;KDELR1;KIAA1191;KIAA1549;KIF13A;KLF11;KLF6;KLF9;KLHL21;KLHL28;KLHL36;KLRD1;KMT2D;KMT5B;KREMEN1;LBR;LCLAT1;LDLR;LEFTY1;LILRA2;LIMA1;LIPA;LMLN;LPGAT1;LRP8;LZIC;MAN1A2;MAN1C1;MAP3K9;MAP7;MAPK1;MAPKAPK5;MAPRE3;MASTL;MAVS;MB21D2;MBNL1;MBNL3;MCC;MED18;MED8;MFF;MID1IP1;MIDN;MIGA2;MKRN2;MLEC;MMGT1;MMP2;MOCS2;MPHOSPH9;MPP5;MREG;MRPL52;MSANTD4;MSMO1;MST1;MTMR4;MTMR9;MTPN;MYH11;MYLIP;NAA30;NAA50;NABP1;NACC2;NARS;NCAPD2;NCKAP5;NDEL1;NF2;NFE2L1;NFIB;NIN;NIP7;NIPA1;NKAP;NKD2;NOM1;NPTX1;NR2C2AP;NR3C1;NRBF2;NUS1;ODF4;OMD;ORC1;OSBP;OTUD3;OTUD4;PAFAH1B2;PAPD4;PAPOLA;PARP1;PBXIP1;PDE3B;PDGFRA;PDP2;PDRG1;PDZD11;PDZD8;PEX13;PGRMC1;PHF12;PHF3;PIGA;PIGG;PLA2G12A;PLEKHF2;PLEKHS1;PNRC1;POC1B-GALNT4;POGK;POGZ;POLR1B;POLR2D;POP7;PPARA;PPARG;PPARGC1A;PPIG;PPP1R14C;PPP1R15B;PPP1R9A;PPP6R1;PPP6R2;PPP6R3;PRKAA1;PRNP;PRPF38A;PRPF4;PRR23A;PRRG4;PRUNE2;PSAP;PSMB5;PSMD9;PTEN;PTER;PTGR2;PTP4A1;PTPN4;PTPRG;PURG;PXK;PXYLP1;QKI;QSOX1;RAB11FIP1;RAB12;RAB14;RAB1B;RAB34;RAB5A;RAB5B;RACGAP1;RAG1;RAN;RAP2C;RASSF1;RASSF8;RB1;RBM20;RBM23;RBM27;RBM43;RDH11;RFC2;RFT1;RFX7;RFXAP;RGMB;RLIM;RNF11;RNF125;RNF145;RNF149;RNF41;ROMO1;RPA2;RPF2;RPRD1B;RPRD2;RPS15A;RPS27A;RPS6KA5;RRAGD;RSRP1;RUNDC1;RUNX3;S1PR2;SALL3;SAMD8;SATB2;SAV1;SCAMP2;SCD;SEC16A;SEC23B;SECISBP2L;SEL1L3;SERINC3;SESTD1;SF3A1;SFTPA1;SFXN5;SGMS1;SGTB;SH3BP5;SH3PXD2A;SIGLEC9;SIK1;SIX4;SLAIN1;SLC10A3;SLC12A7;SLC31A1;SLC35D1;SLC35E2B;SLC35E3;SLC38A2;SLC38A9;SLC44A1;SLC46A1;SLC5A3;SLMAP;SMAD4;SMCR8;SMOC1;SMTNL2;SMYD2;SNAI3;SNAPIN;SNTB1;SNTB2;SNX12;SNX5;SOCS4;SON;SOX4;SPART;SPATA2;SPOPL;SRSF2;SRSF7;SSTR2;STARD13;STAT3;STK11;STRBP;STX16;STX6;STYX;SUN2;SYBU;TAOK1;TAX1BP1;TCF4;TCF7L2;TERF2;TES;TGFBR2;THAP6;THOP1;THRA;TIMM50;TMCO1;TMEM109;TMEM127;TMEM168;TMEM2;TMEM30A;TMEM9B;TMOD3;TMTC1;TNFRSF10B;TNFRSF21;TNKS1BP1;TNRC6A;TNRC6B;TOLLIP;TOM1L2;TP53INP1;TPP1;TRIM2;TRIM37;TRIM4;TRIM59;TRIM71;TRIP10;TROVE2;TRPC3;TSG101;TSPAN3;TXNIP;UBB;UBBP4;UBC;UBE2D2;UBE2D3;UBN2;UBXN2A;UCP1;UQCRB;UQCRQ;USP13;USP32;UVRAG;VCPKMT;VLDLR;VPS37A;VPS37B;WASL;WDR31;WDR33;WEE1;WIPF2;WNK3;XIAP;YY1;ZBTB18;ZBTB4;ZBTB7A;ZBTB7B;ZBTB8A;ZCCHC14;ZDHHC14;ZEB1;ZFYVE26;ZFYVE9;ZIC5;ZMAT3;ZNF107;ZNF12;ZNF154;ZNF202;ZNF217;ZNF224;ZNF24;ZNF264;ZNF317;ZNF354B;ZNF417;ZNF431;ZNF529;ZNF567;ZNF620;ZNF678;ZNF711;ZNF800
hsa-miR-30b-5p-478007_mir	-5.063	0.183	ACER3;ACTC1;ACTR10;ADAM9;ADO;ADPRHL1;AFF4;AKIRIN1;ALG9;ANKRA2;AP2A1;APLN;APLP2;ARF1;ARID3A;ARPP19;ASB3;ATG12;ATM;ATP2A2;ATP5C1;AVL9;AZIN1;B3GNT5;B4GALT1;B4GALT5;BAG4;BAHD1;BAZ1B;BCL2;BCL6;BCL9;BDP1;BECN1;BLOC1S6;BMT2;BTBD1;BTBD7;C1QBP;C7ORF43;C8ORF76;CAMKV;CAND1;CASP3;CAT;CBX2;CBX3;CCDC71L;CCNE2;CCNF;CD2AP;CDC37L1;CDC7;CDCA7;CELF1;CELSR3;CEP152;CEP350;CFDP1;CHAT;CHD1;CHST15;CLCC1;CLDN12;COPS7B;CPEB4;CREG1;CREM;CSF1;CSNK1G1;CTHRC1;CXCL11;CYB5B;DBF4;DCAF12;DCTN4;DCUN1D1;DDAH1;DHX40;DLL4;DNMT1;DPY19L3;DYNLT3;EDC3;EED;EIF2B1;EIF2S1;EIF5A2;ELOVL4;ELOVL5;EML4;ENPP4;ENTPD1;EPB41;EPG5;ERG;ERLIN1;EYA3;FAM104A;FAM81B;FAM8A1;FAM91A1;FANCF;FANCL;FBXO3;FBXO45;FEM1B;FERMT2;FOXA1;FOXG1;FOXN2;FRS2;FRZB;FUCA1;FYCO1;FYTTD1;G3BP1;GAB1;GALNT1;GCLC;GCSAM;GFPT2;GIGYF1;GLCE;GNA13;GNAI2;GNAZ;GNPDA1;GOLGA1;GOLGA8B;GPR75-ASB3;GTF2E2;GXYLT1;H6PD;HABP4;HEY1;HHIPL1;HIC2;HMGN2;HOXA1;IER5;IFNAR2;IFNE;IKZF2;IKZF4;IL1A;IL21R;IMPDH2;INSL6;IP6K3;IQCB1;IREB2;JADE2;JADE3;JAK1;JDP2;JOSD1;KBTBD6;KCTD5;KDM3A;KIF11;KIF5B;KLF10;KLF4;KLHDC10;KLHL15;KLHL28;KPNA6;KREMEN1;LARP1;LCLAT1;LCOR;LCP1;LDLR;LHFPL2;LIFR;LIN28B;LIN7C;LMBR1L;LPCAT1;LRP8;LRRC3C;LRRC8B;LRRC8D;LYPLAL1;MAPK8;MARCH4;MARCH6;MARCKSL1;MAST3;MATR3;MBNL1;MBNL2;MBNL3;MCFD2;MED29;MFAP3;MIA3;MIB1;MIER3;MKRN3;MLXIP;MOGAT1;MRO;MSANTD4;MTDH;MTF2;MTR;MTRNR2L10;MYBL2;MYLIP;MYO1E;MZT1;N4BP2;NAA25;NACC2;NAP1L1;NAPG;NCOA3;NCOA6;NDEL1;NDUFA12;NFAT5;NIPBL;NOTCH1;NRBP1;OLFML2B;OPHN1;OTUD4;PAPD5;PARP1;PAWR;PBRM1;PCDH10;PCGF5;PCMTD2;PCNT;PDCD10;PDGFRB;PEG10;PER2;PGGT1B;PGM3;PGPEP1;PHF13;PHTF2;PICALM;PIK3C2B;PIP4K2A;PLAGL2;PLEKHO2;PLIN3;PLSCR1;PLXNA1;PNMA1;PNN;POLR3E;POLRMT;POU4F1;PPARGC1B;PPP1R12A;PPP1R12B;PPP1R15B;PPP1R2;PPP2R1B;PPP3CB;PPTC7;PRDM1;PREPL;PRKAR1A;PRMT7;PSMD7;PTAR1;PTP4A1;QRFPR;RAB10;RAB18;RAB22A;RAD23B;RAP1B;RASAL2;RASGRP3;RBBP7;RBM14;RBSN;RETREG3;REV3L;RFX7;RGMB;RNF122;RNF135;RNF138;RNF220;RNF34;ROCK2;RPA

			2;RPS27A;RPS4X;RRAGD;RRM2;RUBCNL;RUNX2;S100PBP;SACS;SAE1;SBF1;SCML2;SEC24A;SEC61A2;SEMA6A;SERBP1;SERPINC1;SERPINE1;SETD3;SETD5;SFXN1;SH2B3;SH3GL1;SH3PXD2A;SHC1;SHROOM3;SIKE1;SIX1;SIX4;SKIDA1;SKIL;SLC35C1;SLC35G2;SLC38A7;SLC4A7;SLC7A5;SLFN5;SMAD1;SNAI1;SNRNP200;SNTB2;SOBP;SOCS1;SOCS3;SOX12;SOX4;SP4;SRPRA;SRSF7;ST3GAL5;STAU1;STRIP1;STRN;STX12;STX16;STX17;SYPL1;TAF4B;TANK;TAOK1;TBC1D10B;TBPL1;TFDP1;TGFA;TMED2;TMED5;TMEM106B;TNFRSF10B;TNRC6A;TNRC6C;TOMM5;TP53;TPRG1L;TRERF1;TRIM23;TRIM59;TSHZ3;TSPYL1;TTLL12;TWF1;TXNDC5;UBE2D3;UBE3C;UBN1;UBXN4;UHRF1BP1;USP37;USP53;VAPA;VASH1;VCPKMT;VPS33A;VPS41;WDFY2;WDR37;WDR43;WDR82;WDR89;XPO1;YOD1;ZBTB38;ZBTB39;ZCRB1;ZDHHC17;ZDHHC20;ZFAND5;ZFP36L1;ZMPSTE24;ZMYND8;ZNF200;ZNF264;ZNF460;ZNF507;ZNF543;ZNF567;ZNF589;ZNF646;ZNF770;ZNRF1;ZSCAN29;ZXDB
hsa-miR-146a-5p-478399_mir	-4.654	0.184	ACTBL2;AKAP8;ALG10B;ARL8A;ATG9A;ATP13A3;AVL9;BCL2A1;BCL7B;BCLAF1;BGLAP;BRCA1;BRCA2;BRWD1;BTN2A2;C16ORF52;C1ORF21;CARD10;CASP7;CCDC6;CCDC83;CCL5;CCNA2;CCND1;CCND2;CCR9;CD300LB;CD40LG;CD80;CDC73;CDKN1A;CDKN3;CFH;CNOT6L;COPA;COPS8;CPM;CXCL12;CXCL8;CXCR4;CYBRD1;CYTIP;DECR1;DGCR6L;DUSP1;EDEM3;EGFR;ELAVL1;ELP2;ENTPD5;EPSTI1;ERBB4;ESD;FADD;FAF1;FANCF;FANCM;FAS;FOS;GIMAP4;GPM6B;GPRIN2;GRAP2;GXYLT2;HAAO;HORMAD2;HOXD10;HSPA1A;ICAM1;IFI27;IFI44;IFI44L;IFIT1;IFIT3;IFIT5;IFITM1;IFITM3;IL1RAP;IL1RL2;IL6;IRAK1;IRAK2;IRF7;ISG15;ITGB2;ITGBL1;KCTD15;KDM2B;KDM6B;KIF22;L1CAM;LAMC2;LBR;LFNG;LIMD2;LIN52;LINC00304;LRP2;LSM4;MAN1C1;MBD4;MDN1;METTL7A;MFSD6;MICAL2;MIF;MKRN2;MPP2;MRPL10;MRPS30;MSC;MT-CO2;MTA2;MTUS2;MX2;MYLK;MYO6;NACC1;NANOS1;NFAT5;NFKB1;NMI;NOTCH1;NOTCH2;NSL1;NUMB;OASL;OLFML2A;OSBPL1A;PA2G4;PACS2;PARD6B;PLAUR;PLEKHG5;PMAIP1;POU3F1;PPP1R11;PPP1R1C;PPWD1;PRKCE;PTGES2;PTGS2;RAB2B;RAC1;RARB;RGS13;RGS9BP;RHO;RHOA;RHOBTB3;RNF11;ROBO1;ROCK1;RSAD2;RUFY2;S100A12;SAMD9L;SERBP1;SERPINA4;SERTAD2;SFRP1;SHCBP1;SIKE1;SLC10A3;SLPI;SMAD2;SMAD4;SMN1;SNTG2;SOS1;SOX2;SPATS2L;SPP1;SQSTM1;SRPRB;ST6GAL2;STAT1;STON2;SYT12;TGFB1;TLL1;TLR2;TLR4;TMEM101;TMEM136;TMEM167A;TMEM214;TMPRSS5;TRAF6;TRIM22;UHRF1;UMPS;USP48;UTP15;WASF2;WSB2;ZNF117;ZNF260;ZNF292;ZNF629;ZNF738
hsa-miR-142-3p-477910_mir	-4.668	0.189	ABCC9;ABCG2;ACACA;ACBD3;ACBD5;ACSL1;ACSL4;ADPRHL1;AFF1;AFF4;AGBL2;AGTR2;AK4;AKIRIN2;AKR1B10;AKT1S1;ALAD;APC;ARF4;ARHGAP12;ARL10;ARL14EP;ARL15;ARL5B;ARL6IP6;ARNTL;ATF5;ATG16L1;ATG9A;ATP2A2;ATP2B1;ATXN3;AZF1;BACH1;BAK1;BCLAF1;BMP8A;BOD1;BRMS1L;BTBD7;BTG2;C11ORF16;C11ORF74;C16ORF70;C18ORF25;C2;C4BPB;C5ORF24;C7ORF31;C9ORF72;CA5B;CALCOCO2;CALM1;CBY1;CCDC6;CCNG1;CCNJ;CCNT2;CDC14B;CDC25C;CDC6;CDCA4;CDKN1B;CEP192;CHRNA6;CLDN12;CLEC4D;CLIC4;CLOCK;CLTA;CLUAP1;CNIH4;CNN3;COPA;COPG1;CPEB2;CPS1;CREB3L2;CRH;CSNK1G3;CSRP2;CTNNB1;CUL5;CYR61;DAG1;DCAF12;DCTN4;DDX17;DDX39B;DEPDC1;DIP2A;DNAJB4;DNAJB6;DOCK10;DOCK6;DPY19L4;DTD1;DYRK3;ECT2;EDEM3;EEF2K;EGR2;EIF5;EIF5AL1;ELL2;EML4;ENAH;ENTPD1;EPM2AIP1;ESR1;ETNK2;EVI2B;FAM177A1;FAM208B;FAM209A;FAM222B;FAM98A;FBXO3;FICD;FKBP14;FLVCR1;FNDC3A;FREM1;FRS2;FYCO1;FZD7;GFI1;GINS2;GLCE;GNAQ;GNAS;GNB2;GOLGA1;GOLGA5;GOLT1B;GPC4;GPD1L;H3F3C;HECTD1;HGS;HIST1H4D;HMGA1;HMGA2;HMGB1;HOXA10;HOXA13;HOXA7;HOXA9;HSBP1;HSD17B12;HSD17B4;HSFY2;HSPA1B;IFNAR2;IL1A;IL6;IL6ST;INCENP;INPP5A;INPP5F;INSIG2;INTS7;IP6K1;IPMK;IRAK1;IRF1;IRF2BP1;ITGAV;KAT2B;KAT7;KBTBD2;KCND3;KCTD12;KIAA0232;KIAA1191;KIF5A;KIF5B;KLHL15;KMT2A;KRTAP4-5;LAMC1;LCOR;LGR5;LINC00598;LOX;LPP;LRRC32;LRRC59;LYRM4;MALT1;MAN1A2;MANBAL;MARCKS;MBD6;MCFD2;MEF2D;MESP2;MFSD8;MGLL;MKLN1;MMD;MMGT1;MON1B;MORF4L1;MORF4L2;MPLKIP;MRFAP1;MRGPRX1;MROH7;MSANTD4;MTCH1;MTFR1;MTMR9;MTUS1;NAP1L2;NBPF3;NCK2;NCKAP1;NEFH;NFATC2IP;NR2C1;NR2F6;NSD2;NSFL1C;NUDT8;OSBP;OTUD4;PANK3;PAPD7;PARP2;PCDHB14;PCDHGA10;PCDHGA2;PCMTD2;PDSS2;PEX26;PHF12;PIK3CG;PITPNC1;PNO1;POLI;PPFIA1;PPIL4;PPP1R14C;PPP1R15B;PPP1R2;PPP2R2C;PRKCA;PROM1;PRPF38B;PSMB5;PSMD11;PSMD12;PSME4;PSTK;PTPN23;PTPRJ;PUM1;RAB2A;RAB30;RAB39A;RAC1;RAD51;RBM27;RGL2;RGS5;RICTOR;RIPOR1;RNF170;RNF20;RNF38;ROCK2;RPE;RPS19;RRM2B;RTL8A;RTL8C;S100A11;SCAMP1;SCD;SCYL3;SDCCAG3;SEC23A;SECISBP2;SERPINA4;SERPINH1;SGMS1;SH2D1A;SIGLEC10;SIK1;SIK2;SKP2;SLC17A5;SLC25A13;SLC30A6;SLC35E1;SLC35F5;SLC35F6;SLC37A3;SLC39A9;SLC7A11;SLC7A9;SMG1;SNTN;SOCS5;SOCS6;SPPL2A;SPPL3;SRRD;SS18;STAM;STRBP;STRN3;STX2;STX6;SUCO;SYPL1;SYT4;TAB2;TAOK1;TAS2R7;TBC1D13;TBRG1;TBX4;TET3;TFG;TGFBR1;THBS4;TIFA;TIPARP;TMED4;TMED7;TMEM101;TMEM136;TMEM209;TMEM245;TMEM59;TMTC3;TNIP3;TNKS;TNPO1;TNRC18;TOR1AIP2;TPSG1;TRIM36;TRIM37;TROVE2;TSEN34;TSPAN6;TSPYL1;TUBAL3;TWF1;TWSG1;U2SURP;UBE2W;UCK2;UGT2B17;USP37;USP6NL;VAPB;VPS52;VSIG1;WASL;WHAMM;WIZ;XIAP;XRCC1;YES1;YOD1;YWHAE;YWHAH;ZCCHC14;ZFP36L2;ZFPM1;ZFX;ZMY

			ND8;ZNF217;ZNF236;ZNF24;ZNF264;ZNF460;ZNF473;ZNF529;ZNF608;ZNF676;ZNF678;ZNF682;ZNF701
hsa-miR-155-5p-477927_mir	-1.922	0.198	AAK1;ABCC4;ABHD16A;ABI2;ACOT7;ACOX1;ACTR2;ADAM10;ADAMTS4;ADD3;ADH5;AGL;AGO4;AGRN;AGTR1;AGTRAP;AIFM1;AIMP1;AKAP10;AKR1C3;AKR7A2;AKT1;ALDH1A2;ALDH3A2;ALDH5A1;ALDH9A1;AMIGO2;ANAPC16;ANKFY1;ANKRD12;ANPEP;ANTXR1;ANXA2;AP1G1;APAF1;APC;ARFIP1;ARFIP2;ARGLU1;ARID2;ARL10;ARL15;ARL5A;ARL5B;ARL6IP5;ARL8B;ARMC2;ARPC3;ASB6;ASNS;ASPH;ATG3;ATL2;ATP13A1;ATP6V1C1;ATP6V1H;ATPAF1;ATXN10;AURKA;AURKB;AXL;B4GALT1;BACH1;BAG5;BCAT1;BCL6;BCL7C;BET1;BRI3BP;BRPF3;BTBD1;BUD31;C12ORF10;C16ORF62;C17ORF80;C3ORF18;C3ORF58;CAB39;CAB39L;CALU;CAMTA1;CARD11;CARHSP1;CARS;CARS2;CASP3;CAT;CBFB;CBL;CBR4;CCDC137;CCDC82;CCL2;CCND1;CCND2;CCNT2;CCR9;CCT2;CD109;CD36;CD3EAP;CD68;CD81;CDC37;CDC40;CDC42BPB;CDC73;CDH13;CDH2;CDH6;CDK2;CDK4;CDK5;CDK5RAP3;CDKN1B;CDKN2A;CDV3;CEBPB;CEP41;CEP55;CEP83;CFAP73;CFL2;CHAF1A;CHAF1B;CHD7;CHD8;CHD9;CHRAC1;CHRDL1;CHTOP;CHURC1;CIAO1;CIAPIN1;CISD2;CKAP5;CLDN1;CLIC4;CLINT1;CLTA;CLTC;CLUAP1;CMSS1;CNDP2;CNNM3;CNOT10;CNOT6;CNOT9;CNPPD1;COG2;COL4A2;COLGALT1;COPS3;CORO1B;CPD;CPEB4;CPT1A;CRAT;CREB3L2;CREBRF;CS;CSE1L;CSF1R;CSNK1A1;CSNK1A1L;CSNK1G2;CSRP2;CTLA4;CTNNA1;CTNNB1;CTNNBL1;CTSA;CUL4B;CUTA;CUX1;CXCL8;CYFIP1;CYP1A1;CYP2U1;CYP51A1;CYR61;DAG1;DBN1;DCAF10;DCAF7;DCUN1D2;DDB2;DDRGK1;DDX10;DDX17;DDX3Y;DEGS1;DEK;DENND1B;DET1;DHCR24;DHX40;DIAPH3;DMD;DMTF1;DNAAF5;DNAJB1;DNAJC19;DNAJC2;DNMT1;DOCK1;DOCK4;DOK2;DPP7;DPY19L1;DR1;DRAP1;DSG2;DYNC2H1;E2F2;ECI1;EDEM3;EDN1;EEF1A2;EEF1E1;EEF2;EGFR;EHD1;EIF2B2;EIF2B5;EIF3A;EIF3C;EIF3CL;EIF3E;EIF3F;EIF3G;EIF3J;EIF4A1;EIF4E2;EIF4G2;ELK4;ENTPD1;EOGT;EPB41L2;EPRS;ERBIN;ERCC4;ERGIC1;ERI1;ERMP1;ESRRA;ETNK2;ETS1;EXOC2;EXOC3;EXOC7;EXOSC2;EZH1;F5;FADD;FADS1;FAM120A;FAM135A;FAM177A1;FAM199X;FAM3C;FAM76A;FAM91A1;FAM96B;FAM98B;FAR1;FASTKD1;FBXW7;FCAMR;FDFT1;FEZ2;FGF2;FGF7;FGL2;FIP1L1;FITM2;FKBP3;FLI1;FLNA;FLNB;FLT1;FMNL2;FMNL3;FNDC3B;FOS;FOXE1;FOXK1;FOXO3;FSTL1;FUBP1;FUBP3;GABARAPL1;GALC;GANAB;GAPVD1;GAR1;GATM;GCFC2;GCLC;GCSAM;GEMIN5;GEN1;GHITM;GLB1;GLG1;GLIPR1;GLIPR2;GMPPA;GMPS;GNA13;GNAS;GNB4;GNL3L;GNPNAT1;GOLPH3;GOLT1B;GPAM;GPM6B;GPT2;GRPEL1;GSK3B;H2AFY;HAL;HAX1;HBP1;HDHD5;HERC4;HHIP;HIF1A;HIVEP2;HK2;HLA-DPA1;HMGCS1;HNRNPA3;HNRNPA3P1;HOMEZ;HSD17B12;HSD17B7;HSDL1;HSPA4L;HSPB11;HTRA1;ICAM1;IER3IP1;IFIT5;IFNGR1;IGF2R;IKBIP;IKBKE;IL13RA1;IL17RB;IL2;IL6;IMPAD1;INA;INPP5A;INPP5D;INPP5F;INSL6;INTS10;INTS4;INTS6;INTS7;INTS8;IPO8;IRF2BP2;ITGB4;ITGB5;ITK;JADE1;JARID2;JCHAIN;JUN;JUNB;JUP;KANK2;KANSL1;KBTBD2;KCNN3;KCTD3;KCTD5;KDELC1;KDELC2;KDM1A;KDM3A;KIAA0368;KIAA1841;KIF14;KIF22;KIF3A;KLF9;KLHL28;KLHL42;KLHL5;KMT5A;KPNA5;KRAS;KRCC1;KRT6B;KRT80;L2HGDH;LARS;LAT2;LCLAT1;LCORL;LDOC1;LEMD3;LIN7C;LNPK;LNX2;LONP2;LPGAT1;LPL;LRIF1;LRRC40;LRRC59;LSM3;LTN1;LUC7L2;LUC7L3;LUZP1;LY6K;MAFB;MAN1A2;MAP3K10;MAP3K14;MAPK13;MAPK14;MARC1;MARC2;MARCKS;MARF1;MARVELD1;MASTL;MAT2B;MATR3;MAVS;MBLAC2;MBNL3;MCAM;MCM8;MECP2;MECR;MEF2A;MEIS1;MEST;METAP2;METTL7A;MEX3C;MFF;MGST2;MIDN;MITF;MKLN1;MLH1;MMP16;MMS22L;MORC3;MOSPD2;MOV10;MPP2;MPP5;MPZL1;MRPL16;MRPL18;MRPS27;MRPS34;MRS2;MSH2;MSH6;MSI2;MTAP;MTFMT;MTHFD2;MTRNR2L1;MTRNR2L3;MTRNR2L5;MTRNR2L7;MTRNR2L9;MUS81;MUT;MXI1;MYB;MYBL1;MYC;MYD88;MYLK;MYO10;MYO1D;MYO1E;MYO6;N4BP1;NAA16;NAA25;NAA50;NAMPT;NARS;NASP;NCAPD2;NCAPG;NCKAP1;NEMP1;NES;NEU1;NEUROG1;NFAT5;NFATC2IP;NFKB1;NFYC;NKX3-1;NMD3;NOB1;NOLC1;NOS3;NOTCH2;NOVA1;NR1H3;NR3C1;NSA2;NSD3;NSUN5;NT5E;NUCKS1;NUDT19;NUP155;NUP62;NUPL2;OBSCN;OGFOD1;OLR1;OSBPL10;OSBPL9;OSTM1;OTULIN;OVCA2;OXCT1;OXNAD1;P3H3;PACSIN2;PACSIN3;PAK2;PALD1;PALLD;PAM16;PAPOLA;PATJ;PAXBP1;PAXX;PBRM1;PCCA;PCCB;PCDH9;PCNT;PCYOX1;PDAP1;PDCD10;PDCD4;PDE12;PDE3A;PDK1;PDLIM5;PDPR;PEA15;PEBP1;PELI1;PFDN4;PGRMC2;PHACTR2;PHC2;PHF14;PHF6;PHGDH;PHIP;PICALM;PIK3CA;PIK3R1;PKIA;PKN2;PLAUR;PLEKHA2;PLEKHA5;PLK1;PLS1;PLXND1;PNPLA4;PNPLA8;PNPT1;PODXL;POLE3;POLE4;POLR1B;POLR2C;PPFIBP1;PPL;PPM1G;PPP2R2A;PPP5C;PRAF2;PRKAR1A;PRKAR2A;PRKCI;PRRC1;PRSS21;PSAT1;PSEN1;PSIP1;PSME3;PSME4;PSMG1;PTAR1;PTEN;PTMS;PTN;PTPRJ;PUS7;PYGL;QPCTL;QRICH1;RAB11FIP2;RAB14;RAB23;RAB27B;RAB2A;RAB30;RAB34;RAB3B;RAB3IP;RAB5C;RAB6A;RAB6C;RAC1;RAD23B;RAD51;RAI14;RAP1B;RAPGEF2;RAPH1;RARS;RBAK;RBM22;RBM42;RBPJ;RCN2;RCOR1;RDH13;RETSAT;REV1;RGL1;RHEB;RHEBP1;RHOA;RICTOR;RING1;RIOK2;RNF123;RNF2;RORA;RPAP1;RPL39;RPRD1A;RPS20;RPTOR;RRAGA;RREB1;RRM2;RSF1;RTFDC1;RTN3;RUNX2;S100A11;S1PR1;SACM1L;SAMHD1;SAP30L;SARAF;SCAMP1;SCD;SDCBP;SEC24B;SECISBP2;SEL1L;SELE;SELENOT;SEPT11;SERGEF;SERTAD2;SGPL1;SH3BP4;SH3PXD2A;SHANK2;SIN3A;SIRT1;SKI;SKIV2L2;SLC11A2;SLC12A4;SLC1A5;SLC25A19;SLC25A40;SLC27A2;SLC30

			A1;SLC30A7;SLC33A1;SLC35A1;SLC35F2;SLC38A5;SLC39A10;SLC39A14;SLC7A1;SLC7A11;SLC9A3R2;SMAD1;SMAD2;SMAD3;SMAD4;SMAD5;SMARCA4;SMARCD2;SMARCE1;SNAP29;SNTB2;SNX6;SOCS1;SOCS3;SOCS6;SOX6;SP1;SPCS1;SPECC1;SPI1;SPIN1;SPRED1;SRPK2;SRSF1;SRSF2;SSH2;SSSCA1;SSU72;SSX2IP;STAG2;STAT1;STAT3;STIM1;STK24;STRBP;STRN;STRN3;STX5;STXBP2;SUPT5H;SUZ12;SWSAP1;SYNE2;SYPL1;TAB2;TACSTD2;TADA2B;TAF5L;TAPT1;TBC1D14;TBC1D8B;TBCA;TBRG1;TCEA1;TCF12;TCF4;TDRD6;TERF1;TFAM;TFCP2;TFPI;TGM2;THBS1;THOC7;THRB;TICAM2;TIMM13;TIMM8A;TJP1;TLE4;TM6SF1;TMBIM6;TMEM123;TMEM136;TMEM167A;TMEM33;TMOD3;TMTC3;TMX3;TNFAIP2;TNFRSF10A;TNKS1BP1;TNPO1;TOMM20;TOMM34;TP53INP1;TPBG;TPD52;TPP2;TPRKB;TRAK1;TRAM1;TRIM24;TRIM32;TRIO;TRIP13;TRMT1;TROVE2;TRPS1;TSHZ3;TSPAN14;TSPAN3;TTC37;TTC8;TTF1;TWF1;TWSG1;TXNDC12;TXNRD1;TYRP1;TYSND1;UAP1;UBA2;UBA3;UBE2D2;UBE2D3;UBE2G1;UBE2H;UBE2J1;UBE2J2;UBL3;UBQLN1;UBQLN2;UBR4;UBTD2;UBXN1;UBXN2B;UFL1;UGDH;UGT8;UPF2;UQCRB;UQCRFS1;USP8;VAMP3;VANGL1;VAV2;VBP1;VCAM1;VCPIP1;VEZF1;VHL;VPS18;VPS36;VPS4B;WBP1L;WDFY1;WDR82;WEE1;WNK1;WNT5A;WRB;WWC1;XPC;XPNPEP1;XPO1;XPR1;YARS;YBX3;YEATS2;YWHAZ;ZBTB18;ZBTB38;ZFP36;ZIC3;ZKSCAN1;ZKSCAN5;ZNF148;ZNF160;ZNF207;ZNF236;ZNF248;ZNF254;ZNF260;ZNF273;ZNF28;ZNF300;ZNF384;ZNF431;ZNF468;ZNF492;ZNF493;ZNF500;ZNF561;ZNF611;ZNF644;ZNF652;ZNF678;ZNF703;ZNF714;ZNF83;ZNF98;ZSWIM6
hsa-miR-190a-5p-478358_mir	-7.636	0.211	ADAMTS8;ADCYAP1R1;ATXN7L3B;BCL2L13;CALML4;CCDC160;CDKL2;CDKN1B;CSDE1;CYGB;DAB2;DUSP10;ENO4;FAM13B;FOXN2;FOXP2;GPC5;HACD4;HBS1L;IGF1;IL6ST;IL7R;INVS;KCNMB4;KCNQ5;KIAA0232;KLF6;KLHL24;LDHA;LRRC58;MAP3K2;MAP3K9;MARK2;MED4;MKL2;MSANTD4;MTRNR2L10;MTRNR2L8;NOTCH2NL;OMD;OVOL1;PCDHB11;PGAM4;PHLPP1;PIGM;PM20D2;PTPRJ;RAB30;SDC3;SERP1;SGMS2;SIGMAR1;SPRED1;STAMBP;TATDN2;TMEM161B;TNRC6A;TRA2B;TRIM5;ZNF223;ZNF264;ZNF460;ZNF608;ZNF850
hsa-let-7a-5p-478575_mir	-3.399	0.214	ABHD17C;ABT1;ACAD8;ACER2;ACOT9;ACTA1;ACTB;ACVR1B;ADH5;ADIPOR2;ADSL;AGO1;AGO2;AGO4;AHCYL2;AHR;AIDA;AK4;AKAP17A;AKAP8;ALDH7A1;AMD1;AMMECR1;AMPD2;ANGEL2;ANKRD17;ANKRD46;ANO6;AOC2;AP1S1;APP;AQP6;AREL1;ARG2;ARID3A;ARID3B;ARIH1;ARL6IP6;ARL8B;ARRDC1-AS1;ARRDC3;ASCC3;ASPM;ATG12;ATG4C;ATG9A;ATP2A2;ATP2B1;ATP6V1B2;ATP6V1F;ATP6V1G1;ATXN2;ATXN2L;ATXN7L3;ATXN7L3B;AURKB;B4GALNT3;BACH1;BAZ1B;BAZ2A;BCAT1;BCL2;BCL2L11;BCOR;BEND4;BIRC6;BMPR1B;BMT2;BNIP3L;BPTF;BRI3BP;BSDC1;BTBD1;BUD13;BZW1;C11ORF57;C12ORF4;C19ORF47;C19ORF53;C1GALT1;C1ORF21;C1ORF210;C1RL;C2CD4A;C5ORF51;CALU;CAMK2D;CAND2;CARHSP1;CASP3;CASP8;CASP9;CASTOR2;CBX5;CCNB2;CCND1;CCND2;CCNG1;CCNT2;CCR7;CD276;CD2AP;CD55;CD59;CDC25B;CDC34;CDC7;CDK6;CDKAL1;CDKN1A;CDV3;CELF1;CEP120;CEP135;CEP170B;CFL2;CHTOP;CLDN12;CLPB;CNBP;CNOT9;CNTRL;COIL;COL8A1;COLEC12;COPZ1;COX14;COX6B1;CPA4;CRX;CRY2;CRYBG3;CS;CSNK2A1;CTDP1;CTPS1;CXCL8;CYP2R1;DDX18;DEGS1;DHX57;DHX9;DIABLO;DICER1;DIS3L2;DISC1;DNA2;DNAH9;DNAJC28;DNAJC6;DNAL1;DPH5;DSC2;DTX3L;DUSP1;DVL3;DYNC2H1;DYRK3;E2F1;E2F2;E2F6;ECHDC1;EDEM3;EDN1;EEF2;EFHD2;EGFR;EGR3;EIF3C;EIF4A3;EIF4G2;EMILIN2;EPHA4;ERN1;ERO1A;ESPL1;EWSR1;EXPH5;EZH2;F2R;FAM104A;FAM105A;FAM13C;FAM171A2;FAM222B;FAM43A;FAM83G;FARSA;FBXL20;FBXW2;FIGN;FKBP8;FMNL3;FMO4;FNDC3A;FNDC9;FNIP1;FOXA1;FOXJ3;FPR1;FUT10;FXN;FZD9;G2E3;GABPAP;GADD45GIP1;GATM;GBF1;GCN1;GGA3;GGPS1;GLO1;GLUL;GNG5;GOLGA4;GORASP2;GP5;GPAT4;GPM6B;GPN1;GRPEL2;GTF2I;GTF3C1;GTF3C5;H1F0;HAND1;HAS2;HASPIN;HDAC5;HERPUD1;HIST1H1D;HIST1H2BD;HIST1H2BK;HIST1H4D;HIST2H2BE;HIST2H2BF;HMGA1;HMGA2;HMGN2;HNRNPDL;HNRNPUL1;HOXA6;HOXB4;HOXC4;HRAS;HSF2;HUWE1;IARS;ICOSLG;IFNLR1;IGDCC4;IGF1R;IGF2;IGF2BP1;IGF2BP3;IKZF3;IL6;IL6R;ILKAP;INTS12;INTS7;IPO7;IPO9;ITGA3;ITGB3;ITM2B;IVD;JAZF1;JPH4;KCTD1;KCTD21;KDM6B;KIAA0391;KIAA0930;KIAA1143;KIAA1147;KIAA1328;KIAA2026;KIF27;KLHDC8B;KMT2D;KPNA5;KPNA6;KRAS;KREMEN1;KRTAP19-1;LARP1;LEFTY1;LHFPL2;LILRB4;LIMD2;LIN28A;LIN28B;LIX1L;LMNA;LRIG3;LRRC20;LRRC37A2;LRRC42;LTA4H;LYN;MAGEA12;MAGEA3;MAGEA6;MAP2K7;MAP3K1;MAP4K4;MAPK11;MAPK6;MARCKSL1;MARS2;MBD2;MCF2L2;MCM3AP;MDM4;MED13L;MEF2D;MEIS1;MEIS3P1;MEPCE;METTL6;MFSD8;MIB1;MIDN;MIEF1;MLLT10;MPL;MRPL12;MRPL15;MRPS2;MRS2;MSI2;MT-ATP6;MT-CO1;MT-CO2;MT-ND1;MT-ND2;MT-ND5;MTFR1L;MTHFD1;MTUS1;MTX3;MXD1;MYC;MYO9A;NAA20;NAA30;NAA35;NAP1L1;NAT8L;NCBP1;NCKIPSD;NCOA3;NDUFA4P1;NDUFAF3;NDUFAF7;NDUFS2;NEFM;NEU3;NF2;NFATC2IP;NFKB1;NHLRC2;NHLRC3;NID2;NIPBL;NKIRAS2;NME4;NOA1;NOLC1;NOM1;NPC1;NPTX1;NR1I2;NR6A1;NRAS;NSD1;NUCB2;NUDT4;NUDT5;NUP155;NUP58;ONECUT2;OPA3;OPRL1;ORAI2;OTULIN;PAFAH2;PAK1;PALD1;PAQR5;PARP1;PARP16;PAXBP1;PBX2;PCGF3;PCIF1;PDE12;PDE4DIP;PDGFB;PDLIM5;PDP2;PDZD8;PEG10;PEX11B;PEX14;PGM2L1;PGRMC1;PHACTR4;PHF6;PHTF2;PIGN;PIK3R1;PKM;PLAGL2;PLCG2;PLD3;PLEKHA3;PLEKHO1;PLOD1;PLXND1;PM20D2;PMAIP1;PMPCA;PNRC1;POLL;POLR2D;POLR3D;POTEG;POTEM;POU3F2;PPP

			1R15B;PPP2R2A;PRDM1;PREB;PRIM2;PRR5-ARHGAP8;PRSS22;PSMA2;PTGES2;PTP4A1;QDPR;QKI;RAB11FIP4;RAB13;RAB19;RAB40C;RABL2A;RABL2B;RAD18;RAD21;RANBP2;RAVER1;RAVER2;RBFOX2;RBM12B;RBM6;RBMXL1;RDX;REPS1;RFC2;RHBDF2;RHD;RIOK3;RNF144B;RNF44;RNFT1;RPL30;RPL35A;RPL4;RPL9;RPS10;RPS13;RPS15A;RPS24;RPS3A;RPS4X;RPSA;RRAD;RRM1;RRM2;RWDD1;S100PBP;SALL3;SAMD4B;SAR1A;SDR42E1;SEL1L3;SEMA4C;SESN1;SESN2;SETD7;SF3A1;SFSWAP;SHMT2;SIK1;SKA3;SLC10A7;SLC11A2;SLC12A7;SLC16A9;SLC19A3;SLC20A1;SLC35A4;SLC35C1;SLC38A7;SLC5A6;SMARCAD1;SMC1A;SMCR8;SMG7;SMPDL3B;SNRPC;SNX17;SNX2;SNX6;SOCS1;SOCS4;SOD2;SP1;SPATS2;SPTBN1;SRSF2;STAT2;STAT3;STK4;STRN;STX3;SUMO1;SUOX;SURF4;SYNJ2BP;SYNPR;SYT1;SYVN1;TAB2;TAF5;TAF5L;TAF9B;TARS;TBC1D19;TCERG1;TGFBR3;TGOLN2;THBS1;THEM6;THYN1;TIAF1;TMED4;TMED5;TMED7;TMEM165;TMEM241;TMEM39A;TMEM64;TMTC3;TMX2;TNFAIP3;TNFRSF10B;TNFSF9;TOMM40L;TRAF7;TRAPPC10;TRIM5;TRIM71;TRIO;TRMO;TRRAP;TSC22D2;TSPAN3;TTC22;TTC26;TUBB2A;TUBB4A;TUSC2;TXLNA;TXLNG;UBA6;UBAP2;UBAP2L;UBE2Q1;UBE3C;UBOX5;UBXN2B;UHRF1;UHRF2;UNK;UPP2;USP14;USP35;USP38;USP47;UTP4;VAV2;VCL;VDR;VGLL4;WASL;WDR46;WDR6;WIPI2;WNT1;WWP2;YAE1D1;YOD1;YWHAZ;ZBTB37;ZBTB5;ZBTB8OS;ZC3H18;ZC3HAV1L;ZCCHC3;ZCCHC9;ZFAND4;ZFP36L1;ZMYM3;ZNF200;ZNF260;ZNF264;ZNF28;ZNF417;ZNF443;ZNF460;ZNF555;ZNF556;ZNF566;ZNF578;ZNF584;ZNF587;ZNF609;ZNF611;ZNF629;ZNF644;ZNF738;ZNF746;ZNF774;ZNF780A;ZNF799;ZNF8
hsa-miR-27a-3p-478384_mir	-3.430	0.217	ABCA1;ABHD17C;ABL2;ACER2;ADAMTS5;ADAR;ADD1;ADGRL1;ADORA2B;AFF4;AGPAT3;AKIRIN1;ALDH9A1;ALDOA;ALG9;AMOTL2;ANKRD40;AP1G1;AP3B2;AP3D1;APBB2;APC;APEX1;APPBP2;ARL4C;ARL5B;ARL6IP1;ARNTL;ATN1;ATP5G3;ATP7B;ATPAF1;BAG2;BDNF-AS;BEX3;BMI1;BMPR2;BNIP3;BRAT1;BTG2;BUB3;C2CD2L;C5ORF51;C6ORF120;C8ORF4;CAB39;CAB39L;CAPN15;CARM1;CCND1;CCNK;CCNL2;CCNT2;CCNYL1;CD2AP;CDC27;CDH6;CELF1;CELF2;CEP162;CEP55;CFDP1;CHEK2;CNN3;COASY;COPZ1;CPEB4;CPPED1;CRISP2;CSRP2;CTNND1;CUL1;CYP1B1;DAZAP2;DCAF7;DCUN1D4;DDI2;DERL1;DKK2;DNAJC27;DPY19L3;DPYD;DUSP5;DYNC2LI1;DYNLL2;DYRK3;E2F7;ECT2;EFHD2;EGFR;EIF2S2;EIF5;EIF5A2;ELL2;ELMO1;EN2;ENDOU;ERC1;EZH2;FAM136A;FAM217B;FAM49B;FAM69A;FAM84B;FARSA;FBXO46;FBXW7;FCHSD2;FEM1B;FHL2;FIZ1;FNDC3A;FOXJ3;FOXN2;FOXO1;FOXP4;FRYL;FSTL1;FZD9;GATA2;GATA3;GCSAM;GIPC1;GLCCI1;GNB5;GNG12;GNPNAT1;GPAM;GPATCH11;GPT2;GRB2;GRWD1;GSE1;GSK3B;GTF2IRD2;GYS1;H3F3B;H3F3C;HAT1;HIF1A;HINT1;HIPK2;HIST2H2AA3;HIST2H2AA4;HLA-DRA;HMGCR;HMGN1;HMGXB4;HNRNPF;HOXA10;HOXB3;HOXC11;HOXC6;HOXD11;HRK;ICK;ID4;IER3;IFNG;IFNR;IGF1;IMPA1;INO80D;ITCH;ITSN2;JMY;KCTD12;KCTD14;KHSRP;KIAA1551;KLHDC3;KMT2A;KMT2C;KMT2D;KMT5B;KRAS;KRTAP13-2;LAMP2;LBR;LDLR;LENG1;LFNG;LIFR;LIMA1;LIN54;LITAF;LMNB2;LONRF1;LPCAT1;LPIN1;LRRC61;LYSMD3;MACC1;MANEA;MAP1B;MAP2K4;MAPK14;MBTD1;MED13;MED14;MED30;MET;METTL8;MGEA5;MIER3;MIGA2;MKLN1;MKNK2;MMP13;MMS22L;MPRIP;MRC1;MRPS14;MRPS27;MYO1C;MYO1F;MYO9A;MYT1;NAA50;NCBP2;NDUFC2-KCTD14;NECAP1;NEURL1B;NF1;NFE2L2;NLK;NLN;NME2;NOLC1;NPEPPS;NR1D2;NR2F2;NRAS;NRBP1;NSD1;NUDT21;NUFIP2;NUP133;NUP93;NUS1;OLR1;OSBPL10;OTUD4;PAIP2;PAK2;PAX3;PDHX;PDIA5;PDK1;PDS5B;PEG10;PFAS;PGM2L1;PHB;PHC2;PHLPP2;PIGO;PIK3CG;PINK1;PISD;PITHD1;PJA2;PKNOX1;PLA2G2D;PLAG1;PLAGL2;PLXND1;PMAIP1;PNKD;PNRC2;PPARG;PPIC;PPIF;PREX1;PRKAA2;PROX2;PRR14L;PSAP;PSPC1;RAB14;RABGAP1;RAP1B;RARA;RBBP5;RETREG2;RGPD4;RGS6;RMND5A;RNF139;RNF152;RNF182;RNF38;RPL19;RPP25L;RPS18;RPS24;RPS6KA5;RPTOR;RREB1;RUNX1;RXRA;RYBP;SEC16A;SEC24A;SEMA6A;SEMA6D;SEMA7A;SERBP1;SERP1;SERTAD3;SETD1B;SETD7;SFRP1;SFXN1;SFXN4;SGMS1;SGPL1;SIGLEC1;SLC16A1;SLC25A25;SLC25A44;SLC26A2;SLC46A3;SLC6A17;SLC6A8;SLC7A11;SLC7A2;SMAD2;SMAD4;SMAD5;SNCG;SNRNP27;SNX25;SOCS6;SOS1;SP1;SP3;SP4;SPATA13;SPIB;SPICE1;SPRY2;SRSF1;STAU1;SUCO;SUN2;SZRD1;TAOK1;TARDBP;TBC1D9;TERF2IP;TFPI;TGFBR1;TGFBR3;TGOLN2;THOC3;THRB;TIAL1;TIGD2;TIMM10;TLNRD1;TMBIM6;TMED5;TMEM167A;TMEM170B;TMEM254;TMEM30A;TMEM68;TMEM91;TMSB10;TMTC3;TNPO1;TOMM40L;TOPBP1;TOR2A;TP53;TP53INP1;TP53INP2;TPT1;TRIM23;TRIM27;TROVE2;TRUB2;TSEN54;TSPYL1;TTC3;TXNDC5;TXNIP;UBE2D1;UBE2G1;UBR5;UBXN11;UGCG;USP25;USP46;VPS26B;VPS35;WASHC2C;WASL;WDCP;WDFY1;WDR77;WDTC1;WEE1;WNK1;WNT9B;YAP1;YIPF4;YWHAZ;ZBTB10;ZBTB18;ZBTB20;ZBTB37;ZFHX3;ZFP1;ZFP36;ZFP36L1;ZFP36L2;ZFX;ZNF106;ZNF148;ZNF175;ZNF384;ZNF460;ZNF618;ZNF627;ZNF638;ZNF778;ZNF800
hsa-miR-23a-3p-478532_mir	-0.493	0.217	ABCD1;ACSS3;ACTN4;ADAM17;ADAM28;ADAM9;ADD3;APAF1;ARL6IP1;ASNS;ATAT1;ATP5A1;ATXN7L3B;BAZ2A;BCAP29;BRWD1;BSDC1;BTLA;C2ORF69;C8ORF58;CAMKV;CCL8;CCT5;CD302;CDH1;CELF1;CENPM;CEP57L1;CHUK;CNN2;CNOT1;CNOT6;CNOT9;CRLS1;CSDE1;CXCL12;CXCL8;DCP2;DDX5;DNAJC21;DOCK4;DSTN;DYNC2LI1;DYNLL2;EGLN3;EN2;ERBIN;ETNK1;FAM222B;FAM91A1;FANCG;FAS;F

			ASTKD1;FASTKD5;FAU;FBN2;FKBP4;FLNA;FNIP1;FOXA1;FOXM1;FOXO3;FOXR2;FSD1L;FUT4;FZD5;G6PC;GGA3;GJA1;GLS;GMPS;GNAI3;GNB2;GRK2;GRTP1;GTF3C4;HAPLN1;HCFC1;HES1;HIP1R;HIST1H3B;HIVEP1;HMGB2;HMGCS1;HMGN2;HNF1B;HNRNPUL1;HOXB4;HRG;HS2ST1;HSP90AA1;IL6R;IMPDH2;IRF1;ITPKC;JMJD1C;KDM3B;KIAA1210;KIAA1551;KIF20A;KIF22;KLF10;KLF12;KLF3;LAMP1;LDHA;LDHB;LEMD2;LMAN2;LMNB1;LPAR1;LRP5;MAP1B;MC2R;MCFD2;MCU;MEF2C;MGAT5;MMGT1;MPHOSPH6;MRPS34;MT2A;MTAP;MYC;MYH1;MYH10;MYH2;MYH4;NACC1;NDUFV3;NEK6;NLGN4X;NLK;NOL11;NUFIP2;NUP153;P2RY11;PAFAH1B3;PDCD6IP;PDIA6;PEX26;PIGM;PIK3R1;PKM;PLAG1;PNRC2;POM121C;POU4F2;PPARGC1A;PPIC;PPP2R5E;PSAP;PSMC3;PTEN;PTGFRN;PTPDC1;PTPN11;PTPN14;QSER1;RGS5;RNF111;RNF38;RNMT;RPL7L1;RPP14;RPRD2;RPS4X;RPS5;RPSAP58;S100A7A;SCAMP2;SDHD;SEMA6D;SEPT2;SERINC3;SESN2;SLC27A4;SLC35G3;SLC6A15;SLC6A6;SMAD3;SMAD5;SNTG1;SOCS6;SOD2;SPRY2;SPTY2D1;SSR1;SSRP1;ST7L;STAMBPL1;STARD7;STAT3;STS;STT3B;SWAP70;TBL2;TCF25;TERF2;THUMPD3;TLR6;TMED7;TMEM170A;TMEM64;TNFAIP3;TNFAIP8;TNPO1;TNRC6A;TOP1;TOPBP1;TOR1AIP1;TPM3;TRIM59;TRIM63;TRRAP;TSC1;TSNAX;TXNIP;TXNL4A;UBALD1;UQCRFS1;USP34;USP5;VAV3;VCAM1;WEE1;XIAP;ZBTB10;ZCCHC2;ZIK1;ZNF117;ZNF138;ZNF208;ZNF253;ZNF257;ZNF267;ZNF268;ZNF273;ZNF275;ZNF281;ZNF319;ZNF426;ZNF485;ZNF550;ZNF578;ZNF669;ZNF682;ZNF701;ZNF704
hsa-let-7d-5p-478439_mir	-2.014	0.224	ABHD17C;ABT1;ACER2;ACOT9;ACTA1;ADCY9;ADH5;ADIPOR2;AGO1;AHCYL2;AHR;AK4;AKAP8;AMD1;ANKRD46;AP1S1;APP;AQP6;AREL1;ARID3A;ARID3B;ARIH1;ARL8B;ARRDC4;ATG12;ATG9A;ATP6V1F;ATP6V1G1;ATXN2;ATXN7L3;ATXN7L3B;BACH1;BEND4;BIN3;BRI3BP;BUB3;BZW1;C11ORF57;C12ORF4;C19ORF47;C19ORF53;C1ORF21;C1ORF210;C1RL;C5ORF51;CACNG8;CALCOCO2;CALU;CARNMT1;CASTOR2;CBR4;CBX5;CCND1;CCNT2;CD59;CDKAL1;CDKN1A;CDV3;CELF1;CEP120;CEP135;CHTOP;CIZ1;CLDN12;COIL;COL3A1;COL8A1;COLEC12;COX6B1;CPA4;CPNE1;CRX;CRY2;CRYGEP;CTDP1;CTPS1;CXCL8;DHX57;DIABLO;DICER1;DISC1;DLC1;DNA2;DNAH9;DNAJC28;DNAL1;DSC2;DTX3L;DUSP1;DVL3;DYRK3;E2F6;ECHDC1;EDEM3;EDN1;EFHD2;EIF4A3;EIF4G2;EMILIN2;EPHA4;ERC1;ERO1A;ESPL1;FAM104A;FAM105A;FAM135A;FAM222B;FAM43A;FAM83G;FBXL20;FBXO3;FBXW2;FIGN;FMNL3;FMO4;FNDC3A;FNDC9;FPR1;FUT10;FXN;FZD9;GABPAP;GATM;GGA3;GLMN;GLO1;GNG5;GOLGA4;GOLT1B;GPAT4;GPR137B;GPR63;GRPEL2;H2AFX;HAND1;HASPIN;HERPUD1;HIST1H2BD;HIST1H2BK;HMGA1;HMGA2;HOMER1;ICOSLG;IFNLR1;IGDCC4;IGF1R;IGF2BP1;IGF2BP3;IKZF3;IL13;IL6R;INTS7;IPO9;ISL1;ITGA3;KATNAL1;KCTD21;KIAA0391;KIAA0930;KIAA1143;KIAA1328;KIF27;KLHDC8B;KMT2A;KMT2D;KPNA5;KREMEN1;LARP4B;LBR;LEFTY1;LIMD2;LIN28B;LIN52;LRIG3;LRRC20;LRRC42;LYN;MAGEA12;MAGEA3;MAGEA6;MAP2K7;MAPK6;MARCKSL1;MARS2;MBD2;MCF2L2;MDM4;MEF2D;MEIS3P1;MFSD8;MIDN;MIEF1;MLLT10;MPL;MRPL12;MRPL51;MSI2;MTUS1;MTX3;MXD1;MYC;NAA20;NAA30;NAP1L1;NAT8L;NCKIPSD;NCOA1;NCOA3;NDUFA4P1;NEMP1;NHLRC2;NHLRC3;NOA1;NOM1;NR4A1;NR6A1;NSD1;NUCB2;NUP155;ONECUT2;OPA3;OPRL1;PAFAH2;PARP16;PAXBP1;PBX2;PCGF3;PCNP;PDE12;PDGFA;PDGFB;PDLIM5;PDP2;PDZD8;PEG10;PEX11B;PGM2L1;PGRMC1;PHACTR4;PLAGL2;PLCG2;PLD3;PLEKHA3;PLEKHO1;PLXND1;PM20D2;PMAIP1;PMPCA;POLL;POLR2D;POLR3D;POTEG;POTEM;PPP1R15B;PPP2R2A;PRIM2;PRR13;PRR5-ARHGAP8;PRSS22;QDPR;RAB11FIP4;RAB19;RAB40C;RABL2A;RABL2B;RAD18;RAD9A;RALGAPB;RBFOX2;RBM12;RBM12B;RDX;RFC2;RFX5;RHBDF2;RHD;RNF144B;RNF44;RNFT1;RPL31;RPS6;RPUSD2;RRAD;RRM1;RRM2;RWDD1;SALL3;SAR1A;SDR42E1;SEMA4C;SENP5;SLC10A7;SLC11A2;SLC12A7;SLC16A9;SLC19A3;SLC20A1;SLC30A6;SLC35A4;SLC38A7;SLC5A6;SMARCAD1;SMC1A;SMCR8;SNX17;SOCS1;SOCS4;SOD2;SPATA2;SPRYD4;SREK1;SREK1IP1;STAT2;STK4;STK40;STRN;STX3;SUMO1;SUOX;SURF4;SYNJ2BP;SYT1;TBC1D19;TBC1D9B;TGFBR1;TGFBR3;TGOLN2;THBS1;THEM6;THYN1;TIAF1;TLE4;TMED4;TMED5;TMEM135;TMEM2;TMTC3;TNFRSF10B;TNFSF9;TNRC6A;TOMM20;TOMM40L;TPD52L2;TRAPPC10;TRIM71;TRMO;TSC22D2;TST;TUBB2A;TUBB4A;TXLNA;TXLNG;UBXN2B;USP24;USP38;USP47;UTP6;VCL;WASL;YAE1D1;YOD1;YWHAZ;ZBTB24;ZBTB37;ZBTB39;ZBTB5;ZBTB8OS;ZC3HAV1L;ZCCHC3;ZEB2;ZFAND4;ZNF200;ZNF264;ZNF28;ZNF280B;ZNF354B;ZNF417;ZNF443;ZNF460;ZNF526;ZNF556;ZNF566;ZNF578;ZNF584;ZNF587;ZNF609;ZNF611;ZNF644;ZNF738;ZNF746;ZNF763;ZNF774;ZNF799;ZNF8
hsa-miR-27b-3p-478270_mir	-2.501	0.224	ABCA1;ABHD17C;ABL2;ACER2;ADAMTS5;ADAR;ADD1;ADGRL1;ADORA2B;AFF4;AGPAT3;AKIRIN1;ALDOA;ALG9;ANKRD2;ANKRD40;ANKRD52;AP1G1;AP3B2;AP3D1;APEX1;APPBP2;ARL5B;ARL6IP1;ARNTL;ATN1;ATP5G3;ATP7B;ATPAF1;BAG2;BAZ2A;BDNF-AS;BEX3;BMPR2;BNIP3;BRAT1;BTG2;BUB3;C1ORF74;C2CD2L;C5ORF51;C6ORF120;C8ORF4;CAB39;CAB39L;CALM3;CAPN15;CARM1;CCNA2;CCND3;CCNG1;CCNK;CCNL2;CCNT1;CCNT2;CCNYL1;CD2AP;CDH11;CDH5;CDH6;CELF1;CELF2;CEP162;CEP192;CFDP1;CHEK2;CNN3;COASY;COPZ1;CPEB4;CPPED1;CREB1;CRISP2;CSRP2;CTNND1;CTSB;CUL1;CUL4B;CX3CL1;CYP1B1;CYP3A4;DAZAP2;DCAF7;DCUN1D4;DDI2;DDX17;DERL1;DIAPH1;DPF1;DPYD;DUSP5;DYNC2LI1;DYNLL2;DYRK3;E2F7;EDNRA;EFHD2;EGFR;EHMT2;EIF2S2;EIF5;EIF5A2;ELL2;ELMO1;EML1;EN2;ENDO

			U;ERC1;EYA4;FAM136A;FAM217B;FAM84B;FARSA;FASN;FBXO46;FCHSD2;FEM1B;FHL2;FNDC3A;FOXJ3;FOXN2;FOXO1;FRYL;FZD7;FZD9;GALNT11;GCSAM;GIPC1;GLCCI1;GNB4;GNG12;GNPNAT1;GPAM;GPATCH11;GSE1;GTF2IRD2;GYS1;H3F3B;H3F3C;HAT1;HINT1;HIP1R;HIST1H1C;HIST2H2AA3;HIST2H2AA4;HIST2H3A;HLA-DRA;HMGB3;HMGN1;HMGXB4;HNRNPA2B1;HNRNPF;HNRNPU;HOXA10;HOXB3;HOXC11;HOXD11;HRK;ICK;ID4;IER3;INPPL1;ITCH;ITSN2;JMY;KCTD12;KHDRBS1;KHSRP;KIAA1211;KIAA1551;KIF1B;KLHDC3;KMT2A;KMT2C;KRTAP13-2;LAPTM4B;LBR;LCOR;LDLR;LFNG;LIFR;LIN54;LMF2;LMNB2;LONRF1;LPCAT1;LPIN1;LRIG3;LRRC61;LYSMD3;MACC1;MANEA;MAP1B;MAPK9;MDH2;MED13;MED14;MED30;MET;METTL8;MFF;MGEA5;MIER3;MIGA2;MKNK2;MMP13;MMS22L;MPRIP;MRPS14;MRPS27;MTMR3;MYO1F;NAA50;NCBP2;NDUFC2-KCTD14;NDUFS6;NECAP1;NEURL1B;NF1;NFE2L2;NLK;NLN;NOLC1;NOTCH1;NOTCH2;NPEPPS;NR1D2;NR2F2;NR5A2;NRAS;NRBP1;NSD1;NUDT21;NUFIP2;NUP133;NUP93;NUS1;OLR1;OSBPL10;OSBPL6;OTUD4;PAIP2;PAK2;PAX3;PAX7;PAXBP1;PDHX;PDIA5;PDK1;PEG10;PGM2L1;PHB;PHC2;PHLPP2;PIGO;PINK1;PISD;PITHD1;PKNOX1;PLA2G2D;PLAGL2;PLK2;PLXND1;PMAIP1;PNKD;PNRC2;POGZ;PPARG;PPIC;PPIF;PREX1;PROX2;PRPF8;PRR3;PSAP;PSPC1;RAB14;RAB3B;RABGAP1;RAP1B;RAPGEF1;RBBP5;RET;RETREG2;RGMB;RGPD4;RGS6;RMND5A;RNF139;RNF152;RNF38;ROR1;RPL19;RPS15A;RPS24;RPTOR;RREB1;RUNX1;RYBP;SEC16A;SEC24A;SEMA6A;SEMA6D;SERP1;SERTAD3;SETD1B;SFXN1;SFXN4;SGMS1;SGPL1;SHC1;SLC16A1;SLC25A25;SLC25A44;SLC25A5;SLC26A2;SLC43A1;SLC46A3;SLC5A6;SLC6A17;SLC7A2;SMAD2;SMAD4;SNCG;SNRNP27;SNX19;SNX25;SOCS6;SOS1;SP1;SPAG9;SPATA13;SPIB;SRCAP;SRSF1;ST14;SUCO;SUPV3L1;SZRD1;TAOK1;TARDBP;TBC1D9;TGFBR1;TGFBR3;TGOLN2;THBS1;THBS2;THOC3;TIGD2;TIMM10;TLNRD1;TMBIM6;TMED5;TMEM167A;TMEM19;TMEM254;TMEM68;TMEM91;TMSB10;TMTC1;TMTC3;TNPO1;TOMM40L;TOPBP1;TOR2A;TP53INP1;TP53INP2;TPT1;TRAPPC2B;TRIM23;TROVE2;TRUB2;TSEN54;TSPYL1;TSR1;TTC3;TXNDC5;TXNIP;UBA1;UBE2D1;UBE2G1;UBR5;UBXN1;UBXN11;UCA1;UGCG;USP25;USP46;UTP14A;VDR;VEGFC;VPS26B;WASL;WDCP;WDFY1;WDTC1;WEE1;WNK1;WNT9B;YIPF4;ZADH2;ZBTB18;ZBTB20;ZBTB37;ZC3H11A;ZFHX3;ZFP1;ZFP36;ZFP36L1;ZFP36L2;ZFX;ZMYND11;ZNF106;ZNF148;ZNF175;ZNF384;ZNF45;ZNF460;ZNF618;ZNF627;ZNF800
hsa-miR-652-3p-478189_mir	-2.058	0.229	ACTG1;ACTN4;ADM2;AGAP3;AGO1;AGO2;AGO3;ANKRD9;AP1G1;ARCN1;ATF7IP;ATP11B;ATP11C;ATP5B;BSG;BTF3;CACNG8;CAPZB;CARS;CBS;CCDC74B;CD46;CDC42EP1;CDKN2AIP;CEBPG;CHST1;CHST6;CNN3;CNOT3;COX20;CSNK1A1;CSNK2A1;CTC1;CYTH2;DDX39A;DGCR8;DOCK11;EEF1A1;EIF4ENIF1;ELOVL1;EML4;FAM120AOS;G6PC3;GAN;GCM1;GDPD5;GEN1;GGCX;GLMP;GPR161;GRK6;GRPEL1;GTF3C5;H3F3B;HACD1;HIST1H1C;HIST1H1E;HIST1H2BB;HIST1H2BD;HIST1H2BH;HIST1H2BJ;HIST1H3B;HIST2H3A;HIST2H4B;HMGB1;HNRNPAB;HOXA9;HSPA1B;IARS;IMMT;IPO13;IRS4;ISOC1;KDM2B;KIF1A;KMT2C;LAT2;LBHD1;LLGL1;LRRC1;MASTL;MCTS1;MGST1;MORF4L2;MRAS;MRPL36;MSI2;MT-CO2;MT-ND1;MT-ND4;MTA3;MXRA7;NDE1;NFAT5;NFE2L1;NOM1;NUP98;NXN;PCSK7;PHF12;PNPLA4;PNPT1;POLR2A;POM121;PPIL4;PRKAA1;PRPF38A;QKI;QSOX1;RAC1;RAP1GAP2;RNF152;RPL18A;RPL21;RPL26;RPL27;RPL29;RPL32;RPL35A;RPL4;RPS16;RPS19;RPS29;RPS6;SAT1;SEC13;SEPT2;SERBP1;SERF1B;SF3A1;SLC12A6;SLC31A1;SLC7A1;SMAD2;SMAP2;SNX5;SRM;SRPK1;STK4;SUPT6H;TAZ;TCP1;TMED5;TMEM107;TMEM250;TMEM33;TNRC6A;TUBA1B;UBE2I;UHRF1BP1;USP10;VPS37B;VPS41;YBX3;YIPF6;ZBTB4;ZBTB44;ZBTB8A;ZEB1;ZNF431;ZNF567
hsa-miR-10a-5p-479241_mir	-4.357	0.236	ABCB7;ABCG2;ACAA1;ACAD8;ACOT13;ACTG1;ACVR2A;ADCY9;ADGRV1;ADPRHL2;AGBL2;AGER;AHCY;AHCYL2;AHSA2;AJUBA;ALDH1A2;ALKBH4;AMMECR1L;AMPD1;AMPD2;ANKRD33B;ANO6;ANP32E;AP5S1;API5;APOC3;APRT;AQP12B;ARF3;ARFGEF1;ARHGAP18;ARHGAP19;ARMCX3;ARPC5;ARSK;ASB1;ATG3;ATIC;ATP1A2;ATP1B4;ATP5F1;AXL;B3GNT5;BAMBI;BCKDK;BCL2L11;BCL2L13;BCL6;BCR;BDNF;BIRC5;BLOC1S5;BORCS5;BTAF1;BTRC;C11ORF63;C12ORF76;C17ORF105;C1GALT1;C21ORF59;C2CD2;C3ORF36;C5;C6ORF89;CAB39L;CADM1;CARD8;CARHSP1;CCDC151;CCDC8;CCNG1;CCS;CD3D;CD59;CDC6;CDK19;CDK8;CENPL;CEP128;CHAF1B;CHDH;CHFR;CHL1;CHMP1B;CHRNA5;CLN8;CMPK1;CNKSR3;CNOT6;CNST;CNTLN;COL4A2;COL4A3;COL6A2;COPA;COX7B;CPED1;CREB5;CRIPT;CRK;CRLF3;CRY2;CSGALNACT1;CSNK2A1;CSRNP3;CTNND1;CUL3;CYP8B1;DDX42;DDX54;DGAT1;DLG4;DNAJB1;DPYSL2;DUSP3;DVL1;DVL3;E2F1;E2F7;EBNA1BP2;EEF2;EHD4;EIF1;EIF1AD;EIF4H;ELOVL2;ELOVL7;EMB;EPB41L2;EPHA4;ERLIN1;ERMN;ESPL1;ETS1;EXOSC2;EXTL3;FAHD1;FAM118A;FAM168A;FAM208A;FAM219B;FASN;FAT2;FBXO31;FEM1A;FEM1B;FGFR1;FHL2;FOXRED2;FRRS1;FUS;FUT1;FZD2;GALNT1;GATAD2A;GEMIN5;GK5;GLB1L3;GLOD4;GNAL;GOLGA8A;GORASP2;GOSR2;GP1BA;GPCPD1;GPR63;GRK6;GSR;GSS;GTF3C2;H3F3B;H3F3C;H6PD;HHIP;HIVEP3;HK1;HLA-E;HMGN2;HNRNPF;HOXA1;HOXA3;HOXB3;HOXD13;HSP90AA1;HSPA1B;HSPA4;HSPA8;ID4;IER3IP1;IGSF1;IGSF9B;IL12A;IL23A;INTS2;IPCEF1;IQCB1;IRGQ;KANK1;KCTD11;KIAA0100;KIAA1143;KIAA1147;KIAA1551;KIF21A;KIF3B;KLHDC8A;KLHL23;KLHL6;KXD1;LAMC1;LCA5;LEMD3;LHFPL3;LILRA2;LIMA1;LIX1L;LMBR1L;LPIN3;LRFN1;LRP3;LSS;LYPD6;MAP3K7;MAPK8;MAPK8IP1;MAPKAPK5;MARS;MAVS

			;MBD1;MBD4;MCPH1;MCTS1;MCU;MEAF6;MED12;METTL7A;MIS12;MKNK2;MLNR;MMP14;MOB3C;MRC2;MRPS14;MSANTD1;MSL3;MTF2;MTR;MTRNR2L10;MTRNR2L11;MTRNR2L3;MTRNR2L7;MTX3;MYEF2;NACC2;NAP1L1;NCBP3;NCOR2;NCSTN;NDUFB5;NDUFB6;NEK7;NF2;NIPSNAP2;NOB1;NOD2;NOMO1;NOP16;NOP2;NPTX1;NR1D2;NR2C2;NT5DC1;NT5DC3;NTMT1;NUB1;NUDT4;NUP205;NUP37;OAZ1;OCRL;OMA1;ONECUT3;OPA3;ORAI2;ORC1;PABPC1;PABPC3;PAFAH1B1;PAICS;PANK2;PANX1;PAPD5;PARL;PDK3;PDPK1;PFAS;PHAX;PHB2;PHF20;PIAS3;PIK3CG;PLA2G2C;PLA2G4A;PLA2G4F;PLEKHB2;PLSCR1;POC1A;POLR2A;POLR2H;POLR3A;POU2F2;PPFIBP1;PPM1G;PPP1R12A;PPP1R15B;PRDX2;PRPF8;PRRC2C;PSD4;PSMB1;PSMD13;PTEN;PTPRG;PTPRT;PUM2;PWP1;PYGL;QPCT;RAB15;RAB18;RABL6;RALBP1;RAP2A;RAPGEF2;RBM12B;RBM17;RELN;RIOK2;RIOK3;RLIM;RNF123;RNF2;RNF213;RORA;RPL15;RPRD1A;RPS15A;RPS29;RPS9;RRP1B;RTKN2;RUNDC3B;S1PR2;SAPCD2;SCAP;SCD;SEPT9;SERBP1;SERPINE1;SF3B3;SF3B4;SFPQ;SFRP1;SFT2D2;SHBG;SLC11A2;SLC24A4;SLC25A13;SLC25A43;SLC25A5;SLC26A2;SLC2A3;SLC3A2;SLC41A3;SLC48A1;SLC5A5;SMCHD1;SNTB2;SNX4;SON;SORD;SPARC;SPECC1;SREBF1;SRP19;SRSF1;STT3A;SUPT6H;SYMPK;SYNJ1;SYNPO;TAF15;TCF15;TENM3;TFAP2C;TFPI;TGFB3;THUMPD1;TIAM1;TIMM50;TLE3;TMED4;TMED5;TMEM101;TMEM106B;TMEM109;TMEM127;TMEM170A;TMEM179B;TPI1;TPM4;TRA2B;TRAPPC12;TRIM2;TRPM7;TSPAN33;TTC7A;TTYH3;TUBA1A;UBE2D2;UBE2Z;UBE3C;UBN2;UBTF;UBXN7;UGDH;UHRF1;URGCP;USF2;USP11;USP6NL;USP9X;VDAC1;WDR13;WDR74;WDR77;WEE1;XIAP;XPNPEP3;XPO7;XRN1;YES1;YOD1;ZBTB10;ZBTB8A;ZC3H18;ZFAND5;ZFP30;ZNF223;ZNF280B;ZNF318;ZNF329;ZNF394;ZNF445;ZNF460;ZNF502;ZNF574;ZNF592;ZNF618;ZNF629;ZNF708;ZNF878
hsa-miR-224-5p-477986_mir	-1.303	0.236	ABCC1;ADAP1;ADNP2;AKIRIN1;AP2M1;API5;APLN;ARL4D;ARSB;ASCC1;B3GALNT2;BCL2;C12ORF49;C17ORF82;C21ORF91;C8ORF46;CACNG7;CASP3;CASP7;CCND1;CD40;CDC42;CDH1;CHAC1;CLDN12;CNTN1;COASY;COQ7;COX6B1;CSTF2;CXCR4;DIDO1;DPYSL2;EDNRA;EFNA3;ENC1;EYA4;F8A2;F8A3;FAM84B;FEM1B;FOSB;FRYL;GABARAPL3;GALNT1;GGA2;GIGYF1;GNA11;GNB4;GORAB;GRPEL2;GSK3B;HIPK3;HNRNPC;HNRNPF;HOXB3;HOXD10;HSP90AA1;IGF1R;IL20RB;ITGB3BP;KBTBD8;KCTD2;KDELR1;KLK10;KRAS;KRT74;LHFPL2;LONRF2;MAP2K2;MBD2;MED13;MITF;MOCS2;MPDU1;MTOR;MYLIP;NCOA3;NCOA6;NIT1;NPTN;NUFIP2;PAK2;PDGFRB;PEBP1;PELI1;PHLPP1;PHLPP2;PIGO;PRRG4;PSMD3;PTX3;QKI;RAB15;RAB9B;RABGAP1;RAC1;RASSF8;RBM3;RETSAT;RPL15;SEC14L5;SERPINE1;SERPINF2;SLC12A5;SLC41A1;SLC46A3;SLC7A14;SMAD4;STARD5;TADA2B;TCEAL1;TIAL1;TMTC3;TNRC6A;TOM1L2;TOMM20;TPD52;TPR;TRIB1;TXNIP;USP6NL;VGLL3;WRN;XPO1;YES1;ZKSCAN8;ZNF135;ZNF207;ZNF573;ZNF585B;ZWINT
hsa-miR-16-5p-477860_mir	-0.531	0.245	AAAS;AADAT;ABCB7;ABCC1;ABCC4;ABCC6;ABCF1;ABCF2;ABHD10;ABHD2;ABL2;ACBD5;ACOT8;ACOX1;ACP2;ACTB;ACTG1;ACTN1;ACTN4;ACTR1A;ACTR2;ACTR3B;ACVR2A;ADAD2;ADGRE5;ADGRL1;ADK;ADORA2A;ADORA3;ADRA2B;ADSS;AFF4;AFG3L2;AGER;AGK;AGO2;AGO4;AGPAT5;AGRN;AHCYL1;AHCYL2;AHNAK2;AIFM2;AIMP1;AK2;AKAP11;AKAP13;AKR1B10;AKT3;ALDH18A1;ALDH2;ALDH3B1;ALG2;ALG3;ALKAL2;AMER1;AMOT;AMOTL1;AMPD1;ANAPC13;ANAPC16;ANKLE2;ANKMY1;ANKRD13B;ANKRD17;ANKRD36;ANPEP;AP2A1;AP2B1;AP2M1;AP3D1;AP3M1;AP5Z1;APLNR;APP;AQP12B;ARCN1;ARG2;ARHGAP12;ARHGAP32;ARHGDIA;ARIH1;ARL10;ARL2;ARL2BP;ARL3;ARMC12;ARMCX2;ARPC5L;ASB6;ASCC1;ASCC3;ASGR2;ASH1L;ASNS;ASPH;ASXL1;ASXL2;ATAD5;ATF7;ATG14;ATG9A;ATL2;ATOX1;ATP13A3;ATP5A1;ATP5C1;ATP5G3;ATP6V0E1;ATP6V1B2;ATP6V1E1;ATP8A2;ATXN2L;ATXN7L3;ATXN7L3B;AUP1;AURKAIP1;AURKB;AVL9;AXIN2;B3GNT2;B4GALT1;BACE1;BAG4;BAG6;BAMBI;BAZ1B;BAZ2A;BCAS2;BCCIP;BCL11B;BCL2;BCL2L12;BCL7A;BDNF;BEX3;BFAR;BHLHE40;BIRC5;BMI1;BMS1;BNC2;BRAT1;BRCA1;BSG;BSPRY;BTAF1;BTBD2;BTF3;BTG2;BTN3A3;BTRC;BYSL;BZW1;C11ORF24;C15ORF39;C16ORF58;C16ORF72;C17ORF75;C17ORF80;C19ORF54;C1ORF21;C1ORF226;C1ORF56;C21ORF62;C2ORF42;C2ORF74;C3ORF36;C6ORF106;C8ORF44;CA12;CA8;CAAP1;CABIN1;CACNA2D1;CACNB2;CACUL1;CADM1;CALR;CALU;CAMK2G;CAMKK2;CAMKV;CAMSAP1;CAMSAP3;CANX;CAPRIN1;CAPZA2;CARD10;CARD19;CARD8;CARM1;CASK;CASKIN1;CBFA2T3;CBX2;CBX4;CBX6;CCDC58;CCDC59;CCDC80;CCDC83;CCDC88C;CCND1;CCND2;CCND3;CCNE1;CCNE2;CCNJ;CCNT1;CCNT2;CCPG1;CCT3;CCT6B;CCT8;CD180;CD274;CD2AP;CD44;CD55;CDADC1;CDC123;CDC14B;CDC20;CDC23;CDC25A;CDC27;CDC37;CDC37L1;CDC42SE2;CDC5L;CDCA4;CDCA8;CDIPT;CDK1;CDK17;CDK5RAP1;CDK6;CDK9;CDKN1A;CDKN2A;CDKN2AIPNL;CDS2;CDV3;CENPF;CENPJ;CEP55;CEP63;CFAP45;CFL2;CHAC1;CHD3;CHEK1;CHERP;CHIC1;CHMP3;CHMP4B;CHORDC1;CHPT1;CHUK;CIB1;CKAP5;CLASP1;CLDN2;CLEC2D;CLIP2;CLIP4;CLNS1A;CLSPN;CLTC;CLU;CLUH;CMPK1;CMTM4;CNKSR3;CNN3;CNNM2;CNOT7;CNP;CNPY3;COA6;COA7;COL4A1;COL4A2;COMMD10;COMT;COPS5;COPS7B;COQ3;CPEB2;CPEB3;CPNE1;CPNE8;CPSF7;CPT1A;CREB3L2;CREBL2;CREBRF;CREG1;CRHBP;CRIM1;CRK;CRKL;CSDE1;CSGALNACT1;CSHL1;CSNK1E;CTDSPL;CTSD;CUL2;CUL3;CUL4A;CXORF38;CXORF40B;CYB561A3;CYB5R1;CYCS;CYLD;CYP26B1;CYP27B1;DAP3;DCAF13;DCAF17;DCAF7;DCTN5;DCUN1D5;DDHD2;DDN;DDX21;DDX31;DDX3X;DDX3Y;DDX41;DDX52;DDX54;DDX6;DECR1;DENND6A;DESI1;DHTKD1;DHX30;DHX35;DHX36;DHX37;DHX38;DHX

			8;DIABLO;DIAPH1;DICER1;DIEXF;DIXDC1;DKC1;DLC1;DLD;DLGAP3;DMAP1;DMD;DMPK;DMRT2;DMTF1;DNAAF5;DNAJA1;DNAJA2;DNAJA4;DNAJB1;DNAJB4;DNAJC1;DNAJC10;DNAJC15;DNAJC2;DNAJC9;DNTTIP2;DOCK11;DOCK5;DOCK9;DPP8;DSCR3;DSP;DTD1;DUSP14;DYNLL2;DYRK3;E2F3;E2F7;EARS2;ECHDC1;EDC3;EDC4;EEF1A1;EEF1G;EEF2;EEF2K;EFNB2;EFTUD2;EGFR;EGLN2;EIF1;EIF1AX;EIF2A;EIF2B2;EIF2B5;EIF3A;EIF3C;EIF3CL;EIF3E;EIF3F;EIF3H;EIF3K;EIF3M;EIF4B;EIF4E;EIF4G1;EIF4G3;EIF5;EIF5B;ELAC2;ELK4;ELOVL1;ELOVL5;ELP3;EMC6;EML4;EN2;ENPP4;ENTPD1;ENTPD6;ENTPD7;ENY2;EPC1;EPHA2;EPM2AIP1;ERLIN2;ETFRF1;ETNK1;EXD2;EXT1;EZH1;F2;FAM103A1;FAM122A;FAM122B;FAM122C;FAM129B;FAM155B;FAM168A;FAM189B;FAM208A;FAM229B;FAM69A;FAM89A;FAM96B;FASN;FASTKD1;FASTKD2;FAT2;FAT3;FBXL18;FBXL20;FBXO3;FBXO41;FBXW11;FBXW7;FCF1;FDXR;FECH;FEM1C;FGF2;FGFR1;FGFR4;FKBP1A;FLCN;FLNA;FLOT2;FNBP1;FNDC3B;FNTA;FOXK1;FRYL;FSCN2;FTH1;FTL;FUBP1;FURIN;FZD6;FZD9;G3BP2;GABARAP;GABARAPL1;GABPA;GALNT1;GALNT3;GALNT7;GANAB;GARS;GATAD2A;GCC1;GEMIN4;GEMIN5;GFM1;GFPT1;GGA3;GLB1L3;GLP2R;GLRX;GLS2;GLT8D1;GNAL;GNAT1;GNB1;GNB2;GNG12;GNL3L;GOLGA5;GOLGA7;GOLPH3L;GOLT1B;GOSR1;GOT2;GPAA1;GPAM;GPATCH4;GPATCH8;GPR157;GPR180;GPR27;GPR55;GPRC5A;GRAMD2B;GRB2;GRPEL2;GRWD1;GSG1;GSK3B;GSTM4;GSTT2B;GTF2H1;GTF3C1;GTF3C2;GTF3C3;GTF3C4;GTPBP1;GTPBP4;GTPBP8;H2AFX;H3F3B;HACE1;HARS;HARS2;HAUS3;HCFC2;HDGF;HDHD2;HDHD5;HEATR1;HEATR3;HELZ;HERC6;HEYL;HGF;HIGD1A;HIST1H1C;HIST1H2AJ;HIST1H2BC;HIST1H2BK;HIST1H4B;HIST2H2BE;HM13;HMBOX1;HMGA1;HMGA2;HMGN1;HMOX1;HNRNPA1;HNRNPA1L2;HNRNPA2B1;HNRNPDL;HNRNPF;HNRNPL;HOXA10;HOXA3;HOXC8;HOXD13;HPF1;HSD17B8;HSDL2;HSP90AA1;HSP90B1;HSP90B2P;HSPA1A;HSPA1B;HSPA4L;HSPA5;HSPA8;HSPA9;HSPBP1;HSPD1;HSPE1-MOB4;HSPH1;HTRA1;HTT;HYAL3;HYOU1;IARS;IBTK;IDH3A;IDS;IER2;IER3IP1;IFNG;IFRD1;IFRD2;IFT74;IGF1R;IGF2R;IGSF1;IKZF4;IL12B;IL36RN;IMPAD1;IMPDH2;INF2;INO80D;INSL6;INTS5;IPO11;IPO4;IPO5;IPO7;IPPK;IRAK1BP1;IRAK3;IRF4;IRS4;ITGA2;ITGB4;ITPR1;IVNS1ABP;IWS1;JAK2;JARID2;JPT2;JUN;KANK1;KANSL3;KATNAL1;KCNC4;KCND3;KCNG3;KCNN4;KCNS2;KDM2A;KDR;KDSR;KIAA0368;KIAA0895;KIAA1456;KIAA2013;KIDINS220;KIF14;KIF1B;KIF23;KIF2A;KIF2C;KIF3B;KIF5A;KIF5B;KIN;KLC2;KLF14;KLHDC10;KLHL15;KLHL34;KLHL40;KMT2A;KMT2D;KPNA1;KPNA2;KPNA3;KRAS;KRT33B;L1CAM;L2HGDH;LAMB1;LAMB3;LAMC1;LAMP2;LAMTOR1;LAMTOR2;LAMTOR3;LAMTOR4;LAMTOR5;LANCL1;LAPTM4B;LARP1;LARP4;LATS1;LDAH;LIG4;LIMA1;LIN7C;LITAF;LMAN2L;LMF2;LMO7;LMTK3;LONP1;LONRF2;LRIF1;LRIG2;LRPPRC;LRRC57;LRRFIP2;LSG1;LSM10;LSM11;LSM5;LUC7L;LUC7L3;LURAP1L;LUZP1;LY6K;LYAR;LYPD2;LYPLA2;MACF1;MAFK;MAIP1;MALSU1;MAP2K3;MAP3K7;MAP4;MAP4K2;MAP7;MAPKAPK2;MARCH6;MARS;MBD4;MCFD2;MCL1;MCM3AP-AS1;MCU;MDN1;MED11;MED12;MED13;MED24;MEGF8;MEPCE;MERTK;METAP2;METTL13;MFN2;MGAT4A;MIB1;MICU2;MIGA1;MINK1;MIS12;MKI67;MKX;MLF2;MLLT1;MLLT11;MLLT6;MLXIP;MMP25;MMS19;MOB3C;MOB4;MORF4L1;MOV10;MPDU1;MRC2;MRFAP1;MRM3;MRPL1;MRPL10;MRPL12;MRPL20;MRPL21;MRPL3;MRPL40;MRPS10;MRPS12;MRPS14;MRPS2;MRPS23;MRPS25;MRPS31;MRPS35;MRRF;MSANTD4;MSH2;MSL1;MSL3;MT-CO1;MTFR1L;MTHFD1L;MTHFD2;MTHFR;MTMR3;MTMR4;MTOR;MYB;MYC;MYO19;MYO5A;MYO5B;N4BP1;NAA10;NAA15;NAA25;NACA;NAMPT;NAPG;NARF;NARS;NARS2;NAT8L;NAV2;NCAPD2;NCAPG;NCKAP1;NCKAP5L;NCOR2;NCSTN;NDUFA4;NDUFA9;NDUFAF4;NEGR1;NEMF;NEURL4;NFIC;NFKB1;NHLRC3;NIN;NIPAL2;NISCH;NLE1;NMD3;NNT;NOB1;NOC3L;NOC4L;NOL11;NOL4L;NOP10;NOP14;NOP53;NOTCH2;NPR3;NPRL3;NR1I2;NR2C2;NR6A1;NRP1;NRXN1;NSF;NSUN2;NT5C3A;NT5DC1;NT5DC2;NTHL1;NUCKS1;NUDT3;NUFIP2;NUP155;NUP160;NUP50;NUP98;NXPH2;OCRL;ODF2L;OGDH;OGT;OIP5;OMA1;ONECUT2;OPRM1;ORAI2;ORC4;OSBPL3;OSCAR;OSGEPL1;OTOL1;OTUB1;OTUD7B;OTULIN;OXNAD1;PA2G4;PABPC4;PACSIN3;PAFAH1B1;PAFAH1B2;PAG1;PAGR1;PAK1IP1;PAK2;PANK1;PANX1;PAPOLG;PAQR3;PCDHGB4;PCK2;PCMT1;PDCD1;PDCD4;PDCD6IP;PDE3B;PDE4D;PDF;PDHX;PDIA6;PDIK1L;PDK4;PDLIM5;PDPR;PDXK;PELO;PEX12;PEX13;PEX14;PGD;PGLYRP1;PHC3;PHEX;PHF19;PHIP;PHKA1;PHKB;PHLDA2;PHLDB2;PHLPP2;PHYHIP;PI4K2B;PIK3R1;PIM1;PIP4K2B;PISD;PLAG1;PLAUR;PLCXD2;PLD3;PLEC;PLEKHA1;PLEKHB2;PLEKHM2;PLEKHN1;PLK1;PLPBP;PLPP3;PLRG1;PLSCR4;PMS1;PNISR;PNN;PNP;PNPLA6;PNPO;PNRC2;POFUT1;POLB;POLDIP2;POLDIP3;POLE4;POLR1C;POLR2A;POLR2E;POLR3A;POM121C;PON2;POTEF;POU2AF1;PPA1;PPAN;PPIF;PPIG;PPIL1;PPIP5K2;PPM1A;PPM1D;PPP1R11;PPP2R1A;PPP2R1B;PPP2R5C;PPP4R3B;PPP6C;PPP6R3;PPT1;PRDM4;PRELID3B;PRICKLE2;PRIM1;PRIMPOL;PRKAA1;PRKAB2;PRKAR1A;PRKAR2A;PRKCD;PRNP;PRPF8;PRPSAP1;PRR12;PRR3;PRRC2C;PRSS21;PSAT1;PSKH1;PSMB5;PSMC1;PSMC2;PSMD11;PSMD12;PSME3;PSME4;PSPH;PTCD3;PTGS2;PTPDC1;PTPN18;PTPN3;PTPRD;PTPRJ;PTPRT;PTRH1;PUM1;PURA;PVR;PWWP2A;PYGB;QSOX2;RAB11FIP2;RAB12;RAB15;RAB1A;RAB1B;RAB21;RAB23;RAB30;RAB3IP;RAB40B;RAB9B;RABGGTB;RACGAP1;RAD23B;RAD51C;RAF1;RALGAPB;RAN;RANGAP1;RAP2C;RAPH1;RARB;RARS;RASEF;RASSF2;RASSF5;RBBP6;RB

			M15B;RBM28;RBM6;RBMS1;RBMS3;RBPJ;RCAN3;RCC1L;RCL1;RCOR1;RECK;REL;RELT;REXO1;RFK;RFT1;RFWD2;RHOF;RHOT1;RHOV;RIC1;RICTOR;RIDA;RIMS3;RIOK3;RIOX2;RMDN1;RNASEH1P1;RNASEL;RNF111;RNF138;RNF144B;RNF149;RNF168;RNF217;RNF38;RNMT;RNPS1;ROGDI;RP1L1;RPH3AL;RPL10;RPL10L;RPL12;RPL13;RPL14;RPL21;RPL27A;RPL3;RPL30;RPL31;RPL36;RPL4;RPL5;RPL6;RPL9;RPLP0;RPLP1;RPRD1B;RPRD2;RPS17;RPS2;RPS24;RPS25;RPS27;RPS3;RPS3A;RPS5;RPS6;RPS6KA3;RPS6KB1;RPSA;RRP12;RRP15;RRP36;RRP9;RS1;RSL1D1;RTN4;RUBCN;RUNDC3B;RUNX1T1;S100A11P1;SACM1L;SALL1;SARS;SART1;SAV1;SBF1;SBNO1;SCAF4;SCAMP3;SCAMP4;SCAMP5;SDHAF2;SEC11A;SEC24A;SEC24B;SEC61A1;SEC61A2;SEH1L;SELENOI;SEMA4C;SENP6;SEPT2;SERBP1;SERINC5;SERPINB5;SERPINE2;SESTD1;SETD1B;SETD5;SF3A3;SF3B3;SFXN1;SGK3;SGTA;SH3BP4;SHOC2;SIDT2;SIK1;SIPA1L2;SIRT4;SKAP2;SKI;SLC11A2;SLC12A2;SLC16A3;SLC19A1;SLC1A5;SLC25A12;SLC25A22;SLC25A29;SLC25A32;SLC25A38;SLC25A39;SLC25A6;SLC27A4;SLC29A1;SLC2A3;SLC30A1;SLC35A1;SLC35A4;SLC35B2;SLC35B3;SLC35E2B;SLC38A1;SLC38A2;SLC38A5;SLC39A10;SLC39A14;SLC39A9;SLC3A2;SLC4A1AP;SLC4A2;SLC6A4;SLC7A1;SLC7A5;SLC9A2;SLC9A6;SLCO3A1;SLFN13;SLIRP;SMAD1;SMAD3;SMAD5;SMAD7;SMARCA4;SMDT1;SMPD4;SMURF1;SMURF2;SNCG;SND1;SNRPA1;SNRPB2;SNRPC;SNTB2;SNX11;SNX12;SNX15;SNX16;SNX6;SOCS2;SOCS3;SOCS5;SON;SOWAHC;SOX5;SOX6;SP1;SPATA2;SPATA5;SPCS3;SPEN;SPOUT1;SPRED1;SPRYD3;SPTBN2;SPTLC1;SQSTM1;SREK1;SRP19;SRP68;SRP72;SRPK1;SRPRA;SRPRB;SRSF1;SSRP1;SSSCA1;SSU72;STAU1;STEAP3;STIP1;STK33;STK38;STRADB;STRAP;STT3B;STX17;STX4;STXBP3;SUCLA2;SUN1;SUPT16H;SUPT5H;SYF2;SYNJ1;SYNRG;SYPL1;SYT11;SZRD1;TACO1;TADA2B;TAF13;TAF15;TAF9;TAOK1;TARBP2;TASP1;TBC1D14;TBC1D20;TBCCD1;TBL1XR1;TBL3;TBP;TBPL1;TBRG1;TBRG4;TCF3;TCFL5;TCP1;TDRD3;TECPR2;TELO2;TEP1;TERF2IP;TES;TET3;TEX15;TFAP2A;TFAP4;TFB1M;TFPI;TFRC;TGFBI;TGFBR3;TGOLN2;THAP7;THEM4;THRAP3;TIA1;TIGD3;TIMM10B;TIMM13;TIMM17A;TIMP3;TKTL1;TLE4;TLK1;TLL1;TM4SF1;TM7SF3;TM9SF2;TMBIM6;TMCC1;TMED1;TMED7;TMEM100;TMEM109;TMEM126A;TMEM135;TMEM138;TMEM154;TMEM161B;TMEM168;TMEM189;TMEM189-UBE2V1;TMEM245;TMEM251;TMEM255A;TMEM41A;TMEM43;TMEM69;TMEM87A;TMTC3;TNFAIP2;TNFRSF10A;TNFRSF12A;TNFSF9;TNK1;TNPO1;TNPO3;TNRC6B;TOB2;TOMM34;TOR1A;TOR4A;TOX4;TP53;TPBG;TPI1;TPM2;TPM3;TPPP3;TPT1;TRAF4;TRAK1;TRAM1;TRIM32;TRIM35;TRIM4;TRIM44;TRMT1;TRMT10C;TRMT13;TRUB2;TSC22D2;TSPAN3;TSR1;TTC1;TTC17;TTF2;TTLL12;TTLL5;TUBA1A;TUBA1C;TUBB;TUBB2A;TUBB3;TUBB4B;TUBGCP2;TXLNG;TXN2;TXNIP;TXNL1;U2SURP;UBE2C;UBE2H;UBE2Q1;UBE2Q2;UBE2S;UBE2V1;UBE2Z;UBE3C;UBE4A;UBFD1;UBN2;UBR3;UBXN2B;UCA1;UFC1;UFSP2;UGDH;UGP2;UGT2B4;UGT8;ULK1;UNG;UQCC3;USMG5;USP15;USP3;USP31;USP42;USP48;USP53;USP7;USP8;USP9X;UTP14A;UTP15;UTP20;UTP23;UTP3;UTP4;UXT;VAMP8;VASN;VAV2;VCL;VEGFA;VEZT;VIM;VKORC1;VMA21;VMP1;VOPP1;VPS33B;VPS45;VPS4A;VSIR;VTI1B;WARS;WBP11;WDR13;WDR18;WDR3;WDR43;WDR5B;WDR75;WEE1;WIPF1;WIPI2;WNK3;WNT3A;WNT4;WNT5A;WT1;XKR7;XKR8;XPNPEP3;XPO1;XPO4;XPO6;XPO7;XPOT;XYLT1;YAP1;YARS2;YBX1;YBX3;YIF1B;YIPF2;YIPF4;YIPF6;YRDC;YTHDC1;YWHAH;YWHAQ;YY1AP1;ZBTB10;ZBTB16;ZBTB2;ZBTB33;ZBTB34;ZBTB5;ZC3H11A;ZCCHC3;ZDHHC16;ZEB2;ZFHX3;ZFHX4;ZFP28;ZFPL1;ZMAT3;ZNF207;ZNF267;ZNF275;ZNF280C;ZNF284;ZNF367;ZNF384;ZNF391;ZNF449;ZNF460;ZNF559;ZNF585B;ZNF598;ZNF620;ZNF622;ZNF638;ZNF644;ZNF691;ZNF704;ZNF791;ZNF827;ZNF91;ZNRF1;ZNRF2;ZNRF3;ZRANB1;ZRANB2;ZYX;ZZEF1
hsa-miR-16-5p-477860_mir	-0.531	0.245	AAAS;AADAT;ABCB7;ABCC1;ABCC4;ABCC6;ABCF1;ABCF2;ABHD10;ABHD2;ABL2;ACBD5;ACOT8;ACOX1;ACP2;ACTB;ACTG1;ACTN1;ACTN4;ACTR1A;ACTR2;ACTR3B;ACVR2A;ADAD2;ADGRE5;ADGRL1;ADK;ADORA2A;ADORA3;ADRA2B;ADSS;AFF4;AFG3L2;AGER;AGK;AGO2;AGO4;AGPAT5;AGRN;AHCYL1;AHCYL2;AHNAK2;AIFM2;AIMP1;AK2;AKAP11;AKAP13;AKR1B10;AKT3;ALDH18A1;ALDH2;ALDH3B1;ALG2;ALG3;ALKAL2;AMER1;AMOT;AMOTL1;AMPD1;ANAPC13;ANAPC16;ANKLE2;ANKMY1;ANKRD13B;ANKRD17;ANKRD36;ANPEP;AP2A1;AP2B1;AP2M1;AP3D1;AP3M1;AP5Z1;APLNR;APP;AQP12B;ARCN1;ARG2;ARHGAP12;ARHGAP32;ARHGDIA;ARIH1;ARL10;ARL2;ARL2BP;ARL3;ARMC12;ARMCX2;ARPC5L;ASB6;ASCC1;ASCC3;ASGR2;ASH1L;ASNS;ASPH;ASXL1;ASXL2;ATAD5;ATF7;ATG14;ATG9A;ATL2;ATOX1;ATP13A3;ATP5A1;ATP5C1;ATP5G3;ATP6V0E1;ATP6V1B2;ATP6V1E1;ATP8A2;ATXN2L;ATXN7L3;ATXN7L3B;AUP1;AURKAIP1;AURKB;AVL9;AXIN2;B3GNT2;B4GALT1;BACE1;BAG4;BAG6;BAMBI;BAZ1B;BAZ2A;BCAS2;BCCIP;BCL11B;BCL2;BCL2L12;BCL7A;BDNF;BEX3;BFAR;BHLHE40;BIRC5;BMI1;BMS1;BNC2;BRAT1;BRCA1;BSG;BSPRY;BTAF1;BTBD2;BTF3;BTG2;BTN3A3;BTRC;BYSL;BZW1;C11ORF24;C15ORF39;C16ORF58;C16ORF72;C17ORF75;C17ORF80;C19ORF54;C1ORF21;C1ORF226;C1ORF56;C21ORF62;C2ORF42;C2ORF74;C3ORF36;C6ORF106;C8ORF44;CA12;CA8;CAAP1;CABIN1;CACNA2D1;CACNB2;CACUL1;CADM1;CALR;CALU;CAMK2G;CAMKK2;CAMKV;CAMSAP1;CAMSAP3;CANX;CAPRIN1;CAPZA2;CARD10;CARD19;CARD8;CARM1;CASK;CASKIN1;CBFA2T3;CBX2;CBX4;CBX6;CCDC58;CCDC59;CCDC80;CCDC83;CCDC88C;CCND1;CCND2;CCND3;CCNE1;CCNE2;CCNJ;CCNT1;CCNT2;CCPG1;CCT3;CCT6B;CCT8;CD180;CD274;CD2AP;CD44;CD55;CDADC1;

			CDC123;CDC14B;CDC20;CDC23;CDC25A;CDC27;CDC37;CDC37L1;CDC42SE2;CDC5L;CDCA4;CDCA8;CDIPT;CDK1;CDK17;CDK5RAP1;CDK6;CDK9;CDKN1A;CDKN2A;CDKN2AIPNL;CDS2;CDV3;CENPF;CENPJ;CEP55;CEP63;CFAP45;CFL2;CHAC1;CHD3;CHEK1;CHERP;CHIC1;CHMP3;CHMP4B;CHORDC1;CHPT1;CHUK;CIB1;CKAP5;CLASP1;CLDN2;CLEC2D;CLIP2;CLIP4;CLNS1A;CLSPN;CLTC;CLU;CLUH;CMPK1;CMTM4;CNKSR3;CNN3;CNNM2;CNOT7;CNP;CNPY3;COA6;COA7;COL4A1;COL4A2;COMMD10;COMT;COPS5;COPS7B;COQ3;CPEB2;CPEB3;CPNE1;CPNE8;CPSF7;CPT1A;CREB3L2;CREBL2;CREBRF;CREG1;CRHBP;CRIM1;CRK;CRKL;CSDE1;CSGALNACT1;CSHL1;CSNK1E;CTDSPL;CTSD;CUL2;CUL3;CUL4A;CXORF38;CXORF40B;CYB561A3;CYB5R1;CYCS;CYLD;CYP26B1;CYP27B1;DAP3;DCAF13;DCAF17;DCAF7;DCTN5;DCUN1D5;DDHD2;DDN;DDX21;DDX31;DDX3X;DDX3Y;DDX41;DDX52;DDX54;DDX6;DECR1;DENND6A;DESI1;DHTKD1;DHX30;DHX35;DHX36;DHX37;DHX38;DHX8;DIABLO;DIAPH1;DICER1;DIEXF;DIXDC1;DKC1;DLC1;DLD;DLGAP3;DMAP1;DMD;DMPK;DMRT2;DMTF1;DNAAF5;DNAJA1;DNAJA2;DNAJA4;DNAJB1;DNAJB4;DNAJC1;DNAJC10;DNAJC15;DNAJC2;DNAJC9;DNTTIP2;DOCK11;DOCK5;DOCK9;DPP8;DSCR3;DSP;DTD1;DUSP14;DYNLL2;DYRK3;E2F3;E2F7;EARS2;ECHDC1;EDC3;EDC4;EEF1A1;EEF1G;EEF2;EEF2K;EFNB2;EFTUD2;EGFR;EGLN2;EIF1;EIF1AX;EIF2A;EIF2B2;EIF2B5;EIF3A;EIF3C;EIF3CL;EIF3E;EIF3F;EIF3H;EIF3K;EIF3M;EIF4B;EIF4E;EIF4G1;EIF4G3;EIF5;EIF5B;ELAC2;ELK4;ELOVL1;ELOVL5;ELP3;EMC6;EML4;EN2;ENPP4;ENTPD1;ENTPD6;ENTPD7;ENY2;EPC1;EPHA2;EPM2AIP1;ERLIN2;ETFRF1;ETNK1;EXD2;EXT1;EZH1;F2;FAM103A1;FAM122A;FAM122B;FAM122C;FAM129B;FAM155B;FAM168A;FAM189B;FAM208A;FAM229B;FAM69A;FAM89A;FAM96B;FASN;FASTKD1;FASTKD2;FAT2;FAT3;FBXL18;FBXL20;FBXO3;FBXO41;FBXW11;FBXW7;FCF1;FDXR;FECH;FEM1C;FGF2;FGFR1;FGFR4;FKBP1A;FLCN;FLNA;FLOT2;FNBP1;FNDC3B;FNTA;FOXK1;FRYL;FSCN2;FTH1;FTL;FUBP1;FURIN;FZD6;FZD9;G3BP2;GABARAP;GABARAPL1;GABPA;GALNT1;GALNT3;GALNT7;GANAB;GARS;GATAD2A;GCC1;GEMIN4;GEMIN5;GFM1;GFPT1;GGA3;GLB1L3;GLP2R;GLRX;GLS2;GLT8D1;GNAL;GNAT1;GNB1;GNB2;GNG12;GNL3L;GOLGA5;GOLGA7;GOLPH3L;GOLT1B;GOSR1;GOT2;GPAA1;GPAM;GPATCH4;GPATCH8;GPR157;GPR180;GPR27;GPR55;GPRC5A;GRAMD2B;GRB2;GRPEL2;GRWD1;GSG1;GSK3B;GSTM4;GSTT2B;GTF2H1;GTF3C1;GTF3C2;GTF3C3;GTF3C4;GTPBP1;GTPBP4;GTPBP8;H2AFX;H3F3B;HACE1;HARS;HARS2;HAUS3;HCFC2;HDGF;HDHD2;HDHD5;HEATR1;HEATR3;HELZ;HERC6;HEYL;HGF;HIGD1A;HIST1H1C;HIST1H2AJ;HIST1H2BC;HIST1H2BK;HIST1H4B;HIST2H2BE;HM13;HMBOX1;HMGA1;HMGA2;HMGN1;HMOX1;HNRNPA1;HNRNPA1L2;HNRNPA2B1;HNRNPDL;HNRNPF;HNRNPL;HOXA10;HOXA3;HOXC8;HOXD13;HPF1;HSD17B8;HSDL2;HSP90AA1;HSP90B1;HSP90B2P;HSPA1A;HSPA1B;HSPA4L;HSPA5;HSPA8;HSPA9;HSPBP1;HSPD1;HSPE1-MOB4;HSPH1;HTRA1;HTT;HYAL3;HYOU1;IARS;IBTK;IDH3A;IDS;IER2;IER3IP1;IFNG;IFRD1;IFRD2;IFT74;IGF1R;IGF2R;IGSF1;IKZF4;IL12B;IL36RN;IMPAD1;IMPDH2;INF2;INO80D;INSL6;INTS5;IPO11;IPO4;IPO5;IPO7;IPPK;IRAK1BP1;IRAK3;IRF4;IRS4;ITGA2;ITGB4;ITPR1;IVNS1ABP;IWS1;JAK2;JARID2;JPT2;JUN;KANK1;KANSL3;KATNAL1;KCNC4;KCND3;KCNG3;KCNN4;KCNS2;KDM2A;KDR;KDSR;KIAA0368;KIAA0895;KIAA1456;KIAA2013;KIDINS220;KIF14;KIF1B;KIF23;KIF2A;KIF2C;KIF3B;KIF5A;KIF5B;KIN;KLC2;KLF14;KLHDC10;KLHL15;KLHL34;KLHL40;KMT2A;KMT2D;KPNA1;KPNA2;KPNA3;KRAS;KRT33B;L1CAM;L2HGDH;LAMB1;LAMB3;LAMC1;LAMP2;LAMTOR1;LAMTOR2;LAMTOR3;LAMTOR4;LAMTOR5;LANCL1;LAPTM4B;LARP1;LARP4;LATS1;LDAH;LIG4;LIMA1;LIN7C;LITAF;LMAN2L;LMF2;LMO7;LMTK3;LONP1;LONRF2;LRIF1;LRIG2;LRPPRC;LRRC57;LRRFIP2;LSG1;LSM10;LSM11;LSM5;LUC7L;LUC7L3;LURAP1L;LUZP1;LY6K;LYAR;LYPD2;LYPLA2;MACF1;MAFK;MAIP1;MALSU1;MAP2K3;MAP3K7;MAP4;MAP4K2;MAP7;MAPKAPK2;MARCH6;MARS;MBD4;MCFD2;MCL1;MCM3AP-AS1;MCU;MDN1;MED11;MED12;MED13;MED24;MEGF8;MEPCE;MERTK;METAP2;METTL13;MFN2;MGAT4A;MIB1;MICU2;MIGA1;MINK1;MIS12;MKI67;MKX;MLF2;MLLT1;MLLT11;MLLT6;MLXIP;MMP25;MMS19;MOB3C;MOB4;MORF4L1;MOV10;MPDU1;MRC2;MRFAP1;MRM3;MRPL1;MRPL10;MRPL12;MRPL20;MRPL21;MRPL3;MRPL40;MRPS10;MRPS12;MRPS14;MRPS2;MRPS23;MRPS25;MRPS31;MRPS35;MRRF;MSANTD4;MSH2;MSL1;MSL3;MT-CO1;MTFR1L;MTHFD1L;MTHFD2;MTHFR;MTMR3;MTMR4;MTOR;MYB;MYC;MYO19;MYO5A;MYO5B;N4BP1;NAA10;NAA15;NAA25;NACA;NAMPT;NAPG;NARF;NARS;NARS2;NAT8L;NAV2;NCAPD2;NCAPG;NCKAP1;NCKAP5L;NCOR2;NCSTN;NDUFA4;NDUFA9;NDUFAF4;NEGR1;NEMF;NEURL4;NFIC;NFKB1;NHLRC3;NIN;NIPAL2;NISCH;NLE1;NMD3;NNT;NOB1;NOC3L;NOC4L;NOL11;NOL4L;NOP10;NOP14;NOP53;NOTCH2;NPR3;NPRL3;NR1I2;NR2C2;NR6A1;NRP1;NRXN1;NSF;NSUN2;NT5C3A;NT5DC1;NT5DC2;NTHL1;NUCKS1;NUDT3;NUFIP2;NUP155;NUP160;NUP50;NUP98;NXPH2;OCRL;ODF2L;OGDH;OGT;OIP5;OMA1;ONECUT2;OPRM1;ORAI2;ORC4;OSBPL3;OSCAR;OSGEPL1;OTOL1;OTUB1;OTUD7B;OTULIN;OXNAD1;PA2G4;PABPC4;PACSIN3;PAFAH1B1;PAFAH1B2;PAG1;PAGR1;PAK1IP1;PAK2;PANK1;PANX1;PAPOLG;PAQR3;PCDHGB4;PCK2;PCMT1;PDCD1;PDCD4;PDCD6IP;PDE3B;PDE4D;PDF;PDHX;PDIA6;PDIK1L;PDK4;PDLIM5;PDPR;PDXK;PELO;PEX12;PEX13;PEX14;PGD;PGLYRP1;PHC3;PHEX;PHF19;PHIP;PHKA1;PHKB;PHLDA2;PHLDB2;PHLPP2;PHYHI

			P;PI4K2B;PIK3R1;PIM1;PIP4K2B;PISD;PLAG1;PLAUR;PLCXD2;PLD3;PLEC;PLEKHA1;PLEKHB2;PLEKHM2;PLEKHN1;PLK1;PLPBP;PLPP3;PLRG1;PLSCR4;PMS1;PNISR;PNN;PNP;PNPLA6;PNPO;PNRC2;POFUT1;POLB;POLDIP2;POLDIP3;POLE4;POLR1C;POLR2A;POLR2E;POLR3A;POM121C;PON2;POTEF;POU2AF1;PPA1;PPAN;PPIF;PPIG;PPIL1;PPIP5K2;PPM1A;PPM1D;PPP1R11;PPP2R1A;PPP2R1B;PPP2R5C;PPP4R3B;PPP6C;PPP6R3;PPT1;PRDM4;PRELID3B;PRICKLE2;PRIM1;PRIMPOL;PRKAA1;PRKAB2;PRKAR1A;PRKAR2A;PRKCD;PRNP;PRPF8;PRPSAP1;PRR12;PRR3;PRRC2C;PRSS21;PSAT1;PSKH1;PSMB5;PSMC1;PSMC2;PSMD11;PSMD12;PSME3;PSME4;PSPH;PTCD3;PTGS2;PTPDC1;PTPN18;PTPN3;PTPRD;PTPRJ;PTPRT;PTRH1;PUM1;PURA;PVR;PWWP2A;PYGB;QSOX2;RAB11FIP2;RAB12;RAB15;RAB1A;RAB1B;RAB21;RAB23;RAB30;RAB3IP;RAB40B;RAB9B;RABGGTB;RACGAP1;RAD23B;RAD51C;RAF1;RALGAPB;RAN;RANGAP1;RAP2C;RAPH1;RARB;RARS;RASEF;RASSF2;RASSF5;RBBP6;RBM15B;RBM28;RBM6;RBMS1;RBMS3;RBPJ;RCAN3;RCC1L;RCL1;RCOR1;RECK;REL;RELT;REXO1;RFK;RFT1;RFWD2;RHOF;RHOT1;RHOV;RIC1;RICTOR;RIDA;RIMS3;RIOK3;RIOX2;RMDN1;RNASEH1P1;RNASEL;RNF111;RNF138;RNF144B;RNF149;RNF168;RNF217;RNF38;RNMT;RNPS1;ROGDI;RP1L1;RPH3AL;RPL10;RPL10L;RPL12;RPL13;RPL14;RPL21;RPL27A;RPL3;RPL30;RPL31;RPL36;RPL4;RPL5;RPL6;RPL9;RPLP0;RPLP1;RPRD1B;RPRD2;RPS17;RPS2;RPS24;RPS25;RPS27;RPS3;RPS3A;RPS5;RPS6;RPS6KA3;RPS6KB1;RPSA;RRP12;RRP15;RRP36;RRP9;RS1;RSL1D1;RTN4;RUBCN;RUNDC3B;RUNX1T1;S100A11P1;SACM1L;SALL1;SARS;SART1;SAV1;SBF1;SBNO1;SCAF4;SCAMP3;SCAMP4;SCAMP5;SDHAF2;SEC11A;SEC24A;SEC24B;SEC61A1;SEC61A2;SEH1L;SELENOI;SEMA4C;SENP6;SEPT2;SERBP1;SERINC5;SERPINB5;SERPINE2;SESTD1;SETD1B;SETD5;SF3A3;SF3B3;SFXN1;SGK3;SGTA;SH3BP4;SHOC2;SIDT2;SIK1;SIPA1L2;SIRT4;SKAP2;SKI;SLC11A2;SLC12A2;SLC16A3;SLC19A1;SLC1A5;SLC25A12;SLC25A22;SLC25A29;SLC25A32;SLC25A38;SLC25A39;SLC25A6;SLC27A4;SLC29A1;SLC2A3;SLC30A1;SLC35A1;SLC35A4;SLC35B2;SLC35B3;SLC35E2B;SLC38A1;SLC38A2;SLC38A5;SLC39A10;SLC39A14;SLC39A9;SLC3A2;SLC4A1AP;SLC4A2;SLC6A4;SLC7A1;SLC7A5;SLC9A1;SLC9A2;SLC9A6;SLCO3A1;SLFN13;SLIRP;SMAD1;SMAD3;SMAD5;SMAD7;SMARCA4;SMDT1;SMPD4;SMURF1;SMURF2;SNCG;SND1;SNRPA1;SNRPB2;SNRPC;SNTB2;SNX11;SNX12;SNX15;SNX16;SNX6;SOCS2;SOCS3;SOCS5;SON;SOWAHC;SOX5;SOX6;SP1;SPATA2;SPATA5;SPCS3;SPEN;SPOUT1;SPRED1;SPRYD3;SPTBN2;SPTLC1;SQSTM1;SREK1;SRP19;SRP68;SRP72;SRPK1;SRPRA;SRPRB;SRSF1;SSRP1;SSSCA1;SSU72;STAU1;STEAP3;STIP1;STK33;STK38;STRADB;STRAP;STT3B;STX17;STX4;STXBP3;SUCLA2;SUN1;SUPT16H;SUPT5H;SYF2;SYNJ1;SYNRG;SYPL1;SYT11;SZRD1;TACO1;TADA2B;TAF13;TAF15;TAF9;TAOK1;TARBP2;TASP1;TBC1D14;TBC1D20;TBCCD1;TBL1XR1;TBL3;TBP;TBPL1;TBRG1;TBRG4;TCF3;TCFL5;TCP1;TDRD3;TECPR2;TELO2;TEP1;TERF2IP;TES;TET3;TEX15;TFAP2A;TFAP4;TFB1M;TFPI;TFRC;TGFBI;TGFBR3;TGOLN2;THAP7;THEM4;THRAP3;TIA1;TIGD3;TIMM10B;TIMM13;TIMM17A;TIMP3;TKTL1;TLE4;TLK1;TLL1;TM4SF1;TM7SF3;TM9SF2;TMBIM6;TMCC1;TMED1;TMED7;TMEM100;TMEM109;TMEM126A;TMEM135;TMEM138;TMEM154;TMEM161B;TMEM168;TMEM189;TMEM189-UBE2V1;TMEM245;TMEM251;TMEM255A;TMEM41A;TMEM43;TMEM69;TMEM87A;TMTC3;TNFAIP2;TNFRSF10A;TNFRSF12A;TNFSF9;TNK1;TNPO1;TNPO3;TNRC6B;TOB2;TOMM34;TOR1A;TOR4A;TOX4;TP53;TPBG;TPI1;TPM2;TPM3;TPPP3;TPT1;TRAF4;TRAK1;TRAM1;TRIM32;TRIM35;TRIM4;TRIM44;TRMT1;TRMT10C;TRMT13;TRUB2;TSC22D2;TSPAN3;TSR1;TTC1;TTC17;TTF2;TTLL12;TTLL5;TUBA1A;TUBA1C;TUBB;TUBB2A;TUBB3;TUBB4B;TUBGCP2;TXLNG;TXN2;TXNIP;TXNL1;U2SURP;UBE2C;UBE2H;UBE2Q1;UBE2Q2;UBE2S;UBE2V1;UBE2Z;UBE3C;UBE4A;UBFD1;UBN2;UBR3;UBXN2B;UCA1;UFC1;UFSP2;UGDH;UGP2;UGT2B4;UGT8;ULK1;UNG;UQCC3;USMG5;USP15;USP3;USP31;USP42;USP48;USP53;USP7;USP8;USP9X;UTP14A;UTP15;UTP20;UTP23;UTP3;UTP4;UXT;VAMP8;VASN;VAV2;VCL;VEGFA;VEZT;VIM;VKORC1;VMA21;VMP1;VOPP1;VPS33B;VPS45;VPS4A;VSIR;VTI1B;WARS;WBP11;WDR13;WDR18;WDR3;WDR43;WDR5B;WDR75;WEE1;WIPF1;WIPI2;WNK3;WNT3A;WNT4;WNT5A;WT1;XKR7;XKR8;XPNPEP3;XPO1;XPO4;XPO6;XPO7;XPOT;XYLT1;YAP1;YARS2;YBX1;YBX3;YIF1B;YIPF2;YIPF4;YIPF6;YRDC;YTHDC1;YWHAH;YWHAQ;YY1AP1;ZBTB10;ZBTB16;ZBTB2;ZBTB33;ZBTB34;ZBTB5;ZC3H11A;ZCCHC3;ZDHHC16;ZEB2;ZFHX3;ZFHX4;ZFP28;ZFPL1;ZMAT3;ZNF207;ZNF267;ZNF275;ZNF280C;ZNF284;ZNF367;ZNF384;ZNF391;ZNF449;ZNF460;ZNF559;ZNF585B;ZNF598;ZNF620;ZNF622;ZNF638;ZNF644;ZNF691;ZNF704;ZNF791;ZNF827;ZNF91;ZNRF1;ZNRF2;ZNRF3;ZRANB1;ZRANB2;ZYX;ZZEF1
hsa-miR-16-5p-477860_mir	-0.531	0.245	AAAS;AADAT;ABCB7;ABCC1;ABCC4;ABCC6;ABCF1;ABCF2;ABHD10;ABHD2;ABL2;ACBD5;ACOT8;ACOX1;ACP2;ACTB;ACTG1;ACTN1;ACTN4;ACTR1A;ACTR2;ACTR3B;ACVR2A;ADAD2;ADGRE5;ADGRL1;ADK;ADORA2A;ADORA3;ADRA2B;ADSS;AFF4;AFG3L2;AGER;AGK;AGO2;AGO4;AGPAT5;AGRN;AHCYL1;AHCYL2;AHNAK2;AIFM2;AIMP1;AK2;AKAP11;AKAP13;AKR1B10;AKT3;ALDH18A1;ALDH2;ALDH3B1;ALG2;ALG3;ALKAL2;AMER1;AMOT;AMOTL1;AMPD1;ANAPC13;ANAPC16;ANKLE2;ANKMY1;ANKRD13B;ANKRD17;ANKRD36;ANPEP;AP2A1;AP2B1;AP2M1;AP3D1;AP3M1;AP5Z1;APLNR;APP;AQP12B;ARCN1;ARG2;ARHGAP12;ARHGAP32;ARHGDIA;ARIH1;ARL10;ARL2;ARL2BP;ARL3;ARMC12;ARMCX2;ARPC5L;ASB6;ASCC1;ASCC3;ASGR2;ASH1L;ASNS;ASPH;ASXL1;ASXL2;ATAD5;ATF7;ATG14;ATG9A;

			ATL2;ATOX1;ATP13A3;ATP5A1;ATP5C1;ATP5G3;ATP6V0E1;ATP6V1B2;ATP6V1E1;ATP8A2;ATXN2L;ATXN7L3;ATXN7L3B;AUP1;AURKAIP1;AURKB;AVL9;AXIN2;B3GNT2;B4GALT1;BACE1;BAG4;BAG6;BAMBI;BAZ1B;BAZ2A;BCAS2;BCCIP;BCL11B;BCL2;BCL2L12;BCL7A;BDNF;BEX3;BFAR;BHLHE40;BIRC5;BMI1;BMS1;BNC2;BRAT1;BRCA1;BSG;BSPRY;BTAF1;BTBD2;BTF3;BTG2;BTN3A3;BTRC;BYSL;BZW1;C11ORF24;C15ORF39;C16ORF58;C16ORF72;C17ORF75;C17ORF80;C19ORF54;C1ORF21;C1ORF226;C1ORF56;C21ORF62;C2ORF42;C2ORF74;C3ORF36;C6ORF106;C8ORF44;CA12;CA8;CAAP1;CABIN1;CACNA2D1;CACNB2;CACUL1;CADM1;CALR;CALU;CAMK2G;CAMKK2;CAMKV;CAMSAP1;CAMSAP3;CANX;CAPRIN1;CAPZA2;CARD10;CARD19;CARD8;CARM1;CASK;CASKIN1;CBFA2T3;CBX2;CBX4;CBX6;CCDC58;CCDC59;CCDC80;CCDC83;CCDC88C;CCND1;CCND2;CCND3;CCNE1;CCNE2;CCNJ;CCNT1;CCNT2;CCPG1;CCT3;CCT6B;CCT8;CD180;CD274;CD2AP;CD44;CD55;CDADC1;CDC123;CDC14B;CDC20;CDC23;CDC25A;CDC27;CDC37;CDC37L1;CDC42SE2;CDC5L;CDCA4;CDCA8;CDIPT;CDK1;CDK17;CDK5RAP1;CDK6;CDK9;CDKN1A;CDKN2A;CDKN2AIPNL;CDS2;CDV3;CENPF;CENPJ;CEP55;CEP63;CFAP45;CFL2;CHAC1;CHD3;CHEK1;CHERP;CHIC1;CHMP3;CHMP4B;CHORDC1;CHPT1;CHUK;CIB1;CKAP5;CLASP1;CLDN2;CLEC2D;CLIP2;CLIP4;CLNS1A;CLSPN;CLTC;CLU;CLUH;CMPK1;CMTM4;CNKSR3;CNN3;CNNM2;CNOT7;CNP;CNPY3;COA6;COA7;COL4A1;COL4A2;COMMD10;COMT;COPS5;COPS7B;COQ3;CPEB2;CPEB3;CPNE1;CPNE8;CPSF7;CPT1A;CREB3L2;CREBL2;CREBRF;CREG1;CRHBP;CRIM1;CRK;CRKL;CSDE1;CSGALNACT1;CSHL1;CSNK1E;CTDSPL;CTSD;CUL2;CUL3;CUL4A;CXORF38;CXORF40B;CYB561A3;CYB5R1;CYCS;CYLD;CYP26B1;CYP27B1;DAP3;DCAF13;DCAF17;DCAF7;DCTN5;DCUN1D5;DDHD2;DDN;DDX21;DDX31;DDX3X;DDX3Y;DDX41;DDX52;DDX54;DDX6;DECR1;DENND6A;DESI1;DHTKD1;DHX30;DHX35;DHX36;DHX37;DHX38;DHX8;DIABLO;DIAPH1;DICER1;DIEXF;DIXDC1;DKC1;DLC1;DLD;DLGAP3;DMAP1;DMD;DMPK;DMRT2;DMTF1;DNAAF5;DNAJA1;DNAJA2;DNAJA4;DNAJB1;DNAJB4;DNAJC1;DNAJC10;DNAJC15;DNAJC2;DNAJC9;DNTTIP2;DOCK11;DOCK5;DOCK9;DPP8;DSCR3;DSP;DTD1;DUSP14;DYNLL2;DYRK3;E2F3;E2F7;EARS2;ECHDC1;EDC3;EDC4;EEF1A1;EEF1G;EEF2;EEF2K;EFNB2;EFTUD2;EGFR;EGLN2;EIF1;EIF1AX;EIF2A;EIF2B2;EIF2B5;EIF3A;EIF3C;EIF3CL;EIF3E;EIF3F;EIF3H;EIF3K;EIF3M;EIF4B;EIF4E;EIF4G1;EIF4G3;EIF5;EIF5B;ELAC2;ELK4;ELOVL1;ELOVL5;ELP3;EMC6;EML4;EN2;ENPP4;ENTPD1;ENTPD6;ENTPD7;ENY2;EPC1;EPHA2;EPM2AIP1;ERLIN2;ETFRF1;ETNK1;EXD2;EXT1;EZH1;F2;FAM103A1;FAM122A;FAM122B;FAM122C;FAM129B;FAM155B;FAM168A;FAM189B;FAM208A;FAM229B;FAM69A;FAM89A;FAM96B;FASN;FASTKD1;FASTKD2;FAT2;FAT3;FBXL18;FBXL20;FBXO3;FBXO41;FBXW11;FBXW7;FCF1;FDXR;FECH;FEM1C;FGF2;FGFR1;FGFR4;FKBP1A;FLCN;FLNA;FLOT2;FNBP1;FNDC3B;FNTA;FOXK1;FRYL;FSCN2;FTH1;FTL;FUBP1;FURIN;FZD6;FZD9;G3BP2;GABARAP;GABARAPL1;GABPA;GALNT1;GALNT3;GALNT7;GANAB;GARS;GATAD2A;GCC1;GEMIN4;GEMIN5;GFM1;GFPT1;GGA3;GLB1L3;GLP2R;GLRX;GLS2;GLT8D1;GNAL;GNAT1;GNB1;GNB2;GNG12;GNL3L;GOLGA5;GOLGA7;GOLPH3L;GOLT1B;GOSR1;GOT2;GPAA1;GPAM;GPATCH4;GPATCH8;GPR157;GPR180;GPR27;GPR55;GPRC5A;GRAMD2B;GRB2;GRPEL2;GRWD1;GSG1;GSK3B;GSTM4;GSTT2B;GTF2H1;GTF3C1;GTF3C2;GTF3C3;GTF3C4;GTPBP1;GTPBP4;GTPBP8;H2AFX;H3F3B;HACE1;HARS;HARS2;HAUS3;HCFC2;HDGF;HDHD2;HDHD5;HEATR1;HEATR3;HELZ;HERC6;HEYL;HGF;HIGD1A;HIST1H1C;HIST1H2AJ;HIST1H2BC;HIST1H2BK;HIST1H4B;HIST2H2BE;HM13;HMBOX1;HMGA1;HMGA2;HMGN1;HMOX1;HNRNPA1;HNRNPA1L2;HNRNPA2B1;HNRNPDL;HNRNPF;HNRNPL;HOXA10;HOXA3;HOXC8;HOXD13;HPF1;HSD17B8;HSDL2;HSP90AA1;HSP90B1;HSP90B2P;HSPA1A;HSPA1B;HSPA4L;HSPA5;HSPA8;HSPA9;HSPBP1;HSPD1;HSPE1-MOB4;HSPH1;HTRA1;HTT;HYAL3;HYOU1;IARS;IBTK;IDH3A;IDS;IER2;IER3IP1;IFNG;IFRD1;IFRD2;IFT74;IGF1R;IGF2R;IGSF1;IKZF4;IL12B;IL36RN;IMPAD1;IMPDH2;INF2;INO80D;INSL6;INTS5;IPO11;IPO4;IPO5;IPO7;IPPK;IRAK1BP1;IRAK3;IRF4;IRS4;ITGA2;ITGB4;ITPR1;IVNS1ABP;IWS1;JAK2;JARID2;JPT2;JUN;KANK1;KANSL3;KATNAL1;KCNC4;KCND3;KCNG3;KCNN4;KCNS2;KDM2A;KDR;KDSR;KIAA0368;KIAA0895;KIAA1456;KIAA2013;KIDINS220;KIF14;KIF1B;KIF23;KIF2A;KIF2C;KIF3B;KIF5A;KIF5B;KIN;KLC2;KLF14;KLHDC10;KLHL15;KLHL34;KLHL40;KMT2A;KMT2D;KPNA1;KPNA2;KPNA3;KRAS;KRT33B;L1CAM;L2HGDH;LAMB1;LAMB3;LAMC1;LAMP2;LAMTOR1;LAMTOR2;LAMTOR3;LAMTOR4;LAMTOR5;LANCL1;LAPTM4B;LARP1;LARP4;LATS1;LDAH;LIG4;LIMA1;LIN7C;LITAF;LMAN2L;LMF2;LMO7;LMTK3;LONP1;LONRF2;LRIF1;LRIG2;LRPPRC;LRRC57;LRRFIP2;LSG1;LSM10;LSM11;LSM5;LUC7L;LUC7L3;LURAP1L;LUZP1;LY6K;LYAR;LYPD2;LYPLA2;MACF1;MAFK;MAIP1;MALSU1;MAP2K3;MAP3K7;MAP4;MAP4K2;MAP7;MAPKAPK2;MARCH6;MARS;MBD4;MCFD2;MCL1;MCM3AP-AS1;MCU;MDN1;MED11;MED12;MED13;MED24;MEGF8;MEPCE;MERTK;METAP2;METTL13;MFN2;MGAT4A;MIB1;MICU2;MIGA1;MINK1;MIS12;MKI67;MKX;MLF2;MLLT1;MLLT11;MLLT6;MLXIP;MMP25;MMS19;MOB3C;MOB4;MORF4L1;MOV10;MPDU1;MRC2;MRFAP1;MRM3;MRPL1;MRPL10;MRPL12;MRPL20;MRPL21;MRPL3;MRPL40;MRPS10;MRPS12;MRPS14;MRPS2;MRPS23;MRPS25;MRPS31;MRPS35;MRRF;MSANTD4;MSH2;MSL1;MSL3;MT-CO1;MTFR1L;MTHFD1L;MTHFD2;MTHFR;MTMR3;MTMR4;MTOR;MYB;MYC;MY

			O19;MYO5A;MYO5B;N4BP1;NAA10;NAA15;NAA25;NACA;NAMPT;NAPG;NARF;NARS;NARS2;NAT8L;NAV2;NCAPD2;NCAPG;NCKAP1;NCKAP5L;NCOR2;NCSTN;NDUFA4;NDUFA9;NDUFAF4;NEGR1;NEMF;NEURL4;NFIC;NFKB1;NHLRC3;NIN;NIPAL2;NISCH;NLE1;NMD3;NNT;NOB1;NOC3L;NOC4L;NOL11;NOL4L;NOP10;NOP14;NOP53;NOTCH2;NPR3;NPRL3;NR1I2;NR2C2;NR6A1;NRP1;NRXN1;NSF;NSUN2;NT5C3A;NT5DC1;NT5DC2;NTHL1;NUCKS1;NUDT3;NUFIP2;NUP155;NUP160;NUP50;NUP98;NXPH2;OCRL;ODF2L;OGDH;OGT;OIP5;OMA1;ONECUT2;OPRM1;ORAI2;ORC4;OSBPL3;OSCAR;OSGEPL1;OTOL1;OTUB1;OTUD7B;OTULIN;OXNAD1;PA2G4;PABPC4;PACSIN3;PAFAH1B1;PAFAH1B2;PAG1;PAGR1;PAK1IP1;PAK2;PANK1;PANX1;PAPOLG;PAQR3;PCDHGB4;PCK2;PCMT1;PDCD1;PDCD4;PDCD6IP;PDE3B;PDE4D;PDF;PDHX;PDIA6;PDIK1L;PDK4;PDLIM5;PDPR;PDXK;PELO;PEX12;PEX13;PEX14;PGD;PGLYRP1;PHC3;PHEX;PHF19;PHIP;PHKA1;PHKB;PHLDA2;PHLDB2;PHLPP2;PHYHIP;PI4K2B;PIK3R1;PIM1;PIP4K2B;PISD;PLAG1;PLAUR;PLCXD2;PLD3;PLEC;PLEKHA1;PLEKHB2;PLEKHM2;PLEKHN1;PLK1;PLPBP;PLPP3;PLRG1;PLSCR4;PMS1;PNISR;PNN;PNP;PNPLA6;PNPO;PNRC2;POFUT1;POLB;POLDIP2;POLDIP3;POLE4;POLR1C;POLR2A;POLR2E;POLR3A;POM121C;PON2;POTEF;POU2AF1;PPA1;PPAN;PPIF;PPIG;PPIL1;PPIP5K2;PPM1A;PPM1D;PPP1R11;PPP2R1A;PPP2R1B;PPP2R5C;PPP4R3B;PPP6C;PPP6R3;PPT1;PRDM4;PRELID3B;PRICKLE2;PRIM1;PRIMPOL;PRKAA1;PRKAB2;PRKAR1A;PRKAR2A;PRKCD;PRNP;PRPF8;PRPSAP1;PRR12;PRR3;PRRC2C;PRSS21;PSAT1;PSKH1;PSMB5;PSMC1;PSMC2;PSMD11;PSMD12;PSME3;PSME4;PSPH;PTCD3;PTGS2;PTPDC1;PTPN18;PTPN3;PTPRD;PTPRJ;PTPRT;PTRH1;PUM1;PURA;PVR;PWWP2A;PYGB;QSOX2;RAB11FIP2;RAB12;RAB15;RAB1A;RAB1B;RAB21;RAB23;RAB30;RAB3IP;RAB40B;RAB9B;RABGGTB;RACGAP1;RAD23B;RAD51C;RAF1;RALGAPB;RAN;RANGAP1;RAP2C;RAPH1;RARB;RARS;RASEF;RASSF2;RASSF5;RBBP6;RBM15B;RBM28;RBM6;RBMS1;RBMS3;RBPJ;RCAN3;RCC1L;RCL1;RCOR1;RECK;REL;RELT;REXO1;RFK;RFT1;RFWD2;RHOF;RHOT1;RHOV;RIC1;RICTOR;RIDA;RIMS3;RIOK3;RIOX2;RMDN1;RNASEH1P1;RNASEL;RNF111;RNF138;RNF144B;RNF149;RNF168;RNF217;RNF38;RNMT;RNPS1;ROGDI;RP1L1;RPH3AL;RPL10;RPL10L;RPL12;RPL13;RPL14;RPL21;RPL27A;RPL3;RPL30;RPL31;RPL36;RPL4;RPL5;RPL6;RPL9;RPLP0;RPLP1;RPRD1B;RPRD2;RPS17;RPS2;RPS24;RPS25;RPS27;RPS3;RPS3A;RPS5;RPS6;RPS6KA3;RPS6KB1;RPSA;RRP12;RRP15;RRP36;RRP9;RS1;RSL1D1;RTN4;RUBCN;RUNDC3B;RUNX1T1;S100A11P1;SACM1L;SALL1;SARS;SART1;SAV1;SBF1;SBNO1;SCAF4;SCAMP3;SCAMP4;SCAMP5;SDHAF2;SEC11A;SEC24A;SEC24B;SEC61A1;SEC61A2;SEH1L;SELENOI;SEMA4C;SENP6;SEPT2;SERBP1;SERINC5;SERPINB5;SERPINE2;SESTD1;SETD1B;SETD5;SF3A3;SF3B3;SFXN1;SGK3;SGTA;SH3BP4;SHOC2;SIDT2;SIK1;SIPA1L2;SIRT4;SKAP2;SKI;SLC11A2;SLC12A2;SLC16A3;SLC19A1;SLC1A5;SLC25A12;SLC25A22;SLC25A29;SLC25A32;SLC25A38;SLC25A39;SLC25A6;SLC27A4;SLC29A1;SLC2A3;SLC30A1;SLC35A1;SLC35A4;SLC35B2;SLC35B3;SLC35E2B;SLC38A1;SLC38A2;SLC38A5;SLC39A10;SLC39A14;SLC39A9;SLC3A2;SLC4A1AP;SLC4A2;SLC6A4;SLC7A1;SLC7A5;SLC9A1;SLC9A2;SLC9A6;SLCO3A1;SLFN13;SLIRP;SMAD1;SMAD3;SMAD5;SMAD7;SMARCA4;SMDT1;SMPD4;SMURF1;SMURF2;SNCG;SND1;SNRPA1;SNRPB2;SNRPC;SNTB2;SNX11;SNX12;SNX15;SNX16;SNX6;SOCS2;SOCS3;SOCS5;SON;SOWAHC;SOX5;SOX6;SP1;SPATA2;SPATA5;SPCS3;SPEN;SPOUT1;SPRED1;SPRYD3;SPTBN2;SPTLC1;SQSTM1;SREK1;SRP19;SRP68;SRP72;SRPK1;SRPRA;SRPRB;SRSF1;SSRP1;SSSCA1;SSU72;STAU1;STEAP3;STIP1;STK33;STK38;STRADB;STRAP;STT3B;STX17;STX4;STXBP3;SUCLA2;SUN1;SUPT16H;SUPT5H;SYF2;SYNJ1;SYNRG;SYPL1;SYT11;SZRD1;TACO1;TADA2B;TAF13;TAF15;TAF9;TAOK1;TARBP2;TASP1;TBC1D14;TBC1D20;TBCCD1;TBL1XR1;TBL3;TBP;TBPL1;TBRG1;TBRG4;TCF3;TCFL5;TCP1;TDRD3;TECPR2;TELO2;TEP1;TERF2IP;TES;TET3;TEX15;TFAP2A;TFAP4;TFB1M;TFPI;TFRC;TGFBI;TGFBR3;TGOLN2;THAP7;THEM4;THRAP3;TIA1;TIGD3;TIMM10B;TIMM13;TIMM17A;TIMP3;TKTL1;TLE4;TLK1;TLL1;TM4SF1;TM7SF3;TM9SF2;TMBIM6;TMCC1;TMED1;TMED7;TMEM100;TMEM109;TMEM126A;TMEM135;TMEM138;TMEM154;TMEM161B;TMEM168;TMEM189;TMEM189-UBE2V1;TMEM245;TMEM251;TMEM255A;TMEM41A;TMEM43;TMEM69;TMEM87A;TMTC3;TNFAIP2;TNFRSF10A;TNFRSF12A;TNFSF9;TNK1;TNPO1;TNPO3;TNRC6B;TOB2;TOMM34;TOR1A;TOR4A;TOX4;TP53;TPBG;TPI1;TPM2;TPM3;TPPP3;TPT1;TRAF4;TRAK1;TRAM1;TRIM32;TRIM35;TRIM4;TRIM44;TRMT1;TRMT10C;TRMT13;TRUB2;TSC22D2;TSPAN3;TSR1;TTC1;TTC17;TTF2;TTLL12;TTLL5;TUBA1A;TUBA1C;TUBB;TUBB2A;TUBB3;TUBB4B;TUBGCP2;TXLNG;TXN2;TXNIP;TXNL1;U2SURP;UBE2C;UBE2H;UBE2Q1;UBE2Q2;UBE2S;UBE2V1;UBE2Z;UBE3C;UBE4A;UBFD1;UBN2;UBR3;UBXN2B;UCA1;UFC1;UFSP2;UGDH;UGP2;UGT2B4;UGT8;ULK1;UNG;UQCC3;USMG5;USP15;USP3;USP31;USP42;USP48;USP53;USP7;USP8;USP9X;UTP14A;UTP15;UTP20;UTP23;UTP3;UTP4;UXT;VAMP8;VASN;VAV2;VCL;VEGFA;VEZT;VIM;VKORC1;VMA21;VMP1;VOPP1;VPS33B;VPS45;VPS4A;VSIR;VTI1B;WARS;WBP11;WDR13;WDR18;WDR3;WDR43;WDR5B;WDR75;WEE1;WIPF1;WIPI2;WNK3;WNT3A;WNT4;WNT5A;WT1;XKR7;XKR8;XPNPEP3;XPO1;XPO4;XPO6;XPO7;XPOT;XYLT1;YAP1;YARS2;YBX1;YBX3;YIF1B;YIPF2;YIPF4;YIPF6;YRDC;YTHDC1;YWHAH;YWHAQ;YY1AP1;ZBTB10;ZBTB16;ZBTB2;ZBTB33;ZBTB34;ZBTB5;ZC3H11A;ZCCHC3;ZDHHC16;ZEB2;ZFHX3;ZFHX4;ZFP28;ZFPL1;ZMAT3;ZNF207;ZNF267;ZNF275;ZNF280C;ZNF284;ZNF367;ZNF384;ZNF391;ZNF449;ZNF460;ZNF559;ZNF585B;ZNF598;Z

			NF620;ZNF622;ZNF638;ZNF644;ZNF691;ZNF704;ZNF791;ZNF827;ZNF91;ZNRF1;ZNRF2;ZNRF3;ZRANB1;ZRANB2;ZYX;ZZEF1
hsa-miR-16-5p-477860_mir	-0.531	0.245	AAAS;AADAT;ABCB7;ABCC1;ABCC4;ABCC6;ABCF1;ABCF2;ABHD10;ABHD2;ABL2;ACBD5;ACOT8;ACOX1;ACP2;ACTB;ACTG1;ACTN1;ACTN4;ACTR1A;ACTR2;ACTR3B;ACVR2A;ADAD2;ADGRE5;ADGRL1;ADK;ADORA2A;ADORA3;ADRA2B;ADSS;AFF4;AFG3L2;AGER;AGK;AGO2;AGO4;AGPAT5;AGRN;AHCYL1;AHCYL2;AHNAK2;AIFM2;AIMP1;AK2;AKAP11;AKAP13;AKR1B10;AKT3;ALDH18A1;ALDH2;ALDH3B1;ALG2;ALG3;ALKAL2;AMER1;AMOT;AMOTL1;AMPD1;ANAPC13;ANAPC16;ANKLE2;ANKMY1;ANKRD13B;ANKRD17;ANKRD36;ANPEP;AP2A1;AP2B1;AP2M1;AP3D1;AP3M1;AP5Z1;APLNR;APP;AQP12B;ARCN1;ARG2;ARHGAP12;ARHGAP32;ARHGDIA;ARIH1;ARL10;ARL2;ARL2BP;ARL3;ARMC12;ARMCX2;ARPC5L;ASB6;ASCC1;ASCC3;ASGR2;ASH1L;ASNS;ASPH;ASXL1;ASXL2;ATAD5;ATF7;ATG14;ATG9A;ATL2;ATOX1;ATP13A3;ATP5A1;ATP5C1;ATP5G3;ATP6V0E1;ATP6V1B2;ATP6V1E1;ATP8A2;ATXN2L;ATXN7L3;ATXN7L3B;AUP1;AURKAIP1;AURKB;AVL9;AXIN2;B3GNT2;B4GALT1;BACE1;BAG4;BAG6;BAMBI;BAZ1B;BAZ2A;BCAS2;BCCIP;BCL11B;BCL2;BCL2L12;BCL7A;BDNF;BEX3;BFAR;BHLHE40;BIRC5;BMI1;BMS1;BNC2;BRAT1;BRCA1;BSG;BSPRY;BTAF1;BTBD2;BTF3;BTG2;BTN3A3;BTRC;BYSL;BZW1;C11ORF24;C15ORF39;C16ORF58;C16ORF72;C17ORF75;C17ORF80;C19ORF54;C1ORF21;C1ORF226;C1ORF56;C21ORF62;C2ORF42;C2ORF74;C3ORF36;C6ORF106;C8ORF44;CA12;CA8;CAAP1;CABIN1;CACNA2D1;CACNB2;CACUL1;CADM1;CALR;CALU;CAMK2G;CAMKK2;CAMKV;CAMSAP1;CAMSAP3;CANX;CAPRIN1;CAPZA2;CARD10;CARD19;CARD8;CARM1;CASK;CASKIN1;CBFA2T3;CBX2;CBX4;CBX6;CCDC58;CCDC59;CCDC80;CCDC83;CCDC88C;CCND1;CCND2;CCND3;CCNE1;CCNE2;CCNJ;CCNT1;CCNT2;CCPG1;CCT3;CCT6B;CCT8;CD180;CD274;CD2AP;CD44;CD55;CDADC1;CDC123;CDC14B;CDC20;CDC23;CDC25A;CDC27;CDC37;CDC37L1;CDC42SE2;CDC5L;CDCA4;CDCA8;CDIPT;CDK1;CDK17;CDK5RAP1;CDK6;CDK9;CDKN1A;CDKN2A;CDKN2AIPNL;CDS2;CDV3;CENPF;CENPJ;CEP55;CEP63;CFAP45;CFL2;CHAC1;CHD3;CHEK1;CHERP;CHIC1;CHMP3;CHMP4B;CHORDC1;CHPT1;CHUK;CIB1;CKAP5;CLASP1;CLDN2;CLEC2D;CLIP2;CLIP4;CLNS1A;CLSPN;CLTC;CLU;CLUH;CMPK1;CMTM4;CNKSR3;CNN3;CNNM2;CNOT7;CNP;CNPY3;COA6;COA7;COL4A1;COL4A2;COMMD10;COMT;COPS5;COPS7B;COQ3;CPEB2;CPEB3;CPNE1;CPNE8;CPSF7;CPT1A;CREB3L2;CREBL2;CREBRF;CREG1;CRHBP;CRIM1;CRK;CRKL;CSDE1;CSGALNACT1;CSHL1;CSNK1E;CTDSPL;CTSD;CUL2;CUL3;CUL4A;CXORF38;CXORF40B;CYB561A3;CYB5R1;CYCS;CYLD;CYP26B1;CYP27B1;DAP3;DCAF13;DCAF17;DCAF7;DCTN5;DCUN1D5;DDHD2;DDN;DDX21;DDX31;DDX3X;DDX3Y;DDX41;DDX52;DDX54;DDX6;DECR1;DENND6A;DESI1;DHTKD1;DHX30;DHX35;DHX36;DHX37;DHX38;DHX8;DIABLO;DIAPH1;DICER1;DIEXF;DIXDC1;DKC1;DLC1;DLD;DLGAP3;DMAP1;DMD;DMPK;DMRT2;DMTF1;DNAAF5;DNAJA1;DNAJA2;DNAJA4;DNAJB1;DNAJB4;DNAJC1;DNAJC10;DNAJC15;DNAJC2;DNAJC9;DNTTIP2;DOCK11;DOCK5;DOCK9;DPP8;DSCR3;DSP;DTD1;DUSP14;DYNLL2;DYRK3;E2F3;E2F7;EARS2;ECHDC1;EDC3;EDC4;EEF1A1;EEF1G;EEF2;EEF2K;EFNB2;EFTUD2;EGFR;EGLN2;EIF1;EIF1AX;EIF2A;EIF2B2;EIF2B5;EIF3A;EIF3C;EIF3CL;EIF3E;EIF3F;EIF3H;EIF3K;EIF3M;EIF4B;EIF4E;EIF4G1;EIF4G3;EIF5;EIF5B;ELAC2;ELK4;ELOVL1;ELOVL5;ELP3;EMC6;EML4;EN2;ENPP4;ENTPD1;ENTPD6;ENTPD7;ENY2;EPC1;EPHA2;EPM2AIP1;ERLIN2;ETFRF1;ETNK1;EXD2;EXT1;EZH1;F2;FAM103A1;FAM122A;FAM122B;FAM122C;FAM129B;FAM155B;FAM168A;FAM189B;FAM208A;FAM229B;FAM69A;FAM89A;FAM96B;FASN;FASTKD1;FASTKD2;FAT2;FAT3;FBXL18;FBXL20;FBXO3;FBXO41;FBXW11;FBXW7;FCF1;FDXR;FECH;FEM1C;FGF2;FGFR1;FGFR4;FKBP1A;FLCN;FLNA;FLOT2;FNBP1;FNDC3B;FNTA;FOXK1;FRYL;FSCN2;FTH1;FTL;FUBP1;FURIN;FZD6;FZD9;G3BP2;GABARAP;GABARAPL1;GABPA;GALNT1;GALNT3;GALNT7;GANAB;GARS;GATAD2A;GCC1;GEMIN4;GEMIN5;GFM1;GFPT1;GGA3;GLB1L3;GLP2R;GLRX;GLS2;GLT8D1;GNAL;GNAT1;GNB1;GNB2;GNG12;GNL3L;GOLGA5;GOLGA7;GOLPH3L;GOLT1B;GOSR1;GOT2;GPAA1;GPAM;GPATCH4;GPATCH8;GPR157;GPR180;GPR27;GPR55;GPRC5A;GRAMD2B;GRB2;GRPEL2;GRWD1;GSG1;GSK3B;GSTM4;GSTT2B;GTF2H1;GTF3C1;GTF3C2;GTF3C3;GTF3C4;GTPBP1;GTPBP4;GTPBP8;H2AFX;H3F3B;HACE1;HARS;HARS2;HAUS3;HCFC2;HDGF;HDHD2;HDHD5;HEATR1;HEATR3;HELZ;HERC6;HEYL;HGF;HIGD1A;HIST1H1C;HIST1H2AJ;HIST1H2BC;HIST1H2BK;HIST1H4B;HIST2H2BE;HM13;HMBOX1;HMGA1;HMGA2;HMGN1;HMOX1;HNRNPA1;HNRNPA1L2;HNRNPA2B1;HNRNPDL;HNRNPF;HNRNPL;HOXA10;HOXA3;HOXC8;HOXD13;HPF1;HSD17B8;HSDL2;HSP90AA1;HSP90B1;HSP90B2P;HSPA1A;HSPA1B;HSPA4L;HSPA5;HSPA8;HSPA9;HSPBP1;HSPD1;HSPE1-MOB4;HSPH1;HTRA1;HTT;HYAL3;HYOU1;IARS;IBTK;IDH3A;IDS;IER2;IER3IP1;IFNG;IFRD1;IFRD2;IFT74;IGF1R;IGF2R;IGSF1;IKZF4;IL12B;IL36RN;IMPAD1;IMPDH2;INF2;INO80D;INSL6;INTS5;IPO11;IPO4;IPO5;IPO7;IPPK;IRAK1BP1;IRAK3;IRF4;IRS4;ITGA2;ITGB4;ITPR1;IVNS1ABP;IWS1;JAK2;JARID2;JPT2;JUN;KANK1;KANSL3;KATNAL1;KCNC4;KCND3;KCNG3;KCNN4;KCNS2;KDM2A;KDR;KDSR;KIAA0368;KIAA0895;KIAA1456;KIAA2013;KIDINS220;KIF14;KIF1B;KIF23;KIF2A;KIF2C;KIF3B;KIF5A;KIF5B;KIN;KLC2;KLF14;KLHDC10;KLHL15;KLHL34;KLHL40;KMT2A;KMT2D;KPNA1;KPNA2;KPNA3;KRAS;KRT33B;L1CAM;L2HGDH;LAMB1;LAMB3;LAMC1;LAMP2;LAMTOR1;LAMTOR2;LAMTOR3;LAMTOR4;LAMTOR5;LANCL1;LAPTM4B;L

			ARP1;LARP4;LATS1;LDAH;LIG4;LIMA1;LIN7C;LITAF;LMAN2L;LMF2;LMO7;LMTK3;LONP1;LONRF2;LRIF1;LRIG2;LRPPRC;LRRC57;LRRFIP2;LSG1;LSM10;LSM11;LSM5;LUC7L;LUC7L3;LURAP1L;LUZP1;LY6K;LYAR;LYPD2;LYPLA2;MACF1;MAFK;MAIP1;MALSU1;MAP2K3;MAP3K7;MAP4;MAP4K2;MAP7;MAPKAPK2;MARCH6;MARS;MBD4;MCFD2;MCL1;MCM3AP-AS1;MCU;MDN1;MED11;MED12;MED13;MED24;MEGF8;MEPCE;MERTK;METAP2;METTL13;MFN2;MGAT4A;MIB1;MICU2;MIGA1;MINK1;MIS12;MKI67;MKX;MLF2;MLLT1;MLLT11;MLLT6;MLXIP;MMP25;MMS19;MOB3C;MOB4;MORF4L1;MOV10;MPDU1;MRC2;MRFAP1;MRM3;MRPL1;MRPL10;MRPL12;MRPL20;MRPL21;MRPL3;MRPL40;MRPS10;MRPS12;MRPS14;MRPS2;MRPS23;MRPS25;MRPS31;MRPS35;MRRF;MSANTD4;MSH2;MSL1;MSL3;MT-CO1;MTFR1L;MTHFD1L;MTHFD2;MTHFR;MTMR3;MTMR4;MTOR;MYB;MYC;MYO19;MYO5A;MYO5B;N4BP1;NAA10;NAA15;NAA25;NACA;NAMPT;NAPG;NARF;NARS;NARS2;NAT8L;NAV2;NCAPD2;NCAPG;NCKAP1;NCKAP5L;NCOR2;NCSTN;NDUFA4;NDUFA9;NDUFAF4;NEGR1;NEMF;NEURL4;NFIC;NFKB1;NHLRC3;NIN;NIPAL2;NISCH;NLE1;NMD3;NNT;NOB1;NOC3L;NOC4L;NOL11;NOL4L;NOP10;NOP14;NOP53;NOTCH2;NPR3;NPRL3;NR1I2;NR2C2;NR6A1;NRP1;NRXN1;NSF;NSUN2;NT5C3A;NT5DC1;NT5DC2;NTHL1;NUCKS1;NUDT3;NUFIP2;NUP155;NUP160;NUP50;NUP98;NXPH2;OCRL;ODF2L;OGDH;OGT;OIP5;OMA1;ONECUT2;OPRM1;ORAI2;ORC4;OSBPL3;OSCAR;OSGEPL1;OTOL1;OTUB1;OTUD7B;OTULIN;OXNAD1;PA2G4;PABPC4;PACSIN3;PAFAH1B1;PAFAH1B2;PAG1;PAGR1;PAK1IP1;PAK2;PANK1;PANX1;PAPOLG;PAQR3;PCDHGB4;PCK2;PCMT1;PDCD1;PDCD4;PDCD6IP;PDE3B;PDE4D;PDF;PDHX;PDIA6;PDIK1L;PDK4;PDLIM5;PDPR;PDXK;PELO;PEX12;PEX13;PEX14;PGD;PGLYRP1;PHC3;PHEX;PHF19;PHIP;PHKA1;PHKB;PHLDA2;PHLDB2;PHLPP2;PHYHIP;PI4K2B;PIK3R1;PIM1;PIP4K2B;PISD;PLAG1;PLAUR;PLCXD2;PLD3;PLEC;PLEKHA1;PLEKHB2;PLEKHM2;PLEKHN1;PLK1;PLPBP;PLPP3;PLRG1;PLSCR4;PMS1;PNISR;PNN;PNP;PNPLA6;PNPO;PNRC2;POFUT1;POLB;POLDIP2;POLDIP3;POLE4;POLR1C;POLR2A;POLR2E;POLR3A;POM121C;PON2;POTEF;POU2AF1;PPA1;PPAN;PPIF;PPIG;PPIL1;PPIP5K2;PPM1A;PPM1D;PPP1R11;PPP2R1A;PPP2R1B;PPP2R5C;PPP4R3B;PPP6C;PPP6R3;PPT1;PRDM4;PRELID3B;PRICKLE2;PRIM1;PRIMPOL;PRKAA1;PRKAB2;PRKAR1A;PRKAR2A;PRKCD;PRNP;PRPF8;PRPSAP1;PRR12;PRR3;PRRC2C;PRSS21;PSAT1;PSKH1;PSMB5;PSMC1;PSMC2;PSMD11;PSMD12;PSME3;PSME4;PSPH;PTCD3;PTGS2;PTPDC1;PTPN18;PTPN3;PTPRD;PTPRJ;PTPRT;PTRH1;PUM1;PURA;PVR;PWWP2A;PYGB;QSOX2;RAB11FIP2;RAB12;RAB15;RAB1A;RAB1B;RAB21;RAB23;RAB30;RAB3IP;RAB40B;RAB9B;RABGGTB;RACGAP1;RAD23B;RAD51C;RAF1;RALGAPB;RAN;RANGAP1;RAP2C;RAPH1;RARB;RARS;RASEF;RASSF2;RASSF5;RBBP6;RBM15B;RBM28;RBM6;RBMS1;RBMS3;RBPJ;RCAN3;RCC1L;RCL1;RCOR1;RECK;REL;RELT;REXO1;RFK;RFT1;RFWD2;RHOF;RHOT1;RHOV;RIC1;RICTOR;RIDA;RIMS3;RIOK3;RIOX2;RMDN1;RNASEH1P1;RNASEL;RNF111;RNF138;RNF144B;RNF149;RNF168;RNF217;RNF38;RNMT;RNPS1;ROGDI;RP1L1;RPH3AL;RPL10;RPL10L;RPL12;RPL13;RPL14;RPL21;RPL27A;RPL3;RPL30;RPL31;RPL36;RPL4;RPL5;RPL6;RPL9;RPLP0;RPLP1;RPRD1B;RPRD2;RPS17;RPS2;RPS24;RPS25;RPS27;RPS3;RPS3A;RPS5;RPS6;RPS6KA3;RPS6KB1;RPSA;RRP12;RRP15;RRP36;RRP9;RS1;RSL1D1;RTN4;RUBCN;RUNDC3B;RUNX1T1;S100A11P1;SACM1L;SALL1;SARS;SART1;SAV1;SBF1;SBNO1;SCAF4;SCAMP3;SCAMP4;SCAMP5;SDHAF2;SEC11A;SEC24A;SEC24B;SEC61A1;SEC61A2;SEH1L;SELENOI;SEMA4C;SENP6;SEPT2;SERBP1;SERINC5;SERPINB5;SERPINE2;SESTD1;SETD1B;SETD5;SF3A3;SF3B3;SFXN1;SGK3;SGTA;SH3BP4;SHOC2;SIDT2;SIK1;SIPA1L2;SIRT4;SKAP2;SKI;SLC11A2;SLC12A2;SLC16A3;SLC19A1;SLC1A5;SLC25A12;SLC25A22;SLC25A29;SLC25A32;SLC25A38;SLC25A39;SLC25A6;SLC27A4;SLC29A1;SLC2A3;SLC30A1;SLC35A1;SLC35A4;SLC35B2;SLC35B3;SLC35E2B;SLC38A1;SLC38A2;SLC38A5;SLC39A10;SLC39A14;SLC39A9;SLC3A2;SLC4A1AP;SLC4A2;SLC6A4;SLC7A1;SLC7A5;SLC9A1;SLC9A2;SLC9A6;SLCO3A1;SLFN13;SLIRP;SMAD1;SMAD3;SMAD5;SMAD7;SMARCA4;SMDT1;SMPD4;SMURF1;SMURF2;SNCG;SND1;SNRPA1;SNRPB2;SNRPC;SNTB2;SNX11;SNX12;SNX15;SNX16;SNX6;SOCS2;SOCS3;SOCS5;SON;SOWAHC;SOX5;SOX6;SP1;SPATA2;SPATA5;SPCS3;SPEN;SPOUT1;SPRED1;SPRYD3;SPTBN2;SPTLC1;SQSTM1;SREK1;SRP19;SRP68;SRP72;SRPK1;SRPRA;SRPRB;SRSF1;SSRP1;SSSCA1;SSU72;STAU1;STEAP3;STIP1;STK33;STK38;STRADB;STRAP;STT3B;STX17;STX4;STXBP3;SUCLA2;SUN1;SUPT16H;SUPT5H;SYF2;SYNJ1;SYNRG;SYPL1;SYT11;SZRD1;TACO1;TADA2B;TAF13;TAF15;TAF9;TAOK1;TARBP2;TASP1;TBC1D14;TBC1D20;TBCCD1;TBL1XR1;TBL3;TBP;TBPL1;TBRG1;TBRG4;TCF3;TCFL5;TCP1;TDRD3;TECPR2;TELO2;TEP1;TERF2IP;TES;TET3;TEX15;TFAP2A;TFAP4;TFB1M;TFPI;TFRC;TGFBI;TGFBR3;TGOLN2;THAP7;THEM4;THRAP3;TIA1;TIGD3;TIMM10B;TIMM13;TIMM17A;TIMP3;TKTL1;TLE4;TLK1;TLL1;TM4SF1;TM7SF3;TM9SF2;TMBIM6;TMCC1;TMED1;TMED7;TMEM100;TMEM109;TMEM126A;TMEM135;TMEM138;TMEM154;TMEM161B;TMEM168;TMEM189;TMEM189-UBE2V1;TMEM245;TMEM251;TMEM255A;TMEM41A;TMEM43;TMEM69;TMEM87A;TMTC3;TNFAIP2;TNFRSF10A;TNFRSF12A;TNFSF9;TNK1;TNPO1;TNPO3;TNRC6B;TOB2;TOMM34;TOR1A;TOR4A;TOX4;TP53;TPBG;TPI1;TPM2;TPM3;TPPP3;TPT1;TRAF4;TRAK1;TRAM1;TRIM32;TRIM35;TRIM4;TRIM44;TRMT1;TRMT10C;TRMT13;TRUB2;TSC22D2;TSPAN3;TSR1;TTC1;TTC17;TTF2;TTLL12;TTLL5;TUBA1A;TUBA1C

			;TUBB;TUBB2A;TUBB3;TUBB4B;TUBGCP2;TXLNG;TXN2;TXNIP;TXNL1;U2SURP;UBE2C;UBE2H;UBE2Q1;UBE2Q2;UBE2S;UBE2V1;UBE2Z;UBE3C;UBE4A;UBFD1;UBN2;UBR3;UBXN2B;UCA1;UFC1;UFSP2;UGDH;UGP2;UGT2B4;UGT8;ULK1;UNG;UQCC3;USMG5;USP15;USP3;USP31;USP42;USP48;USP53;USP7;USP8;USP9X;UTP14A;UTP15;UTP20;UTP23;UTP3;UTP4;UXT;VAMP8;VASN;VAV2;VCL;VEGFA;VEZT;VIM;VKORC1;VMA21;VMP1;VOPP1;VPS33B;VPS45;VPS4A;VSIR;VTI1B;WARS;WBP11;WDR13;WDR18;WDR3;WDR43;WDR5B;WDR75;WEE1;WIPF1;WIPI2;WNK3;WNT3A;WNT4;WNT5A;WT1;XKR7;XKR8;XPNPEP3;XPO1;XPO4;XPO6;XPO7;XPOT;XYLT1;YAP1;YARS2;YBX1;YBX3;YIF1B;YIPF2;YIPF4;YIPF6;YRDC;YTHDC1;YWHAH;YWHAQ;YY1AP1;ZBTB10;ZBTB16;ZBTB2;ZBTB33;ZBTB34;ZBTB5;ZC3H11A;ZCCHC3;ZDHHC16;ZEB2;ZFHX3;ZFHX4;ZFP28;ZFPL1;ZMAT3;ZNF207;ZNF267;ZNF275;ZNF280C;ZNF284;ZNF367;ZNF384;ZNF391;ZNF449;ZNF460;ZNF559;ZNF585B;ZNF598;ZNF620;ZNF622;ZNF638;ZNF644;ZNF691;ZNF704;ZNF791;ZNF827;ZNF91;ZNRF1;ZNRF2;ZNRF3;ZRANB1;ZRANB2;ZYX;ZZEF1
hsa-miR-16-5p-477860_mir	-0.531	0.245	AAAS;AADAT;ABCB7;ABCC1;ABCC4;ABCC6;ABCF1;ABCF2;ABHD10;ABHD2;ABL2;ACBD5;ACOT8;ACOX1;ACP2;ACTB;ACTG1;ACTN1;ACTN4;ACTR1A;ACTR2;ACTR3B;ACVR2A;ADAD2;ADGRE5;ADGRL1;ADK;ADORA2A;ADORA3;ADRA2B;ADSS;AFF4;AFG3L2;AGER;AGK;AGO2;AGO4;AGPAT5;AGRN;AHCYL1;AHCYL2;AHNAK2;AIFM2;AIMP1;AK2;AKAP11;AKAP13;AKR1B10;AKT3;ALDH18A1;ALDH2;ALDH3B1;ALG2;ALG3;ALKAL2;AMER1;AMOT;AMOTL1;AMPD1;ANAPC13;ANAPC16;ANKLE2;ANKMY1;ANKRD13B;ANKRD17;ANKRD36;ANPEP;AP2A1;AP2B1;AP2M1;AP3D1;AP3M1;AP5Z1;APLNR;APP;AQP12B;ARCN1;ARG2;ARHGAP12;ARHGAP32;ARHGDIA;ARIH1;ARL10;ARL2;ARL2BP;ARL3;ARMC12;ARMCX2;ARPC5L;ASB6;ASCC1;ASCC3;ASGR2;ASH1L;ASNS;ASPH;ASXL1;ASXL2;ATAD5;ATF7;ATG14;ATG9A;ATL2;ATOX1;ATP13A3;ATP5A1;ATP5C1;ATP5G3;ATP6V0E1;ATP6V1B2;ATP6V1E1;ATP8A2;ATXN2L;ATXN7L3;ATXN7L3B;AUP1;AURKAIP1;AURKB;AVL9;AXIN2;B3GNT2;B4GALT1;BACE1;BAG4;BAG6;BAMBI;BAZ1B;BAZ2A;BCAS2;BCCIP;BCL11B;BCL2;BCL2L12;BCL7A;BDNF;BEX3;BFAR;BHLHE40;BIRC5;BMI1;BMS1;BNC2;BRAT1;BRCA1;BSG;BSPRY;BTAF1;BTBD2;BTF3;BTG2;BTN3A3;BTRC;BYSL;BZW1;C11ORF24;C15ORF39;C16ORF58;C16ORF72;C17ORF75;C17ORF80;C19ORF54;C1ORF21;C1ORF226;C1ORF56;C21ORF62;C2ORF42;C2ORF74;C3ORF36;C6ORF106;C8ORF44;CA12;CA8;CAAP1;CABIN1;CACNA2D1;CACNB2;CACUL1;CADM1;CALR;CALU;CAMK2G;CAMKK2;CAMKV;CAMSAP1;CAMSAP3;CANX;CAPRIN1;CAPZA2;CARD10;CARD19;CARD8;CARM1;CASK;CASKIN1;CBFA2T3;CBX2;CBX4;CBX6;CCDC58;CCDC59;CCDC80;CCDC83;CCDC88C;CCND1;CCND2;CCND3;CCNE1;CCNE2;CCNJ;CCNT1;CCNT2;CCPG1;CCT3;CCT6B;CCT8;CD180;CD274;CD2AP;CD44;CD55;CDADC1;CDC123;CDC14B;CDC20;CDC23;CDC25A;CDC27;CDC37;CDC37L1;CDC42SE2;CDC5L;CDCA4;CDCA8;CDIPT;CDK1;CDK17;CDK5RAP1;CDK6;CDK9;CDKN1A;CDKN2A;CDKN2AIPNL;CDS2;CDV3;CENPF;CENPJ;CEP55;CEP63;CFAP45;CFL2;CHAC1;CHD3;CHEK1;CHERP;CHIC1;CHMP3;CHMP4B;CHORDC1;CHPT1;CHUK;CIB1;CKAP5;CLASP1;CLDN2;CLEC2D;CLIP2;CLIP4;CLNS1A;CLSPN;CLTC;CLU;CLUH;CMPK1;CMTM4;CNKSR3;CNN3;CNNM2;CNOT7;CNP;CNPY3;COA6;COA7;COL4A1;COL4A2;COMMD10;COMT;COPS5;COPS7B;COQ3;CPEB2;CPEB3;CPNE1;CPNE8;CPSF7;CPT1A;CREB3L2;CREBL2;CREBRF;CREG1;CRHBP;CRIM1;CRK;CRKL;CSDE1;CSGALNACT1;CSHL1;CSNK1E;CTDSPL;CTSD;CUL2;CUL3;CUL4A;CXORF38;CXORF40B;CYB561A3;CYB5R1;CYCS;CYLD;CYP26B1;CYP27B1;DAP3;DCAF13;DCAF17;DCAF7;DCTN5;DCUN1D5;DDHD2;DDN;DDX21;DDX31;DDX3X;DDX3Y;DDX41;DDX52;DDX54;DDX6;DECR1;DENND6A;DESI1;DHTKD1;DHX30;DHX35;DHX36;DHX37;DHX38;DHX8;DIABLO;DIAPH1;DICER1;DIEXF;DIXDC1;DKC1;DLC1;DLD;DLGAP3;DMAP1;DMD;DMPK;DMRT2;DMTF1;DNAAF5;DNAJA1;DNAJA2;DNAJA4;DNAJB1;DNAJB4;DNAJC1;DNAJC10;DNAJC15;DNAJC2;DNAJC9;DNTTIP2;DOCK11;DOCK5;DOCK9;DPP8;DSCR3;DSP;DTD1;DUSP14;DYNLL2;DYRK3;E2F3;E2F7;EARS2;ECHDC1;EDC3;EDC4;EEF1A1;EEF1G;EEF2;EEF2K;EFNB2;EFTUD2;EGFR;EGLN2;EIF1;EIF1AX;EIF2A;EIF2B2;EIF2B5;EIF3A;EIF3C;EIF3CL;EIF3E;EIF3F;EIF3H;EIF3K;EIF3M;EIF4B;EIF4E;EIF4G1;EIF4G3;EIF5;EIF5B;ELAC2;ELK4;ELOVL1;ELOVL5;ELP3;EMC6;EML4;EN2;ENPP4;ENTPD1;ENTPD6;ENTPD7;ENY2;EPC1;EPHA2;EPM2AIP1;ERLIN2;ETFRF1;ETNK1;EXD2;EXT1;EZH1;F2;FAM103A1;FAM122A;FAM122B;FAM122C;FAM129B;FAM155B;FAM168A;FAM189B;FAM208A;FAM229B;FAM69A;FAM89A;FAM96B;FASN;FASTKD1;FASTKD2;FAT2;FAT3;FBXL18;FBXL20;FBXO3;FBXO41;FBXW11;FBXW7;FCF1;FDXR;FECH;FEM1C;FGF2;FGFR1;FGFR4;FKBP1A;FLCN;FLNA;FLOT2;FNBP1;FNDC3B;FNTA;FOXK1;FRYL;FSCN2;FTH1;FTL;FUBP1;FURIN;FZD6;FZD9;G3BP2;GABARAP;GABARAPL1;GABPA;GALNT1;GALNT3;GALNT7;GANAB;GARS;GATAD2A;GCC1;GEMIN4;GEMIN5;GFM1;GFPT1;GGA3;GLB1L3;GLP2R;GLRX;GLS2;GLT8D1;GNAL;GNAT1;GNB1;GNB2;GNG12;GNL3L;GOLGA5;GOLGA7;GOLPH3L;GOLT1B;GOSR1;GOT2;GPAA1;GPAM;GPATCH4;GPATCH8;GPR157;GPR180;GPR27;GPR55;GPRC5A;GRAMD2B;GRB2;GRPEL2;GRWD1;GSG1;GSK3B;GSTM4;GSTT2B;GTF2H1;GTF3C1;GTF3C2;GTF3C3;GTF3C4;GTPBP1;GTPBP4;GTPBP8;H2AFX;H3F3B;HACE1;HARS;HARS2;HAUS3;HCFC2;HDGF;HDHD2;HDHD5;HEATR1;HEATR3;HELZ;HERC6;HEYL;HGF;HIGD1A;HIST1H1C;HIST1H2AJ;HIST1H2BC;HIST1H2BK;HIST1H4B;HIST2H2BE;HM13;HMBOX1;HMGA1;HMGA2;HMGN1;HMOX1;HNRNPA1;HNRNP

			A1L2;HNRNPA2B1;HNRNPDL;HNRNPF;HNRNPL;HOXA10;HOXA3;HOXC8;HOXD13;HPF1;HSD17B8;HSDL2;HSP90AA1;HSP90B1;HSP90B2P;HSPA1A;HSPA1B;HSPA4L;HSPA5;HSPA8;HSPA9;HSPBP1;HSPD1;HSPE1-MOB4;HSPH1;HTRA1;HTT;HYAL3;HYOU1;IARS;IBTK;IDH3A;IDS;IER2;IER3IP1;IFNG;IFRD1;IFRD2;IFT74;IGF1R;IGF2R;IGSF1;IKZF4;IL12B;IL36RN;IMPAD1;IMPDH2;INF2;INO80D;INSL6;INTS5;IPO11;IPO4;IPO5;IPO7;IPPK;IRAK1BP1;IRAK3;IRF4;IRS4;ITGA2;ITGB4;ITPR1;IVNS1ABP;IWS1;JAK2;JARID2;JPT2;JUN;KANK1;KANSL3;KATNAL1;KCNC4;KCND3;KCNG3;KCNN4;KCNS2;KDM2A;KDR;KDSR;KIAA0368;KIAA0895;KIAA1456;KIAA2013;KIDINS220;KIF14;KIF1B;KIF23;KIF2A;KIF2C;KIF3B;KIF5A;KIF5B;KIN;KLC2;KLF14;KLHDC10;KLHL15;KLHL34;KLHL40;KMT2A;KMT2D;KPNA1;KPNA2;KPNA3;KRAS;KRT33B;L1CAM;L2HGDH;LAMB1;LAMB3;LAMC1;LAMP2;LAMTOR1;LAMTOR2;LAMTOR3;LAMTOR4;LAMTOR5;LANCL1;LAPTM4B;LARP1;LARP4;LATS1;LDAH;LIG4;LIMA1;LIN7C;LITAF;LMAN2L;LMF2;LMO7;LMTK3;LONP1;LONRF2;LRIF1;LRIG2;LRPPRC;LRRC57;LRRFIP2;LSG1;LSM10;LSM11;LSM5;LUC7L;LUC7L3;LURAP1L;LUZP1;LY6K;LYAR;LYPD2;LYPLA2;MACF1;MAFK;MAIP1;MALSU1;MAP2K3;MAP3K7;MAP4;MAP4K2;MAP7;MAPKAPK2;MARCH6;MARS;MBD4;MCFD2;MCL1;MCM3AP-AS1;MCU;MDN1;MED11;MED12;MED13;MED24;MEGF8;MEPCE;MERTK;METAP2;METTL13;MFN2;MGAT4A;MIB1;MICU2;MIGA1;MINK1;MIS12;MKI67;MKX;MLF2;MLLT1;MLLT11;MLLT6;MLXIP;MMP25;MMS19;MOB3C;MOB4;MORF4L1;MOV10;MPDU1;MRC2;MRFAP1;MRM3;MRPL1;MRPL10;MRPL12;MRPL20;MRPL21;MRPL3;MRPL40;MRPS10;MRPS12;MRPS14;MRPS2;MRPS23;MRPS25;MRPS31;MRPS35;MRRF;MSANTD4;MSH2;MSL1;MSL3;MT-CO1;MTFR1L;MTHFD1L;MTHFD2;MTHFR;MTMR3;MTMR4;MTOR;MYB;MYC;MYO19;MYO5A;MYO5B;N4BP1;NAA10;NAA15;NAA25;NACA;NAMPT;NAPG;NARF;NARS;NARS2;NAT8L;NAV2;NCAPD2;NCAPG;NCKAP1;NCKAP5L;NCOR2;NCSTN;NDUFA4;NDUFA9;NDUFAF4;NEGR1;NEMF;NEURL4;NFIC;NFKB1;NHLRC3;NIN;NIPAL2;NISCH;NLE1;NMD3;NNT;NOB1;NOC3L;NOC4L;NOL11;NOL4L;NOP10;NOP14;NOP53;NOTCH2;NPR3;NPRL3;NR1I2;NR2C2;NR6A1;NRP1;NRXN1;NSF;NSUN2;NT5C3A;NT5DC1;NT5DC2;NTHL1;NUCKS1;NUDT3;NUFIP2;NUP155;NUP160;NUP50;NUP98;NXPH2;OCRL;ODF2L;OGDH;OGT;OIP5;OMA1;ONECUT2;OPRM1;ORAI2;ORC4;OSBPL3;OSCAR;OSGEPL1;OTOL1;OTUB1;OTUD7B;OTULIN;OXNAD1;PA2G4;PABPC4;PACSIN3;PAFAH1B1;PAFAH1B2;PAG1;PAGR1;PAK1IP1;PAK2;PANK1;PANX1;PAPOLG;PAQR3;PCDHGB4;PCK2;PCMT1;PDCD1;PDCD4;PDCD6IP;PDE3B;PDE4D;PDF;PDHX;PDIA6;PDIK1L;PDK4;PDLIM5;PDPR;PDXK;PELO;PEX12;PEX13;PEX14;PGD;PGLYRP1;PHC3;PHEX;PHF19;PHIP;PHKA1;PHKB;PHLDA2;PHLDB2;PHLPP2;PHYHIP;PI4K2B;PIK3R1;PIM1;PIP4K2B;PISD;PLAG1;PLAUR;PLCXD2;PLD3;PLEC;PLEKHA1;PLEKHB2;PLEKHM2;PLEKHN1;PLK1;PLPBP;PLPP3;PLRG1;PLSCR4;PMS1;PNISR;PNN;PNP;PNPLA6;PNPO;PNRC2;POFUT1;POLB;POLDIP2;POLDIP3;POLE4;POLR1C;POLR2A;POLR2E;POLR3A;POM121C;PON2;POTEF;POU2AF1;PPA1;PPAN;PPIF;PPIG;PPIL1;PPIP5K2;PPM1A;PPM1D;PPP1R11;PPP2R1A;PPP2R1B;PPP2R5C;PPP4R3B;PPP6C;PPP6R3;PPT1;PRDM4;PRELID3B;PRICKLE2;PRIM1;PRIMPOL;PRKAA1;PRKAB2;PRKAR1A;PRKAR2A;PRKCD;PRNP;PRPF8;PRPSAP1;PRR12;PRR3;PRRC2C;PRSS21;PSAT1;PSKH1;PSMB5;PSMC1;PSMC2;PSMD11;PSMD12;PSME3;PSME4;PSPH;PTCD3;PTGS2;PTPDC1;PTPN18;PTPN3;PTPRD;PTPRJ;PTPRT;PTRH1;PUM1;PURA;PVR;PWWP2A;PYGB;QSOX2;RAB11FIP2;RAB12;RAB15;RAB1A;RAB1B;RAB21;RAB23;RAB30;RAB3IP;RAB40B;RAB9B;RABGGTB;RACGAP1;RAD23B;RAD51C;RAF1;RALGAPB;RAN;RANGAP1;RAP2C;RAPH1;RARB;RARS;RASEF;RASSF2;RASSF5;RBBP6;RBM15B;RBM28;RBM6;RBMS1;RBMS3;RBPJ;RCAN3;RCC1L;RCL1;RCOR1;RECK;REL;RELT;REXO1;RFK;RFT1;RFWD2;RHOF;RHOT1;RHOV;RIC1;RICTOR;RIDA;RIMS3;RIOK3;RIOX2;RMDN1;RNASEH1P1;RNASEL;RNF111;RNF138;RNF144B;RNF149;RNF168;RNF217;RNF38;RNMT;RNPS1;ROGDI;RP1L1;RPH3AL;RPL10;RPL10L;RPL12;RPL13;RPL14;RPL21;RPL27A;RPL3;RPL30;RPL31;RPL36;RPL4;RPL5;RPL6;RPL9;RPLP0;RPLP1;RPRD1B;RPRD2;RPS17;RPS2;RPS24;RPS25;RPS27;RPS3;RPS3A;RPS5;RPS6;RPS6KA3;RPS6KB1;RPSA;RRP12;RRP15;RRP36;RRP9;RS1;RSL1D1;RTN4;RUBCN;RUNDC3B;RUNX1T1;S100A11P1;SACM1L;SALL1;SARS;SART1;SAV1;SBF1;SBNO1;SCAF4;SCAMP3;SCAMP4;SCAMP5;SDHAF2;SEC11A;SEC24A;SEC24B;SEC61A1;SEC61A2;SEH1L;SELENOI;SEMA4C;SENP6;SEPT2;SERBP1;SERINC5;SERPINB5;SERPINE2;SESTD1;SETD1B;SETD5;SF3A3;SF3B3;SFXN1;SGK3;SGTA;SH3BP4;SHOC2;SIDT2;SIK1;SIPA1L2;SIRT4;SKAP2;SKI;SLC11A2;SLC12A2;SLC16A3;SLC19A1;SLC1A5;SLC25A12;SLC25A22;SLC25A29;SLC25A32;SLC25A38;SLC25A39;SLC25A6;SLC27A4;SLC29A1;SLC2A3;SLC30A1;SLC35A1;SLC35A4;SLC35B2;SLC35B3;SLC35E2B;SLC38A1;SLC38A2;SLC38A5;SLC39A10;SLC39A14;SLC39A9;SLC3A2;SLC4A1AP;SLC4A2;SLC6A4;SLC7A1;SLC7A5;SLC9A1;SLC9A2;SLC9A6;SLCO3A1;SLFN13;SLIRP;SMAD1;SMAD3;SMAD5;SMAD7;SMARCA4;SMDT1;SMPD4;SMURF1;SMURF2;SNCG;SND1;SNRPA1;SNRPB2;SNRPC;SNTB2;SNX11;SNX12;SNX15;SNX16;SNX6;SOCS2;SOCS3;SOCS5;SON;SOWAHC;SOX5;SOX6;SP1;SPATA2;SPATA5;SPCS3;SPEN;SPOUT1;SPRED1;SPRYD3;SPTBN2;SPTLC1;SQSTM1;SREK1;SRP19;SRP68;SRP72;SRPK1;SRPRA;SRPRB;SRSF1;SSRP1;SSSCA1;SSU72;STAU1;STEAP3;STIP1;STK33;STK38;STRADB;STRAP;STT3B;STX17;STX4;STXBP3;SUCLA2;SUN1;SUPT16H;SUPT5H;SYF2;SYNJ1;S

			YNRG;SYPL1;SYT11;SZRD1;TACO1;TADA2B;TAF13;TAF15;TAF9;TAOK1;TARBP2;TASP1;TBC1D14;TBC1D20;TBCCD1;TBL1XR1;TBL3;TBP;TBPL1;TBRG1;TBRG4;TCF3;TCFL5;TCP1;TDRD3;TECPR2;TELO2;TEP1;TERF2IP;TES;TET3;TEX15;TFAP2A;TFAP4;TFB1M;TFPI;TFRC;TGFBI;TGFBR3;TGOLN2;THAP7;THEM4;THRAP3;TIA1;TIGD3;TIMM10B;TIMM13;TIMM17A;TIMP3;TKTL1;TLE4;TLK1;TLL1;TM4SF1;TM7SF3;TM9SF2;TMBIM6;TMCC1;TMED1;TMED7;TMEM100;TMEM109;TMEM126A;TMEM135;TMEM138;TMEM154;TMEM161B;TMEM168;TMEM189;TMEM189-UBE2V1;TMEM245;TMEM251;TMEM255A;TMEM41A;TMEM43;TMEM69;TMEM87A;TMTC3;TNFAIP2;TNFRSF10A;TNFRSF12A;TNFSF9;TNK1;TNPO1;TNPO3;TNRC6B;TOB2;TOMM34;TOR1A;TOR4A;TOX4;TP53;TPBG;TPI1;TPM2;TPM3;TPPP3;TPT1;TRAF4;TRAK1;TRAM1;TRIM32;TRIM35;TRIM4;TRIM44;TRMT1;TRMT10C;TRMT13;TRUB2;TSC22D2;TSPAN3;TSR1;TTC1;TTC17;TTF2;TTLL12;TTLL5;TUBA1A;TUBA1C;TUBB;TUBB2A;TUBB3;TUBB4B;TUBGCP2;TXLNG;TXN2;TXNIP;TXNL1;U2SURP;UBE2C;UBE2H;UBE2Q1;UBE2Q2;UBE2S;UBE2V1;UBE2Z;UBE3C;UBE4A;UBFD1;UBN2;UBR3;UBXN2B;UCA1;UFC1;UFSP2;UGDH;UGP2;UGT2B4;UGT8;ULK1;UNG;UQCC3;USMG5;USP15;USP3;USP31;USP42;USP48;USP53;USP7;USP8;USP9X;UTP14A;UTP15;UTP20;UTP23;UTP3;UTP4;UXT;VAMP8;VASN;VAV2;VCL;VEGFA;VEZT;VIM;VKORC1;VMA21;VMP1;VOPP1;VPS33B;VPS45;VPS4A;VSIR;VTI1B;WARS;WBP11;WDR13;WDR18;WDR3;WDR43;WDR5B;WDR75;WEE1;WIPF1;WIPI2;WNK3;WNT3A;WNT4;WNT5A;WT1;XKR7;XKR8;XPNPEP3;XPO1;XPO4;XPO6;XPO7;XPOT;XYLT1;YAP1;YARS2;YBX1;YBX3;YIF1B;YIPF2;YIPF4;YIPF6;YRDC;YTHDC1;YWHAH;YWHAQ;YY1AP1;ZBTB10;ZBTB16;ZBTB2;ZBTB33;ZBTB34;ZBTB5;ZC3H11A;ZCCHC3;ZDHHC16;ZEB2;ZFHX3;ZFHX4;ZFP28;ZFPL1;ZMAT3;ZNF207;ZNF267;ZNF275;ZNF280C;ZNF284;ZNF367;ZNF384;ZNF391;ZNF449;ZNF460;ZNF559;ZNF585B;ZNF598;ZNF620;ZNF622;ZNF638;ZNF644;ZNF691;ZNF704;ZNF791;ZNF827;ZNF91;ZNRF1;ZNRF2;ZNRF3;ZRANB1;ZRANB2;ZYX;ZZEF1
hsa-miR-139-5p-478312_mir	-3.678	0.247	ABL2;ACTC1;ADAMTS17;ADGRL4;ARHGAP32;B3GALNT2;BCL2;BCL2L15;BTG2;C21ORF59;CAND1;CCT5;CHD9;CIAPIN1;CLIC4;CTTNBP2NL;CXCR4;DCAF4L1;DCBLD2;DDX6;DERL1;DUSP22;EPGN;FAM162A;FAM84B;FBXL18;FNBP4;FOS;GABARAP;GLP2R;HMGB2;HNRNPF;HOOK1;HRAS;HSP90AA1;IGF1R;IPPK;JUN;KCND3;KCNQ5;KLF10;KLHL36;KPNA2;KSR2;LCOR;MARCH9;MCL1;MET;MKL2;MLEC;MMP11;NANOGNB;NCR3LG1;NFKB1;NOTCH1;NR5A2;OIP5;PAGR1;PAPD4;PDE4D;PDE4DIP;PDZD8;PEX5L;PGAM4;PIK3CA;PSME3;PTGFRN;RAP1B;RAP2C;RHOT1;ROCK2;RPS23;RPS26;RREB1;SGPL1;SLC25A6;SLC39A6;SMARCA4;SPATA13;SPRY4;SRPK1;STAMBP;SUGT1;SYT5;TAF13;TAF1D;TCF12;TGS1;TMC7;TMED7;TMEM19;TMPRSS12;TNPO1;TNRC6A;TPD52;UHMK1;USP6NL;WNT1;YTHDF1;ZBTB26;ZBTB34;ZHX2;ZNF367;ZNF431;ZNF480
hsa-miR-101-3p-477863_mir	-2.160	0.251	ABHD17C;ACVR2B;ADO;AEBP2;AFF4;AGO4;AKAP11;ALG14;AMD1;AMMECR1L;ANKDD1A;ANKFY1;ANKRD11;ANKRD17;AP1G1;AP1S3;AP3M1;APP;ARAP2;ARID1A;ARID5B;ATG12;ATG4D;ATM;ATP5B;ATXN1;ATXN1L;B3GALNT2;BCL2L11;BCL9;BEND4;BICD2;BIRC5;BLOC1S6;BMT2;BTG2;BTRC;BZW1;C10ORF88;C1ORF147;C1ORF52;C8ORF4;CADM1;CAPN2;CAPZB;CARNMT1;CBFA2T2;CBX4;CCDC125;CCND1;CCNF;CD180;CD46;CD81;CDC123;CDC42EP4;CDC7;CDH5;CDK8;CDKN1A;CERK;CERS2;CFTR;CHAMP1;CLIC4;CMTM6;CNEP1R1;COX10;CPEB1;CPS1;CTNNB1;CTR9;DAZAP2;DCAF12L2;DCBLD2;DCTD;DDIT4;DDX19B;DENND5B;DICER1;DIDO1;DIMT1;DNAJA1;DNAJC28;DNMT3A;DSC1;DUSP1;DYNC1LI2;DYRK2;E2F3;EEA1;EED;EIF4G2;ELAVL2;ELAVL3;EXTL3;EYA1;EZH2;FAM103A1;FAM217B;FAM69A;FAM84B;FAR1;FBN2;FBXO11;FBXW7;FGF2;FKBP14;FMR1;FNDC3A;FOS;FOXP4;FRMD6;FRS2;FZD6;G3BP1;GAN;GCLC;GFPT2;GLRX5;GMEB2;GNB1;GOLGA7;GPAM;GPR135;GPR50;GRIK3;GRSF1;HFE;HNRNPAB;HNRNPF;HNRNPU;HOXA9;HSP90AA1;HSP90B1;HSPA13;HSPE1-MOB4;ICK;IER5;IL20RB;INA;INO80D;IPO7;ITGA3;JAK2;JCAD;JUN;KCNG3;KCNQ5;KCTD14;KDM3B;KDM6B;KIAA1456;KIAA1586;KIF2C;KLF12;KLF6;KLHL23;KPNA2;L2HGDH;LANCL3;LBR;LCOR;LDB1;LEFTY1;LIFR;LIN28B;LIN7C;LMNB1;LRCH2;LRRC1;LTN1;LYSMD3;LZIC;MAML3;MAP2K1;MAP3K4;MBNL1;MBNL2;MBTD1;MCL1;MEIS1;MET;MFSD6;MITF;MKLN1;MKNK2;MLEC;MMS22L;MNX1;MOB4;MORC3;MPPE1;MRGBP;MRPL42;MRPL44;MSH2;MT-CO2;MTOR;MTSS1L;MYCN;MYO9A;N4BP1;NAA30;NACA;NACA2;NACC1;NANOGNB;NAP1L1;NCKAP1;NEK7;NKAP;NKX3-2;NLK;NOP2;NOTCH1;NR2F2;NR2F6;NT5C3A;NUFIP2;NUPL2;NXT2;ORAI2;OTUD4;PABPC1L2A;PAFAH1B1;PAK3;PANK1;PAPD7;PCCB;PDK1;PEX5L;PGBD4;PHF3;PIAS1;PIK3C2B;PIK3CB;PIK3CD;PIM1;PIP4K2A;PIP5K1C;PLAG1;PLEKHA1;PLEKHA3;POU2F1;PPP1CC;PPP1R15B;PPP2R2A;PPP2R5E;PPP4R1;PRDM1;PRDM16;PRKAA1;PRKAB1;PRPF38B;PSPC1;PTGER4;PTGS2;PURB;QDPR;QSER1;RAB11FIP1;RAB33B;RAB39B;RAB5A;RAB8B;RAC1;RANBP9;RAP1B;RAP2C;RARS2;RBM12B;REEP5;REL;RHOA;RIOK2;RMI1;RNF111;RNF152;RNF213;RNF219;RNF44;RORA;RPL7L1;RPS6KA5;RREB1;RRM2;RTN4;RUNX1;SACM1L;SEPT11;SGPL1;SHMT1;SIX4;SLC11A2;SLC25A33;SLC30A5;SLC35F5;SLC38A2;SLC39A6;SLC7A2;SMARCA5;SMARCD1;SMN2;SNHG1;SNRNP27;SNRNP35;SOX9;SPAG1;SPATA2;SPIRE1;SREBF2;SREK1IP1;SRF;SSFA2;STAMBP;STMN1;STX16;STYX;SUB1;SUZ12;SZRD1;TAF13;TBC1D12;TBX18;TB

			X20;TET2;TFAP4;TGFBR1;TGFBR2;TGFBR3;TGIF2;TGOLN2;THRB;TMED5;TMEM168;TMEM170B;TMEM192;TMTC3;TNFAIP1;TNPO1;TNRC18P2;TOR1AIP1;TOR1AIP2;TRERF1;TRIB1;TSC22D2;TSN;TSPAN12;TTC37;TVP23C;UBE2A;UBE2B;UBE2D3;UBN2;UGT2A1;UGT2A2;USP25;USP36;VAPA;VEGFA;VEGFC;VEZT;VHL;WNK1;WNT7A;XIAP;XPO7;ZBTB21;ZBTB7A;ZC3H11A;ZCCHC2;ZDHHC15;ZDHHC24;ZEB1;ZEB2;ZFP36L2;ZFX;ZNF100;ZNF124;ZNF207;ZNF223;ZNF284;ZNF350;ZNF431;ZNF480;ZNF490;ZNF567;ZNF645;ZNF654;ZNF792;ZNF800;ZNF827
hsa-miR-153-3p-477922_mir	-1.292	0.260	ABCC12;ABLIM1;ACVR1B;ARF1;ARID1A;AZF1;BAMBI;BCL2;BMPR2;C16ORF52;CBFB;CDKN1B;CEP350;CERK;COX15;CRIPT;CSNK1A1;CTTN;DDIT4;ERGIC2;FAM210B;FEM1C;FOXO1;FXR1;GDNF;GOLGA8A;GOLGA8IP;GOLGA8J;GUCY2C;GULP1;HCCS;HECTD3;HMGB1;IDO1;KANK4;KIAA1191;KLF5;KMT2B;LAMC1;MAGEA3;MAGEA6;MCL1;MED28;MESD;MKNK2;MORC3;MTDH;NFE2L2;P2RY1;POLE3;PPM1D;PRDX2;PTEN;QTRT2;RAET1L;REEP5;RPL7L1;RPS6KA5;RPS9;SATB1;SEPT8;SH3PXD2A;SIX4;SKA2;SLAIN2;SLC10A3;SLC16A9;SMIM13;SNAI1;SNCA;SNTB2;SS18;TRIP10;TRUB1;ULBP2;UNKL;USP28;USP6NL;YKT6;ZBTB43;ZCCHC14;ZEB2;ZNF354B;ZNF703;ZNFX1
hsa-miR-135a-5p-478581_mir	-7.021	0.261	AMOTL2;APC;APOA1;ARC;ATXN7L1;AZF1;BCL2;BIRC5;BMPR2;CAPZA2;CCDC85C;CDR1;CEBPD;CENPN;CEP135;COX6B1;DAPK2;DPP8;E2F1;EGFR;ESRRA;FOXO1;GAGE1;GATA6;GNL1;GPX8;HEYL;HMGB2;HOXA10;HTR1A;IL17RA;IRS2;JAK2;KIAA1143;KIAA1958;KIF6;KLF4;KLF8;LAX1;LDHA;LRRC15;MARCKS;MBNL1;MMP11;MPL;MTSS1;MYC;NHS;NR3C2;NSA2;NUFIP2;PCP4L1;PCTP;PEX2;PHLPP2;PIAS4;PIP5K1A;POLH;PPM1E;PTK2;PTPRD;RAB3GAP2;RBAK;ROCK1;ROCK2;RUNX2;SCYL3;SIAH1;SKIL;SLC19A3;SLC39A6;SLC6A4;SMAD5;SNED1;SRC;ST8SIA4;STAT6;TNPO2;TOX4;TRAF6;TRIM4;TRIM66;TSC22D2;TTLL7;TXNIP;VLDLR;VNN3;WDR82P1;XBP1P1;ZNF107;ZNF468;ZNF609;ZNF805;ZNF846
hsa-miR-148a-3p-477814_mir	-1.771	0.264	ABLIM1;ACVR1;ADARB1;AGO2;AGO3;AKAP17A;ALCAM;AMELX;ANP32A;AP5B1;APC;APLN;APLP2;APPBP2;ARID3A;ARL6IP1;ARL8B;ARRDC3;ASB6;AURKB;B4GALT7;BAX;BAZ2B;BCL2;BCL2L11;BMP3;BTBD3;CBX3;CCKBR;CCNA2;CCNI;CCT6A;CDC25B;CDK19;CDKN1A;CDKN1B;CEBPG;CEP55;CHRFAM7A;CNOT4;COLEC12;CSNK2A1;CYCS;DCUN1D3;DDX6;DENR;DICER1;DNAJB4;DNMT1;DNMT3B;DSTYK;DTX4;DYNLL2;DYRK1A;EOGT;ERRFI1;ETV7;FAM104A;FAM212B;FOXP1;FURIN;FXR1;FZD5;GAS1;GLRX5;GNB5;GOLIM4;GPATCH8;GPRC5A;HCCS;HLA-A;HLA-C;HLA-G;HMGB1;HOXC8;HSP90AA1;HSP90B1;HSPA4;IGFBP5;IKBKB;IL23R;INO80;IRS1;ITGA5;ITGB8;JARID2;KANSL1;KDM6B;KIAA0907;KIAA1456;KIAA1549;KIF2C;KLF6;KPNA4;LBR;LDLR;LNPEP;LYSMD1;MAFB;MAP3K4;MAP3K9;MCFD2;MET;MLEC;MMP7;MPP5;MRPL45;MRPS27;MSL3;MTMR9;MYC;MYCBP2;MYO3A;NDRG1;NEURL4;NONO;NPTX1;NR1I2;NRP1;OR2C3;OTUD4;OVOL1;PAN3;PAPD4;PATL1;PBXIP1;PDIA3;PDIK1L;PHACTR2;PHLDB2;PLA2G12A;PLPP4;POC1A;POFUT1;PPARD;PPP6R1;PRNP;PSMD9;PTPN23;PTPN4;QKI;RAB10;RAB12;RAB14;RAB1B;RAB34;RALY;RASSF8;RBM23;RBM38;RCC2;RFT1;RNF219;ROCK1;RPS17;RPS6KA4;RPS6KA5;RUNX3;S1PR1;S1PR2;SECISBP2L;SERPINE1;SESN3;SESTD1;SH3PXD2A;SIK1;SLC12A7;SLC25A3;SLC2A3;SLC38A2;SMAD2;SNAPIN;SORD;SOS2;SPRY2;STARD13;STAT3;STX16;STX6;TGFB2;TGIF2;TMED7;TMEM14A;TMEM246;TMEM9B;TNRC6A;TNRC6B;TRIM59;TTLL1;TXNIP;UBE2D3;UNKL;UQCRQ;USP38;USP4;VAV2;VGLL2;VPS37A;VPS37B;VPS41;WAPL;WASL;WDTC1;WNT1;WNT10B;WNT2B;YPEL1;YWHAB;ZDHHC6;ZFYVE26;ZIC5;ZNF490;ZNF92
hsa-miR-494-3p-478135_mir	-2.801	0.272	ACACA;AGPS;AJAP1;AKT1;ARHGAP12;ARHGAP5;ARNTL;ARSJ;ATF3;ATXN1;BAG1;BASP1;BCL2;BCL2L11;BIRC5;BMI1;C2CD4A;CC2D1B;CCDC149;CCND1;CDK6;CDS2;CEP97;CFTR;CMPK1;CNR1;CREG2;CXCR4;CYCS;DAZAP2;DCAF4L2;DCAF7;DCBLD2;DSG2;EFCAB1;ELFN2;EN2;ERO1A;ETF1;EVI5;EYS;FNBP1;FOXJ3;FYCO1;GALP;GCH1;GLO1;GTF2F1;GTF3C4;HIF1A;HNRNPA3;HOXA10;HSD17B12;IGF1R;IL1RAP;INO80C;INSL4;INSL6;ITPR1;KIF2C;KMT2D;LATS2;MAP1LC3B;MAP2K1;MAPK1;MAPKAPK5;MCC;MDM4;MINDY2;MKNK2;MLLT10;MYC;MYH2;NCL;NHS;NPNT;NPTN;PABPN1;PCNP;PDIA3;PEX11B;PITX2;PLEKHA3;PPP1CC;PRKAA2;PROS1;PRRC2B;PTEN;PTPN12;PTPN14;RAB4A;RAD23B;RAP1B;RB1;REST;RGS16;RHOB;RNF115;RPS14;RRAGD;SAMD15;SDC1;SERP1;SHOC2;SLC25A12;SLC26A3;SLC8A1;SMIM13;SPATA13;SS18;SSFA2;SUPT16H;SYNCRIP;TBCA;TEAD1;TFAM;TFPI;TM6SF1;TMEFF2;TMEM178B;TMEM64;TMLHE;TMOD3;TRIM36;UGT2B17;UHMK1;WDR45B;WNT16;ZBED2;ZBTB25;ZC3HAV1L;ZEB1;ZNF24;ZNF532;ZNF543;ZNF620;ZNF736;ZSCAN16
hsa-miR-17-5p-478447_mir	-1.159	0.281	A1CF;AAK1;ABCA1;ABCG8;ABHD15;ABHD18;ABHD2;ABI2;ACADSB;ACAP2;ACBD5;ACER2;ACOT2;ACOT9;ACOX1;ACSL4;ACTR2;ACVR1B;ADAR;ADARB1;ADAT2;ADD1;AFF1;AGFG2;AGMAT;AGO1;AGO3;AK1;AKAP11;AKR7A2;AKTIP;ALDH9A1;AMD1;ANKFY1;ANKH;ANKIB1;ANKRD12;ANKRD13C;ANKRD27;ANKRD33B;ANKRD50;ANKRD52;ANKS4B;AP1G1;APEX1;APOH;APP;ARAP2;ARCN1;ARHGAP1;ARHGAP10;ARHGAP12;ARHGAP35;ARHGAP5;ARHGEF18;ARHGEF7;ARID4B;ARIH1;ARL1;ARL9;ARMT1;ARPC2;ARSJ;ASB1;ASB16;ASH1L;ASNS;ATAD2;ATAT1;ATF3;ATG14;ATG16L1;ATG2A;ATG2B;ATL3;ATP1B3;ATP2B1;ATP5B;ATP6V0E1;ATRX;ATXN1;ATXN7;ATXN7L3B;AZIN1;B2M;BACE1;BAGE5;BAZ2A;BBX;BCAS4;BCL2;B

			CL2L11;BCL2L2;BHMT2;BICD2;BLOC1S3;BLVRA;BMP2;BMP8B;BMPR2;BMT2;BNIP2;BRCA2;BRI3BP;BRMS1L;BSCL2;BTBD7;BTF3L4;BTG2;BTG3;BTN3A1;BTN3A2;BTN3A3;BZW1;C11ORF54;C12ORF65;C14ORF119;C14ORF28;C15ORF40;C15ORF41;C16ORF52;C16ORF70;C17ORF75;C18ORF32;C1ORF50;C2ORF69;C3ORF38;C6ORF120;C7ORF43;C9ORF40;CABLES1;CADM2;CAMK2N2;CAMTA1;CANX;CAP1;CAPN15;CAPRIN2;CAPZA2;CASP2;CAV1;CAVIN1;CBL;CBX1;CBX5;CBX8;CCDC125;CCDC137;CCDC198;CCDC30;CCDC47;CCDC6;CCDC71L;CCL1;CCL5;CCND1;CCND2;CCP110;CCSER2;CCT6A;CD28;CD47;CDIPT;CDKN1A;CDKN2AIPNL;CENPQ;CEP104;CEP170;CEP57;CEP72;CEP97;CERCAM;CETN2;CFL2;CHAF1A;CHD4;CHD9;CHIC1;CHST14;CHSY1;CHTF8;CHURC1;CIT;CKAP2;CLEC12B;CLIC4;CLIP4;CLOCK;CLPTM1;CLU;CMPK1;CMTR2;CNEP1R1;CNKSR3;CNOT4;CNOT6L;CNOT7;CNTN1;COA1;COIL;COPS3;COX19;COX6B1;COX7B;CPE;CPOX;CPS1;CPSF1;CPT1A;CRCP;CREB1;CRISPLD2;CRK;CROT;CRTC3;CRY2;CSDE1;CSNK1A1;CTSA;CTSS;CXORF38;CYB5A;CYBRD1;CYCS;CYCSP5;CYLD;CYP7B1;DAPK3;DCAF1;DCAF8;DCBLD2;DCTN5;DCTN6;DCTPP1;DCUN1D4;DDHD1;DDI2;DDX5;DEGS1;DENND5B;DEPDC1;DHODH;DHX33;DIS3L;DNAJB13;DNAJB4;DNAJB6;DNAJB9;DNAJC10;DNAJC27;DNAJC28;DNAL1;DNM1L;DNMBP;DNMT1;DNTTIP2;DPP9;DPYSL2;DRAXIN;DSPP;DSTYK;DUSP18;DUSP2;DYNC1LI2;DYRK2;E2F1;E2F2;E2F3;E2F5;EARS2;EEA1;EEF1A1;EFCAB11;EFCAB14;EFHC1;EGLN3;EGR2;EHMT2;EIF2B2;EIF2S1;EIF4A2;EIF4G2;EIF4G3;EIF4H;EIF5A2;ELAVL2;ELK4;ELMO2;ELMSAN1;ELOC;ELP2;EMC1;EMSY;ENPP5;ENTPD4;ENTPD7;EPAS1;EPB41L2;EPB41L5;EPHA4;EPS15L1;ERAP1;ERCC2;EREG;ERGIC2;ERLIN1;ESR2;ETF1;ETV1;EXO5;EZH1;F2R;F2RL1;F2RL3;FAAP24;FAF2;FAHD1;FAM102A;FAM117B;FAM126B;FAM129A;FAM160B1;FAM210A;FAM213A;FAM241A;FAM46C;FAM57A;FAM83D;FAM89A;FAM8A1;FANCA;FAS;FASN;FAXC;FBXL5;FBXL7;FBXO10;FBXO21;FBXO28;FBXO31;FBXO48;FCHO2;FEM1A;FEM1B;FEM1C;FER;FEZ2;FGFR1OP;FHDC1;FICD;FJX1;FKBP14;FMNL2;FMNL3;FNBP1L;FOPNL;FOXC1;FOXJ2;FOXJ3;FOXK1;FOXK2;FOXQ1;FOXRED2;FRMD6;FRS2;FTH1;FUT10;FXR2;FXYD5;FYCO1;FZD9;GAB1;GABBR1;GABPAP;GAK;GANAB;GAPDH;GATA6;GATAD1;GBF1;GBP3;GDAP1;GDF11;GDF5OS;GEMIN8;GID4;GIGYF1;GINS4;GLO1;GNAS;GNB1;GNB5;GNPTAB;GNS;GOLGA1;GOLGA2;GPAM;GPI;GPM6A;GPR137B;GPR155;GPR157;GPR183;GPRIN3;GRAMD1A;GRK3;GRK7;GRPEL2;GTF2H2C;GTF2H3;GTF2IRD2;GTF2IRD2B;HAS2;HAUS8;HBP1;HDAC10;HECA;HEXIM1;HIF1A;HIF1AN;HIP1;HIST1H2AM;HIST1H2BD;HIST1H2BG;HIST1H2BJ;HIST1H4C;HIST2H2AA3;HIST2H3A;HIST2H4B;HIST3H2A;HMBOX1;HMGB1;HMGB2;HMGB3;HNRNPR;HNRNPU;HOOK3;HOXD11;HS3ST1;HSP90AA1;HSP90B2P;HSPA4L;HSPA8;HSPB2;HTT;HUWE1;HYPK;ICA1L;ICAM1;ICMT;IER3;IFNAR1;IFNAR2;IGFBP3;IGFBP5;ILF3;IMMT;INPP5F;INSL3;IPP;IQSEC1;IRAK1;IRAK4;ISCA2;ISOC1;ISY1;ITCH;ITGA2;ITGB1;ITGB8;ITPKB;JAK1;JPT1;KANSL1;KAT2A;KAT2B;KATNAL1;KCNA7;KCNB1;KCND3;KCNJ8;KCNK6;KCNMA1;KCNMB1;KCTD7;KDM4A;KDM6B;KIAA0232;KIAA0513;KIAA1147;KIAA1191;KIAA1551;KIAA1841;KIF1A;KIF23;KIF5C;KIF6;KLF10;KLF3;KLF6;KLHL15;KLHL20;KLHL28;KLHL36;KLRD1;KMT2A;KMT2B;KMT5B;KPNA2;KPNA6;KRT10;LAMC1;LAMTOR1;LAPTM4A;LARP1;LAS1L;LASP1;LCOR;LDHD;LDLR;LEPROT;LGSN;LIAS;LIMA1;LIMK1;LINC00598;LLPH;LPAR2;LPGAT1;LPIN1;LRIF1;LRP12;LRPAP1;LRRC58;LRRD1;LSM14A;LSM3;LUZP2;LY6G5B;LYSMD3;LZIC;M6PR;MAK16;MAN2B2;MANEAL;MAP3K12;MAP3K14;MAP3K2;MAP3K3;MAP3K8;MAP3K9;MAP7;MAPK1;MAPK14;MAPK9;MAPKAPK5;MAPRE3;MARCH4;MARCH6;MASTL;MAVS;MBNL1;MCC;MCL1;MDK;MDM2;MECP2;MED12;MED13;MED16;MED17;MED18;MEF2D;MELK;MEN1;METTL8;MFHAS1;MFN1;MFN2;MFSD2A;MFSD4B;MFSD8;MGEA5;MICB;MIDN;MINK1;MINOS1-NBL1;MIXL1;MKI67;MKNK2;MKRN1;MLF2;MLLT1;MLXIP;MMP2;MNT;MOB1B;MORC1;MORF4L1;MORF4L2;MPPE1;MRPL40;MRPS10;MRPS6;MSH3;MSMO1;MT-ATP6;MT-CO2;MT-ND2;MT-ND4;MTF1;MTMR3;MTMR9;MTPAP;MTRF1L;MUC17;MUC21;MXI1;MYC;MYCBP2;MYH9;MYLIP;MYLK3;MYO1D;MYO1F;MYPN;N4BP1;N4BP2L2;NAA50;NABP1;NACC2;NAGK;NAP1L1;NAPEPLD;NARS;NAT8L;NBL1;NBR1;NCAPD2;NCOA3;NEK8;NETO2;NFAT5;NFATC2IP;NFIB;NHLRC3;NIN;NIPA1;NKIRAS1;NME6;NOC2L;NONO;NOTCH2;NPAS2;NPAS3;NPAT;NPNT;NR2C2;NR2F6;NR3C1;NRBP1;NRIP3;NSD2;NUCKS1;NUDT3;NUFIP2;NUGGC;NUP35;NUP98;OCIAD1;OCRL;OFD1;OLAH;OPTN;ORAI1;ORAI2;ORMDL3;OSTM1;OTUD4;OXR1;PAFAH1B1;PAIP1;PAK6;PANK3;PAPD5;PARD3;PARD6B;PBXIP1;PCBP2;PCGF5;PCLAF;PCMTD1;PCNX1;PCNX2;PDE4C;PDGFB;PDHB;PDLIM5;PDLIM7;PDPK1;PDRG1;PDZD11;PEA15;PEAK1;PELI1;PER1;PFKFB2;PFKP;PGAM1;PGM2L1;PHF6;PHLPP1;PHLPP2;PHTF2;PIGO;PIGS;PIK3CA;PIP4K2A;PIP4K2C;PITPNA;PIWIL2;PKD2;PKMYT1;PKNOX1;PLAG1;PLAGL2;PLEKHM1;PLEKHO2;PLRG1;PLS1;PLXNA1;PMAIP1;PNPLA4;PNRC1;POFUT1;POGK;POGZ;POLK;POLM;POLR1B;POLR3A;POLR3F;POLR3G;PPP1CA;PPP1R12B;PPP1R15A;PPP1R15B;PPP1R3B;PPP2R1A;PPP2R2A;PPP3R1;PPP6C;PPP6R3;PRICKLE1;PRICKLE4;PRIM1;PRKACB;PRKAR1A;PRKCB;PRNP;PRPF4;PRPF8;PRR14L;PRRG1;PRRG4;PSD3;PTBP1;PTEN;PTENP1;PTGER4;PTGES3;PTGFRN;PTGIS;PTP4A1;PTPDC1;PTPN4;PTPRO;PTTG1;PUDP;PURB;PVR;PXK;QARS;QKI;QRFPR;QSOX1;RAB10;RAB11FIP1;RAB12;RAB22A;RAB23;RAB30;RAB3IP;RAB42;RAB5B;RABEP1;RABGAP1L;RACGAP1;

			RAD21;RALGAPA1;RAN;RANGAP1;RAP2C;RAPGEF4;RB1;RBBP7;RBL1;RBL2;RBM12B;RBM20;RBM41;RBM5;RCCD1;REEP3;REEP5;REST;REV1;RFK;RFXANK;RFXAP;RGMB;RHOC;RLIM;RMDN1;RMND1;RNASEH1;RND3;RNF115;RNF145;RNF146;RNF19B;RNF216;RNF34;RORA;RPA2;RPF2;RPL14;RPL17;RPL17-C18ORF32;RPL21;RPL37;RPL7;RPRD2;RPS15A;RPS27A;RPS6KA5;RPSA;RRAGD;RRAS2;RRM2;RRN3;RSRP1;RTCA;RTN2;RTN3;RTTN;RUFY2;RUNDC1;RUNX1;RUNX3;RYR2;SACS;SALL3;SAMD12;SAMD8;SAMD9L;SCAMP2;SCAMP5;SCD;SDHA;SEC16A;SEC23A;SELE;SEMA4B;SEMA7A;SENP1;SEPT11;SEPT2;SERF1A;SERF1B;SERF2;SERINC1;SESN1;SESN2;SESN3;SF3B3;SGMS1;SGPL1;SGTB;SH3BP5;SH3GLB1;SH3GLB2;SHOC2;SIK1;SIKE1;SIPA1L3;SIRPA;SKI;SKIL;SLAIN2;SLC12A6;SLC16A2;SLC16A9;SLC1A5;SLC22A23;SLC25A28;SLC25A3;SLC25A33;SLC25A37;SLC25A44;SLC25A46;SLC28A1;SLC30A1;SLC30A7;SLC35E2B;SLC35F5;SLC35F6;SLC4A7;SLC5A3;SLC6A4;SLC7A11;SLCO5A1;SLK;SMAD3;SMAD4;SMAD5;SMAD6;SMIM13;SMIM15;SMOC1;SMURF1;SNAP47;SNTB2;SOCS4;SOCS5;SOCS6;SOD2;SON;SORCS2;SOX4;SP2;SP4;SPCS1;SPIB;SPOPL;SPRED1;SPTLC2;SQSTM1;SRCAP;SREK1IP1;SRSF2;SSH2;SSX2IP;STAC2;STAT3;STIL;STK11;STK11IP;STK17B;STX4;STX6;SUCO;SUGP1;SUOX;SURF4;SUSD6;SYNDIG1;SYNJ2BP;SYNPO2L;TADA2B;TAF9B;TANC1;TAX1BP1;TBC1D15;TBC1D17;TBC1D2;TBL1XR1;TCEA2;TCEAL1;TCF3;TCF4;TCF7L2;TEFM;TET3;TFAM;TGFB1;TGFBR2;TGOLN2;THBS1;THEM4;TIMM17A;TIMP3;TJP1;TLR7;TM4SF5;TMBIM6;TMED10;TMEM100;TMEM123;TMEM127;TMEM131L;TMEM133;TMEM134;TMEM138;TMEM165;TMEM167A;TMEM19;TMEM196;TMEM200C;TMEM242;TMEM245;TMEM267;TMEM38A;TMEM64;TMEM67;TMEM9;TMEM9B;TMOD3;TMSB10;TMTC4;TMX3;TNF;TNFAIP1;TNFAIP8L1;TNFRSF10B;TNFRSF21;TNFSF12;TNIP3;TNKS2;TNPO3;TNRC6A;TNRC6B;TOB1;TOPORS;TP53;TP53COR1;TP53INP1;TPK1;TPM4;TPRG1L;TRA2B;TRAF3IP2;TRAPPC10;TRAPPC2;TRIM11;TRIM32;TRIM37;TRIM44;TRIM65;TRIM71;TRIM8;TRIOBP;TRIP10;TRRAP;TRUB1;TSC2;TSG101;TSKU;TSPAN6;TSR1;TTC9;TTPAL;TUBB4B;TUT1;TWF1;TXK;TXLNA;TXNIP;U2SURP;UBC;UBE2C;UBE2Q2;UBE2S;UBE2V2;UBE3C;UBE4A;UBFD1;UBOX5;UBR5;UBXN2A;UGCG;ULK1;UNK;UQCRFS1;USP16;USP28;USP3;USP32;USP38;USP48;UXS1;VCPKMT;VDAC1;VEGFA;VLDLR;VPS13C;VPS26A;VPS50;VPS53;VTI1A;WAC;WASL;WDR1;WDR37;WDR53;WDR73;WDR82;WDR89;WDR92;WEE1;WIPF2;WNK3;WSB1;WWC1;XIAP;XIRP2;YES1;YIPF4;YOD1;YTHDC1;YWHAZ;ZBED1;ZBED3;ZBTB18;ZBTB25;ZBTB33;ZBTB37;ZBTB4;ZBTB5;ZBTB6;ZBTB7A;ZBTB9;ZC3H12C;ZC3H18;ZDHHC20;ZFYVE21;ZFYVE26;ZFYVE9;ZIK1;ZMAT3;ZMYM1;ZNF107;ZNF12;ZNF174;ZNF180;ZNF202;ZNF264;ZNF280B;ZNF280C;ZNF347;ZNF35;ZNF354B;ZNF385A;ZNF417;ZNF426;ZNF446;ZNF454;ZNF507;ZNF514;ZNF532;ZNF578;ZNF597;ZNF598;ZNF652;ZNF665;ZNF681;ZNF682;ZNF689;ZNF7;ZNF70;ZNF770;ZNF780A;ZNF785;ZNF786;ZNF800;ZNF805;ZNF93;ZNFX1;ZRANB1;ZSWIM3;ZYG11A
hsa-miR-376c-3p-478459_mir	2.416	0.286	ACVR1C;ADH4;ANK1;ANKRD36;ARF6;ARHGAP42;ATAT1;ATM;BBS4;BCL11A;BCL2;BMI1;BZW1;CBX4;CD59;CDK14;CENPA;CHEK2;CLDN1;CYTOR;DAPK1;DFFA;DNAJB14;ELF4;ENOX2;ENPP5;ERO1B;FAM8A1;FNBP1L;FOXJ3;FRS2;FXR2;FZD6;G3BP1;GOLGA7B;GRB2;IGF1R;JADE3;LSM14A;MC2R;MDM2;METTL22;MORF4L2;MTRNR2L1;MTRNR2L2;MTRNR2L6;MTRNR2L8;NEDD4L;NR5A2;ORC4;PDPK1;PRKCH;PRPS1L1;PRRG3;PSPC1;RAB1A;RBM20;RPS6KA5;RTN4;RUNX2;SECISBP2L;SIAH2;SKIL;SNCB;SNRNP27;SP4;SYF2;TAT;TGFA;TGFBR1;TIMM8A;TMEM106B;TMEM170B;TNFRSF10B;TSPAN6;TVP23C;UBE2D3;UGT2B15;UGT2B17;VLDLR;WNT10B;YTHDF1;ZNF829;ZNFX1
hsa-miR-24-3p-477992_mir	-1.580	0.290	AAK1;AAMP;ABCB10;ABCB9;ABCE1;ABHD2;ACAA2;ACACA;ACD;ACOX1;ACP6;ACTL6A;ACVR1B;ADAM17;ADD1;ADD3;ADPGK;ADRM1;AEN;AGFG1;AGO3;AGPAT2;AGPAT3;AK3;AK4;AKAP5;AKAP7;AKIP1;ALDH5A1;ALDH6A1;ALDOA;ALG1;ALG5;ALOX5AP;AMBRA1;AMOTL2;AMPD2;ANAPC16;ANKRD9;ANPEP;AP5B1;AP5M1;APAF1;APOBEC3C;APOBEC3F;APOL2;APOOL;APPL1;APTX;ARHGAP19;ARHGAP39;ARHGEF18;ARHGEF7;ARL1;ARL17B;ARSK;ASB6;ATAD3A;ATAD3C;ATF3;ATG4A;ATL3;ATP1B4;ATP5B;ATP6V0A2;ATP6V0E1;ATP6V1A;AUNIP;AURKA;AURKB;AVL9;B3GNT2;BAZ2B;BBC3;BCAR1;BCL2L11;BCL2L12;BCL2L2;BCL7A;BEX1;BMP7;BRCA1;BRD8;BRMS1L;BTBD3;BVES;BZW1;C11ORF84;C12ORF49;C15ORF39;C17ORF49;C17ORF75;C1QTNF6;C8ORF33;C8ORF58;CAPN15;CARD10;CASP10;CBY3;CCAR1;CCAR2;CCDC117;CCDC122;CCDC32;CCDC58;CCDC59;CCDC69;CCL16;CCL2;CCL3;CCL4;CCNA2;CCNB1;CCND1;CCNG1;CD34;CD3D;CDC73;CDCA7;CDH7;CDK1;CDK4;CDKAL1;CDKN1B;CDKN2A;CEP57L1;CHD8;CHEK1;CHFR;CINP;CIRBP;CISD2;CLEC7A;CLN8;CLSTN1;CMTM3;CMTM4;CNDP2;CNKSR3;CNNM3;COL8A1;COMMD9;COPS5;COPS7A;CORO1A;COX20;COX6B1;CPE;CPSF2;CPT1A;CRCP;CRIPT;CRISPLD2;CRTAP;CRX;CRY2;CSK;CSTF1;CSTF3;CTCF;CTDSP1;CTDSP2;CWC27;CYP11B2;CYP20A1;CYP51A1;DARS;DARS2;DBF4;DBNDD1;DBR1;DCAF10;DCAF4;DCAF7;DCP1A;DCP2;DDHD2;DDN;DDX5;DEDD;DENND6B;DEPDC1;DHCR24;DHFR;DHFRP1;DNA2;DNAJB12;DNAJB13;DNAJC10;DNAJC3;DND1;DNPEP;DRAXIN;DSC2;DSN1;DTD2;DTL;DUS1L;DUSP19;DUSP7;DVL3;DYNLL2;DYRK2;E2F1;E2F2;E2F3;EEF1A1;EHD2;EIF1AD;EIF2S3;EIF4G3;EIF4H;EIF5;ELP1;ELP2;EMC2;EMC3;EMC7;EMP1;EMP2;EPB41;EPG5;ERBB3;EREG;ERGIC1;ERO1A;ESCO1;ESF1;ESYT2;EXOSC1;EXOSC2;EXOSC8;FAF1;FAH;FAM177A1;FAM210B;FAM217B;FAM234A;FAM241A

			;FANCF;FBLIM1;FBXO34;FBXO36;FBXW7;FEN1;FGF11;FGFR3;FKBP1B;FLCN;FNTB;FOSL2;FOXQ1;FPR1;FSCN1;FURIN;FZD4;FZD5;GABRB3;GATA3;GATA6;GBA2;GCLM;GFOD1;GGA2;GGCX;GIGYF1;GINM1;GJD3;GLRX2;GLUL;GLYR1;GMEB1;GMFB;GMPS;GNPAT;GORASP2;GPAT4;GPC4;GPR55;GPR82;GREB1;GRINA;GRK4;GRWD1;GSDMA;GSTO2;GTF2E1;GTF3C2;GTF3C6;GUCD1;GYG2;H2AFX;HACD3;HACD4;HBQ1;HDAC1;HDDC2;HECTD3;HEXA;HEYL;HIC2;HINT1;HIST1H2BJ;HIST3H2BB;HKR1;HM13;HMGB2;HMOX1;HNF4A;HNRNPA1;HOOK3;HSD17B12;HSF2;HUS1;IDS;IER5;IFNG;IFNR;IGF1;IGF2R;IGSF6;IKZF3;IL17REL;IL18;IL1B;IL4;IMP4;INAVA;ING1;INMT;INSIG1;INSL6;IQCB1;IRAK3;ITGB3;JADE3;JARID2;JPH2;KAZALD1;KCNE4;KCNJ14;KCNJ5;KCNK2;KCNK5;KHNYN;KHSRP;KIAA0100;KIAA0754;KIAA1210;KIAA1456;KIF18B;KIF1C;KIF3A;KLF17;KLHDC3;KLHL15;KLHL23;KLLN;KPNA6;LAMTOR3;LAPTM4B;LAX1;LBR;LDHA;LDHB;LEPROTL1;LIAS;LIMD1;LIMS1;LINC00346;LINC00632;LIPG;LLGL1;LMNB2;LONRF2;LRIG2;LRRC10;LRRC58;LRRTM2;LSM12;LSM14A;LY6G5B;LYRM4;MAFB;MAK16;MALL;MALSU1;MAP2K7;MAP3K9;MAPK14;MAPK7;MAPKAPK5;MARCKSL1;MATR3;MBD6;MBOAT1;MCM10;MCM4;MCM9;MDM4;MED16;MED17;MED22;MED24;MED28;MEN1;MESD;METAP2;MFSD14C;MIDN;MIEF2;MINOS1;MIS18A;MLEC;MMP14;MMS19;MOB1A;MOB3A;MOCS3;MPIG6B;MRO;MRPL27;MRPL40;MRPS16;MRPS22;MRPS24;MSANTD4;MT1E;MT1M;MTF2;MTHFD1L;MXI1;MYC;MYH9;MYPN;NAE1;NARF;NARS;NASP;NAV1;NCAN;NCBP2;NCOA5;NCSTN;NDRG1;NDST1;NDUFA4P1;NDUFA7;NECAB3;NEDD4L;NEGR1;NEK6;NEMP1;NET1;NETO2;NFAT5;NFE2L1;NFKBIA;NFX1;NHS;NICN1;NIPA1;NIPAL1;NIPSNAP2;NKD1;NOL9;NOP14;NOP56;NOS3;NOTCH1;NOTUM;NR0B2;NRIP1;NT5C1A;NTPCR;NUBPL;NUDCD2;NUDT7;NUP43;NUP54;NXPE2;OARD1;OGFR;OLR1;OR7D2;OSBPL10;OSBPL2;OSMR;OTUD7B;OXSR1;PA2G4;PACS2;PACSIN3;PAF1;PAFAH1B2;PAK4;PAPOLA;PARP2;PCDHB11;PCDHB2;PCGF6;PCK2;PCLAF;PCNA;PCNP;PCYOX1;PDE11A;PDE12;PDE6B;PDE7A;PDF;PDGFRB;PDLIM5;PDLIM7;PDPK1;PDXK;PER2;PEX2;PGBD5;PHAX;PHLDA3;PHOSPHO2;PI4K2B;PIGR;PIM2;PKMYT1;PLA2G4A;PLAGL2;PLEKHA2;PLEKHH1;PLEKHS1;PLIN3;PLIN5;PMPCA;POGZ;POLA2;POLD1;POLL;POLR1B;POLR2D;POLR3D;POPDC2;POU2F3;PPM1D;PPM1F;PPP3R1;PPTC7;PRDX6;PRICKLE1;PRIM1;PRKCA;PRKCH;PROSER1;PRPF6;PRR11;PRR13;PRRG4;PRSS8;PSAP;PSMD1;PSME3;PSTPIP2;PTAFR;PTDSS2;PTENP1;PTGFRN;PTGIS;PTPN9;PTPRF;PUM3;QPCTL;R3HDM2;R3HDM4;RAB11FIP1;RAB5C;RABAC1;RAD54L2;RAET1E;RALA;RANBP1;RAP2B;RAP2C;RASGRP3;RASSF4;RASSF9;RBBP4;RBBP9;RBL1;RBM22;RBM3;RBM48;RBMS2;RCE1;RDH10;REG4;REPS2;RFTN2;RFWD2;RHOF;RHOT2;RILPL1;RMDN1;RNF11;RNF144A;RNF2;RNF24;RPL37A;RPL7L1;RPRD2;RPS16;RPS6KA5;RPS7;RRAS;RRM2;RRP12;S100A16;S100A8;S100P;S1PR2;SAMD5;SCAMP2;SCD5;SCML1;SCML2;SCUBE3;SELPLG;SEPHS1;SERGEF;SESN1;SETD5;SETD7;SETX;SFT2D2;SGPL1;SGTB;SH3PXD2A;SHISA2;SHOC2;SHROOM3;SIK2;SIT1;SLC11A2;SLC16A13;SLC19A3;SLC1A2;SLC1A5;SLC25A15;SLC29A4;SLC2A3;SLC33A1;SLC35B2;SLC35E2B;SLC52A2;SLC5A6;SLC6A4;SLC7A1;SLC7A2;SLC9A7;SLIT3;SLU7;SMTNL2;SMYD4;SNAP29;SNRPB2;SNRPD1;SNRPD3;SNTB1;SNTN;SNX1;SNX12;SORD;SP1;SPATA5;SPIN4;SPPL2A;SPTLC3;SRCIN1;SRP19;SRRD;SRRT;SS18;SSR1;SSR3;SSSCA1;ST7L;STEAP3;STIL;STK35;STK4;STRADB;STRN3;STX16;STX4;SUGT1;SULT2A1;SUMO1;SUMO3;SVIP;SYNRG;TAB1;TACC3;TAF1;TAF15;TAF1B;TAF8;TAOK1;TBC1D14;TBCCD1;TBPL1;TCEA3;TCF23;TDRP;TESMIN;TFDP2;TGFB1;THAP12;THEM4;THOP1;TIAL1;TLN1;TM9SF3;TMED7;TMEM105;TMEM173;TMEM19;TMEM192;TMEM209;TMEM216;TMEM236;TMEM239;TMEM241;TMEM33;TMEM41B;TMEM70;TMEM92;TMEM94;TMTC4;TNF;TNFAIP3;TNFRSF13B;TNFSF15;TNIP2;TNK2;TNPO3;TOMM22;TOMM34;TOMM40;TOP1;TOR1AIP2;TOR2A;TP53;TRAF3IP1;TRAF3IP2;TRIB3;TRIM11;TRPM6;TRUB2;TSC22D2;TSPAN14;TTC9C;TTLL7;TUBGCP2;TWSG1;TXNDC16;TYMSOS;TYW3;UBC;UBD;UBE2C;UBE2K;UBE3A;UCK2;UGCG;UGDH;UHRF1BP1L;ULBP3;ULK2;UNG;UQCC1;URM1;USP10;VCPIP1;VGLL3;VHL;VPS25;VPS35;VPS8;VRK1;WASF2;WDFY2;WDR17;WDR55;WIZ;WNT4;WRB;WWTR1;XIAP;XKR4;XPNPEP3;XRCC2;YES1;YME1L1;YOD1;YPEL1;YRDC;YTHDC1;YWHAZ;ZBED1;ZBTB16;ZBTB3;ZBTB8B;ZC3H12B;ZC3H15;ZCCHC14;ZDHHC20;ZFP30;ZFYVE9;ZKSCAN1;ZKSCAN3;ZMAT2;ZMYM1;ZMYND19;ZNF106;ZNF107;ZNF124;ZNF17;ZNF217;ZNF260;ZNF264;ZNF281;ZNF284;ZNF286B;ZNF317;ZNF430;ZNF451;ZNF490;ZNF516;ZNF556;ZNF566;ZNF581;ZNF621;ZNF623;ZNF70;ZNF708;ZNF730;ZNF783;ZNF786;ZNF8;ZNF813;ZNF878;ZSCAN22;ZSCAN29;ZXDA;ZXDB
hsa-let-7f-5p-478578_mir	3.319	0.291	ABHD17C;ABT1;ACER2;ACOT9;ACTA1;ACTR10;ADH5;ADIPOR2;AGO1;AHCYL2;AHR;AK4;AKAP8;ALDH7A1;AMD1;ANKRD46;ANP32C;AP1S1;AQP6;AREL1;ARID3A;ARID3B;ARIH1;ARL6IP1;ARL8B;ARMC8;ASCC3;ASTE1;ATG12;ATG9A;ATL1;ATP2A2;ATP5B;ATP6V1F;ATP6V1G1;ATR;ATXN2;ATXN2L;ATXN7L3;ATXN7L3B;B4GAT1;BACH1;BAZ1B;BCOR;BEND4;BRI3BP;BZW1;C11ORF57;C12ORF4;C19ORF47;C19ORF53;C1ORF21;C1ORF210;C1RL;C5ORF51;CALM1;CALU;CAPRIN2;CASTOR2;CBX5;CCL7;CCNB2;CCND1;CCNG1;CCNT2;CD59;CDKAL1;CDKN1A;CDV3;CELF1;CEP120;CEP135;CEP170B;CHTOP;CLDN12;CNEP1R1;CNOT8;COIL;COL8A1;COLEC12;COPS6;COPS8;COX6B1;CPA4;CRX;CRY2;CTPS1;CXCL8;CYP19A1;DIABLO;DISC1;DNA2;DNAH9;DNAJC28;DNAL1;DTX3L;DUSP1;DVL3;DYNC1LI2;DYRK2;DYRK3;E2F6;ECHDC1;EDA;EDEM3;EDN1;EFHD2;EIF3C;EIF4A3;EIF4G2;ELF4;ELOA;EMILIN2;

			EPHA4;ERO1A;ESPL1;FAM104A;FAM105A;FAM222B;FAM43A;FAM83G;FARP1;FBXL20;FBXW2;FIGN;FMNL3;FMO4;FNDC3A;FNDC9;FPR1;FUT10;FXN;FZD4;FZD9;GABPAP;GALC;GATM;GBF1;GGA3;GLO1;GLUL;GNG5;GOLGA4;GPAT4;GPS1;GRPEL2;GTF2E1;HAND1;HASPIN;HDAC2;HERPUD1;HIST1H2BD;HIST1H2BK;HMGA1;HMGA2;HNRNPA2B1;HNRNPUL1;ICOSLG;IFNLR1;IGDCC4;IGF1R;IGF2BP1;IGF2BP3;IKZF3;IL13;IL6;IL6R;INTS7;IPO9;ITGA3;KCTD21;KIAA0391;KIAA0930;KIAA1143;KIAA1328;KIF27;KLHDC8B;KLK10;KLK6;KMT2D;KPNA5;KREMEN1;LARP1;LEFTY1;LIMD2;LRIG3;LRRC20;LYN;MAGEA12;MAGEA3;MAGEA6;MAP2K7;MAPK6;MARCKSL1;MARS2;MBD2;MCF2L2;MDM4;MEF2D;MEIS3P1;MFSD8;MIDN;MIEF1;MLLT10;MPL;MRPL12;MSI2;MSL3;MT-ND4;MT-ND5;MTSS1L;MTUS1;MTX3;MXD1;MYC;MYH9;NAA20;NAA30;NAP1L1;NARS;NAT8L;NCKIPSD;NCOA3;NDUFA4P1;NHLRC2;NHLRC3;NKX2-1;NOA1;NOM1;NR6A1;NSD1;NUCB2;NUDT4;NUP155;ONECUT2;OPA3;OPRL1;PAFAH2;PARP16;PBX2;PCGF3;PDE12;PDGFB;PDLIM5;PDP2;PDZD8;PEG10;PEX11B;PGM2L1;PGRMC1;PHACTR4;PLAGL2;PLCG2;PLD3;PLEKHA3;PLEKHO1;PLXND1;PM20D2;PMAIP1;PMPCA;POLL;POLR2D;POLR3D;POSTN;POTEG;POTEM;PPP1R15B;PPP2R2A;PRDM1;PRIM2;PRR5-ARHGAP8;PRSS22;QDPR;RAB11FIP4;RAB19;RAB40C;RABEP1;RABL2A;RABL2B;RAD18;RBFOX2;RBM12B;RDX;RFC2;RHBDF2;RHD;RNF144B;RNF44;RNFT1;ROR2;RRAD;RRM1;RRM2;RWDD1;SALL3;SAR1A;SART3;SDR42E1;SEMA4C;SF3A1;SLC10A7;SLC11A2;SLC12A7;SLC16A9;SLC19A3;SLC20A1;SLC25A12;SLC33A1;SLC38A7;SLC5A5;SLC5A6;SMARCAD1;SMARCC1;SMC1A;SMCR8;SNRPC;SNX17;SOCS1;SOCS3;SOCS4;SOD2;SP1;SPTBN5;STAT2;STK4;STRN;STX3;SUMO1;SUOX;SURF4;SYNJ2BP;SYT1;TAB2;TBC1D19;TG;TGFBR1;TGFBR3;TGOLN2;THBS1;THEM6;THOC5;THYN1;TIAF1;TMED4;TMED5;TMTC3;TNFSF9;TOMM40L;TRAPPC10;TRIM71;TRMO;TSC22D2;TSPAN3;TUBB2A;TUBB4A;TXLNA;TXLNG;UBAP2;UBAP2L;UBXN2B;USP2;USP38;USP45;USP47;VCL;WASL;YAE1D1;YARS2;YOD1;YWHAQ;YWHAZ;ZBTB37;ZBTB5;ZBTB8OS;ZC3H18;ZC3HAV1L;ZCCHC3;ZFAND4;ZNF200;ZNF264;ZNF28;ZNF280B;ZNF417;ZNF443;ZNF460;ZNF556;ZNF566;ZNF578;ZNF584;ZNF587;ZNF609;ZNF611;ZNF644;ZNF738;ZNF774;ZNF799;ZNF8
hsa-miR-134-5p-477901_mir	-2.314	0.294	A1CF;ABCC1;ADAT1;AKAP17A;AKR7A2;ALPI;ANGPTL4;AP5M1;ARHGAP18;ATXN7L3B;BBS1;BTNL3;CA12;CCNG1;CERS4;CMTM6;COX8A;DCP2;EGLN1;EIF4EBP2;ELFN2;ERBB2;ERGIC1;F8A2;F8A3;FAM126B;FAM129B;FEM1A;FIBCD1;FMNL2;FMO4;FOXM1;GABRB2;GNL1;GOLPH3;HIVEP3;HSPD1;IL17REL;IL6ST;INTS7;ISCU;ITGB1;KAT6B;KCTD5;KIAA0040;KMT2D;KRAS;LHFPL2;LRIG2;MAGI2;MAPKAPK5;MED28;MKNK2;MNT;MRNIP;NANOG;NPM1;OPRM1;PAK2;PEX26;PIGP;PLAGL2;POLA2;POLR2F;POT1;POTED;PUM2;RAB27A;RBBP5;RCAN1;RFC5;SERTAD2;SH2D5;SLC25A45;SLC6A6;SMAD6;SMOC1;SMOC2;SOX17;SRSF2;SSX5;STAT5B;SURF4;TJAP1;TMEM101;TP53INP1;TRMO;TSC22D2;TULP1;TULP4;TXNIP;UPF3A;USP13;USP37;USP48;VEGFA;VIM;ZDHHC9;ZNF106;ZNF440;ZNF621
hsa-miR-363-3p-478060_mir	3.921	0.296	AAED1;ABCF2;ACOD1;ACTC1;ADAM15;AEN;AGBL5;AGMAT;AKAP10;ALG14;AMD1;ANP32E;AP3S2;AP5Z1;APOBEC3F;ARF1;ARGFX;ARID1B;ARNTL2;ASGR2;ATF7IP;ATOX1;AURKA;B4GALT7;BAK1;BAZ2B;BCAT1;BCL11B;BCL2L11;BMP8A;BMPR1A;BPTF;BTG2;C11ORF24;C15ORF38-AP3S2;C17ORF75;C1GALT1C1;C1ORF35;C5ORF24;CAPZB;CARD6;CASKIN1;CASP3;CCDC113;CCDC171;CCNB1;CCSER2;CD180;CD226;CDC5L;CDK5R1;CDKN1A;CHST1;CIC;CIDEC;CLN8;CLTA;CNEP1R1;CNIH1;CNNM4;CNOT2;CNOT4;COX20;CPEB3;CPEB4;CREB1;CTDSPL;CYP20A1;CYP2C19;CYTH2;DAND5;DDI2;DDIT4;DDX3X;DENND2C;DNAJB9;DUS2;DUSP5;DYNC1LI2;EDEM1;EFTUD2;EID2B;EIF1;EIF2S2;EIF4EBP1;EIF4EBP2;EIF5A2;ELOA;ENTHD1;ERGIC2;ESRP1;EXOC5;F11R;FAM129A;FAM49A;FASLG;FBXW2;FBXW7;FGF2;FKBP1A;FKBP9;FLCN;FMN1;FNIP1;FOPNL;FOXN2;FUT11;G2E3;GALNT7;GAP43;GATA6;GATAD2B;GCNT3;GEMIN2;GFPT2;GGCX;GID4;GM2A;GNAQ;GOLGA3;GOLGA8A;GOLGA8B;GOLGA8IP;GOLGA8J;GPBP1L1;GRAMD4;GTF2A1;GTF2E1;GUF1;GULP1;H3F3B;H3F3C;HIVEP1;HMGA2;HOXA13;HOXC8;HP1BP3;IFITM1;IL6ST;INCENP;INSIG1;IPP;IRGQ;ITGB8;ITPR1;KCNC4;KIAA1109;KIAA1586;KIAA1958;KIF5B;KLHDC10;KLHL15;LAX1;LCOR;LETM1;LHFPL2;LILRA2;LONRF3;MAP1B;MAP2K4;MCF2L2;MCOLN2;MDM2;MED19;MED29;MED7;MFF;MKNK2;MOAP1;MRO;MRPS16;MRPS21;MTMR10;MUC21;MYLIP;MYO1B;MYZAP;NARF;NCAPG2;NF2;NFYB;NKAP;NLRP9;NOTCH1;NPY4R;NRAS;NUCKS1;NUFIP2;NUGGC;NUP43;OPA3;OR2A4;ORAI2;OSMR;OTUD7B;PAIP1;PAPD7;PARD6B;PAWR;PAX9;PCBD2;PCMTD1;PDPN;PDZD8;PELP1;PER2;PGAM4;PGPEP1;PHLPP2;PIK3CD;PIP5K1C;PKNOX1;PLEKHA1;PLXNA3;PMEPA1;PNO1;POLK;PPIC;PPP1R37;PPP1R3D;PRPS1;PRRC2B;PRRG4;PTAR1;PTGES2;PURG;RAB3D;RAD51;RANBP6;RBFOX2;RBM27;RBM28;RBMS2;RBPJ;REG4;REL;REV3L;RLIM;RNF4;RNF44;RPL23;RPL24;RPL9;RRN3;RSBN1;S1PR1;SASH1;SERTAD3;SESN3;SH2B3;SHE;SLC12A5;SLC25A32;SLC25A36;SLC33A1;SLC39A14;SLC7A11;SLX4;SMARCA5;SMU1;SNRPD1;SNRPD3;SOX11;SOX4;SPATS2L;SPCS3;SPRYD4;SRFBP1;SSFA2;STAT2;SUPT7L;SZRD1;TAF8;TANK;TATDN3;TBC1D8;TEF;TIRAP;TLR3;TMEM184B;TMEM239;TMEM33;TMEM41A;TMEM44;TMF1;TNFRSF13C;TOB1;TOR1B;TOR4A;TPPP;TRAM2;TRIM36;TRMT2B;TSPAN31;TULP4;TWF1;TXLNA;TXNDC15;UBE2Z;UBXN4;UCK2;UGDH;U

			HRF1BP1;UQCRFS1;USP28;VHLL;VMA21;VPS4B;WASL;WDR81;XKR7;YIPF4;ZADH2;ZBTB34;ZBTB8B;ZC3HAV1L;ZDHHC21;ZDHHC24;ZDHHC5;ZFC3H1;ZFP62;ZFYVE21;ZIC5;ZNF134;ZNF157;ZNF24;ZNF264;ZNF267;ZNF277;ZNF317;ZNF354B;ZNF383;ZNF417;ZNF460;ZNF598;ZNF607;ZNF695;ZNF75A;ZNF772;ZNF850;ZNRF3;ZSCAN12
hsa-miR-148b-3p-477824_mir	-1.815	0.304	ABCA13;ABCB7;ABLIM1;ACACB;ACVR1;ADAM33;ADAMTS4;ADAMTS6;ADGRL2;AGGF1;AGO1;AGO2;AGO3;AGTR2;AHI1;AKAP11;AKAP17A;ALCAM;ALDH3B2;ALPI;ANKUB1;AP4E1;AP5B1;APBA2;APBB1;APBB2;APLN;APLP2;ARID3A;ARL6IP1;ARL8B;ARMT1;ASB13;ASB6;ATP10B;ATP6AP2;ATP7A;AVPI1;B4GALT7;BAX;BAZ2B;BCL11A;BIK;BMP3;BPGM;BTBD3;BTBD7;BTNL3;C12ORF40;C17ORF77;C1GALT1;C3ORF58;CA10;CALN1;CAPN13;CCDC197;CCDC92;CCKBR;CCL11;CCL17;CCL19;CCL28;CCNA2;CCNC;CCT6A;CD300A;CDH20;CDK19;CDKN1A;CDKN1B;CEBPG;CEP55;CHRFAM7A;CIDEA;CKLF;CLCN3;CLSPN;CLUAP1;CNOT4;COLEC12;COX7A2;CPA3;CPNE4;CPNE5;CRISP1;CSF1;CSNK2A1;CSTF2T;CTHRC1;CUL5;CXCL5;CYB5R4;CYBB;DCK;DCP2;DCUN1D3;DDX6;DICER1;DLG2;DLX6;DMRT2;DNAH3;DNAJB4;DNMT1;DPT;DSG1;DTX4;EML4;ENPP6;EOGT;EPB41;ERICH5;ETV7;EYA4;FAM104A;FAM212B;FAM81A;FBXO28;FBXW11;FCAR;FETUB;FLOT2;FOXN4;FOXP1;FRY;FSCB;FURIN;FXR1;FXYD2;FYB2;FZD5;GADD45A;GAS1;GDF9;GDNF;GFRA1;GLI3;GLRX5;GMPPB;GNAI2;GPHB5;GPR143;GPRC5A;GSTCD;GTSF1L;H2AFY;H2AFZ;HAVCR1;HCCS;HEMGN;HEY2;HIF1AN;HLA-A;HLA-C;HLA-G;HMGB1;HMGB3;HMOX1;HOTAIR;HRH4;HSP90AA1;HSP90B1;IGFBP5;IGFBPL1;IGSF1;IGSF10;IL13RA2;IL21R;IL23R;INHBA;IQCG;ITGA5;ITGB8;ITSN2;IZUMO2;JARID2;JMJD4;KCNJ16;KCNJ6;KCNQ2;KCTD14;KDM1B;KDM6B;KHDRBS2;KIAA0907;KIAA1456;KIAA2013;KIF2C;KLF6;KLHL12;KLRC1;KLRK1;KPNA4;LACRT;LAMC2;LARP4;LCA5;LCN9;LDLR;LEPROTL1;LHCGR;LIPC;LNPEP;LOXL1;LPL;LRP4;LYSMD1;MAP3K9;MARCH2;MAST1;MATN3;MAZ;MCFD2;MCM10;MECP2;MEP1B;MKX;MLEC;MLH1;MORF4L1;MRPL28;MRPS27;MT-ND2;MT4;MTMR10;MTX3;MUC13;MYF5;MYO3A;MYOM1;NCKIPSD;NCS1;NDN;NDRG1;NEURL4;NIN;NLGN4X;NME5;NOL10;NONO;NOX4;NPEPL1;NPTX1;NR3C2;NRAS;NTN1;NXPE3;OR1A1;OSBP2;OSBPL11;OTUD4;OVOL1;PAPD4;PATL1;PCDHA4;PCNX1;PDIK1L;PDSS1;PDZK1;PHACTR2;PHLDB2;PIH1D3;PIK3CA;PIK3CG;PIK3R3;PKP1;PLA2G12A;PLPP4;PLXDC2;PNLIPRP2;PNPLA2;POC1A;POU4F3;PPARD;PPP1R3A;PPP2R3A;PPP6R1;PRKAA1;PRKAB1;PRKD3;PRNP;PROKR2;PROX1;PSG2;PSG9;PTGER2;RAB12;RAB14;RAB1B;RAB34;RAB3B;RALY;RAPH1;RASSF6;RBM23;RBM38;RBM8A;RCC2;RFT1;RFX3;RGMA;RHAG;RHOF;RHOV;RNF150;RNF219;RNF7;ROCK1;ROPN1L;RORC;RPIA;RPS6KA4;RTL3;RTN4;RUNX3;S1PR2;SARAF;SECISBP2L;SERAC1;SESN3;SESTD1;SH3PXD2A;SIK1;SIRT4;SLC12A7;SLC22A3;SLC22A5;SLC25A18;SLC2A1;SLC32A1;SLC38A2;SLC39A11;SLC6A19;SLC7A11;SLCO4C1;SMARCA1;SNAI2;SNAPIN;SNRPD1;SORD;SOS2;SOX11;SPINT4;SPZ1;SRSF10;SSR3;ST6GALNAC5;STARD13;STEAP4;STON2;STS;STX16;STX6;SUMF1;SUMF2;SYNCRIP;SYNE1;SYNE2;TACC1;TBL1XR1;TCF7L2;TEX13A;TGM7;TKTL2;TMEM14A;TMEM246;TMEM266;TMEM47;TMPRSS11A;TNRC6A;TNRC6B;TOM1L2;TOMM70;TPGS2;TPTE2;TRDN;TTLL1;TUBA8;TUBAL3;TUBG1;TXNIP;UBE2D3;UGT8;UHMK1;USP33;USP48;UTS2;VGLL2;VPS37A;VPS37B;WASL;WNT1;WNT2B;YPEL1;YPEL4;YWHAB;ZCCHC2;ZDHHC15;ZDHHC17;ZDHHC6;ZFYVE26;ZIC5;ZPLD1
hsa-miR-222-3p-477982_mir	-1.787	0.308	ABCG2;ABLIM1;ACACA;ACTB;ACTG1;ADAM1A;AGO2;AHSA1;AKT3;ALG3;AP1B1;APP;ARHGAP42;ARID1A;ARL6IP1;ATF5;ATP11B;ATP7B;ATXN7L3;AUTS2;B3GALNT2;BAG6;BBC3;BCL2L11;BMF;BPTF;BRWD1;C11ORF57;C12ORF65;C18ORF25;C19ORF12;C5ORF51;C8ORF33;CABYR;CANX;CAPRIN1;CASC3;CASKIN2;CAST;CBX2;CCDC47;CDC27;CDK18;CDK6;CDKL5;CDKN1B;CDKN1C;CEP250;CERS2;CHORDC1;CIAPIN1;CKAP2;CLDN23;COG5;COIL;COL5A2;CORO1A;CPSF6;CSAG1;CSNK1G1;CSTF2T;DAG1;DARS2;DAXX;DAZAP1;DBN1;DBNL;DCAF7;DDX21;DDX6;DERA;DERL2;DICER1;DIRAS3;DKK2;DMWD;DNAJB14;DOCK5;DPP8;DYRK3;EEF2;EIF2S1;EIF2S3;EIF3B;EIF3I;ELOB;ENO1;EPB41L2;ERCC6L;ESR1;ETS1;EXO5;EXOC8;EXOSC1;EXOSC10;EZR;FAM126B;FAM214A;FAM35A;FAM53C;FAM83G;FAM84A;FASN;FAT1;FBXL18;FBXO21;FBXW2;FICD;FLNA;FOS;FOXO1;FOXO3;FPGS;FUBP1;FYTTD1;GALNT3;GANAB;GAS5;GCN1;GDI1;GDI2;GJA1;GLO1;GLRX5;GNAI2;GNAI3;GPR107;GRB10;GTF3C5;HAUS8;HCFC1;HDGF;HDLBP;HERC4;HES1;HEXIM1;HIPK2;HIST2H4B;HMBOX1;HMGA1;HNRNPD;HNRNPH1;HOXA13;HSP90AA1;HSP90B2P;HSPA14;IARS;ICAM1;ILK;INPP4B;IPO5;IPP;IRAK1;IRF2BP2;IRS4;ISG20L2;JTB;KIF3B;KIF4A;KIF5C;KIT;KITLG;KLHDC10;KLHL8;KMT2D;KNSTRN;KPNA2;KPNA6;KPNB1;LGALS3BP;LRP10;LUC7L2;LYN;LYPLA1;MACF1;MAK16;MAP1B;MARS;MAT2A;MAZ;MCM3;MCM3AP;MCM7;MDH2;MDM2;MDN1;MEA1;MEGF9;MESD;MGAT4B;MGMT;MIDN;MINK1;MKKS;MMP1;MORF4L1;MROH1;MROH8;MTA2;MYC;MYLIP;NARS;NCAPD2;NCKAP5L;NDC1;NFS1;NGRN;NKRF;NLE1;NOLC1;NSMF;NSUN5;OAZ2;OGFOD1;OIP5;OLA1;OSBPL10;PACS2;PAFAH1B2;PAIP2;PAN2;PANK3;PARK7;PBX2;PCBP2;PCNT;PDIK1L;PDXDC1;PEBP1;PELI2;PFN1;PGPEP1;PHACTR4;PHAX;PHRF1;PIGQ;PIP5K1A;PIWIL1;PKM;PKP2;PLXNB2;PLXNC1;PNRC2;POLD2;POLE;POM121;POMGNT1;PPM1H;PPP1R14C;PPP2R1A;PPP2R2A;PPP6C;PPRC1;PRDM1;PRDX4;PRICKLE4;PRPS1L1;PRRC2A;PSMC4;PTBP3;PTDSS2;PTEN;QKI;RABL6;RANBP10;

			RAP1GAP2;RBM10;RBM15B;RBMS2;RBSN;RECK;RFC1;RNF10;RNF215;RNF4;RNPS1;RPL12;RPL8;RPS17;RPS2;RUNX2;SAP30L;SAPCD2;SART1;SCARB2;SDHA;SEC24C;SELE;SEPT11;SERPINH1;SERTAD4;SETD1B;SF3B3;SHISA2;SKIV2L;SLC10A7;SLC19A3;SLC25A10;SLC25A36;SLC25A51;SLC35A2;SLC37A4;SLFN11;SMAD5;SMARCA4;SMC2;SMC6;SNX4;SOD2;SON;SORT1;SPTBN2;SRRM2;SRRT;SRSF2;SS18;SSH2;SSSCA1;SSX2IP;STAT5A;STMN1;STN1;STOX2;SUN2;SV2A;TANGO6;TATDN2;TCEAL1;TCERG1;TCOF1;TEX10;TFAP2A;THOP1;TIMM50;TIMP3;TIPARP;TLE3;TLN1;TMCC1;TMED7;TMEM2;TMEM245;TMUB1;TNFSF10;TNRC6B;TOM1;TOP3A;TP53;TP53BP2;TRAT1;TRIM44;TRMT5;TRPS1;TSPAN13;TXN;UBA1;UBE2N;UBIAD1;UBN2;UBR7;UHRF2;UROD;USP15;USP9X;VANGL1;VCL;VCP;VGLL4;WDR6;XPO6;YLPM1;YWHAG;YY1AP1;ZBTB37;ZBTB5;ZBTB7A;ZEB2;ZFAND5;ZFP1;ZFP30;ZFYVE16;ZFYVE9;ZMYND11;ZNF131;ZNF236;ZNF275;ZNF460;ZNF652;ZNF708;ZNF714;ZNF770;ZNF772;ZNF805;ZW10
hsa-miR-490-3p-478131_mir	3.630	0.309	ABCC2;ABCG8;ABL2;ARHGAP29;ASPA;BHLHB9;BMP3;BRIP1;C11ORF98;C14ORF2;C17ORF75;C6ORF132;CAB39;CASTOR2;CBFA2T2;CCDC77;CCND1;CDC14B;CDC42EP4;CDK19;CDKN2AIPNL;CDR1;CERS4;CHST1;CKAP2;CLASP1;CLCC1;CLSPN;CNBP;COL4A3BP;COL9A2;CRAMP1;CREBZF;CXCL16;CYP20A1;DDX19B;DFFA;DISC1;DMKN;DNAJC24;DPPA4;EBNA1BP2;EEF2K;EI24;EIF3L;ENTPD5;ERGIC3;ERN1;ESF1;FADS6;FAHD1;FAM105A;FAM136A;FAM208A;FAM241A;FBXO48;FDXACB1;FOXK1;FOXP4;GCNT3;GEN1;GLP2R;GNB2;GOLGA3;GREB1;HARBI1;HAVCR1;HES2;HINT1;HIST1H2BD;HMGA2;HNRNPA1L2;HOMEZ;IBA57;ICOSLG;INSL6;IRAK4;IRGQ;KBTBD12;KIAA1456;KLF13;KLHL26;KLHL42;KLLN;LILRA2;LMNB2;LONRF2;LPAR2;LRIG2;MAP3K9;MAPK1IP1L;MICB;MOCS3;MORN4;MRNIP;MRPS10;MRPS23;MTMR3;MTRNR2L1;MTRNR2L7;MTURN;MYH9;MYO5A;MYOM2;NCKIPSD;NFASC;NFAT5;NOA1;NOL9;NUDT19;NUFIP2;NUP205;NUS1;NXPE3;PAPLN;PAPPA;PATJ;PCBP1;PCCB;PCF11;PDZD8;PELP1;PGBD4;PLCE1;PLEKHG3;PLXNA3;PPIC;PPM1L;PPP1R15A;PPP1R26;PPP1R3G;PRNP;PRR13;QDPR;RAB33B;RAB36;RAD51;RANBP6;RAP2B;RASA4;RBM28;RBM38;RBM7;RBMS2;RBPJ;RCSD1;RHOA;RILPL1;RPL18A;RSRC1;RSRC2;SCRG1;SDHAF1;SENP5;SIGLEC14;SLC10A6;SLC35E2;SLC35F6;SMARCD1;SMS;SNRPD3;SPAST;SPCS3;SPEM1;SPTY2D1;SRM;SRRD;SUOX;SYT15;TERF1;TGFA;TGFBR1;TGS1;TMEM167A;TMEM59;TMPRSS12;TNKS2;TNNI1;TRAF3IP1;TRIM63;TRIM72;TRMO;TSEN34;TSPAN15;TTC9C;ULK2;VPS53;XKR4;ZBTB3;ZMYM1;ZNF117;ZNF124;ZNF175;ZNF250;ZNF394;ZNF417;ZNF490;ZNF500;ZNF548;ZNF549;ZNF554;ZNF570;ZNF607;ZNF641;ZNF674;ZNF681;ZNF699;ZNF726;ZNF737;ZNF766;ZNF845;ZNF860;ZNF891;ZYG11A
hsa-miR-193a-5p-477954_mir	-2.137	0.316	ARSD;BDP1;C9ORF64;CEBPA;CEBPG;CEP126;CIAO1;CSNK2A1;DAZAP1;DIRC2;ERBB2;FAM20B;FGF19;GDE1;GSKIP;HKR1;IARS;IGF2BP1;ING5;KIAA0391;KLF13;LRRC40;MAVS;METTL8;MLXIP;MRPS23;MTOR;MTRNR2L8;MYO9B;NAA20;NCBP2;NLN;NUP210;NXPE3;OLFML2A;PCSK9;PIK3R3;PKMYT1;PLCXD2;PLXDC2;PPIC;PTPDC1;RBBP6;REPIN1;RPL35A;RPL41;RTL8A;SRR;SURF4;TFAP2A;TFDP2;TMEM245;TOMM70;TP73;TRIM25;WT1;ZC3H7B
hsa-miR-19b-3p-478264_mir	-2.638	0.334	ABCA2;ABHD14B;ABHD17C;ABHD5;ACBD5;ACSL4;ACTB;ACVR1;ADGRL2;ADIPOR2;ADRM1;ADSS;AFF1;AFTPH;AGO1;AGO3;AGPAT5;AHDC1;AKAP2;ALAD;ALG1;ALG2;ALKBH6;AMMECR1L;ANGEL2;ANKIB1;ANKRD10;ANKRD12;ANKRD50;ANKRD52;AP2B1;AP3S2;ARAP2;ARC;ARFGEF1;ARHGAP1;ARHGAP11A;ARHGAP12;ARHGEF26;ARHGEF28;ARID4B;ARL6IP1;ARL8A;ARMC8;ARPIN;ARPP19;ARRDC3;ASNA1;ATF2;ATG14;ATG16L1;ATG2B;ATG5;ATM;ATMIN;ATP6V0E1;ATP6V1B2;ATP6V1C1;ATPAF1;ATXN1;ATXN7;ATXN7L1;AZIN1;B3GALNT2;B4GALT1;BACE1;BAHD1;BAMBI;BCL2L11;BCL3;BCL7A;BCL7B;BEND3;BLCAP;BMP3;BMPR2;BRD9;BRWD1;BRWD3;BTBD10;BTBD3;BTBD7;BTF3L4;BTG1;BTN2A2;C11ORF57;C11ORF96;C12ORF66;C15ORF38-AP3S2;C16ORF70;C2ORF42;C5ORF24;C5ORF30;C5ORF51;C6ORF132;CA13;CAB39;CALM1;CAMSAP1;CAMSAP2;CAMTA1;CAPRIN2;CASQ1;CASZ1;CBX5;CBX7;CBY1;CC2D1A;CCAR2;CCDC137;CCDC80;CCNA2;CCND2;CCNL1;CCNT2;CCSER2;CD164;CD2AP;CD46;CDK19;CENPN;CEP170;CEP350;CEP55;CERCAM;CFL2;CGN;CHD9;CHEK1;CHEK2;CHERP;CIT;CLIC4;CLIP1;CLOCK;CLVS2;CNOT4;CNOT6;CNOT7;COQ10B;COX10;CPD;CPPED1;CREB1;CREB3L2;CREBL2;CREBRF;CSNK1G1;CSNK2A1;CTR9;CUL5;CYP19A1;CYP2U1;DAAM2;DAD1;DBN1;DCAF7;DCAF8;DCBLD2;DCC;DCP2;DCUN1D3;DDX3X;DDX3Y;DDX6;DEF8;DEGS1;DENND6A;DEPDC1;DERL1;DGKH;DHX40;DICER1;DIP2A;DLG5;DMXL2;DNAJA2;DNMT1;DSCR3;DSEL;DSN1;DUT;DYNC1LI2;DYNLL2;E2F8;EFR3A;EGLN3;EHD1;EIF3L;EIF4A2;EIF4E2;ELL2;ELMOD2;ELOVL4;ELOVL5;ENPP4;ENPP5;EOGT;EPC1;EPN2;EPS15;ERCC4;EREG;ESR1;ESRRB;ESYT1;ETV3;EVI5L;EXOC7;FAM102A;FAM218A;FAM46A;FAM46C;FAM83D;FAS;FAT3;FBLIM1;FBXO10;FBXO28;FBXO48;FBXO8;FGFR1OP;FKBP15;FN3KRP;FNDC3A;FOXP1;FOXQ1;FRMD6;FRS2;FXR1;FYCO1;FZD6;G3BP2;GAK;GATAD2B;GCM1;GDNF;GFOD1;GFPT1;GIGYF1;GINS1;GIT2;GMEB2;GMFB;GNPTAB;GPAM;GPATCH8;GPR137B;GRB10;GRK4;GRSF1;GSKIP;HABP4;HADHB;HARS;HBP1;HDAC4;HECW2;HEG1;HHEX;HIC1;HIF1AN;HIPK1;HIPK3;HIST2H4A;HIST2H4B;HMGCS1;HNRNPA1;HNRNPF;HNRNPU;HNRNPUL1;HOMER1;HOXA5;HOXC8;HPRT1;IDNK;IER3IP1;IFITM1;IKZF1;IMPDH1;IMPDH1P11;INO80;ITGA2;ITPR1;JARID2;JAZF1;KAT2A

			;KAT2B;KATNAL1;KCNJ2;KCTD10;KCTD20;KIAA0907;KIAA1468;KIF13A;KIF3A;KITLG;KLF10;KLF13;KLHDC2;KLHL11;KLHL20;KLHL21;KLHL3;KLHL42;KMT5B;KPNA6;LBR;LCLAT1;LDLR;LIN9;LMLN;LONRF1;LPGAT1;LRIG3;LRP8;LTN1;LZIC;MACF1;MALT1;MAP2K3;MAP3K1;MAP3K14;MAP3K9;MAP7;MAPK1;MAPK14;MAPRE3;MAVS;MB21D1;MB21D2;MBD3;MBD4;MBNL1;MBNL2;MBNL3;MBOAT7;MCC;MCM3AP-AS1;MCRIP2;MECP2;MED12L;MED28;MEF2A;MEF2D;MFF;MFSD6;MID1IP1;MIER1;MIGA1;MIGA2;MKL2;MLEC;MLLT10;MMGT1;MOB1B;MOSPD2;MPHOSPH9;MPRIP;MREG;MRPL17;MRPL19;MRPL32;MSMO1;MTHFD1;MTMR12;MTMR6;MTRR;MTUS1;MTX3;MXD1;MYBL2;MYCN;MYLIP;NACC1;NACC2;NAPB;NCBP2;NCKAP5;NCOA3;NDEL1;NDFIP1;NDRG1;NDUFB2;NF1;NFATC2IP;NFIA;NFIB;NICN1;NIPA1;NPTN;NR3C1;NR3C2;NRBF2;NRBP1;NUFIP2;NUP54;NUS1;NUTF2;OCRL;ODF4;OTUD1;OTUD4;OTUD7B;PABPC4L;PAFAH1B2;PAICS;PALLD;PALM2-AKAP2;PAPD4;PATL1;PCDH10;PDE4A;PDE4D;PDRG1;PDZD11;PFN1;PFN2;PGK1;PGM2L1;PHF13;PHLDA1;PHLDA3;PIGS;PIK3R3;PITX1;PIWIL4;PKM;PKNOX1;PLAU;PLEKHF2;PLXNC1;PNRC1;POGZ;POLI;PPARA;PPP1R15B;PPP2R5E;PPP6R1;PPP6R2;PPTC7;PRICKLE2;PRKAA1;PRKACB;PRKN;PRR14L;PRRG4;PRUNE2;PSAP;PSG4;PSMD9;PTBP2;PTCD2;PTEN;PTENP1;PTP4A1;PTPN4;PTPRB;PTPRG;PURG;PXYLP1;QKI;RAB14;RAB18;RAB1A;RAB21;RAB2B;RAB34;RAB5B;RAB8B;RACGAP1;RAF1;RAN;RAP1A;RAP1B;RAP2C;RAPGEF2;RAPGEF4;RAPGEF6;RASA1;RASSF1;RASSF2;RASSF5;RBBP8;RBM20;RBM25;RBM38;RCOR1;RDH11;REEP3;REM1;RGL1;RHEBL1;RHOB;RLIM;RNASE1;RNF11;RNF111;RNF145;RNF167;RNF216;RNF41;RNF44;ROR1;RORA;RPA2;RPAP2;RPF2;RPS4Y1;RRAGD;RRAS2;S1PR2;SAMD1;SAMD8;SATB1;SBF2;SCD;SDE2;SEC23B;SEC61A1;SEC63;SECISBP2L;SEL1L;SEL1L3;SEMA4C;SEMA6B;SEPHS2;SERBP1;SERINC3;SESN3;SESTD1;SF3B3;SFTPA1;SGK1;SGSM3;SGTB;SH3KBP1;SHCBP1;SIRPB2;SIVA1;SIX4;SKIL;SLAIN1;SLC12A7;SLC25A12;SLC27A1;SLC30A7;SLC35D1;SLC37A1;SLC38A2;SLC44A1;SLC46A1;SLC48A1;SLC4A7;SLC6A8;SLC7A11;SLC9A1;SLC9A6;SLMAP;SMAD4;SMAD5;SMARCA2;SMCR8;SMG1;SMOC1;SMYD2;SNAPIN;SNX17;SNX5;SOCS1;SOCS3;SOCS4;SOGA1;SON;SOX4;SOX6;SPART;SPATA2;SPTSSA;SPTY2D1;SRSF7;ST13;STAT5B;STEAP2;STK38;STK4;STOX2;STX12;STX16;STX6;SUZ12;SWT1;SYBU;TAF4;TBC1D13;TBC1D25;TBC1D4;TBRG1;TCF4;TES;TFB1M;TGFB1;TGFBR2;TGIF1;TGOLN2;THBS1;THOP1;TLR2;TMBIM6;TMEM106B;TMEM107;TMEM117;TMEM138;TMEM2;TMEM45A;TMEM64;TMEM9B;TMOD3;TMTC1;TNFAIP3;TNFRSF10B;TNFRSF11A;TNFRSF12A;TNFRSF1B;TNIP1;TNKS;TNPO2;TNRC6A;TNRC6B;TOM1L2;TP53;TP53INP1;TPGS2;TPRG1L;TRAK2;TRIM2;TRIM33;TRIM37;TRIM59;TRPC3;TSC22D3;TTC9C;TXLNG;UBE2A;UBE2D3;UBL3;UBN2;USP13;USP34;USP37;USP8;VAMP1;VAMP3;VPS37A;VPS37B;VPS4B;WAC;WASL;WBP1L;WBP2;WBP4;WDFY2;WDR1;WDR20;WDR26;WDR33;WDR45B;WEE1;WNK1;WNK3;WNT10A;WNT7B;XIAP;XYLT2;YTHDC1;YTHDF1;YY1;ZBTB18;ZBTB4;ZBTB47;ZBTB7B;ZCCHC14;ZDHHC18;ZDHHC7;ZER1;ZFAND5;ZFHX4;ZFYVE26;ZFYVE9;ZIC5;ZMAT3;ZMYM2;ZMYND11;ZNF107;ZNF134;ZNF138;ZNF154;ZNF217;ZNF367;ZNF417;ZNF423;ZNF507;ZNF521;ZNF526;ZNF544;ZNF567;ZNF618;ZNF644;ZNF680;ZNF711;ZNF721;ZNF772;ZNF800
hsa-miR-28-5p-478000_mir	-0.303	0.335	AAAS;AHDC1;ARHGAP42;ATG9A;BAG1;CASTOR2;CCDC6;CCND3;CD276;CDKN1A;CENPV;CHERP;CMC4;CNN3;CRISPLD2;CRTC3;CS;CTIF;CXCL5;DDTL;DENND4B;DRAXIN;E2F6;EDA2R;FAM168A;FKBP5;FOXJ3;G3BP1;GEMIN4;GTF3C4;HMX3;HNRNPC;IER3;IGF1;IL34;IMPDH1;IQSEC2;KCNH2;KLF7;KLHL11;KLHL12;KPNA4;LINC00346;MAD2L1;MAP2K3;MAPK1;MORF4L1;MPL;MSI2;MYH15;N4BP1;NFE2L1;NFIC;NKIRAS2;NOP53;NOS1AP;NRN1;NUDCD3;OTUB1;PAQR5;PCYOX1;PIANP;PLEKHG5;PRDM2;PRRC2B;PRSS16;PTPRJ;R3HDM4;RAB36;RAP1B;RETREG2;RNF165;SEMA4C;SETD7;SIK1;SLC7A5;SMYD1;SNX1;SRD5A1;SREBF2;SRF;STAT5B;TEAD3;TEX261;THRA;TLK1;TMED4;TMEM167A;TP53;TPM4;TSC22D1;TUBB2A;TUBB4A;TUFM;TULP4;UMPS;UNG;USP9X;VPS37B;YIPF6;ZBTB47;ZCCHC3;ZFP91;ZNF106;ZNF14
hsa-miR-10b-5p-478494_mir	-1.698	0.340	AASDH;ACAD8;ACLY;ACOT13;ACTG1;ACVR2A;ADRA1B;AHCY;AHCYL2;AHSA2;AKT1;ALKBH4;ANKRD33B;ANP32E;ANXA7;AP3M1;API5;APLNR;APOC3;ARFGEF1;ARHGAP18;ARSK;ASCL2;ATG13;ATIC;ATP1B4;AXL;BCL2L11;BORCS5;BRCA1;C17ORF105;C1GALT1;C3ORF36;C6ORF106;C6ORF89;CADM1;CAMSAP3;CARD8;CCDC8;CCNG1;CCS;CD3D;CDC6;CDK12;CDK2;CDKN1A;CDKN2A;CENPL;CHAF1B;CHDH;CHMP1B;CHPF2;CLN8;CLPTM1;CMPK1;CNKSR3;CNOT6;COL6A2;COPS5;CREB1;CRIPT;CRK;CRLF3;CRY2;CSGALNACT1;CSMD1;CSNK2A1;CSRNP3;CTBP1;DDHD2;DDX58;DGAT1;DIAPH2;DLAT;DLX1;DMWD;DPF2;DVL3;EBNA1BP2;EIF1;ELOVL7;EPHA4;ERLIN1;ESYT1;EXOSC2;EXTL3;FAHD1;FAM118A;FAM13A;FAM208A;FASN;FBXO31;FEM1A;FEM1B;FHL2;FOXRED2;FRRS1;FUT1;FUT6;FXR2;FZD2;GATA3;GK;GK5;GLB1L3;GLOD4;GP1BA;GPCPD1;GRINA;GRK6;GSR;GSS;H3F3B;H3F3C;H6PD;HHAT;HHIP;HIST1H2AL;HIVEP2;HK1;HLA-E;HMGN2;HNRNPF;HOXB3;HOXD1;HOXD10;HOXD11;HSPA1B;HTATIP2;ID4;IGSF1;INHBA;IPCEF1;IRGQ;KBTBD6;KCTD11;KIAA0100;KIAA1143;KIAA1551;KIF21A;KL;KLF4;KLHDC8A;LAMC1;LEMD3;LILRA2;LIX1L;LMF2;LPIN3;LRP3;LRRC59;LSS;LYL1;MAPKAPK5;MAPRE1;MAVS;MBNL1;MBNL2;MBNL3;MCTS1;METTL7A;MKN

			K2;MRPS14;MSL3;MSTO1;MTRNR2L10;MTRNR2L11;MTRNR2L3;MTRNR2L7;MTX3;MYF5;NAP1L1;NCBP3;NCOA6;NCOR2;NDUFB5;NEK7;NELL2;NF1;NIPSNAP2;NOTCH1;NPTX1;NR2C2;NR4A3;NSD3;NUAK2;NUDT4;NUFIP2;OLFM3;ONECUT3;OPA3;ORAI2;ORC1;PABPC1;PAFAH1B1;PAICS;PARL;PAX6;PDCD4;PDK3;PFAS;PHAX;PHF20;PIEZO1;PIK3CA;PIK3CD;PLA2G2C;PLA2G4F;POC1A;POLR2A;POU2F2;PPARA;PPFIBP1;PPP1R13B;PPP1R15B;PPP3CB;PRMT7;PRPF8;PRRC2C;PSD4;PSMD11;PSMD13;PTEN;PTPRT;QPCT;RAB15;RALBP1;RAPGEF2;RARG;RBM12B;RBM17;RGP1;RNF2;RORA;RPS15A;RPS8;RPS9;RSRC1;RTKN2;RUNDC3B;S1PR2;SAPCD2;SART3;SCMH1;SDC1;SETD7;SFRP1;SFT2D2;SLC11A2;SLC24A4;SLC25A30;SLC25A43;SLC25A5;SLC2A3;SLC48A1;SLC5A5;SMCHD1;SNX4;SON;SPOUT1;SREBF1;SRP19;SRSF1;STAT6;STK33;STT3A;SURF6;SYNPO;SYT11;TCF15;TCF19;TENM3;TFAP2C;TFPI;THUMPD1;TIAM1;TIMM50;TMED4;TMED5;TMEM101;TMEM109;TMEM127;TMEM170A;TP53;TPM1;TPM4;TRA2B;TRIM2;TSPAN33;TTYH3;TUBA1B;TXNDC16;UBE2Z;UBXN7;UGDH;UHRF1;URGCP;UROD;USP3;USP6;USP6NL;USP9X;WDR13;WDR77;XIAP;XPNPEP3;XPO7;ZBTB8A;ZC3H18;ZEB1;ZMYND11;ZNF223;ZNF280B;ZNF318;ZNF329;ZNF394;ZNF445;ZNF460;ZNF502;ZNF574;ZNF708;ZNF878
hsa-miR-500a-5p-478309_mir	-1.660	0.345	AAK1;AMOT;ANKRD40;APP;BAMBI;BLOC1S3;C16ORF58;C16ORF72;C8ORF82;CCNT1;CCP110;CDC5L;CDKAL1;CKAP2L;COMMD2;CPNE1;CRX;CT62;CYLD;CYP2U1;CYSLTR2;DCAF4L1;DENND6B;DNAH17;DNAJB14;DNAJC21;ERGIC2;EXTL3;FAM104A;FAM229B;FAM71F2;FAM9C;FFAR4;FOXC1;FRRS1L;FSD1L;FSHB;FUT2;GABRG2;GALK2;GAS1;GCM2;GGT6;GNAQ;GPM6B;GRIN2B;GSG1;GSTCD;GTF2F2;H2AFX;HAS2;HSD3B1;INIP;ITPRIPL1;KCNT2;KDM1A;KIF1C;KLRD1;LYPD6;MAOA;MAP3K9;MBD4;MBNL1;MCM2;MED17;MOV10L1;MPIG6B;MTHFD2;MYO5A;NFXL1;NNT;NOX5;NPEPPS;NR2C2;NUP50;NYX;OR9Q1;OTOG;OTUD7B;PARD6B;PGBD4;PIP4K2A;PKNOX1;PLEKHB2;PLEKHG3;POLR1A;POLR1B;POLR3G;PPIL2;PRKAA2;PRRC2A;PRRC2B;PRRG4;PTPN14;PXK;RAB17;RBBP4;REL;RGS6;RMDN1;RNF165;RPS4X;RPS6KB1;SCYL3;SFT2D2;SIK3;SLC1A1;SLC1A2;SLC25A20;SLC2A3;SLC35B3;SLC35G1;SLC36A1;SLC38A9;SLC7A6OS;SLC8A1;SNRPD3;SREK1IP1;SRRD;SRSF1;SRSF10;SRSF2;ST6GALNAC3;STAT1;STK4;SYNJ2BP;TACC1;TAX1BP1;TMEM242;TMX4;TTC33;TTLL12;TUBGCP5;UBE4B;UNC5C;USF3;VANGL2;VPS18;YME1L1;ZBTB34;ZBTB8B;ZDHHC20;ZIC5;ZNF227;ZNF460
hsa-miR-150-5p-477918_mir	-2.744	0.349	A1CF;ABCB7;ABCG8;ABHD15;ABHD18;ABHD2;ACOT9;ACOX1;ACSL6;ADIPOR2;AGA;AGMAT;AGO3;AGTPBP1;AHI1;AIFM2;AKIRIN1;ALDOA;AMD1;AMOTL2;ANKFY1;ANKRD65;ANKS4B;ANO7;APEX2;APOH;APOPT1;ARHGAP29;ARMT1;ARRB2;ARSE;AS3MT;ASB16;ASB8;ATAD2B;ATCAY;ATP13A3;ATP1B3;ATP2B1;ATP9A;BBS5;BCAS4;BCL11B;BDP1;BHMT2;BIRC5;BMP8B;BTN3A2;C11ORF1;C12ORF49;C12ORF65;C14ORF119;C15ORF40;C16ORF58;C18ORF32;C21ORF33;C2CD4B;C3ORF36;C8ORF37;C8ORF46;CACYBP;CALCOCO2;CAMK1D;CAMK4;CAPZA2;CARHSP1;CAST;CAVIN1;CBL;CBX5;CCDC198;CCDC30;CCR6;CCS;CD96;CDC14B;CDIPT;CDK2;CDKN2AIPNL;CDS2;CENPM;CEP104;CEP135;CEP72;CISH;CNKSR3;CNNM2;CNPPD1;CNST;COL4A4;COL9A2;CORO2A;COX19;CPT1A;CRCP;CREB1;CRISPLD2;CSNK1E;CTNS;CWC25;CXCR4;CXORF21;CXORF38;CYB5A;CYCS;CYTIP;DCTN5;DDI2;DDOST;DEGS1;DGCR6L;DHTKD1;DIS3;DIS3L;DLEU1;DNAAF3;DNAJB13;DNAJB4;DNAL1;DNASE2;DPP9;DPYSL5;DRAXIN;DSN1;DSTYK;ECHDC3;EFCAB11;EGR2;EIF2B2;ELK1;ELOB;EMC3;EMC7;EMP2;ENTPD4;EP300;EPHB2;EREG;ESR2;ETV3;EXO5;EZH2;F2R;F2RL1;FAAP24;FAHD1;FAM13B;FAM153B;FAM185A;FAM213A;FAM241A;FAM89A;FAXC;FBXO47;FFAR4;FGD6;FHDC1;FKBP9;FLT3;FOPNL;FOXK1;FOXRED2;FRAT2;FXYD5;GAN;GATAD2B;GDF5OS;GGA2;GJC1;GJD3;GK5;GMEB1;GNB5;GNE;GOLGA2;GOSR1;GPBP1;GPN2;GPR137B;GPR182;GPRIN3;GRM6;GTF2H2;GTF2H2C;GTF2H3;HAS2;HAUS2;HIF1AN;HILPDA;HIP1;HIST1H2BG;HIST1H2BJ;HMGB1;HOOK3;HS3ST1;HSPA4L;HYPK;ICA1L;IGF2;INTS7;INTU;IP6K2;IPP;IRAK4;ISCA2;ISG20L2;ISY1;JDP2;KANSL3;KCNK3;KCNK5;KCTD20;KIAA0930;KIAA1549;KIAA1551;KIAA1841;KIF3A;KLHL21;KLHL7;KPNA6;KRBOX4;LAIR1;LAT2;LCTL;LDHD;LEAP2;LGSN;LINC00598;LIPG;LMTK2;LNPK;LRIF1;LRRC15;LRRC27;LRRC58;LRRD1;LTBP2;LY6G5B;LYRM7;MAN2B2;MANEAL;MAPK13;MASTL;MBD1;MBD6;MCTS1;MED16;METTL21A;METTL8;MFSD11;MFSD2A;MICA;MIPOL1;MIS18A;MIXL1;MLN;MMAB;MMP14;MON1B;MPPE1;MRPL37;MRPS10;MRPS27;MS4A3;MSANTD3;MSH3;MSRB2;MTMR9;MTRNR2L5;MTSS1L;MUC4;MUT;MYB;MYH9;MYLK3;MYO1F;N4BP2L2;NANOG;NDUFV3;NEK8;NFYA;NIPAL1;NKD1;NKX2-1;NME6;NMNAT1;NOL9;NOTCH3;NPHP1;NPHS1;NR2F2;NSUN4;NUDT3;NUGGC;OCIAD1;OLAH;ORAI2;P2RX7;PAIP2B;PAK3;PARD3;PARD6G;PCP4L1;PCYT1A;PDCD4;PDE6A;PDHB;PDIA6;PDZD8;PGBD4;PGM2L1;PHF12;PHLDA1;PIAS2;PIGM;PIGR;PIK3C2A;PLA2G16;PLAA;PLEKHA2;PLEKHM3;PLPBP;PLPP3;PLXDC1;PNPLA3;PNRC1;POFUT1;POLD3;POLK;POLR3A;PPIE;PPM1A;PPP2CB;PPP2R3A;PRIM1;PRKAB1;PRKCA;PROSER2;PRPF38A;PSMC1;PTCHD1;PTCHD3;PTGIS;PTPN4;PTPRR;PURB;PWWP2A;PXMP4;QRFPR;QSOX1;RAB13;RAB21;RAB3IP;RABAC1;RABGAP1L;RABIF;RABL3;RAI1;RANGAP1;RAPGEF6;RBL1;RBM3;RBM41;REL;RFK;RHOH;RNF115;RNF157;RNF165;RNF19B;RNF34;RPL14;RPL24;RRP1B;RRP36;RTN2;RUNDC1;RUNDC3B;S1PR1;S1PR3;SAR1A;SCN2B;SCO1;SEC14L4;SENP8;SEPT14;SERF2;SERINC1;SERINC3;SGO1;SGTB;SH3BP5;SHE;SIGLEC11;SIGLEC9;SKIDA1;SLC1A5;SLC25A37;S

			LC25A44;SLC2A1;SLC33A1;SLC35F5;SLC35F6;SLC35G1;SLC43A2;SLC6A4;SLC7A11;SLFN13;SMIM7;SMUG1;SNX2;SOCS5;SP1;SP2;SPAG16;SPEM1;SPIB;SPIC;SPPL3;SRCAP;SRCIN1;SSSCA1;STAC2;STAT1;STAT5B;STK11;STX4;SUGT1;SYAP1;SYNJ2BP;SYNPO2;SYNPO2L;TBC1D16;TBL3;TCN2;TEAD1;TEP1;THAP1;THAP6;TIAL1;TIMM10;TIMM50;TLDC1;TLR10;TLR7;TM4SF5;TMEM127;TMEM134;TMEM174;TMEM33;TMEM50A;TMEM92;TMOD2;TMOD3;TNFAIP8L1;TNFSF15;TNIP3;TNS4;TOM1;TOR1AIP1;TP53;TPMT;TRAF3IP2;TRAPPC10;TRIM35;TRIM65;TRIM72;TRIOBP;TRIP11;TRPS1;TRPV2;TRUB2;TSPAN11;TTC31;TTC4;TTLL12;TTPAL;TTYH3;TUBB4A;TXK;UBE2V2;UBOX5;ULK2;USP15;VEGFA;VPS53;WDFY2;WDR12;WDR53;WDR77;WDR92;WDR97;WFDC6;WNT7B;WWC1;WWC2;XIAP;XKR4;XPNPEP3;XPOT;XPR1;YPEL1;ZBED1;ZBTB25;ZBTB7A;ZCCHC24;ZEB1;ZFP14;ZHX3;ZMAT3;ZNF207;ZNF257;ZNF347;ZNF350;ZNF426;ZNF454;ZNF460;ZNF514;ZNF551;ZNF573;ZNF578;ZNF582;ZNF626;ZNF665;ZNF682;ZNF699;ZNF7;ZNF70;ZNF708;ZNF786;ZNF844;ZNF878;ZSCAN2;ZYG11A
hsa-miR-380-3p-477854_mir	-2.955	0.350	ADM;ALG9;ANKRD42;ANKRD44;AZIN1;B2M;BMI1;BRD4;C5ORF24;C8ORF4;CAPZA2;CCDC74A;CCDC74B;CD226;COMMD3-BMI1;DSTN;EIF5;EPB42;ERRFI1;FAM182B;FBXW7;FEM1A;FOXN2;FSD1L;GIN1;GLO1;GNA13;HIC2;HIST1H3H;HNRNPA3;IFIT1;IGFBP5;ING1;KLHL42;LINS1;LYSMD3;MAP1B;MIPOL1;MMADHC;MRPS10;NETO2;NFE2L1;NOTCH2NL;PAPD5;PGBD4;PM20D2;PMAIP1;PPP2R1B;PTAR1;RAB11FIP1;RAB9B;RAP1B;REL;ROCK2;RSL24D1;SCML2;SGMS1;SH3TC2;SHOC2;SIKE1;SLC23A3;SNX2;SOCS1;SPRED1;SUMO2;SYPL1;TFDP1;THBS1;TNRC6C;TRPS1;TUBD1;UBE2K;WDYHV1;YIPF6;ZFP37;ZNF449;ZNF460
hsa-miR-885-5p-478207_mir	1.841	0.368	ANKRD17;APOB;ARHGAP42;ATG12;BNIP2;BUB1;BUB3;C2orf91;C8ORF37;CASP16P;CASP3;CDK2;CEP170;CTNNB1;EIF4G2;FBXO28;FEN1;GAL;GLUD1;GLUD2;GREB1;GXYLT1;HHIPL1;HNRNPL;HOXB3;IGF1R;JMY;KNTC1;MACC1;MCM5;METTL1;MSL2;NPVF;NWD1;PAFAH1B1;PAPD4;PELI3;POF1B;PPIP5K2;RAC1;RUNX1T1;SDCBP;SERBP1;SHC3;SIX4;SMIM21;SOD2;SRSF2;STAMBPL1;SULT1B1;SUV39H2;SYNM;TAF1D;TBCEL;TFAP2B;TMC7;TMEM185B;TNIP1;TRIP13;USP22;ZNF148;ZNF350;ZNF460;ZXDB
hsa-miR-103a-3p-478253_mir	-1.657	0.383	AADAT;ABCF2;ABL2;ACTR2;ACVR2B;ADAM10;ADGRL1;ADORA3;AEBP2;AGFG1;AGO1;AGO2;AGO3;ALDH3B1;ALG2;AMOT;ANKFY1;AP2A1;ARF6;ARGLU1;ARIH1;ARL3;ARL4C;ARL8A;ASH1L;ATG12;ATG14;ATG9A;ATP13A3;ATP5G3;AVL9;AXIN2;B3GNT2;BAZ2A;BCKDK;BCL2;BFAR;BNIP3;BTLA;C11ORF58;C16ORF58;C16ORF72;C1ORF21;C20ORF27;C2ORF42;C9ORF62;CA12;CAAP1;CAB39;CACNA1C;CALU;CAMKK2;CAMSAP1;CAPZA2;CARD10;CAV1;CCDC83;CCNE1;CCNT1;CCT3;CD180;CD274;CDADC1;CDC25A;CDC37L1;CDC42SE2;CDCA4;CDK1;CDK17;CDK2;CDK6;CDV3;CEP55;CFAP45;CITED2;CKAP5;CKMT1A;CLIP1;CNNM2;CPEB2;CPEB3;CPSF7;CREB1;CREBRF;CRKL;CSNK1G3;CSNK2A1;CUL4A;CYB561A3;CYP26B1;CYP2C8;CYSLTR2;DAPK1;DECR1;DEPDC1B;DESI1;DHX33;DICER1;DIEXF;DMPK;DMTF1;DNAJA1;DNAJB4;DNAJC10;DNAJC9;DOCK11;DST;DUSP14;DVL1;DYRK2;E2F7;EDC3;EFNB2;EFTUD2;EI24;EIF1AX;ELK4;EML4;EN2;ENPP2;ENPP4;ENTPD1;EPC1;ERN1;EXD2;EXOC5;FAM103A1;FAM122A;FAM122B;FAM171A1;FAM229B;FAM49A;FAM89A;FAM98A;FAM9C;FBXW7;FCF1;FEM1C;FGF2;FGFRL1;FKBP1A;FLCN;FMC1;FOXC1;FURIN;FZD6;G3BP2;GABARAPL1;GABRB1;GATAD2A;GCC1;GGA3;GLP2R;GLRX;GNAI3;GNAT1;GNB1;GNG12;GNS;GOLGA8B;GOLT1B;GOPC;GPCPD1;GPD1;GPR180;GPRC5A;GSG1;GTPBP1;HAUS3;HCFC2;HDDC2;HELZ;HIC2;HIST2H2BE;HNRNPA2B1;HOXA3;HOXC10;HOXD13;HPRT1;ID2;IDH3A;IFNGR2;IFT74;IGSF3;ING1;INSIG1;INSL6;IPPK;IRF2BP2;ITGA2;ITM2C;IVNS1ABP;JAKMIP2;JOSD1;KATNAL1;KCNC4;KCNG3;KDELR1;KIF23;KIF5A;KIF5B;KLF4;KLHL18;KPNA1;L2HGDH;LAMP2;LARP4B;LASP1;LATS1;LBR;LCOR;LIN7C;LRIF1;LUC7L;LUZP1;MAP3K7;MAP4;MCM7;MDM4;MED13;MEF2D;MEX3C;MIB1;MIS18BP1;MLLT6;MPDU1;MPLKIP;MRPL51;MT1E;MTF1;MTFR1L;MTHFR;MTMR3;MTMR4;MYB;MYBPC1;MYCN;MYO5A;MYO5B;N4BP1;NAA15;NACC1;NACC2;NDEL1;NFIA;NIN;NNT;NPY4R;NRBP1;NSF;NUCKS1;NUFIP2;NUMB;NUP50;NUS1;ODF2L;OGT;OIP5;OLFM4;OPRM1;ORC4;OSBPL3;OTUD7B;PAFAH1B2;PAG1;PAGR1;PAQR3;PAWR;PDE4D;PDK4;PDZD8;PEA15;PER1;PHC3;PHF19;PHKA1;PIEZO1;PIK3R1;PLAG1;PLEKHA1;PLEKHF2;PLPBP;PLSCR4;PNISR;PNPLA6;POLD3;POLDIP2;POMGNT1;PPIG;PPIL1;PPP1R11;PPP1R12C;PPP1R16B;PPP2CA;PPP2R5C;PPP6C;PPP6R3;PRDM4;PRKAB2;PRKAR2A;PRR14L;PTEN;PTPN3;PURA;PURB;RAB10;RAB1B;RAB23;RACGAP1;RAD21;RAD51;RBBP6;RCC1;RECK;REL;RFK;RIC1;RIMS3;RNF144B;RNF168;RNF217;RNMT;RPN1;RPRD1B;RPS24;RPS6KB1;RPSAP58;RRAGC;RS1;RSL1D1;RUNX1T1;RUNX2;SALL1;SAV1;SCAF1;SDCBP;SEMA6A;SERPINB5;SETD1B;SF3A1;SF3A3;SFRP4;SH3BP5;SHOC2;SIPA1L2;SIRT4;SLAIN2;SLC25A39;SLC28A1;SLC2A3;SLC30A7;SLC35A4;SLC35B2;SLC39A10;SLC52A2;SLC6A9;SLC9A6;SLCO3A1;SMAD7;SMARCA5;SMARCE1;SNCG;SNTB2;SNX16;SON;SOWAHC;SPATS2;SPATS2L;SPRED1;SREK1;SRSF1;SSU72;STAMBPL1;STK33;STK38;STK40;STX6;SUN2;SVIP;SYNJ1;SYNRG;SYS1;TAF13;TARBP2;TBC1D12;TBPL1;TBRG1;TFAP2A;TGFBR3;TIMP3;TJP1;TK1;TLE4;TLK1;TM4SF1;TM7SF3;TMCC1;TMEM100;TMEM170A;TMEM255A;TMEM43;TNRC6B;TPD52;TRIM35;TRIQK;TSC22D2;TTLL5;TULP4;TWF1;TWNK;UBE2B;UBE2Q1;UBE2R2;UBFD1;UBN2;UBR3;USP

			15;USP42;VCAN;VEZT;VPS4A;WEE1;WNT3A;XPC;XPO7;YAF2;YIF1B;YIPF6;YRDC;YTHDC1;YWHAH;ZBTB10;ZBTB34;ZBTB38;ZBTB8A;ZCCHC14;ZDHHC16;ZFHX3;ZHX1;ZNF100;ZNF273;ZNF284;ZNF449;ZNF585B;ZNF606;ZNF622;ZNF623;ZNF680;ZNRF2;ZNRF3;ZRANB1;ZYX
hsa-miR-133a-3p-478511_mir	-4.262	0.388	ABHD18;AFTPH;ALDOC;ANGEL2;ANGPT4;ANXA2;APOL6;ARL6IP1;ARPC5;ATP13A3;BCAN;BCL2L1;BCL3;C11ORF24;C17ORF64;CACNA1C;CASP9;CCDC39;CCNI;CDC42;CDK5R1;CDKN1A;CERS2;CHMP3;CMTM4;CNN2;COL1A1;CTGF;DCAKD;EFHD2;EGFL7;EGFR;EMID1;ERBB2;FAM120C;FAM160B1;FAM213B;FBN1;FOSL2;FOXL2;FSCN1;FTL;GSTP1;HAPLN1;HCN2;HCN4;HIST2H2AC;IBA57;IGF1;IGF1R;IP6K1;ITPKB;KCNH2;KCNQ1;KPNA6;KRT7;LASP1;LDLRAP1;MC2R;MCL1;MEG3;MLEC;MMP14;MSN;MYPN;NCDN;NFAM1;NGFR;NR2C2;NR4A2;PACS2;PDE1A;PDLIM5;PER2;PIAS2;PIGR;PIK3R2;PKM;PLEKHA3;PLEKHG2;PNP;POU2F2;PRDM16;PRELID1;PRRT2;PSMC4;PSMG1;PTMA;RAB5C;RBMXL1;RBPJ;RFFL;RFT1;RGS3;RHOQ;RNF103-CHMP3;RNF168;SEC61B;SENP1;SEPT6;SERPINH1;SESN2;SESN3;SFTPB;SGMS2;SMIM14;SNX30;SNX33;SOX4;SP1;SUPT16H;SYAP1;TAGLN2;TCTEX1D2;THBS2;TMEM59;TPM1;TPM3;TRIM71;TXNRD3NB;UBA2;UCP2;UGT2B10;VEGFA;VKORC1;ZBTB37;ZEB1;ZFP28;ZMAT4;ZNF394;ZNF704
hsa-miR-1-3p-477820_mir	-2.915	0.394	ABCB1;ABCB5;ABCB6;ABCB7;ABCC1;ABCC4;ABHD11;ABHD12;ABI2;ACADVL;ACOD1;ACP2;ACTA1;ACTB;ACTC1;ACTN1;ACTN4;ADAM12;ADAMTSL4;ADAR;ADPGK;AFAP1;AGMAT;AGO1;AGRN;AGTRAP;AHNAK2;AIFM2;AIM2;AKAP12;AKAP4;AKR1D1;ALDH2;ALG2;ALG3;ALPI;AMDHD1;AMZ1;ANGPTL4;ANK1;ANKFY1;ANKIB1;ANKRD17;ANKRD29;ANP32A;ANP32B;ANP32E;ANPEP;ANXA2;AP1B1;AP1M1;AP1S1;AP1S3;AP2S1;AP3B1;AP3D1;APEH;API5;ARCN1;ARF3;ARF4;ARG1;ARGLU1;ARHGAP29;ARHGEF18;ARID1A;ARID2;ARMC10;ARPC5;ARPP19;ASH2L;ASPH;ASXL2;ATL3;ATP13A1;ATP2B4;ATP5E;ATP6V0A1;ATP6V1A;ATP6V1B2;ATP6V1C1;ATP6V1E1;AXL;B3GNT2;B4GALT1;BAG4;BAG5;BAX;BCAP29;BCAS4;BCKDHB;BCL6B;BCL7B;BDNF;BLCAP;BMP7;BRDT;BRI3BP;BRPF3;BSCL2;BTC;C12ORF10;C12ORF40;C12ORF49;C12ORF57;C17ORF102;C1ORF27;C1ORF56;C2ORF48;C5ORF51;C6ORF118;C8A;CA3;CACNA2D1;CALM1;CALM2;CALM3;CALR;CAMK2G;CAND1;CAP1;CAPG;CAPN1;CAPRIN1;CAPZA1;CAST;CAVIN2;CBR4;CBX2;CBX5;CCDC102A;CCDC124;CCDC134;CCDC170;CCDC180;CCDC22;CCDC88C;CCL14;CCL2;CCND1;CCSAP;CD109;CD2AP;CD44;CD63;CDC42;CDC42BPB;CDC42SE1;CDCP1;CDH13;CDH2;CDH4;CDK14;CDK4;CDK9;CEBPA;CEBPZ;CENPF;CEP295;CERS2;CETN3;CHAF1B;CHAMP1;CHML;CHMP2A;CHRAC1;CHST11;CHSY1;CLCN3;CLDN12;CLEC1A;CLTC;CNIH4;CNN3;CNOT6;COIL;COL12A1;COMMD2;COPB1;COPG1;COPZ1;COQ6;COQ7;CORO1C;COTL1;CPOX;CPSF1;CPSF3;CREB3L2;CRELD2;CRIP2;CRK;CRYGS;CSNK2A2;CSRP1;CSTF3;CTBP1;CTBP2;CTDNEP1;CTSC;CTTN;CUL4B;CXCL1;CXCL12;CXCL2;CXCL3;CXCL8;CYP20A1;DBN1;DCAKD;DCTPP1;DCUN1D1;DDR2;DDT;DDTL;DDX19B;DDX42;DDX5;DDX50;DDX6;DDX60;DEFB125;DFNA5;DGKH;DHRS1;DHX15;DKK1;DLK1;DMTN;DNAAF5;DNAJB1;DNAJC10;DOK6;DOLPP1;DPP7;DPY19L1;DPY30;DRAP1;DROSHA;DSG2;DTX1;DYNC1LI1;E2F5;ECHS1;EDF1;EDN1;EDRF1;EFCAB1;EFR3A;EFTUD2;EGFR;EHMT1;EHMT2;EIF1AX;EIF4E;EIF4G1;ELMOD2;ELP1;EMD;EML3;EML4;EMP3;EPB41L2;EPB41L4B;ERICH3;ERMN;ESYT1;ETNK1;ETS1;ETV7;EXOC2;EXOC3;EXOSC2;F11R;F2;F2RL1;FABP3;FADD;FADS1;FAM102A;FAM167A;FAM208A;FAM216B;FAM57A;FAM81A;FANCI;FASN;FBLN2;FBN1;FBXO22;FBXO33;FBXO45;FERMT2;FGD4;FGFR2;FHDC1;FIG4;FILIP1;FLNA;FLNB;FLNC;FLOT2;FMNL2;FMNL3;FN1;FNDC3A;FNDC3B;FOLR1;FOXN4;FOXP1;FRG1;FRMD7;FRS2;FTSJ1;FUBP1;FUBP3;FYB1;FZD7;G3BP1;G3BP2;G6PD;GAK;GATA3;GATA4;GATA6;GCFC2;GCH1;GIMAP4;GJA1;GJB3;GLI2;GNA13;GNAI1;GNAI2;GNAI3;GNAT2;GNB1;GNB2;GNG5;GNPDA2;GNPNAT1;GNRHR;GOLGA7;GOLPH3;GPAA1;GPAT4;GPC1;GPD2;GPR137C;GPR83;GPX1P1;GSTO1;GTF2H1;GTF3C6;H1FX;H3F3B;H3F3C;HACD2;HACD3;HADH;HAND2;HCN2;HCN4;HDAC2;HDAC4;HERC2;HERC5;HIF3A;HIGD1A;HINT2;HIPK3;HIST1H1B;HIST1H3B;HIST2H2AC;HIST2H3A;HIST2H3C;HIST2H3D;HIST3H3;HMOX1;HNRNPA1;HNRNPA1L2;HNRNPA3;HNRNPD;HNRNPH1;HNRNPU;HOOK1;HP1BP3;HPS4;HS2ST1;HSD17B11;HSD17B8;HSD3B7;HSP90B1;HSPA4;HSPD1;HYI;IFI44;IFIT1;IFIT2;IFIT3;IFT52;IGF1;IGFBP7;IL11;IL27RA;IL6;ILVBL;IMPDH1;INPP5F;INTS6;IP6K2;IPO8;IPO9;IQCD;IQGAP3;IRF2BPL;ISG15;ISG20;IST1;ITGA1;ITGA3;ITGA6;ITGB4;JUP;KANK2;KAT2A;KCND1;KCNE1;KCNJ2;KCNN4;KDELR1;KDM2B;KIAA1522;KIF2A;KIF2C;KIF4A;KIF5B;KLF13;KLHDC4;KLHL3;KLHL42;KLK12;KNCN;KRAS;KRT75;L1CAM;LARP4;LASP1;LCA5L;LCP1;LEMD3;LETM1;LGALS1;LGALS3BP;LHX4;LIFR;LIMS1;LIN7C;LIPC;LMNB1;LNPEP;LONP2;LRP1;LRRC59;LRRC8A;LRRC8B;LRRC8C;LRRC8D;LRWD1;LZTFL1;MACROD1;MAD2L1;MAGEB3;MAN1B1;MAP4K2;MARCKS;MAST4;MATR3;MCAM;MCM2;MCM3;MCM4;MCM5;MCM6;MCM7;MDC1;MECR;MEF2A;MET;MFN2;MFSD10;MIER1;MINPP1;MKI67;MMD;MME;MMS22L;MOB4;MOBP;MON2;MOV10;MPDU1;MPL;MPRIP;MRC2;MRE11;MRFAP1;MRPL19;MRPS27;MSH2;MSH6;MT-CO1;MT-ND1;MTHFD2;MTHFS;MTMR12;MTX1;MXD4;MYEF2;MYO18A;MYO1A;MYO1B;MYO3B;MYOCD;NAB1;NAIP;NAT14;NBL1;NCAPD3;NCAPG;NCS1;NDUFS1;NDUFS4;NELFCD;NETO2;NFAT5;NIFK;NOC2L;NOP53;NOTCH2;NOTCH3;NPTN;NR5A2;NRP1;NSUN4;NT5C1B;NT5C1B-

			RDH14;NT5E;NUAK1;NUDT21;NUP160;NUP210;NUP50;NXN;NXPH2;NXT2;OARD1;OASL;OAT;OCIAD2;ORC3;ORMDL2;OSBPL10;OSBPL7;OSTF1;OXCT1;OXTR;P3H3;P3H4;PACS2;PACSIN3;PAFAH1B3;PARD6B;PARVA;PAX3;PAXBP1;PCDH7;PDCD10;PDCD4;PDE12;PDIA3;PDLIM7;PFDN1;PGD;PGM2;PGRMC2;PHIP;PHLDB2;PI16;PICALM;PIGS;PIGT;PIK3CA;PIM1;PIR;PKD1L1;PLAGL2;PLCB3;PLCXD2;PLEKHB2;PLEKHG2;PLEKHH2;PLGRKT;PLK2;PLPP2;PLS1;PLS3;PLXDC2;PLXNA4;PLXNB2;PNN;PNP;POGK;POLA2;POLD1;POLR2I;POLR2K;POLRMT;POM121;POM121C;PPA2;PPARG;PPIA;PPIB;PPM1H;PPP1R16A;PPP2R1B;PPP2R2A;PPP2R5A;PPP4R3A;PQBP1;PRDX2;PREX1;PRIMA1;PRKAG1;PRKCE;PRKG2;PROCR;PRR14L;PRSS21;PSAT1;PSG3;PSG6;PSG9;PSIP1;PSMA7;PSME1;PSMG1;PTAR1;PTBP1;PTBP2;PTMA;PTMAP7;PTPMT1;PTPN1;PTPN22;PTPRD;PTPRF;PWP1;PXDN;PXYLP1;PYGB;QSER1;RAB11FIP2;RAB27B;RAB30;RAB34;RAB39B;RAB5C;RABEPK;RABGAP1L;RABL2A;RABL2B;RALB;RAP1B;RAPGEF2;RARB;RASSF1;RASSF5;RBBP5;RBM12B;RBM28;RBM39;RBM42;RBM47;RCC2;RCOR2;REM2;RFC2;RFC5;RFT1;RGN;RGP1;RGS17;RHOC;RIMS2;RIMS4;RIN1;RIPK2;RNF138;RNF213;RPP40;RPRD2;RRBP1;RRM1;RRP36;RSF1;RSRC2;RXFP1;S100A11;S100A16;S100G;SAC3D1;SALL2;SAMD15;SCAF11;SCN3A;SDC4;SDR39U1;SEC11C;SEC16A;SEC23IP;SEC61A1;SEC62;SELENOH;SEMA4D;SEMG2;SEPT6;SERP1;SERPINB5;SFI1;SFN;SFXN1;SGK3;SH2D4A;SH3BGRL3;SH3PXD2B;SH3TC2;SHE;SHTN1;SIGMAR1;SIN3A;SLBP;SLC16A9;SLC25A1;SLC25A10;SLC25A19;SLC25A22;SLC25A30;SLC26A7;SLC27A4;SLC29A1;SLC35A5;SLC39A14;SLC39A3;SLC44A1;SLC8A1;SMARCA1;SMARCA2;SMARCB1;SMARCC1;SMC4;SMIM14;SMKR1;SNAI2;SNAPIN;SNRNP35;SNX6;SOS2;SOX5;SOX6;SOX9;SP1;SPC24;SPERT;SPINK1;SPRED1;SPRY2;SPTLC3;SREK1;SRF;SRGAP1;SRI;SRP19;SRRM1;SRSF4;SRSF5;SRSF6;SRSF7;SRSF9;SRXN1;SSNA1;SSR1;STAMBP;STK24;STK35;STX12;STX6;STXBP3;SUCLA2;SUGP1;SULT1B1;SUSD1;SYMPK;SYNE1;SYNE2;SYNPO2L;SYPL1;SYTL2;TAGLN2;TAT;TBC1D9;TBCD;TDP1;TELO2;TFAM;TH;THAP2;THBS1;THY1;TIGD4;TIMP3;TLR4;TM4SF1;TMCC1;TMEM106C;TMEM68;TMEM87A;TMOD3;TMSB4X;TMX1;TNKS1BP1;TNKS2;TNS4;TOX4;TPD52L2;TPM1;TPM2;TPM3;TPM4;TPSD1;TRA2B;TRAPPC3;TRIM2;TRIM24;TRIM26;TRIM56;TRIM9;TROVE2;TRPA1;TRPM4;TRPM6;TRPS1;TSHR;TSPAN1;TSPAN19;TSPAN4;TTC17;TTC37;TUBB2B;TWF1;TWF2;TWIST1;UBA6;UBAC2;UBE2I;UBR5;UBTF;UGGT1;UGGT2;UGT8;UHMK1;UHRF1;UNC119B;UNC13D;UNC45A;UNC93B1;USP33;UST;UTRN;VAMP7;VASP;VEGFA;VMP1;VPS53;VSTM5;WASF2;WBP2;WDFY1;WDR33;WEE1;WLS;XPNPEP3;XPO6;XPOT;YTHDC1;YTHDF2;YWHAQ;YWHAZ;ZBED3;ZBTB20;ZBTB6;ZBTB9;ZC3H11A;ZCCHC3;ZNF185;ZNF207;ZNF215;ZNF264;ZNF280C;ZNF326;ZNF384;ZNF48;ZNF561;ZNF568;ZNF579;ZNF622;ZNF638;ZNF79;ZNF799
hsa-miR-92b-3p-477823_mir	-0.689	0.402	AAED1;ABCA3;ABCF2;ABR;ACAA1;ACOD1;ACTC1;ACTN4;ADAM10;ADAT1;AEN;AGBL5;AGMAT;AKAP10;AKAP8;ALDOA;ALG14;AMD1;AMIGO1;ANKIB1;ANKRD52;ANP32E;AP1B1;AP2A2;AP3S2;AP5Z1;APOBEC3F;APOLD1;APPL1;ARF1;ARFGEF2;ARGFX;ARID1B;ARL6IP4;ARNTL2;ASF1B;ASGR2;ATF7IP;ATOX1;ATP13A1;ATP2A2;ATP2B4;ATP5G3;ATP7A;ATXN1;AURKA;B4GALT7;BAG6;BAK1;BAZ2B;BBX;BCAT1;BCAT2;BCKDK;BCL11B;BCL2L11;BICD2;BMP8A;BMPR1A;BMPR2;BPTF;BRD8;BRMS1L;BTG2;C11ORF24;C15ORF38-AP3S2;C17ORF75;C1GALT1C1;C1ORF35;C21ORF91;C2ORF69;C5ORF24;C6ORF62;C9ORF64;CAMSAP1;CAPN15;CAPRIN1;CAPZB;CARD6;CASD1;CASKIN1;CCDC113;CCDC171;CCDC186;CCDC22;CCNB1;CCNQ;CCSER2;CD180;CD226;CD2AP;CD69;CDC27;CDC5L;CDC6;CDK16;CDK5R1;CDKN1C;CEP152;CHST1;CIC;CIDEC;CISD1;CIT;CLN8;CLTA;CNEP1R1;CNIH1;CNNM4;CNOT2;CNOT4;COG3;COPA;COQ8B;COX20;CPEB2;CPEB3;CPEB4;CPTP;CREB3L2;CRIM1;CSDE1;CTC1;CTDSPL;CYP20A1;CYP2C19;CYTH2;DAB2IP;DAND5;DBT;DDI2;DDIT4;DDX3X;DENND2C;DENND4A;DENND4B;DEXI;DKK3;DLST;DNAAF5;DNAJB12;DNAJB9;DNAJC27;DNAJC30;DOCK11;DOCK9;DSTYK;DUS2;DUSP10;DUSP5;DYNC1LI2;DYNLT3;E2F3;EARS2;EDEM1;EDF1;EDRF1;EFNB1;EID2B;EIF1;EIF2AK1;EIF2B2;EIF3A;EIF4EBP1;EIF4EBP2;EIF5A2;ELOA;ENTHD1;EPM2AIP1;ERGIC2;ERICH1;ESRP1;EVI5;EXOC5;EZH2;F11R;FAM126B;FAM129A;FAM135A;FAM3C;FAM46A;FAM49A;FAM91A1;FAR1;FASLG;FASN;FBN2;FBXO21;FBXO31;FBXW2;FCHO2;FGF2;FJX1;FKBP14;FKBP1A;FKBP4;FKBP9;FLCN;FLNA;FLNB;FMN1;FNDC3B;FNIP1;FOPNL;FOXN2;FOXN3;FUT10;FUT11;FXR1;FZD6;G2E3;G3BP2;G6PD;GAA;GALNT7;GAN;GATA6;GATAD2A;GATAD2B;GCNT3;GEMIN2;GFPT2;GGCX;GID4;GIT2;GLOD4;GLYR1;GM2A;GNAI2;GNAQ;GNB2;GOLGA3;GOLGA4;GOLGA8A;GOLGA8B;GOLGA8IP;GOLGA8J;GPBP1L1;GPR55;GPX1;GRAMD1B;GRAMD2B;GRAMD4;GRB2;GRHPR;GSS;GSTM3;GTF2A1;GTF2E1;GUF1;GULP1;GXYLT1;H3F3B;H3F3C;HAGH;HECTD1;HIST1H2AM;HIST1H2BF;HIST2H2AC;HIST2H4B;HIVEP1;HMGA2;HMGCR;HOXA13;HOXC8;HP1BP3;HPS6;HSPA1B;IARS;IARS2;IBTK;ICAM1;IFIT3;IFITM1;IFT22;IGSF8;IK;IKZF2;IL6ST;ILF3;INCENP;INSIG1;IPP;IRGQ;ITGA6;ITGAV;ITGB8;ITPR1;JOSD1;KAT2B;KCNC4;KDM2A;KDM3A;KEAP1;KIAA0556;KIAA1109;KIAA1586;KIAA1958;KIF1B;KIF1BP;KIF20B;KIF5B;KLHDC10;KLHL14;KLHL15;KLHL18;KLHL42;KMT2D;KMT5B;LAMP2;LARS;LAX1;LCOR;LDLR;LETM1;LHFPL2;LILRA2;LONRF3;LTBP2;LUC7L3;LYST;MAFK;MAN2A1;MAP1B;MAP2K4;MAP3K2;MAPK1;MAST3;MBD2;MBNL1;MCF2L2;MCL1;MCOLN2;MDK;MDM2;MED19;MED29;MED7;MEF2D;METRN;MEX3B;MFF;MFN1;MIA3;MKNK2;M

			MS19;MOAP1;MORC3;MPHOSPH10;MRO;MRPL19;MRPL39;MRPL53;MRPS16;MRPS21;MRPS23;MRS2;MSH3;MT-CO1;MTF1;MTMR1;MTMR10;MUC21;MYC;MYH10;MYLIP;MYO1D;MYO5A;MYZAP;NABP2;NACC2;NARF;NCAPD2;NCAPG2;NCL;NECAP1;NEMP1;NF2;NFATC2IP;NFYB;NIPA2;NKAP;NLK;NLRP9;NOL4L;NONO;NPM1;NPTN;NPY4R;NRAS;NSF;NUCKS1;NUDT19;NUFIP2;NUGGC;NUP43;OGFR;OPA3;OR2A4;OR6V1;ORAI2;ORAOV1;ORC1;OSMR;OTUD3;OTUD7B;PA2G4;PABPN1;PAFAH1B1;PAIP1;PAPD5;PAPD7;PARD6B;PARK7;PARVG;PAWR;PAX9;PBLD;PCBD2;PCMTD1;PDIK1L;PDZD8;PEAK1;PELP1;PER2;PFKFB4;PFN1;PGAM1;PGAM4;PGPEP1;PHF19;PHF23;PHLPP2;PHRF1;PHTF2;PIGO;PIK3AP1;PIK3CD;PIN4;PIP5K1C;PITPNA;PKNOX1;PLA2G12A;PLEKHA1;PLXNA3;PLXND1;PMEPA1;PNO1;POFUT1;POLK;POM121;PPCS;PPIC;PPIL1;PPP1CB;PPP1R12C;PPP1R37;PPP1R3D;PRKCSH;PRMT5;PRPS1;PRR5;PRRC2A;PRRC2B;PRRG4;PSMA7;PSMB3;PSMD3;PSMD5;PSME3;PTAR1;PTBP1;PTEN;PTGER4;PTGES2;PTPRJ;PURG;PWP1;PYCR2;QSER1;RAB23;RAB3D;RAB7B;RAB8B;RAD21;RAD51;RAN;RANBP6;RBBP5;RBFOX2;RBL2;RBM10;RBM14;RBM27;RBM28;RBM8A;RBMS2;RBPJ;RECK;REL;RERE;REV3L;REXO1;REXO2;RLIM;RNF103;RNF168;RNF181;RNF4;RNF44;RPAP1;RPL23;RPL24;RPL3;RPL8;RPL9;RPLP0;RPRD2;RPS14;RPS28;RPS3A;RPS4X;RRN3;RRP1;RSBN1;RTKN2;SASH1;SCAF1;SCAF11;SELENOT;SERTAD2;SERTAD3;SESN3;SETD5;SFPQ;SFXN1;SGK3;SGPP1;SH2B3;SH3PXD2A;SHE;SIK1;SIN3A;SKI;SLC10A7;SLC12A5;SLC15A1;SLC25A16;SLC25A32;SLC25A36;SLC25A4;SLC25A5;SLC33A1;SLC37A3;SLC39A14;SLC39A6;SLC7A1;SLC7A11;SLC9A1;SLCO4A1;SLX4;SMAD3;SMAD6;SMAD7;SMAP2;SMARCA5;SMARCC2;SMG1;SMU1;SNN;SNRNP70;SNRPD1;SNX10;SOGA1;SOX11;SOX4;SPATS2L;SPC24;SPCS3;SPOCK2;SPRYD4;SRFBP1;SRP68;SRPRA;SSFA2;SSRP1;STAT2;STRBP;SUPT5H;SUPT7L;SURF6;SYMPK;SYNGAP1;SYNJ1;SZRD1;TACC1;TAF12;TAF8;TANK;TATDN3;TBC1D8;TCOF1;TCP11L1;TECPR2;TEF;TESK1;THAP10;TIRAP;TLN1;TLR3;TMEM115;TMEM184B;TMEM239;TMEM33;TMEM41A;TMEM44;TMF1;TNFRSF13C;TNPO1;TNPO2;TOB1;TOB2;TOR1B;TOR4A;TPPP;TPT1;TRAFD1;TRAM2;TRIM36;TRIO;TRMT2B;TSC1;TSPAN31;TSPYL1;TUBB6;TULP4;TWF1;TXLNA;TXNDC15;U2AF2;UBE2Q2;UBE2Z;UBXN4;UCK2;UGDH;UHRF1BP1;UNC5B;UQCRFS1;UROD;USF1;USP28;UVRAG;VDAC2;VHLL;VMA21;VPS4B;VPS54;WARS;WASL;WDR81;WDR82;WRNIP1;XKR7;XPO5;XPO7;XPR1;XRCC1;XRN1;YIPF4;YWHAE;ZADH2;ZBTB34;ZBTB48;ZBTB8B;ZC3HAV1L;ZDHHC21;ZDHHC24;ZDHHC5;ZFC3H1;ZFP62;ZFYVE21;ZIC5;ZNF134;ZNF157;ZNF17;ZNF224;ZNF24;ZNF264;ZNF267;ZNF277;ZNF282;ZNF317;ZNF354B;ZNF383;ZNF417;ZNF430;ZNF460;ZNF492;ZNF598;ZNF607;ZNF695;ZNF721;ZNF75A;ZNF772;ZNF850;ZNF98;ZNRF3;ZSCAN12;ZSCAN18
hsa-miR-21-5p-477975_mir	-2.669	0.414	ABCB1;ABCD3;ACAT1;ACBD5;ACTR2;ADGRG2;ADNP;AFTPH;AGGF1;AGO2;AGO4;AHSA2;AKAP9;AKT2;ALMS1;ANKRD28;ANKRD46;ANP32A;AP1AR;AP3M1;APAF1;APC;APOLD1;APPL1;ARHGAP21;ARHGEF12;ARID4A;ARMCX3;ASRGL1;ATAD2B;ATF2;ATF7IP;ATMIN;ATP11B;ATP2B4;ATRX;ATXN10;AUTS2;B3GALNT1;B3GNT5;BASP1;BAZ1B;BCAT1;BCL10;BCL2;BCL6;BCL7A;BDH2;BMI1;BMPR2;BNIP2;BOC;BRCA1;BTBD3;BTBD7;BTG2;BTN3A3;C15ORF52;C1ORF147;C20ORF194;C8ORF17;CADM1;CALD1;CAPRIN1;CASC2;CASP8;CBX4;CCDC14;CCDC34;CCL1;CCL20;CCNG1;CCR1;CCR7;CCT6P1;CD47;CDC25A;CDK19;CDK2AP1;CDK6;CEBPB;CENPQ;CEP152;CEP97;CERS6;CKAP5;CLCN5;CLIP4;CLOCK;CLU;CNTRL;COBLL1;COL4A1;COL5A2;CORO2A;COX20;CPEB3;CRYBG1;CSNK1A1;CXCL10;CYBRD1;CYP4V2;DAAM1;DAXX;DCAF10;DCAF11;DCAF8;DCP1A;DDAH1;DDHD2;DDR2;DDX3X;DDX46;DDX55;DERL1;DICER1;DLG1;DMD;DMTF1;DNAJC16;DNM1L;DOCK10;DOCK4;DOCK5;DOCK7;DSE;DTX3L;DUSP10;DUSP8;DYNC1LI2;E2F1;E2F2;E2F3;ECI2;EDIL3;EDRF1;EGFR;EGLN1;EIF1AX;EIF2S1;EIF4A2;EIF4EBP2;EIF5;ELAVL4;ELOVL4;ELOVL7;ENAH;EPHA4;EPM2A;EPM2AIP1;ERBB2;ERP44;ESYT2;ETNK1;EXOC5;EXOC8;FAM126B;FAM136A;FAM20B;FAM217B;FAM3C;FAM46A;FANCI;FAS;FASLG;FAXDC2;FBXL13;FBXL17;FBXL2;FBXO11;FBXO3;FDXACB1;FERMT2;FGF12;FGFRL1;FIGN;FILIP1L;FKBP5;FMOD;FMR1;FNBP1;FOXN2;FOXN3;FOXO1;FOXO3;FOXP3;FRS2;FUBP1;FZD6;GAPVD1;GAS5;GDF5;GID4;GK5;GLCCI1;GLG1;GNAQ;GNB4;GNE;GOLGA4;GP5;GPAM;GPD1L;GPD2;GRPEL2;GTF2A1;GTF2I;GXYLT2;HAPLN1;HECTD1;HERPUD2;HIC2;HIF1A;HIPK3;HMGB1;HMGB3;HNRNPH1;HNRNPK;HOXA9;HPGD;HPS5;HS3ST3B1;ICAM1;ICOSLG;IGF1R;IL12A;IL1B;IPP;IRAK1;IREB2;ISCU;ITGB8;ITSN2;IVNS1ABP;JADE1;JADE3;JAG1;JMY;JPH1;KANSL3;KAT6A;KBTBD6;KBTBD7;KIAA1551;KIFAP3;KLF5;KLF9;KLHL15;KLHL24;KLHL42;KLK2;KNL1;KRIT1;LAMP2;LARS;LATS1;LCORL;LDAH;LIFR;LIMCH1;LIN7C;LMBR1;LNPK;LONRF2;LPGAT1;LRP6;LRRC57;LRRFIP1;LYRM7;MALT1;MAP2K3;MAP3K1;MAP3K2;MARCKS;MBNL1;MDM4;MED9;MEF2A;MEF2C;MEGF9;MEIS1;MGA;MGAT4A;MIB1;MKNK2;MMP2;MMP9;MOAP1;MON2;MORC3;MOXD1;MPP5;MRAP2;MRPS10;MSH2;MSH6;MSLN;MT-CO2;MTAP;MTMR12;MTMR9;MTPN;MUC1;MYC;MYCBP2;MYD88;MYEF2;MYO9A;NAA30;NAA50;NAV3;NBEA;NCAPG;NCOA3;NCSTN;NEK1;NETO2;NFAT5;NFIA;NFIB;NFKB1;NIN;NIPBL;NKTR;NR2C2;NSD2;NSD3;NSUN2;NT5C2;NTF3;NUBPL;NUDT19;NUFIP2;OLR1;ORC4;OSBPL1A;OSBPL3;OSR1;OTUD1;OXTR;PAG1;PALLD;PAN3;PARP1;PARP9;PATE2;PBRM1;PBX1;PCBP1;PCGF2;PDCD4;PDGFD;PDHA2;PELI1;P

			ER2;PER3;PFKFB2;PGRMC2;PHACTR2;PHF20;PHF20L1;PHIP;PHTF1;PIAS3;PIGN;PIGX;PIK3C2A;PIK3R1;PITHD1;PKD2;PKNOX1;PLAT;PLD1;PLEKHA1;PLEKHA2;PLEKHA8;PLOD3;PLPP1;PM20D2;POLR3B;PPARA;PPFIA4;PPIF;PPM1L;PREPL;PRICKLE2;PRKAB2;PRKCE;PROSER1;PRPF39;PRR14L;PRRC1;PSMD9;PTAR1;PTBP3;PTEN;PTGFR;PTK2;PTPDC1;PTPN14;PTPN3;PTX3;PURA;PURB;PURG;PYM1;RAB11FIP2;RAB22A;RAB6A;RAB6C;RABGAP1;RAI14;RALGPS2;RAPGEF6;RAPH1;RASA1;RASEF;RASGRP1;RASGRP3;RB1;RDH11;RECK;REST;REV1;REV3L;RFFL;RHO;RHOB;RHOQ;RMND5A;RNF103;RNF11;RNF111;RNF185;RNF6;RPRD2;RPS6KA3;RPS7;RRAGC;RSF1;RSPRY1;RTN4;RUFY3;SACM1L;SAMD5;SAR1A;SASH1;SATB1;SCAF11;SCRN1;SEC63;SECISBP2L;SEMA5A;SEPT2;SERAC1;SERPINB5;SERPINI1;SESN1;SESTD1;SET;SETD1B;SETD2;SFXN1;SGCB;SGK3;SGTB;SIRT2;SKP2;SLAIN2;SLC16A10;SLC17A5;SLC26A2;SLC31A1;SLC5A3;SLC9A6;SLK;SLMAP;SMAD7;SMARCA4;SMC1A;SMN1;SMNDC1;SNRK;SNRNP48;SNX30;SOCS1;SOCS4;SOCS5;SOCS6;SOD3;SOWAHC;SOX2;SOX5;SP1;SPATS2L;SPG11;SPIN1;SPPL3;SPRY2;SPRY4;SPTLC3;SREK1;SRPK2;SRSF11;SSFA2;ST6GAL1;STAG2;STAT3;STRBP;STUB1;STXBP5;SUZ12;SYNE2;TAF1;TAF5;TAP1;TBL1XR1;TCEANC2;TCF21;TESK2;TET1;TGFB1;TGFB2;TGFBI;TGFBR2;TGFBR3;TGIF1;THOC2;TIAM1;TICAM2;TIMP3;TLR3;TLR4;TM9SF3;TMEM147;TMEM170A;TMEM2;TMEM245;TMEM56;TMX4;TNFAIP3;TNFRSF10B;TNFRSF11B;TNPO1;TNRC6B;TNS3;TOP2A;TOPORS;TOR1AIP2;TP53BP2;TP63;TPM1;TPRG1L;TRAF7;TRAPPC2;TRIM2;TRIM33;TRIM38;TRIM59;TRPM7;TSHZ3;TSNAX;TTC33;TUBGCP5;TXLNGY;UBE2N;UBR3;UBR5;UGGT1;UQCRB;USP34;USP47;USP7;UTRN;VASH2;VEGFA;VHL;VPS13A;VPS26A;VPS36;VPS54;WDR7;WFS1;WNK1;WNK3;WNT5A;WSB1;WWC2;WWP1;YME1L1;YOD1;ZADH2;ZBTB20;ZBTB38;ZBTB47;ZBTB8A;ZCCHC3;ZFYVE16;ZMYM2;ZNF207;ZNF217;ZNF292;ZNF326;ZNF35;ZNF367;ZNF460;ZNF532;ZNF587;ZNF667;ZRANB1;ZYG11B
hsa-miR-130a-3p-477851_mir	0.779	0.420	ABCD2;ABCG8;ACBD5;ACP6;ACSL4;ACVR1;ADARB2;ADM2;AGO3;AKIRIN2;ANKRD50;ANKRD9;APP;ARHGAP1;ARHGAP12;ARHGEF26;ARL17B;ARL6IP1;ARSA;ASB16;ATF7IP;ATG2B;ATMIN;ATP6V0D1;ATP6V0E1;ATP6V1B2;ATP6V1C1;ATXN1;BLCAP;BMP3;BMPR2;BRWD1;BTBD3;BTF3L4;BTG1;C11ORF57;C16ORF45;C16ORF70;C3ORF18;C4ORF36;CALM2;CAMSAP2;CAPRIN2;CBY1;CCDC137;CCNA2;CCND2;CCR6;CCT6A;CDADC1;CDCA4;CDK19;CDK2AP2;CDK4;CEP170;CEP55;CERCAM;CFL2;CHEK2;CHERP;CHIC1;CINP;CLCN3;CLEC12B;CLIC4;CLIP1;CNOT4;COX10;COX20;CSF1;CSNK2A1;CTSA;CUL3;CXORF21;CYB5D1;CYP20A1;DAD1;DAPK1;DCBLD2;DCUN1D3;DDX6;DEPDC1;DICER1;DLC1;DLG5;DLL4;DNM2;DPYSL2;DSTYK;DTX4;DUSP18;DYNC1LI2;EDN1;EGLN3;ELL2;ENPP4;ENPP5;EOGT;ERBIN;EREG;ESR1;F2RL1;FAM114A1;FAM120AOS;FAM217B;FBXO28;FGFR1OP;FIG4;FKTN;FLYWCH2;FOXQ1;FUT11;FXR1;FZD6;G6PC;GALNT4;GJA1;GMFB;GNPTAB;GP2;GPR161;GPR75;GPR82;GPRC5A;GRB10;GRSF1;HABP4;HADHB;HBP1;HOXA10;HOXA5;HOXB3;HOXD11;HPRT1;HSPA8;IER3IP1;IFITM1;IFNLR1;IGF2R;IGFBP5;IL18;IL23R;IPMK;IRF1;JARID2;JMY;KBTBD6;KCNB1;KCTD10;KDELR1;KIAA1191;KIF13A;KLF4;KLF6;KLHL21;KLHL36;KLRD1;KREMEN1;LCLAT1;LDLR;LEFTY1;LILRA2;LIPA;LMLN;LZIC;MAFB;MAP3K12;MAP3K9;MAP7;MAPK1;MAPKAPK5;MAPRE3;MASTL;MAVS;MB21D2;MBNL1;MBNL3;MCC;MECP2;MED18;MED8;MEOX2;MET;MFF;MID1IP1;MIGA2;MLEC;MMGT1;MOCS2;MREG;MRPL52;MSANTD4;MSMO1;MTPN;MYC;MYH11;MYLIP;NAA50;NABP1;NACC2;NARS;NCAPD2;NF2;NFE2L1;NFIB;NIP7;NIPA1;NKAP;NOM1;NPTX1;NRBF2;NUS1;ODF4;OMD;ORC1;OSBP;OTUD3;PAFAH1B2;PAPD4;PARP1;PDGFRA;PDP2;PDRG1;PDZD11;PEX13;PHF12;PIGA;PIGG;PLA2G12A;PLEKHF2;PLEKHS1;PNRC1;POC1B-GALNT4;POGZ;POLR1B;POLR2D;POP7;PPARA;PPARG;PPARGC1A;PPIG;PPP1R14C;PPP1R15B;PPP6R1;PPP6R3;PRKAA1;PRNP;PRPF38A;PRPF4;PRR23A;PRRG4;PRUNE2;PSMB5;PTEN;PTGR2;PTPN4;PTPRG;PURG;PXK;QKI;QSOX1;RAB11FIP1;RAB14;RAB34;RAB5A;RAB5B;RACGAP1;RAG1;RAN;RBM20;RBM23;RBM27;RBM43;RDH11;RFC2;RFT1;RFX7;RFXAP;RLIM;RNF11;RNF125;RNF149;ROMO1;RPF2;RPRD2;RPS15A;RPS27A;RPS6KA5;RRAGD;RUNDC1;RUNX3;S1PR2;SALL3;SAMD8;SATB2;SEC16A;SEC23B;SECISBP2L;SERINC3;SF3A1;SFTPA1;SIGLEC9;SIK1;SLAIN1;SLC10A3;SLC12A7;SLC31A1;SLC35E2B;SLC35E3;SLC38A2;SLC38A9;SLC46A1;SMAD4;SMAD5;SMOC1;SMTNL2;SNAPIN;SNTB1;SNTB2;SNX5;SOX4;SOX5;SPART;SRSF2;SSTR2;STARD13;STK38L;STX16;STX6;SUN2;TAC1;TAOK1;TCF7L2;TERF2;TGFB1;TGFBR1;TGFBR2;THAP6;THRA;TIMM50;TMCO1;TMEM2;TMEM30A;TMOD3;TMTC1;TNF;TNFRSF10B;TNRC6A;TNRC6B;TPP1;TRIM37;TRIM4;TRIM71;TROVE2;TRPC3;TSPAN3;TXNIP;UBB;UBBP4;UBC;UBE2D2;UBE2D3;UBN2;UQCRB;USP13;USP32;VPS37A;VPS37B;WASL;WDR31;WIPF2;WNK3;WNT10A;XIAP;YY1;ZBTB18;ZBTB4;ZBTB7A;ZBTB7B;ZBTB8A;ZFYVE26;ZFYVE9;ZIC5;ZMAT3;ZNF107;ZNF12;ZNF154;ZNF224;ZNF24;ZNF317;ZNF354B;ZNF417;ZNF431;ZNF529;ZNF620;ZNF678;ZNF711;ZNF800
hsa-miR-26b-5p-478418_mir	-2.534	0.420	AAMDC;ABCA1;ABCA6;ABCB6;ABCD1;ABCD4;ABCF1;ABCG2;ABCG4;ABHD2;ACAA2;ACADM;ACADS;ACADSB;ACBD3;ACBD5;ACE2;ACOD1;ACOX2;ACSF2;ACSL3;ACTR5;ACTR8;ACVR1B;ADAM17;ADAM18;ADAM29;ADAM9;ADAMTS1;ADAMTS12;ADAP2;ADARB1;ADGB;ADGRE5;ADGRG6;ADGRG7;ADGRL2;ADI1;ADM;ADM2;ADNP;ADPGK;ADRA2A;AEN;AFAP1;AGL;AGMAT;AGPAT3;AGPAT5;AGT;AGTPBP1;AGTR1;AIFM1;AK4;AKAP5;AKIRIN1;AKT1;ALDH6A1;ALG1;ALG3;ALG9;ALK

			BH4;ALPI;ALPK3;ALX1;AMDHD2;ANAPC1;ANAPC13;ANKRD36B;ANKRD36BP1;ANKRD46;ANKRD52;ANO2;ANXA1;ANXA3;ANXA5;ANXA8;ANXA8L1;AOX1;AP1S3;AP3M2;APBB2;APOF;APOO;AQR;ARAP2;ARF1;ARFIP2;ARFRP1;ARHGAP44;ARHGEF39;ARHGEF5;ARID3B;ARL17A;ARL4A;ARL4C;ARL5A;ARL8B;ARMC1;ARMC7;ARMC8;ARMCX2;ARNT2;ARNTL2;ARPC5;ARPP19;ARRB1;ASF1B;ASNA1;ASNS;ASPM;ASPN;ASTN2;ASXL3;ATAD2B;ATAT1;ATF3;ATP1A1;ATP1A3;ATP2A2;ATP2B1;ATP6AP1;ATP6AP2;ATP6V0D1;ATP6V1A;ATP7B;ATXN2L;AUP1;B3GALT1;B3GALT5;BABAM1;BACE1;BAG3;BAHD1;BATF;BATF3;BCHE;BCL11B;BCL3;BCL7B;BCLAF1;BEND4;BGLAP;BHLHE40;BID;BLNK;BLOC1S2;BLOC1S5;BLVRB;BMP2;BMP2K;BMP8B;BMPR1A;BMPR2;BNC1;BNC2;BOLA2;BOLA2B;BORCS6;BRCA1;BRD3;BRD4;BRD8;BRDT;BRSK2;BTG1;BTG2;BTN3A3;BUD31;C11ORF16;C14ORF2;C14ORF37;C16ORF70;C17ORF53;C18ORF25;C1D;C1GALT1C1;C1ORF216;C1ORF50;C1QB;C1QTNF1;C3;C5AR1;C6ORF15;C7ORF55-LUC7L2;C8ORF33;C9ORF16;CA1;CA11;CA2;CA9;CAAP1;CAB39L;CACNA1S;CACNB2;CACNB4;CACNG3;CADM1;CAMK4;CAMKMT;CAPN9;CAPZA1;CARD14;CARS2;CASD1;CASP3;CASP4;CASP7;CASP8;CASP9;CASQ1;CATSPERB;CAV1;CAV2;CBLC;CBLL1;CBX6;CBY1;CCDC144A;CCDC15;CCDC170;CCDC181;CCDC25;CCDC28A;CCL2;CCL27;CCL7;CCNB1IP1;CCND1;CCND2;CCNDBP1;CCNE1;CCNI;CCNL2;CCR6;CCSER2;CCT7;CD248;CD28;CD36;CD55;CDC14B;CDC25B;CDCA3;CDH1;CDH5;CDH9;CDIPT;CDK14;CDK18;CDK19;CDK2;CDK2AP2;CDK5R1;CDK6;CDK9;CDKL2;CDKN2D;CDV3;CELA3B;CEMP1;CEND1;CENPQ;CEP83;CEP85;CERK;CES2;CES3;CFI;CH25H;CHAC1;CHAF1A;CHD9;CHEK2;CHERP;CHM;CHMP3;CHMP6;CHMP7;CHORDC1;CHPF;CHST12;CHST15;CHST4;CHST7;CIAO1;CIB1;CISH;CKS2;CLEC2B;CLIC2;CLINT1;CLOCK;CLSTN1;CLSTN2;CLTA;CMC2;CMC4;CMPK1;CMTM6;CNIH4;CNNM2;CNOT4;CNPY3;CNTFR;CNTLN;CNTN1;CNTRL;COA4;COASY;COL12A1;COL15A1;COL1A2;COL4A2;COL4A4;COL4A5;COL5A1;COLEC12;COMMD3;COPZ2;COQ2;COQ9;COX5A;COX7A2L;COX8A;CPA1;CPA4;CPB2;CPM;CPN2;CPSF2;CPSF7;CRADD;CREBRF;CREBZF;CREG1;CRELD1;CRIM1;CRIP2;CRLF1;CRTAM;CRYAA;CRYGC;CRYL1;CRYZ;CSGALNACT2;CSN3;CSNK1A1;CSNK1G1;CSPG5;CST2;CST3;CSTF2;CTDNEP1;CTGF;CTH;CTNS;CTSD;CTSV;CTSZ;CXADR;CXCL13;CXCL6;CXCL9;CXCR1;CXCR4;CXCR6;CXORF38;CYB561D2;CYB5R4;CYBRD1;CYLC2;CYP11B2;CYP24A1;CYP27B1;CYP2D6;CYP2D7;CYP2F1;CYP4A11;CYP4A22;CYP4F11;CYP4F8;CYR61;CYTH1;CYTH2;CYTIP;DAB2;DBNL;DCAF10;DCAF16;DCAF8;DCBLD2;DCHS2;DCP1A;DCSTAMP;DCTD;DCTN3;DCTN4;DCTPP1;DCUN1D2;DDB2;DDIT4;DDX25;DDX3X;DDX6;DDX60;DEFA1;DEFA3;DEFB4A;DEFB4B;DEPDC1;DERA;DERL1;DFNA5;DGAT1;DGKI;DGKZ;DHODH;DHRS11;DHX30;DHX35;DIABLO;DIP2A;DIS3;DLGAP4;DMAC2;DMXL2;DNAJA2;DNAJA3;DNAJB4;DNAJC11;DNAJC12;DNAJC2;DNAJC6;DNAL4;DNMBP;DOCK10;DONSON;DPF3;DPM1;DPPA4;DPY19L1;DPYSL3;DRD3;DSC1;DSCC1;DTL;DTNB;DUSP1;DUSP12;DUSP14;DUSP21;DUSP22;DUSP7;DUT;DVL3;DYNC2LI1;DYRK1A;DYRK3;E2F7;EAF2;EBNA1BP2;ECH1;ECT2;EDEM3;EDN2;EED;EEF1A1;EFCAB14;EFNA2;EFNA4;EGR3;EHD1;EI24;EID2B;EIF1AX;EIF1AY;EIF2AK3;EIF2B1;EIF4A1;EIF4A3;EIF4G2;EIF5;ELAVL2;ELAVL3;ELF4;ELOA2;ELOVL2;ELP1;EMC3;EMC6;EMC7;EMP3;EMSY;ENDOG;ENOSF1;ENOX1;ENTPD7;EP300;EP400;EPB41L3;EPHA2;EPHX1;EPPIN;ERAP1;ERAP2;ERBIN;ERC1;ERCC6L;ERCC8;ERI2;ETF1;ETV4;EVI2A;EXOC6B;EXT2;EZH2;F13B;FADS2;FAF1;FAHD2A;FAHD2B;FAM105A;FAM107B;FAM114A2;FAM118A;FAM126B;FAM135A;FAM136A;FAM168B;FAM177A1;FAM193A;FAM198B;FAM208B;FAM20B;FAM212B;FAM3A;FAM49B;FAM98A;FANCC;FARP1;FASLG;FAXC;FBLN5;FBXL6;FBXO11;FBXO24;FBXO28;FBXO3;FBXO5;FCGR1B;FCN2;FDFT1;FDXR;FES;FGF23;FGF9;FGFR3;FGGY;FGL2;FH;FHL5;FICD;FIG4;FKBP14;FKBP1B;FKBP2;FKBP9;FKBP9P1;FKRP;FKTN;FMO3;FN1;FNDC3B;FOLH1;FOLR2;FOXD2;FOXE1;FOXF2;FOXG1;FOXM1;FPR1;FRAT2;FRS3;FSTL1;FTH1;FUT8;FZD5;G3BP2;G6PD;GABARAP;GABBR1;GABRA3;GABRB3;GABRE;GABRG3;GADD45A;GADD45GIP1;GAL3ST1;GALK1;GALNT11;GALNT3;GALNT7;GALR2;GALR3;GALT;GAS2;GATA2;GATA4;GBP2;GCH1;GCNT2;GDAP1;GDE1;GDF10;GDF11;GDI1;GEM;GFOD1;GFOD2;GGA3;GGH;GHSR;GID4;GINS1;GINS4;GJA5;GKN1;GLI2;GLRX;GLRX2;GLTP;GMNN;GNA13;GNB3;GNB5;GNL3;GNRHR;GP1BB;GPALPP1;GPATCH2;GPBP1L1;GPC4;GPNMB;GPR1;GPR107;GPR135;GPR17;GPR183;GPR22;GPR27;GPR39;GPR63;GPRC5A;GPX4;GRB14;GRB7;GREB1;GREB1L;GREM2;GRHL2;GRIK2;GRK2;GRM8;GRTP1;GRWD1;GSK3B;GSPT2;GSR;GSTA1;GSTP1;GSTT2;GSTT2B;GTF2A1;GTF2A2;GTF2IRD2;GTF2IRD2B;GTPBP4;GUF1;GUSB;GYS1;HADH;HAGH;HAS2;HAUS8;HDAC5;HECA;HECTD3;HERPUD1;HEXA;HEXB;HGD;HGF;HHLA3;HIPK1;HIST1H1D;HIST1H2BC;HIST1H2BI;HIST2H2BF;HIST2H4A;HIST2H4B;HIVEP3;HLA-DQB2;HMGB1;HMGN5;HMOX1;HNRNPA0;HNRNPM;HOXA9;HOXB1;HOXB7;HOXC4;HPS4;HPSE;HSD11B1;HSD17B11;HSD17B14;HSD17B2;HSD3B1;HSF1;HSF2;HSF4;HSPA12A;HSPA13;HSPA1L;HSPA4;HSPA8;HSPB7;HSPD1;HTRA1;HTT;IAPP;IBSP;ICAM5;ICE1;ICMT;ID1;IDH2;IDI1;IER5;IFI16;IFI44;IFIH1;IFITM3;IFNG;IFNGR2;IFRD1;IFRD2;IGF1;IGF1R;IGF2R;IGFBP4;IGFLR1;IGHMBP2;IGSF3;IGSF6;IKZF1;IL12A;IL13RA2;IL17RB;IL17RC;IL1R2;IL1RL1;IL20RA;IL22RA1;IL3;IL36RN;IL7R;IMP3;IMPG1;ING2;INHA;INPP5A;INPP5B;INPP5D;INPP5J;INPP5K;INSL3;INSL4;INSR;INTS7;IPO13;

			IRF4;IRGC;ISOC2;ITFG1;ITGA2;ITGA3;ITGAX;ITGB2;ITM2A;IVNS1ABP;JAG1;JAM2;JARID2;JCAD;JOSD1;JUN;KANK2;KANSL3;KAT2B;KCNA5;KCND1;KCNF1;KCNH4;KCNN2;KCNQ2;KCTD14;KCTD9;KDELC1;KDELR1;KDM4A;KDM5D;KDM7A;KEAP1;KHDC1;KHK;KIAA0232;KIAA0368;KIAA0408;KIAA1024;KIAA1107;KIAA1324;KIAA1644;KIF1B;KIF1C;KIF21B;KIF3A;KIF4A;KIFC3;KIR3DX1;KLHDC10;KLHL15;KLHL21;KLHL24;KLHL42;KLRC1;KLRC2;KLRC4;KMT2C;KMT5A;KPNA2;KPNA6;KREMEN2;KRT18;KRT222;KRT32;KRT4;KRT8;KRTAP5-3;KRTAP5-7;L1CAM;LACTB2;LAMA3;LAMP3;LARP1;LBH;LCAT;LDB2;LETM1;LGALSL;LGMN;LHX6;LIAS;LIF;LIG1;LIG4;LILRA4;LIN28A;LINC00483;LMAN2L;LMCD1;LMNB1;LNX1;LONP1;LOXL2;LPCAT1;LRFN3;LRP1B;LRP4;LRPAP1;LRRC17;LRRC2;LRRC20;LRRC27;LRRC40;LRRC8D;LRRC8E;LRRTM4;LSAMP;LSM1;LSM3;LTA4H;LUC7L;LUC7L2;LY6D;LYZ;MAD2L1;MADD;MAFG;MAGEA11;MAGEA9;MAGEA9B;MAGEC3;MAGIX;MAGT1;MAN2A1;MANF;MANSC1;MAP1B;MAP1LC3C;MAP2K4;MAP3K12;MAP4K3;MAPK6;MAPKAP1;MARC2;MARCH1;MARCH3;MAST4;MAT2A;MAT2B;MATN3;MATR3;MB;MBIP;MCC;MCL1;MCM3;MCM8;MCMBP;MCTP2;MCUB;MCUR1;MDH1;MDM1;MDM2;MEAF6;MED22;MED23;MED31;MED9;MEF2C;MEF2D;MEGF8;MEP1A;MESD;METAP2;METRN;METTL16;METTL2B;METTL9;MFHAS1;MFSD12;MFSD9;MGP;MGST3;MIA3;MICA;MICU2;MIEN1;MINDY1;MIR22HG;MKNK1;MLNR;MLXIP;MMD;MME;MMP10;MMP8;MNAT1;MNDA;MORC4;MPC1;MPC2;MPDU1;MPHOSPH8;MPI;MPV17;MREG;MRM1;MRPL18;MRPL22;MRPL58;MRPS15;MRPS16;MRPS18B;MRPS2;MRPS28;MRPS34;MSMO1;MT-CO2;MT1HL1;MT1M;MT1P3;MTA3;MTCH2;MTDH;MTERF1;MTERF4;MTHFS;MTMR12;MTX1;MUC7;MVD;MXI1;MYBBP1A;MYBPC1;MYEF2;MYOG;MYOZ3;MYT1L;N6AMT1;NAA40;NAA50;NABP1;NACC2;NAE1;NAGK;NAMPT;NAP1L1;NARS2;NAT1;NCAM2;NCAPG2;NCBP1;NDUFA1;NDUFA5;NDUFB1;NDUFB11;NDUFB4;NDUFB5;NDUFS7;NDUFS8;NEK9;NELFCD;NELFE;NET1;NFE2L2;NFKB1;NFKBIE;NIP7;NKX2-2;NKX2-5;NKX3-2;NLGN4X;NMD3;NME5;NMRK1;NMRK2;NOC3L;NOP10;NOP2;NOX3;NPRL2;NPTN;NPTXR;NR0B2;NR2C2;NR2E1;NR4A3;NRBP1;NRDC;NRN1;NSA2;NSF;NSL1;NSMAF;NT5DC2;NTNG1;NTSR1;NUBP2;NUCB2;NUCKS1;NUDC;NUDT19;NUFIP2;NUP153;NUPL2;NUS1;NVL;NWD1;NXPH4;NYX;OAZ1;OAZ2;OCIAD2;ODAM;ODF2;OFD1;OR10H1;OR10H2;OR10H5;OR1A2;OR2F2;OR2J3;OR2S2;OR2W1;ORC6;OSTF1;OSTM1;OTC;OTUD1;OTUD4;OXA1L;OXCT1;OXSR1;P2RX2;P2RY6;PACRG;PAF1;PAK1;PAK6;PANK3;PAPPA;PAQR4;PAQR6;PARK7;PARP11;PARP4;PARP6;PBLD;PBX3;PBXIP1;PCBP3;PCCA;PCCB;PCDHA11;PCDHA4;PCDHA6;PCDHB8;PCK1;PCLAF;PCNA;PCNT;PCOLCE2;PCOTH;PCSK1;PCYOX1;PDCD10;PDCL3;PDE11A;PDE12;PDE4A;PDE4B;PDE4C;PDE4DIP;PDGFB;PDGFRA;PDGFRL;PDHA2;PDHX;PDIA5;PDK1;PDK4;PDLIM1;PDSS2;PER3;PEX11A;PEX5L;PFDN5;PFKFB3;PFKP;PFN2;PHAX;PHF24;PHGDH;PHKA2;PHLDA1;PHLDA2;PHLPP2;PIAS2;PIDD1;PIGA;PIK3CG;PIK3R4;PIM1;PIM2;PIP5K1C;PITPNC1;PKD2;PKIA;PKN1;PLAC8;PLAG1;PLCB2;PLCB4;PLCH2;PLEKHA1;PLGRKT;PLOD2;PLPP1;PLS1;PLXNA2;PLXNA4;PLXNC1;PMAIP1;PMEPA1;PMF1;PMP22;PMPCA;PNKP;PNMT;PNOC;PNPLA3;PNRC1;PODNL1;PODXL;POFUT2;POLA1;POLD4;POLE3;POLG;POLR2E;POLR2F;POLR2G;POLR2H;POLR3G;POM121L1P;POMP;POP4;POTEF;POTEG;POTEJ;POU2F1;POU2F3;POU4F1;POU4F3;PPAT;PPIC;PPM1D;PPP1CC;PPP1R14B;PPP1R15B;PPP2R2A;PPP2R5D;PPP3R1;PPP4R3A;PPT1;PPTC7;PQBP1;PRDM13;PRICKLE3;PRKAA1;PRKCD;PRKN;PRKX;PRPS2;PRR14;PRR4;PRR5L;PRR7;PRRG3;PRSS16;PRY;PRY2;PRYP3;PRYP4;PSAT1;PSMB7;PSMB8;PSMC1;PSMC4;PSMC6;PSMD5;PSMD7;PSME1;PSTPIP2;PTCD3;PTEN;PTGS2;PTP4A1;PTPN1;PTPRJ;PTPRO;PTRH2;PUS7;QARS;QDPR;QPRT;RAB11FIP1;RAB23;RAB27A;RAB30;RAB32;RAB36;RAB3A;RAB3GAP1;RAB40B;RAB8A;RABGAP1L;RABGGTA;RABL3;RACGAP1;RAD51C;RAD51D;RAD54L;RAD54L2;RAE1;RAI14;RAMP2-AS1;RAP1B;RAP2C;RAPGEF4;RARS2;RASAL2;RASGRF1;RASGRP3;RASIP1;RASSF9;RB1;RBM12B;RBM19;RBM3;RBM41;RBM48;RBMS2;RBMY1F;RBMY1HP;RCBTB1;RCBTB2;RCN2;RCOR1;RECQL;REEP3;REEP4;RELB;REPIN1;RFX7;RGCC;RGS17;RGS20;RGS3;RHCG;RHOQ;RIPK4;RMI1;RNASE1;RNASE6;RNASEH1;RNASET2;RNF11;RNF111;RNF125;RNF144A;RNF187;RNF2;RNF216;RNF24;RNF38;RNF6;ROGDI;ROM1;RORB;RORC;ROS1;RPA3;RPGRIP1;RPL28;RPL37;RPL39;RPL6;RPLP1;RPN1;RPRD2;RPS6KA6;RPS6KB2;RPS6KC1;RRAGC;RRAS2;RRP15;RRP36;RRS1;RSAD2;RSRC2;RSRP1;RTCB;RTF1;RTL10;RTL8A;RUVBL1;RXFP3;RXRB;RYR3;S100A1;S100A2;S100A7;S100A7A;S100P;SACM1L;SACS;SAFB;SALL1;SAMD14;SASS6;SCAND2P;SCARA3;SCGB1D1;SCGB1D2;SCN2A;SCO2;SCP2;SDC3;SDE2;SDHAF3;SDHB;SEC22A;SEC61A2;SEC61G;SECISBP2;SELENBP1;SEM1;SEPT14;SEPT7;SERP1;SERPINB1;SERPINB2;SERPINB5;SERPINH1;SERPINI2;SESN1;SETD5;SF3B2;SF3B3;SFMBT1;SFPQ;SFRP4;SFXN1;SH2B3;SH3BP1;SH3GL2;SHOC2;SI;SIDT1;SIPA1;SIRT4;SIX1;SIX5;SKIL;SKIV2L2;SLAMF1;SLBP;SLC10A3;SLC13A4;SLC16A4;SLC17A5;SLC17A6;SLC19A2;SLC1A6;SLC22A13;SLC22A8;SLC25A23;SLC25A28;SLC25A36;SLC25A44;SLC26A2;SLC26A4;SLC27A6;SLC28A1;SLC2A4RG;SLC2A6;SLC30A10;SLC31A1;SLC34A2;SLC35B3;SLC35C1;SLC35D2;SLC35F5;SLC38A10;SLC38A2;SLC38A6;SLC39A6;SLC3A2;SLC5A3;SLC6A14;SLC6A16;SLC6A9;SLC7A1;SLC7A11;SLCO1B1;SLFN12;SLFN13;SMAD1;SMAD6;SMAD7;SMARCE1;SMC4;SMG6;SMIM9;SMN1;SMN2;SMNDC1;SMPD3

			;SMPDL3A;SMS;SMYD2;SNAI1;SNAP91;SNAPC5;SND1;SNORA70;SNRNP70;SNRPC;SNRPD1;SNRPN;SNURF;SNX2;SOCS6;SOGA3;SORCS3;SOWAHC;SOX3;SOX5;SP4;SPAG1;SPATA6;SPC25;SPECC1L;SPOUT1;SPRR1A;SPRY1;SPRY2;SREK1IP1;SRF;SRGN;SRP14;SRP9;SRP9P1;SRPRA;SRPX;SRRM2;SRSF6;SSBP2;SSH1;SSH2;SSR1;SSR3;SSX2IP;SSX7;ST18;ST8SIA2;ST8SIA4;STARD5;STARD7;STAT6;STIL;STK16;STK26;STOML2;STON1-GTF2A1L;STRADB;STUB1;STX12;STX1A;STXBP3;SUCO;SUGCT;SULT1B1;SUN1;SUPT3H;SUPT5H;SUSD5;SV2B;SYNCRIP;SYNE1;SYNE2;TAB1;TAC3;TADA3;TAF13;TAF4B;TAF9;TAF9B;TAGLN;TAGLN3;TARBP2;TAS2R10;TAS2R13;TAS2R9;TASP1;TAX1BP3;TAZ;TBC1D13;TBC1D2B;TBCCD1;TCEAL1;TCEAL9;TCF12;TCN1;TCTA;TCTN2;TDRD7;TEAD3;TERF2;TESK1;TET3;TEX2;TEX30;TFB2M;TFEB;TFPT;TGFB1I1;TGFBI;TGM1;THAP10;THAP3;THOC7;THPO;TIA1;TIGAR;TIMM10;TIMM29;TIMP1;TINF2;TIPARP;TIPRL;TK1;TKFC;TLE4;TLR1;TLR4;TLX1;TLX3;TM9SF3;TMC7;TMCC1;TMCC2;TMCO3;TMED5;TMEM131L;TMEM132A;TMEM154;TMEM156;TMEM165;TMEM168;TMEM187;TMEM19;TMEM2;TMEM206;TMEM208;TMEM230;TMEM248;TMEM254;TMEM30A;TMEM30B;TMEM41B;TMEM50A;TMEM50B;TMEM62;TMEM74B;TMPRSS11E;TMPRSS6;TMSB15A;TMTC3;TMX4;TNFAIP3;TNFAIP8L1;TNFRSF11A;TNFSF10;TNFSF15;TNFSF9;TNKS;TNKS1BP1;TNP2;TNRC6A;TNRC6B;TNS2;TNS4;TOB1;TOM1L2;TOP1;TOP2B;TOR1B;TOR4A;TOX;TP53I3;TP53TG5;TPD52L2;TPM3;TPMT;TPPP;TPRKB;TRAF1;TRAF3;TRAF5;TRAFD1;TRAM1;TRAM2;TRANK1;TRAPPC13;TRAPPC3;TRAPPC6A;TRIB1;TRIB3;TRIM13;TRIM17;TRIM22;TRIM36;TRIM44;TRIM58;TRIM6;TRIM6-TRIM34;TRIM65;TRIM68;TRIOBP;TRIP6;TRMT11;TRMT2A;TRMT2B;TRPC4;TRPC7;TRPM1;TRPS1;TRPV6;TSC22D2;TSEN34;TSFM;TSGA10;TSHB;TSHZ1;TSKU;TSPAN12;TSPAN13;TSPAN3;TSTA3;TTI2;TTK;TTLL12;TUB;TUBD1;TUBGCP4;TUBGCP5;TULP2;TUSC3;TXK;TXLNA;TXNRD1;TYMS;UBB;UBE2E1;UBE2H;UBE2K;UBE2V2;UBE3C;UBL3;UBN2;UBQLN3;UBR3;UBR4;UFC1;UFSP2;UGDH;UGGT1;UGT2A1;ULK2;UNC13A;UNC93B1;UPF3B;UQCR11;UQCRC2;URB2;USB1;USP14;USP18;USP20;USP3;USP32P1;USP6;USP9X;UXT;VAMP7;VANGL1;VANGL2;VAV1;VCP;VEGFD;VIM;VIPAS39;VNN2;VPREB1;VPS13C;VPS33A;VPS54;VTN;WARS;WASF2;WASHC3;WASHC4;WDHD1;WDR25;WDR4;WDR60;WDR83OS;WDR91;WDR92;WFDC6;WHAMMP3;WISP3;WLS;WNK1;WNT5A;WRAP73;WRB;XDH;XK;XRCC2;XRCC5;YAP1;YIF1A;YIPF5;YOD1;ZBED1;ZBTB18;ZBTB20;ZBTB24;ZBTB25;ZBTB38;ZBTB45;ZC3H4;ZCCHC8;ZDHHC18;ZDHHC7;ZFPM2;ZFY;ZKSCAN1;ZKSCAN3;ZKSCAN7;ZMAT4;ZNF133;ZNF134;ZNF136;ZNF140;ZNF141;ZNF195;ZNF207;ZNF23;ZNF254;ZNF324B;ZNF347;ZNF35;ZNF410;ZNF423;ZNF426;ZNF451;ZNF468;ZNF480;ZNF548;ZNF549;ZNF551;ZNF556;ZNF566;ZNF573;ZNF584;ZNF608;ZNF652;ZNF664;ZNF669;ZNF672;ZNF680;ZNF696;ZNF772;ZNF81;ZNF839;ZNF84;ZNF85;ZNF860;ZNF862;ZNHIT1;ZNHIT2;ZSCAN16;ZSWIM6;ZWINT
hsa-let-7e-5p-478579_mir	-2.238	0.422	ABHD17C;ABT1;ACER2;ACOT9;ACTA1;ADH5;ADIPOR2;AEBP2;AGMAT;AGO1;AGO2;AHCYL2;AHR;AK4;AKAP8;ALG13;AMD1;AMPD2;ANKRD40;ANKRD46;AP1S1;APPL1;AQP6;ARCN1;AREL1;ARHGAP19;ARID1A;ARID3A;ARID3B;ARIH1;ARL8B;ARNT2;ATG12;ATG9A;ATP6V1F;ATP6V1G1;ATXN2;ATXN2L;ATXN7L3;ATXN7L3B;AURKB;BACH1;BAHCC1;BAZ1B;BEND4;BMP2K;BRI3;BRI3BP;BSDC1;BTRC;BZW1;C11ORF57;C11ORF91;C12ORF4;C12ORF49;C19ORF47;C19ORF48;C19ORF53;C1ORF21;C1ORF210;C1RL;C5ORF51;CA5B;CABLES1;CALU;CARM1;CASTOR2;CBX5;CCDC106;CCDC113;CCDC97;CCND1;CCNG1;CCNT2;CD59;CDC5L;CDCA3;CDH18;CDKAL1;CDKN1A;CDV3;CELF1;CELF2;CENPP;CEP120;CEP135;CHD7;CHD9;CHTOP;CLDN12;CLDN4;CLTC;CNBP;COIL;COL6A1;COL8A1;COLEC12;COPG1;COX10;COX14;COX16;COX6B1;CPA4;CREBBP;CRK;CRX;CRY2;CTC1;CTPS1;CTSA;CWC15;CXCL8;CYP2B6;DAAM1;DCAF6;DCAF8;DCBLD2;DGCR8;DHX15;DHX57;DIABLO;DIAPH1;DISC1;DNA2;DNAH9;DNAJC28;DNAL1;DSP;DTNB;DTX3L;DUSP1;DVL3;DYRK2;DYRK3;DZIP1;E2F6;ECHDC1;EDEM3;EDN1;EFHD2;EIF3J;EIF4A1;EIF4A3;EIF4EBP2;EIF4G2;ELMSAN1;EMILIN2;EN2;ENSA;EPHA4;ERO1A;ESPL1;EZH2;FAM104A;FAM105A;FAM160B1;FAM219B;FAM222B;FAM43A;FAM83G;FASLG;FBXL20;FBXW2;FDPS;FIGN;FMNL3;FMO4;FNDC3A;FNDC9;FOXD4L6;FPR1;FUT10;FXN;FZD9;GABPAP;GATB;GATM;GGA3;GLO1;GLUL;GMPS;GNG5;GOLGA4;GPAT4;GPM6B;GRPEL2;GTF2I;GTF3C1;GTPBP8;HAND1;HASPIN;HERPUD1;HIF1AN;HIPK1;HIST1H2BD;HIST1H2BK;HIST2H2BF;HLA-C;HMGA1;HMGA2;HMGB1;HNRNPC;HNRNPUL1;HS6ST2;ICOSLG;IFNLR1;IGDCC4;IGF1;IGF1R;IGF2BP1;IGF2BP3;IKZF3;IL6R;INTS7;IPO9;IRGQ;IRS2;IRS4;ITGA3;IVD;JAZF1;JMJD1C;KATNAL1;KATNB1;KCTD21;KIAA0100;KIAA0355;KIAA0391;KIAA0930;KIAA1143;KIAA1328;KIF27;KLHDC8B;KMT2D;KPNA5;KRBOX4;KREMEN1;LEFTY1;LHFPL2;LIMD2;LIN28A;LMLN;LMNA;LRIG3;LRRC20;LRRC41;LRRC8A;LSR;LUZP1;LYN;MACF1;MAGEA12;MAGEA3;MAGEA6;MAP2K7;MAPK6;MARCH5;MARCKSL1;MARS2;MATR3;MBD2;MCF2L2;MDM4;MED13L;MEF2D;MEIS3P1;MFSD8;MIDN;MIEF1;MKI67;MLLT10;MMP9;MPL;MRPL12;MRPS2;MS4A10;MSI2;MSMO1;MT-ATP6;MT-CO1;MT-CO3;MT-ND1;MT-ND2;MT-ND3;MT-ND4;MT-ND5;MTCH2;MTFR1;MTMR14;MTUS1;MTX3;MXD1;MYC;MYCN;MYO9B;NAA20;NAA30;NAA60;NAP1L1;NAT8L;NCBP1;NCKAP5L;NCKIPSD;NCLN;NCOA3;NDST1;N

			DUFA3;NDUFA4P1;NDUFS5;NF1;NHLRC2;NHLRC3;NICN1;NME4;NOA1;NOCT;NOLC1;NOM1;NR6A1;NRSN2;NSD1;NT5DC2;NUCB2;NUDT15;NUDT8;NUP155;OCLN;ONECUT2;OPA3;OPRL1;OR7D2;OTUB1;OTUD5;PA2G4;PACS2;PAFAH2;PAPD4;PARP16;PAX3;PBX2;PCBP2;PCGF3;PDCD11;PDE12;PDGFB;PDLIM5;PDP2;PDZD8;PEG10;PES1;PEX11B;PFAS;PGD;PGM2L1;PGRMC1;PHACTR4;PHF3;PHKA1;PIGN;PIGP;PIGS;PLAGL2;PLCG2;PLCXD1;PLD3;PLEKHA3;PLEKHO1;PLK1;PLXND1;PM20D2;PMAIP1;PMPCA;POLD1;POLL;POLR2D;POLR3D;POTEG;POTEM;PPIG;PPP1R10;PPP1R15B;PPP1R2;PPP2R1A;PPP2R2A;PRAMEF13;PRIM2;PRPF8;PRR5-ARHGAP8;PRRC2A;PRSS22;PSD3;PSMA6;PSMD2;PSME4;PTK2;PYCR1;QDPR;QSOX1;RAB11FIP4;RAB19;RAB40C;RABL2A;RABL2B;RABL6;RACGAP1;RAD18;RAI2;RANBP2;RAP1A;RBBP4;RBFOX2;RBM12B;RBM14;RBM4;RBM8A;RCC1L;RCOR3;RDX;RFC2;RHBDD2;RHBDF2;RHD;RMND5A;RNF144B;RNF26;RNF44;RNFT1;RNMT;RPA1;RPL10;RPL12;RPL27A;RPLP2;RPN2;RPRD2;RPS27;RPSA;RRAD;RRAGC;RRM1;RRM2;RSL1D1;RUNX1T1;RWDD1;SALL1;SALL3;SAR1A;SCD;SCMH1;SCYL1;SDR42E1;SEC23IP;SEMA4B;SEMA4C;SERBP1;SERF2;SETD1A;SGSM3;SHANK1;SKA2;SKIV2L;SLC10A7;SLC11A2;SLC12A4;SLC12A7;SLC16A9;SLC19A3;SLC20A1;SLC2A11;SLC38A2;SLC38A7;SLC5A6;SLCO3A1;SLCO4A1;SMAP2;SMARCAD1;SMC1A;SMCR8;SNX17;SOCS1;SOCS4;SOD2;SPAG9;SPATA13;SPCS2;SPCS3;SPN;SPTBN1;SQLE;SREBF1;SRSF2;SSB;STAM;STARD7;STAT2;STAT3;STK4;STRN;STX3;SUB1;SUGP2;SUMO1;SUOX;SUPT4H1;SURF4;SUZ12;SVIL;SYNJ2BP;SYT1;TBC1D19;TBC1D31;TCF4;TDRD7;TERF1;TGFBR3;TGOLN2;THADA;THBS1;THEM6;THYN1;TIAF1;TIMM50;TIMP3;TMED4;TMED5;TMEM107;TMTC3;TNFAIP1;TNFAIP3;TNFRSF10B;TNFRSF1A;TNFSF9;TOMM40L;TRAPPC10;TRIM71;TRMO;TRPV1;TRRAP;TSC22D2;TTLL12;TUBA1B;TUBB2A;TUBB4A;TUBB4B;TXLNA;TXLNG;TXNRD1;UBAP2L;UBE2H;UBE2V2;UBN2;UBXN2B;UGGT1;UHRF1BP1;UROC1;USE1;USP37;USP38;USP47;UTP15;VAMP2;VARS;VCL;VGLL4;VPS13D;WASL;WDFY3;WDR4;WDR48;WNT1;XIAP;YAE1D1;YOD1;YWHAE;YWHAG;YWHAQ;YWHAZ;ZBTB37;ZBTB5;ZBTB8OS;ZC3HAV1L;ZCCHC3;ZFAND4;ZFAND5;ZFP3;ZFP62;ZKSCAN7;ZNF200;ZNF236;ZNF256;ZNF264;ZNF28;ZNF284;ZNF417;ZNF443;ZNF451;ZNF460;ZNF556;ZNF566;ZNF578;ZNF584;ZNF587;ZNF609;ZNF611;ZNF644;ZNF652;ZNF738;ZNF770;ZNF774;ZNF799;ZNF8;ZNF805
hsa-miR-19a-3p-479228_mir	-1.672	0.424	ABCA1;ABHD14B;ABHD17C;ABHD5;ACBD5;ACSL4;ACTB;ADIPOR2;ADRB1;ADRM1;ADSS;AFF1;AFTPH;AGO1;AGO3;AGPAT5;AHDC1;AKAP2;AKT1;ALG1;ALG2;ALOX5;AMMECR1L;ANGEL2;ANKIB1;ANKRD12;ANKRD50;AP3S2;ARAP2;ARC;ARFGEF1;ARHGAP1;ARHGAP12;ARHGEF26;ARIH2;ARL8A;ARMC8;ARPIN;ARPP19;ARRDC3;ASNA1;ATG14;ATG16L1;ATG2B;ATG5;ATMIN;ATP6V0E1;ATP6V1B2;ATPAF1;ATXN1;ATXN7;AZIN1;B3GALNT2;B4GALT1;BAMBI;BCL2L11;BCL3;BCL7A;BCL7B;BEND3;BLCAP;BMP3;BMPR2;BRD9;BRWD1;BRWD3;BTBD7;BTF3L4;BTG1;C11ORF57;C15ORF38-AP3S2;C16ORF70;C2ORF42;C5ORF24;C5ORF30;C5ORF51;C6ORF132;CAB39;CALM1;CAMSAP1;CAMSAP2;CAMTA1;CAPRIN2;CASZ1;CBX5;CBX7;CBY1;CC2D1A;CCDC137;CCDC80;CCNA2;CCND1;CCND2;CCNL1;CCNT2;CCSER2;CD164;CD22;CEP170;CEP350;CEP55;CERCAM;CFL2;CHD9;CHEK1;CHEK2;CHERP;CIT;CLIC4;CLIP1;CLOCK;CLVS2;CNOT4;CNOT6;CNOT7;COQ10B;COX10;CREB3L2;CREBL2;CREBRF;CSNK2A1;CUL5;CYP2U1;DAD1;DBN1;DCAF7;DCBLD2;DCC;DCP2;DCUN1D3;DDX3X;DDX3Y;DDX6;DEF8;DENND6A;DEPDC1;DGKH;DHX40;DICER1;DIP2A;DLG5;DMXL2;DNMT1;DPYSL2;DSCR3;DSEL;DSN1;DUT;DYNC1LI2;E2F8;EFR3A;EHD1;EIF4A2;EIF4E2;ELL2;ELMOD2;ELOVL5;ENPP4;ENPP5;EOGT;EPN2;EPS15;ERBB4;ERCC4;EREG;ESR1;ESYT1;ETV3;EVI5L;EXOC7;FAM102A;FAM46C;FAM83D;FAS;FBLIM1;FBXO10;FBXO28;FBXO48;FBXO8;FGFR1OP;FKBP15;FN3KRP;FNDC3A;FOXP1;FOXQ1;FRMD6;FRS2;FXR1;FZD6;G3BP2;G6PC;GAK;GATAD2B;GDNF;GFPT1;GIGYF1;GINS1;GIT2;GMFB;GNPTAB;GPAM;GPR137B;GRB10;GRK6;GRSF1;GSKIP;HADHB;HBP1;HDAC4;HECW2;HIC1;HIPK3;HIST2H4A;HIST2H4B;HNRNPF;HNRNPU;HNRNPUL1;HOMER1;HOXA5;HPRT1;IDNK;IFITM1;IKZF1;IL10;IMPDH1;IMPDH1P11;INO80;ITGA2;ITPR1;JARID2;JAZF1;KAT2B;KATNAL1;KCNJ2;KCTD10;KDELR2;KIAA0907;KIAA1468;KIF13A;KIF3A;KIT;KITLG;KLF13;KLHDC2;KLHL11;KLHL20;KLHL3;KLHL42;KPNA6;LCLAT1;LDLR;LIN9;LMLN;LONRF1;LZIC;MACF1;MALT1;MAP2K3;MAP3K1;MAP3K14;MAP3K5;MAP3K9;MAPK1;MAPK14;MAVS;MB21D1;MB21D2;MBD3;MBD4;MBNL1;MBNL2;MBNL3;MCC;MCM3AP-AS1;MCRIP2;MECP2;MED12L;MEF2A;MEF2D;MFF;MFSD6;MID1IP1;MIER1;MIGA1;MIGA2;MKL2;MLEC;MLLT10;MMGT1;MOB1B;MPRIP;MRPL17;MSMO1;MTF2;MTHFD1;MTMR12;MTMR6;MTUS1;MTX3;MXD1;MYC;MYCN;NACC1;NACC2;NAPB;NDRG1;NF1;NFATC2IP;NFIA;NFIB;NICN1;NIPA1;NPEPL1;NPTN;NR4A2;NRBF2;NRBP1;NUFIP2;NUP54;NUS1;OCRL;ODF4;OTUD1;OTUD4;OTUD7B;PABPC4L;PAFAH1B2;PAICS;PALM2-AKAP2;PAPD4;PATL1;PCDH10;PDE4A;PDRG1;PFN1;PFN2;PGK1;PGM2L1;PHF13;PHLDA1;PHLDA3;PHLPP1;PIGS;PIK3CA;PIK3R3;PKM;PKNOX1;PLAU;PLEKHF2;PLXNC1;PMEPA1;PNRC1;POGZ;POLI;PPARA;PPP1R15B;PPP2R5E;PPP6R1;PPTC7;PRICKLE2;PRKAA1;PRKACB;PRKN;PRMT5;PRR14L;PRRC2C;PRRG4;PRUNE2;PSAP;PTBP2;PTCD2;PTEN;PTPRB;PTPRG;PURG;QKI;RAB13;RAB14;RAB18;RAB1A;RAB21;RAB2

			B;RAB34;RAB5B;RAB8B;RACGAP1;RAF1;RAN;RAP1A;RAP1B;RAP2C;RAPGEF2;RAPGEF4;RAPGEF6;RASA1;RASSF2;RASSF5;RBBP8;RBM20;RBM25;RBM38;RCOR1;REEP3;RGL1;RHOB;RLIM;RNF11;RNF111;RNF167;RNF216;RNF4;RNF44;RORA;RPAP2;RPF2;RPS4Y1;RPS6KA5;RRAGD;RRAS2;S1PR2;SAMD8;SATB1;SBF2;SCD;SDE2;SEC61A1;SEC63;SECISBP2L;SEL1L;SEMA4C;SEPHS2;SERBP1;SERINC3;SESN3;SF3B3;SFTPA1;SGK1;SH3KBP1;SHCBP1;SIVA1;SKIL;SLC12A7;SLC25A12;SLC27A1;SLC30A7;SLC37A1;SLC38A2;SLC46A1;SLC48A1;SLC6A8;SLC7A11;SLC9A1;SLC9A6;SMAD4;SMAD5;SMARCA2;SMG1;SMOC1;SNX17;SNX5;SOCS1;SOCS3;SOX4;SOX6;SPART;SPATA2;SPTSSA;STAT5B;STK38;STK4;STOX2;STX12;STX16;STX6;SUZ12;TAF4;TBRG1;TF;TFB1M;TGFBR2;TGIF1;TGOLN2;THBS1;TLR2;TLR7;TMBIM6;TMEM106B;TMEM117;TMEM138;TMEM2;TMEM64;TMEM87A;TMEM9B;TMOD3;TMTC1;TNF;TNFAIP3;TNFRSF10B;TNFRSF11A;TNFRSF12A;TNFRSF1B;TNIP1;TNKS;TNPO2;TNRC6A;TNRC6B;TP53;TP53INP1;TPRG1L;TRAK2;TRIM33;TRIM37;TRPC3;TUSC2;TXLNG;UBE2A;UBE2D3;UBL3;UBN2;USP13;USP37;USP8;VAMP1;VAMP3;VPS37A;VPS37B;VPS4B;WAC;WASL;WBP2;WBP4;WDFY3;WDR1;WDR20;WDR26;WDR45B;WNK1;WNK3;WNT10A;WNT7B;XIAP;YTHDF1;YY1;ZBTB18;ZBTB4;ZBTB47;ZBTB7B;ZDHHC18;ZDHHC7;ZFAND5;ZFYVE26;ZFYVE9;ZIC5;ZMAT3;ZMYND11;ZNF107;ZNF134;ZNF138;ZNF154;ZNF217;ZNF367;ZNF423;ZNF526;ZNF544;ZNF618;ZNF644;ZNF680;ZNF721;ZNF772;ZNF800
hsa-miR-25-3p-477994_mir	1.101	0.427	AAED1;ABCF2;ACOD1;ACP1;ACSS1;ACTC1;ACTL6A;ADAMTSL1;AEN;AGBL5;AGMAT;AGO1;AKAP10;ALG14;AMD1;ANKIB1;ANKRD18A;ANKRD24;ANP32E;ANXA11;AP3M1;AP3S2;AP5Z1;APOBEC3F;ARF1;ARGFX;ARHGAP17;ARID1B;ARNTL2;ARPC3;ARPC5L;ASGR2;ASS1;ATF7IP;ATN1;ATOX1;ATP2A2;AURKA;B4GALT7;BAK1;BAZ2B;BCAT1;BCL11B;BCL2L11;BMP8A;BMPR1A;BPTF;BTBD7;BTG2;BZW1;C11ORF24;C15ORF38-AP3S2;C17ORF75;C1GALT1C1;C1ORF35;C2CD5;C5ORF24;CABIN1;CAPZB;CARD6;CASKIN1;CCDC113;CCDC171;CCDC186;CCDC96;CCL26;CCNB1;CCNE2;CCSER2;CD180;CD226;CDC5L;CDH1;CDK4;CDK5R1;CDKN1C;CEP170B;CEP85;CHCHD10;CHRAC1;CHST1;CIC;CIDEC;CLIC1;CLN8;CLSPN;CLTA;CNEP1R1;CNIH1;CNNM4;CNOT11;CNOT2;CNOT4;COL1A2;COPS3;COX20;CPEB1;CPEB3;CPEB4;CRYBG1;CTC1;CTDSPL;CTNNBL1;CTSE;CYP20A1;CYP2B6;CYP2C19;CYTH2;DAND5;DCTN1;DDI2;DDIT4;DDX23;DDX3X;DDX49;DEAF1;DENND2C;DENND4A;DENND5A;DHFR;DHX9;DKK4;DNAJB9;DNM1L;DPH7;DSC2;DUS2;DUSP5;DYNC1LI2;EDEM1;EEF1A1;EID2B;EIF1;EIF4B;EIF4EBP1;EIF4EBP2;EIF4G2;EIF5A;EIF5A2;ELF4;ELOA;EMD;EME2;ENTHD1;EP300;ERBB2;ERGIC2;ESRP1;ETFA;EXOC5;EXOC6B;EZH2;EZR;F11R;FAM129A;FAM49A;FASLG;FBXW2;FBXW7;FECH;FGF2;FKBP1A;FKBP9;FLCN;FMN1;FNBP1;FNDC3B;FNIP1;FOPNL;FOXN2;FUT11;G2E3;GALNT7;GAPDH;GATA6;GATAD2B;GCNT3;GEMIN2;GFPT2;GGCX;GID4;GM2A;GNAQ;GOLGA3;GOLGA8A;GOLGA8B;GOLGA8IP;GOLGA8J;GPBP1;GPBP1L1;GPR157;GRAMD4;GTF2A1;GTF2E1;GUF1;GULP1;H3F3B;H3F3C;HAND2;HIST2H2AA3;HIST2H2BF;HIVEP1;HMBS;HMGA2;HNRNPA1;HOXA13;HOXC8;HP1BP3;HSP90AA1;HSP90B2P;HSPA1B;HSPA8;HYAL1;IFITM1;IL6ST;INCENP;INSIG1;IPP;IRGQ;ITGB1;ITGB8;ITM2B;ITPR1;KAT2B;KATNB1;KCNC4;KCTD15;KIAA1109;KIAA1586;KIAA1958;KIF23;KIF5B;KLF4;KLHDC10;KLHL15;LATS2;LAX1;LCOR;LETM1;LHFPL2;LILRA2;LONRF3;LOXHD1;MAP1B;MAP2K4;MARCH6;MBD1;MCF2L2;MCOLN2;MDM2;MED19;MED29;MED7;MFF;MKNK2;MLEC;MOAP1;MPHOSPH6;MRO;MRPS16;MRPS21;MTMR10;MUC21;MYC;MYLIP;MYZAP;NARF;NAT1;NCAPG2;NDE1;NDUFAF3;NF2;NFYB;NHP2;NKAP;NLRP9;NOL12;NOTCH2;NPM1;NPY4R;NRAS;NRN1;NSMAF;NSMCE3;NUCKS1;NUFIP2;NUGGC;NUP43;NVL;OPA3;OR2A4;ORAI2;OSMR;OTUD7B;PAIP1;PAPD7;PARD6B;PARL;PAWR;PAX9;PCBD2;PCDH7;PCMTD1;PDGFRL;PDK2;PDXP;PDZD8;PELO;PELP1;PER2;PEX5;PGAM4;PGPEP1;PGRMC1;PHB2;PHIP;PHKA1;PHLPP2;PHTF1;PIK3CD;PIP5K1C;PKDCC;PKNOX1;PLEKHA1;PLEKHA5;PLXNA3;PMEPA1;PNO1;POLK;PPIC;PPP1R37;PPP1R3D;PRKAR1A;PRMT5;PRPF8;PRPS1;PRRC2A;PRRC2B;PRRG4;PSMB4;PTAR1;PTEN;PTGES2;PURG;RAB3D;RAD51;RANBP6;RAPGEF1;RBFOX2;RBM27;RBM28;RBMS2;RBPJ;RCOR1;RECK;REL;REV3L;RGS3;RLIM;RNASEH2A;RNF4;RNF44;RNGTT;ROBO1;RPL23;RPL24;RPL26;RPL7A;RPL9;RPS14;RPS28;RPS3A;RPSA;RRN3;RSBN1;RYR2;SASH1;SBNO2;SCAF4;SEMA4C;SEMA4D;SERTAD3;SESN3;SETD1B;SH2B3;SHE;SLC12A4;SLC12A5;SLC25A32;SLC25A36;SLC33A1;SLC39A14;SLC4A7;SLC7A11;SLX4;SMAD7;SMAP1;SMAP2;SMARCA5;SMU1;SNPH;SNRPD1;SNTB2;SOX11;SOX4;SPATS2L;SPCS3;SPRYD4;SREBF1;SRFBP1;SRPRA;SRRM2;SSFA2;SSRP1;STAT2;STXBP1;SUPT16H;SUPT7L;SYNPR;SZRD1;TAF8;TANK;TATDN3;TBC1D8;TCEAL1;TEF;TIRAP;TLN1;TLR3;TMEM173;TMEM184B;TMEM239;TMEM33;TMEM41A;TMEM44;TMF1;TMUB1;TNFRSF13C;TNFSF10;TNPO2;TOB1;TOR1B;TOR4A;TP53;TPPP;TRAF3IP1;TRAM2;TRAPPC2;TRIM36;TRMT2B;TSFM;TSHZ3;TSPAN31;TUBB;TUBB4B;TUBGCP4;TULP4;TUSC3;TVP23B;TWF1;TXLNA;TXNDC15;UBAP2L;UBE2Z;UBR1;UBXN4;UCK2;UGDH;UHRF1BP1;UQCRFS1;USP28;VHLL;VMA21;VMP1;VPS4B;WASL;WDR4;WDR81;XKR7;XPC;YBX1;YIPF4;ZADH2;ZBTB34;ZBTB8B;ZBTB8OS;ZC3HAV1L;ZDHHC21;ZDHHC24;ZDHHC5;ZDHHC8;ZFC3H1;ZFP62;ZFYVE21;ZIC5;ZNF134;ZNF157;ZNF24;ZNF264;ZNF267;ZNF277;ZNF317;ZNF346;ZNF354B;ZNF383;ZNF417

			;ZNF460;ZNF557;ZNF598;ZNF607;ZNF682;ZNF695;ZNF75A;ZNF772;ZNF850;ZNRF3;ZSCAN12
hsa-miR-125b-5p-477885_mir	-1.709	0.427	ABCC1;ABCC4;ABL1;ABTB1;ACLY;ACSS1;ADM;ADRM1;AHRR;AKAP2;AKT1;ALOX5;ANAPC16;ANGPT2;ANKRD33B;APC;APLN;ARF3;ARID3A;ARID3B;ARIH2;ASB16;ATP5B;ATXN1;AURKB;B4GALT3;BACE2;BACH1;BAK1;BBC3;BCKDK;BCL2;BCL2L13;BCL2L2;BCL3;BCOR;BMF;BMPR1B;BTBD3;BTG2;C15ORF39;C17ORF80;C19ORF54;C1D;C1ORF109;C2ORF15;C2ORF88;CARHSP1;CASC3;CBFB;CCDC124;CCNE1;CCNJ;CD244;CD320;CD44;CDH5;CDKN2A;CDKN2D;CDRT4;CEBPA;CEBPG;CENPP;CEP170;CGN;CHMP3;CHPF2;CLDN12;CMTM4;CNGB1;COPZ1;COX7C;CREBBP;CRTC1;CSNK2A1;CSRNP2;CTDSP1;CYP24A1;DDX50;DGAT1;DHX33;DHX38;DHX57;DKK3;DRAM2;DUSP6;DYNC2H1;DYRK2;E2F2;E2F3;E2F7;EDEM1;EEF1A1;EGFR;EHD1;EHMT1;EIF1AD;EIF4E;EIF4EBP1;EIF5;EIF5A2;EMC1;EMD;ENPEP;EPHA7;EPO;EPOR;ERBB2;ERBB3;ESRP2;ETF1;ETS1;EXOC7;EXTL3;FAM174B;FAM199X;FAM208A;FAM91A1;FAS;FAT1;FBN1;FBXL18;FBXL20;FBXO38;FES;FGFR2;FMNL3;FRAT2;FUS;FXR1;FZD4;FZD6;GAB2;GABRB3;GALNT18;GANAB;GJB7;GLI1;GOLGA1;GOLGA8A;GOLGA8B;GPAT4;GPRIN1;GRIN2A;GRINA;GSS;HBS1L;HDGF;HID1;HIST2H2BF;HK2;HKR1;HMGA1;HMGA2;HMGCLL1;HMGN1;HNRNPK;HOTTIP;HOXA13;HOXD1;HRH4;HSPA1B;HSPBP1;HSPD1;HUWE1;ICAM2;IFRD2;IGF1R;IGF2;IKZF2;IKZF3;IKZF4;IL6R;ILVBL;IP6K1;IPPK;IRF4;IRS4;JAK2;KCNS3;KHSRP;KIAA0141;KIF24;KLC2;KLF13;KMT2D;KPNB1;KRT7;KRTAP5-9;LACTB;LBX2;LIF;LIFR;LIN28A;LIN28B;LINS1;LIPA;LONRF2;LSM14B;LSM4;LSS;LTA4H;LTV1;LUC7L3;LYPLA2;MAN1B1;MAP3K11;MAP3K2;MAPK14;MATR3;MBD4;MCL1;MEGF9;MEPCE;MFHAS1;MFSD9;MKNK2;MMAB;MMP13;MMP2;MMP26;MON1B;MRPS12;MRTO4;MTF1;MTMR12;MTMR3;MTMR4;MTRF1L;MUC1;MXD1;MYCBP2;MYEF2;MYO19;NBPF11;NCOR2;NDC1;NDOR1;NEBL;NEGR1;NES;NEU1;NF2;NKIRAS2;NME2;NNT;NPM1;NPTN;NRARP;NRDC;NSUN2;NTRK3;NUP205;NUP37;NUP93;OPRL1;PABPC1;PABPC4;PANK1;PARP1;PCBP2;PCDHGB2;PCTP;PDS5A;PDZD11;PFKM;PHF8;PHIP;PIAS3;PIGF;PIK3CB;PIK3CD;PIP4K2C;PKM;PLA2G4F;PLEKHA8;PLEKHG5;PLXDC1;PLXND1;PMAIP1;PODXL;PPAT;PPM1H;PPP1CA;PPP1R37;PPP2CA;PRDM1;PRDX2;PRKAG1;PRKRA;PSAT1;PSMB1;PTBP1;PTGES;PTGS1;PTH1R;PTOV1;QSOX2;R3HDM2;RAB4A;RAF1;RANBP2;RASAL2;RASGRP1;REST;RFWD3;RGS19;RHOV;RIMS4;RMND5A;RNASEH1P1;RNASEH2A;RNF144A;RPL29;RPL3;RPL35A;RPLP0;RPS28;RPS3A;RPS6KA1;RPS7;RRP7A;RSG1;RYBP;S100A8;SAMD10;SCARB2;SCNN1A;SDHB;SEC14L3;SEC24A;SEL1L;SEMA4B;SEMA4C;SET;SFRP5;SGPL1;SH3BP5L;SHMT1;SIK2;SINHCAF;SIRT7;SIX1;SLC16A4;SLC19A1;SLC25A1;SLC35A4;SLC35G1;SLC7A1;SLC7A6;SLC9A3R2;SMAD4;SMARCD2;SMC2;SMC6;SMIM8;SMO;SMYD5;SNRPB;SORT1;SPATA5;SPEN;SPHAR;SPHK1;SPRTN;SRRM2;SSR3;STARD13;STARD8;STAT3;STC2;STOX2;STRN3;SUGP2;SUPT6H;SUV39H1;TACC2;TAF15;TARDBP;TBC1D1;TDG;TEF;TET2;THAP3;THRAP3;THUMPD1;THUMPD3;TIMM10;TIMM23;TMEM101;TMEM136;TMEM2;TMEM59;TMEM63C;TMEM9;TNF;TNFAIP3;TNFRSF10B;TNKS1BP1;TNPO3;TNRC6B;TOR2A;TP53;TP53INP1;TRIM71;UBA6;UBAP2L;UBR7;ULK3;UNG;UNKL;USP8;VCL;VDAC1;VDR;VPS4B;VPS51;WNK1;XIAP;YOD1;ZDHHC5;ZFYVE1;ZMYND11;ZNF177;ZNF212;ZNF385A;ZNF395;ZNF483;ZNF592;ZSWIM6
hsa-let-7i-5p-478375_mir	-1.611	0.442	ABHD17C;ABT1;ACER2;ACOT9;ACTA1;ADGRL2;ADH5;ADIPOR2;AGO1;AHCYL2;AHR;AK4;AKAP8;AMD1;ANKRD46;AP1S1;AQP6;AREL1;ARID3A;ARID3B;ARIH1;ARL8B;ARPC1A;ASCL1;ATG12;ATG9A;ATP6V1F;ATP6V1G1;ATXN2;ATXN7L3;ATXN7L3B;AURKB;BACH1;BEND4;BMP4;BRI3BP;BZW1;C11ORF57;C12ORF4;C19ORF47;C19ORF53;C1ORF21;C1ORF210;C1RL;C5ORF51;CALU;CASTOR2;CBX5;CCND1;CCNT2;CD59;CDC25A;CDKAL1;CDKN1A;CDV3;CELF1;CEP120;CEP135;CHTOP;CLDN12;COIL;COL8A1;COLEC12;COPS6;COPS8;COX6B1;CPA4;CRX;CRY2;CTPS1;CXCL8;DDOST;DIABLO;DISC1;DNA2;DNAH9;DNAJC28;DNAL1;DTX3L;DUSP1;DVL3;DYRK3;E2F6;ECHDC1;EDEM3;EDN1;EFHD2;EGLN3;EIF4A3;EIF4G2;EMILIN2;EP400;EPHA4;ERO1A;ESPL1;FAM104A;FAM105A;FAM222B;FAM43A;FAM83G;FBXL20;FBXW2;FIGN;FMNL3;FMO4;FNDC3A;FNDC9;FPR1;FUT10;FXN;FZD9;GABPAP;GATM;GGA3;GLO1;GNG5;GOLGA4;GPAT4;GPS1;GRPEL2;HAND1;HASPIN;HERPUD1;HIST1H2BD;HIST1H2BK;HMGA1;HMGA2;ICOSLG;IFNLR1;IGDCC4;IGF1;IGF1R;IGF2BP1;IGF2BP3;IKZF3;IL13;IL2;IL6R;INTS7;IPO9;ITGA3;KCTD21;KDM5B;KIAA0391;KIAA0930;KIAA1143;KIAA1328;KIF27;KLHDC8B;KMT2D;KPNA5;KREMEN1;LEFTY1;LIMD2;LRIG3;LRRC20;LYN;MAGEA12;MAGEA3;MAGEA6;MAP2K7;MAPK6;MARCKSL1;MARS2;MBD2;MCF2L2;MDM4;MEF2D;MEIS3P1;MFSD8;MIDN;MIEF1;MLLT10;MRPL12;MSI2;MTUS1;MTX3;MXD1;MYC;NAA20;NAA30;NAP1L1;NAT8L;NCKIPSD;NCOA3;NDUFA4P1;NEUROG1;NHLRC2;NHLRC3;NOA1;NOM1;NR6A1;NSD1;NUCB2;NUP155;ONECUT2;OPA3;OPRL1;PAFAH2;PARP16;PBX2;PCGF3;PDE12;PDGFB;PDLIM5;PDP2;PDZD8;PEG10;PEX11B;PGM2L1;PGRMC1;PHACTR4;PIK3C2A;PLAGL2;PLCG2;PLD3;PLEKHA3;PLEKHO1;PLXND1;PM20D2;PMAIP1;PMPCA;POLL;POLR2D;POLR3D;POTEG;POTEM;PPP1R15B;PPP2R2A;PRIM2;PRR5-ARHGAP8;PRSS22;QDPR;RAB11FIP4;RAB19;RAB40C;RABL2A;RABL2B;RAD18;RBFOX2;RBM12B;RDX;RFC2;RHBDF2;RHD;RNF144B;RNF44;RNFT1;RRAD;RRM1;RRM2;RWDD1;SALL3;SAR1A;SDR42E1;SEMA4C;SLC10A7;SLC11A2;SLC12A7;SLC16A

			9;SLC19A3;SLC20A1;SLC38A7;SLC5A6;SMARCAD1;SMC1A;SMCR8;SNX17;SOCS1;SOCS4;SOD2;STAT2;STK4;STRN;STX3;SUMO1;SUOX;SURF4;SYNJ2BP;SYT1;TBC1D19;TGFBR3;TGOLN2;THBS1;THEM6;THYN1;TIAF1;TLR4;TMED4;TMED5;TMTC3;TNFSF9;TNS3;TOMM40L;TRAPPC10;TRIM71;TRMO;TSC22D2;TUBB2A;TUBB4A;TXLNA;TXLNG;UBXN2B;USP38;USP47;VCL;WASL;YAE1D1;YOD1;YWHAZ;ZBTB37;ZBTB5;ZBTB8OS;ZC3HAV1L;ZCCHC3;ZFAND4;ZNF200;ZNF264;ZNF28;ZNF417;ZNF443;ZNF460;ZNF556;ZNF566;ZNF578;ZNF584;ZNF587;ZNF609;ZNF611;ZNF644;ZNF738;ZNF774;ZNF799;ZNF8
hsa-let-7g-5p-478580_mir	-1.506	0.443	ABHD17C;ABT1;ACER2;ACOT9;ACTA1;ADH5;ADIPOR2;AGO1;AHCYL2;AHR;AK4;AKAP11;AKAP8;AKT2;AMD1;ANKRD46;AP1S1;AQP6;AREL1;ARID3A;ARID3B;ARIH1;ARL8B;ATG12;ATG9A;ATP6V1F;ATP6V1G1;ATXN2;ATXN7L3;ATXN7L3B;BACH1;BCL2L1;BEND4;BMI1;BRD1;BRI3BP;BZW1;C11ORF57;C12ORF4;C19ORF47;C19ORF53;C1ORF21;C1ORF210;C1RL;C5ORF51;CALU;CASP3;CASTOR2;CBX5;CCND1;CCNT2;CD59;CDKAL1;CDKN1A;CDKN2A;CDV3;CELF1;CEP120;CEP135;CHTOP;CLDN12;COIL;COL1A2;COL8A1;COLEC12;COX6B1;CPA4;CRX;CRY2;CTPS1;CXCL8;DCAF8;DDR1;DIABLO;DISC1;DNA2;DNAH9;DNAJC28;DNAL1;DTX3L;DUSP1;DVL3;DYRK3;E2F6;ECHDC1;EDEM3;EDN1;EEF1A1;EFHD2;EIF4A3;EIF4G2;EMILIN2;EPHA4;ERO1A;ESPL1;FAM104A;FAM105A;FAM222B;FAM43A;FAM83G;FBXL20;FBXW2;FIGN;FMNL3;FMO4;FN1;FNDC3A;FNDC9;FPR1;FUT10;FXN;FZD9;GAB2;GABPAP;GATM;GGA3;GLO1;GNG5;GOLGA4;GPAT4;GRPEL2;HAND1;HASPIN;HERPUD1;HIST1H2BD;HIST1H2BK;HMGA1;HMGA2;HMGB1;ICOSLG;IFNLR1;IGDCC4;IGF1R;IGF2BP1;IGF2BP3;IKZF3;IL13;IL6R;INTS7;IPO9;ITGA3;KCTD21;KIAA0391;KIAA0930;KIAA1143;KIAA1328;KIF27;KLHDC8B;KMT2D;KPNA5;KRAS;KREMEN1;LEFTY1;LIMD2;LRIG3;LRRC20;LYN;MAGEA12;MAGEA3;MAGEA6;MAP2K7;MAP3K1;MAPK6;MARCKSL1;MARS2;MBD2;MCF2L2;MDM4;MEF2D;MEIS3P1;MFSD8;MIDN;MIEF1;MLLT10;MRPL12;MSI2;MTUS1;MTX3;MXD1;MYC;NAA20;NAA30;NAP1L1;NAT8L;NCKIPSD;NCOA3;NDUFA4P1;NHLRC2;NHLRC3;NOA1;NOM1;NR6A1;NSD1;NUCB2;NUP155;ONECUT2;OPA3;OPRL1;PAFAH2;PARP16;PBX2;PCGF3;PDE12;PDGFB;PDLIM5;PDP2;PDZD8;PEG10;PEX11B;PGM2L1;PGRMC1;PHACTR4;PLAGL2;PLCG2;PLD3;PLEKHA3;PLEKHO1;PLXND1;PM20D2;PMAIP1;PMPCA;POLL;POLR2D;POLR3D;POTEG;POTEM;PPP1R15B;PPP2R2A;PRIM2;PRR5-ARHGAP8;PRSS22;QDPR;RAB11FIP4;RAB19;RAB21;RAB40C;RABL2A;RABL2B;RAD18;RANBP2;RBFOX2;RBM12B;RDX;RFC2;RHBDF2;RHD;RNF144B;RNF44;RNFT1;RPL12;RRAD;RRM1;RRM2;RWDD1;SALL3;SAR1A;SART3;SDR42E1;SEMA4C;SFT2D1;SLC10A7;SLC11A2;SLC12A7;SLC16A9;SLC19A3;SLC20A1;SLC38A7;SLC5A6;SMAD2;SMARCAD1;SMC1A;SMCR8;SNX17;SOCS1;SOCS4;SOD2;STAT2;STK4;STRN;STX3;SUMO1;SUOX;SURF4;SYNJ2BP;SYT1;TBC1D19;TBC1D9;TGFBR1;TGFBR3;TGOLN2;THBS1;THEM6;THYN1;TIAF1;TMED4;TMED5;TMTC3;TNFRSF10B;TNFSF9;TOMM40L;TRAPPC10;TRIM71;TRMO;TSC22D2;TUBB2A;TUBB4A;TXLNA;TXLNG;UBE3C;UBXN2B;USP38;USP47;VCL;WASL;YAE1D1;YOD1;YWHAZ;ZBTB37;ZBTB5;ZBTB8OS;ZC3HAV1L;ZCCHC3;ZFAND4;ZNF200;ZNF264;ZNF28;ZNF417;ZNF443;ZNF460;ZNF556;ZNF566;ZNF578;ZNF584;ZNF587;ZNF609;ZNF611;ZNF644;ZNF738;ZNF774;ZNF799;ZNF8
hsa-miR-92a-3p-477827_mir	-0.589	0.452	AAED1;AAGAB;AARS2;ABCA3;ABCF2;ABHD15;ABHD2;ABL2;ACAA1;ACACA;ACADS;ACAT2;ACLY;ACOD1;ACTB;ACTC1;ACTR1A;ADAM10;ADAMTS1;ADRM1;AEN;AFDN;AGBL5;AGMAT;AGO1;AGO4;AGPAT5;AHCYL1;AHCYL2;AIDA;AK2;AKAP10;ALDH9A1;ALG14;ALKBH4;ALKBH5;ALMS1;AMD1;AMMECR1;AMOTL1;ANKH;ANKIB1;ANKLE2;ANKS1A;ANP32E;ANXA11;AP2B1;AP3D1;AP3S2;AP5Z1;APOBEC3C;APOBEC3F;APOLD1;APPL1;ARCN1;ARF1;ARFGEF2;ARGFX;ARHGDIA;ARHGEF25;ARID1A;ARID1B;ARID4B;ARL2;ARNTL2;ARPC2;ARPP19;ARRB1;ASAH1;ASGR2;ASS1;ATAD3A;ATF7IP;ATG16L1;ATM;ATOX1;ATP11A;ATP13A1;ATP1A1;ATP2A2;ATP2B4;ATP5A1;ATP5B;ATP5J;ATP7A;ATXN1;ATXN2L;ATXN7;AUP1;AURKA;AURKB;AXIN1;B4GALT2;B4GALT7;BAG6;BAHCC1;BAK1;BAZ2B;BBX;BCAT1;BCAT2;BCL11B;BCL2L11;BCL9;BCL9L;BCS1L;BFAR;BICD2;BID;BLMH;BMP8A;BMPR1A;BMPR2;BPTF;BRCC3;BRD2;BRF1;BRMS1L;BSG;BTAF1;BTBD7;BTF3;BTG2;BTRC;C11ORF24;C11ORF57;C12ORF57;C15ORF38-AP3S2;C17ORF51;C17ORF75;C1GALT1C1;C1ORF174;C1ORF35;C21ORF91;C2ORF69;C5ORF24;C5ORF42;C6ORF62;C9ORF64;CA5B;CACFD1;CACUL1;CAMKV;CAMTA2;CAPRIN1;CAPZB;CARD6;CARM1;CARS;CASC10;CASD1;CASKIN1;CBFA2T2;CBFB;CBR1;CBS;CBX2;CBX5;CBX6;CC2D2A;CCAR1;CCDC113;CCDC171;CCDC181;CCDC186;CCDC57;CCDC6;CCDC86;CCL8;CCNB1;CCND1;CCNE1;CCNI;CCNQ;CCSAP;CCSER2;CCT7;CD180;CD226;CD276;CD2AP;CD2BP2;CD3EAP;CD59;CD69;CDC20;CDC25A;CDC27;CDC37;CDC42SE1;CDC5L;CDC6;CDH1;CDK1;CDK11A;CDK16;CDK5R1;CDK9;CDKN2AIPNL;CDKN3;CDV3;CELSR1;CEP85;CEP97;CERS1;CES2;CHAF1A;CHCHD10;CHCHD2;CHCHD5;CHEK1;CHN1;CHST1;CHST9;CHTOP;CIC;CIDEC;CISD1;CIT;CKB;CKMT1B;CLDND1;CLIC1;CLN8;CLNS1A;CLSPN;CLTA;CLTC;CMTM6;CNEP1R1;CNIH1;CNNM4;CNOT1;CNOT2;CNOT4;COA7;COG3;COL18A1;COL4A1;COL4A2;COPG1;COPS6;CORO7;COX18;COX20;COX4I1;CPEB2;CPEB3;CPEB4;CPSF6;CPTP;CRBN;CREB3L2;CRTC2;CRTC3;CSDE1;CSPG5;CSTF2T;CTC1;CTDSPL;CTLA4;CTNNBL1;CTSB;CTTN;CUL5;CUX1;CXCL16;CXORF56;CYB5R3;CYBRD1;CYP20A1;C

			YP2C19;CYP4V2;CYP7A1;CYTH2;DAB2IP;DAND5;DAZAP1;DBT;DCAF12;DCAF16;DCP2;DCTD;DDAH1;DDI2;DDIT4;DDR2;DDRGK1;DDX17;DDX23;DDX39A;DDX3X;DDX5;DDX56;DDX6;DEDD2;DENND2C;DENND4B;DENND5A;DENND6A;DHCR24;DHRS3;DHRS7B;DHTKD1;DHX16;DHX30;DHX8;DIAPH1;DIDO1;DLX6;DMXL1;DNAJA3;DNAJB12;DNAJB9;DNAJC21;DNAJC27;DNAJC30;DNM2;DNMT1;DNPH1;DOCK9;DOPEY2;DOT1L;DSTYK;DTL;DTWD2;DUS2;DUSP10;DUSP11;DUSP5;DYNC1H1;DYNC1LI2;DYNLL1;DYNLT3;DYRK1A;E2F3;E4F1;EARS2;EBP;EDC4;EDEM1;EDRF1;EEF1A1;EEF2;EFNA5;EFNB1;EFTUD2;EGLN2;EHBP1;EID2B;EIF1;EIF2AK1;EIF2B2;EIF2S3;EIF3B;EIF3C;EIF3G;EIF3I;EIF3K;EIF4B;EIF4EBP1;EIF4EBP2;EIF4G2;EIF5A2;EIF6;ELAC2;ELF4;ELOA;ELOB;ELOF1;ELOVL6;ELP1;EMD;ENO1;ENOPH1;ENTHD1;EP300;EPB41L3;EPHA4;EPHA7;EPHB2;EPM2AIP1;ERAP1;ERGIC1;ERGIC2;ERH;ESD;ESR2;ESRP1;ETFA;ETV6;EVI5;EXOC5;EXOSC6;EZR;F11R;F8A3;FAF1;FAM126B;FAM129A;FAM129B;FAM135A;FAM136A;FAM160B2;FAM168B;FAM189B;FAM20C;FAM3A;FAM46A;FAM49A;FAM69B;FAM83G;FAM91A1;FAR1;FASLG;FASN;FAU;FBXL19;FBXO45;FBXW2;FBXW7;FCHO2;FGF2;FHOD1;FKBP14;FKBP1A;FKBP4;FKBP8;FKBP9;FKBPL;FLCN;FLNA;FLNB;FLYWCH1;FMN1;FNDC3B;FNIP1;FOPNL;FOXN2;FRAT2;FTSJ1;FUK;FUT10;FUT11;FXR1;FYCO1;FZD6;G2E3;G3BP2;GAA;GABARAPL2;GABPA;GAK;GALNT7;GAN;GANAB;GAPDH;GARNL3;GATA3;GATA6;GATAD2A;GATAD2B;GBP4;GCHFR;GCNT3;GDPD5;GEMIN2;GEMIN6;GFPT2;GGA2;GGCX;GHITM;GID4;GIT2;GLO1;GLOD4;GLYR1;GM2A;GNAQ;GNG7;GNPNAT1;GOLGA3;GOLGA4;GOLGA8A;GOLGA8B;GOLGA8IP;GOLGA8J;GOLIM4;GOLM1;GOT2;GPBP1L1;GPR89A;GPX3;GRAMD1B;GRAMD2B;GRAMD4;GSK3B;GSTP1;GTF2A1;GTF2E1;GTF3C1;GTF3C2;GUF1;GULP1;GXYLT1;H1FX;H3F3B;H3F3C;HACD4;HADHA;HAUS1;HBB;HCFC1;HDAC1;HDAC2;HDGF;HEATR1;HEATR6;HECTD1;HELQ;HERC1;HES1;HGH1;HIP1R;HIPK1;HIPK3;HIST1H1E;HIST1H2AB;HIST1H2AC;HIST1H2AE;HIST1H2AH;HIST1H2AM;HIST1H3D;HIST1H4C;HIST2H2AA3;HIVEP1;HLA-E;HMGA2;HMGB1;HMGCR;HMGCS1;HMGXB3;HNRNPA2B1;HNRNPF;HNRNPLL;HNRNPM;HOGA1;HOXA13;HOXA5;HOXC8;HP1BP3;HS3ST3A1;HSD17B10;HSP90AB1;HSP90B1;HSP90B2P;HSPA1B;HSPA8;HSPBP1;HSPH1;IARS2;IBTK;ICAM1;IER3IP1;IFITM1;IFT172;IFT22;IGF1R;IGSF1;IK;IKZF1;IKZF2;IL17RA;IL6ST;ILF2;INCENP;INSIG1;IPO4;IPO5;IPO7;IPP;IQGAP1;IRAK1;IRF2BPL;IRGQ;ISM2;ITGA5;ITGB8;ITPR1;ITPR3;JAG2;JMJD6;JOSD1;KANK2;KANSL2;KANSL3;KAT2A;KAT2B;KCNC1;KCNC4;KCTD5;KCTD7;KDELR1;KDM3B;KDM6B;KDSR;KHDRBS1;KHNYN;KHSRP;KIAA0100;KIAA0556;KIAA1109;KIAA1468;KIAA1549;KIAA1586;KIAA1671;KIAA1958;KIF18B;KIF1A;KIF1B;KIF1BP;KIF20A;KIF20B;KIF2C;KIF5B;KIFC1;KLC2;KLF2;KLF4;KLHDC10;KLHL11;KLHL14;KLHL15;KLHL18;KLHL3;KLHL36;KLHL42;KMT2D;KMT5B;KPNA2;KPNA3;KPNA6;KPNB1;KPTN;LAMC1;LAMP2;LARP1;LARS;LASP1;LAX1;LCOR;LDLR;LDLRAP1;LETM1;LHFPL2;LILRA2;LIMD1;LINC00598;LMBR1L;LMNB2;LONRF3;LPIN1;LRIG1;LRP6;LRRC27;LRRC37A2;LSM7;LTBP1;LXN;LY6G5B;MACF1;MAFG;MAML1;MAN2A1;MAP1B;MAP2K4;MAP3K4;MAP3K6;MAP4;MAPK1IP1L;MAPK8;MAPK9;MAPRE1;MARS;MAST3;MAU2;MAVS;MBD2;MBNL1;MCCD1;MCF2L2;MCL1;MCM3;MCM7;MCOLN2;MDM2;MDN1;MECP2;MED16;MED19;MED29;MED7;MEF2C;MEF2D;MEN1;METTL16;METTL2A;METTL2B;MFF;MFN1;MFN2;MFSD1;MIA3;MIGA2;MINK1;MKI67;MKNK2;MKRN2;MLF2;MLX;MOAP1;MORC3;MPG;MRGBP;MRO;MRPL19;MRPL2;MRPL3;MRPL32;MRPL9;MRPS16;MRPS21;MRS2;MSL3;MT-CO1;MTF1;MTFP1;MTHFSD;MTMR1;MTMR10;MTMR14;MUC21;MYBBP1A;MYBL2;MYC;MYCBP2;MYEF2;MYH2;MYH9;MYL6;MYLIP;MYO19;MYO1C;MYO1D;MYO5A;MYO5C;MYO6;MYZAP;NAA25;NAB1;NACC2;NADK;NAPRT;NARF;NBR1;NCAPG2;NCAPH;NCDN;NCL;NDRG3;NDST1;NDUFA5;NDUFA7;NDUFB10;NDUFV3;NECAP1;NEK2;NEK6;NEMP1;NF2;NFATC2IP;NFE2L1;NFIB;NFKB1;NFYB;NHSL1;NIPSNAP2;NKAP;NKX3-1;NLE1;NLRP9;NMD3;NOC2L;NOL4L;NOTCH2;NPC2;NPLOC4;NPM1;NPTN;NPY4R;NR1H4;NRAS;NRXN3;NSD2;NSF;NSFL1C;NSMAF;NT5DC3;NTHL1;NTPCR;NUCB1;NUCKS1;NUDCD3;NUDT19;NUFIP2;NUGGC;NUP155;NUP205;NUP210;NUP43;NXN;OAZ1;OCIAD2;OGFR;OGT;OPA3;OPTN;OR2A4;ORAI2;ORC5;ORC6;OSBPL2;OSBPL8;OSMR;OTUD3;OTUD6A;OTUD7B;P4HB;PA2G4;PABPN1;PAFAH1B1;PAIP1;PANK2;PAPD5;PAPD7;PARD6B;PARK7;PAWR;PAX9;PBLD;PBX2;PCBD2;PCBP2;PCGF5;PCMTD1;PCNX4;PDAP1;PDCD6IP;PDE4DIP;PDIA4;PDIK1L;PDLIM1;PDS5B;PDXK;PDZD8;PEAK1;PEBP1;PELP1;PER2;PFDN2;PFDN6;PFKM;PFN1;PGAM4;PGPEP1;PHACTR4;PHF12;PHF13;PHLPP1;PHLPP2;PHTF2;PIGS;PIK3AP1;PIK3CD;PIP5K1C;PITPNA;PITRM1;PITX1;PKDCC;PKM;PKN1;PKNOX1;PLA2G4F;PLCG1;PLCXD2;PLEKHA1;PLEKHB1;PLEKHH3;PLXNA3;PMEPA1;PNO1;POGZ;POLE;POLI;POLK;POLR2I;POLR2L;POLR3A;POLRMT;POMGNT1;PON2;POP4;POR;POTEG;PPAN;PPARD;PPCS;PPIB;PPIC;PPIL1;PPP1CC;PPP1R12C;PPP1R37;PPP1R3D;PPP1R3G;PPP2R1A;PPP2R2A;PPP4C;PPP6C;PPP6R2;PRC1;PRELID1;PRKCA;PRPF8;PRPS1;PRR12;PRR14L;PRRC2B;PRRG4;PSMA4;PSMA7;PSMB1;PSMB6;PSMC3;PSMD11;PSMD2;PSMD3;PSMD5;PSME3;PSMF1;PSMG3;PTAR1;PTBP1;PTEN;PTGER4;PTGES2;PTGFRN;PTK7;PTP4A2;PTPN13;PTPRJ;PTS;PURB;PURG;PWP2;PXN;PYGL;QSER1;QSOX2;QTRT1;R3HDM4;RAB11A;RAB36;RAB3D;RAB7B;RAB8A;RAB8B;RABGAP1;RABGGTB;RAD21;RAD23B;RA

			D51;RANBP2;RANBP6;RANGAP1;RARS;RASA1;RASA3;RASAL2;RAVER1;RBBP7;RBFOX2;RBL2;RBM10;RBM27;RBM28;RBMS2;RBPJ;RBSN;RBX1;RCAN1;RCC1;RCC2;RCN3;RECQL;REL;REV3L;REXO1;RFFL;RGS5;RHEB;RHOT2;RIC8A;RIOK1;RIOX2;RLIM;RNASEH1P1;RNF103;RNF121;RNF123;RNF128;RNF2;RNF4;RNF40;RNF44;RNPS1;RORA;RPA2;RPAIN;RPL11;RPL13A;RPL15;RPL18A;RPL22;RPL23;RPL24;RPL27;RPL3;RPL39;RPL7A;RPL8;RPL9;RPLP1;RPRD2;RPS10;RPS14;RPS15;RPS15A;RPS23;RPS24;RPS25;RPS28;RPS3A;RPS5;RPS8;RRBP1;RRM1;RRN3;RRP9;RSBN1;RSPRY1;RTCB;RTL10;RUVBL2;RXRB;SACM1L;SAFB;SAP30;SAP30BP;SARAF;SARS2;SART3;SASH1;SCAF11;SCAF8;SCARB1;SCARB2;SCD;SCD5;SCLY;SCML2;SCYL2;SEC14L1;SEC24A;SELENBP1;SELENOT;SERINC1;SERTAD2;SERTAD3;SESN3;SETD1B;SETD5;SETDB1;SF3A3;SF3B3;SGK3;SGPP1;SH2B3;SH3PXD2A;SHC3;SHE;SHISA5;SHMT2;SHOC2;SHPRH;SIK1;SIN3A;SIN3B;SIPA1L1;SIRT1;SKI;SLAIN1;SLC10A7;SLC12A4;SLC12A5;SLC20A1;SLC25A16;SLC25A3;SLC25A32;SLC25A33;SLC25A36;SLC25A38;SLC25A44;SLC2A6;SLC33A1;SLC37A3;SLC39A14;SLC39A6;SLC3A2;SLC4A2;SLC6A8;SLC7A1;SLC7A11;SLC9A1;SLX4;SMAD4;SMAD6;SMAD7;SMAP2;SMARCA5;SMARCD2;SMC1A;SMC3;SMG1;SMU1;SNF8;SNN;SNRNP70;SNRPD1;SNRPE;SNU13;SNX10;SNX9;SOCS5;SOX11;SOX4;SPATS2L;SPCS3;SPEN;SPOCK2;SPRYD4;SPTLC1;SREBF2;SREK1IP1;SRF;SRFBP1;SRPRA;SRPRB;SRRT;SRSF5;SSFA2;SSX2IP;STARD7;STAT2;STAT3;STEAP3;STK25;STK4;STMN3;STX3;SUCLG1;SUCLG2;SUGP1;SUPT5H;SUPT7L;SURF4;SURF6;SV2A;SYNJ1;SYNPR;SZRD1;TACC1;TAF8;TALDO1;TANC2;TANK;TAOK2;TATDN3;TAX1BP3;TBC1D8;TBC1D9B;TBCEL;TBL1X;TCHP;TCOF1;TECPR2;TEF;TERF2;TESK1;TET2;TEX10;TFAM;TFG;TFPI;TGFA;TGFBR2;TGFBRAP1;THBS1;THUMPD1;TIRAP;TLE3;TLN1;TLR10;TLR3;TMA16;TMCC3;TMED9;TMEM160;TMEM161A;TMEM184B;TMEM239;TMEM33;TMEM41A;TMEM44;TMF1;TMUB1;TNFRSF10B;TNFRSF13C;TNPO1;TNRC18;TOB1;TOB2;TOM1L1;TONSL;TOR1B;TOR4A;TP63;TPD52L3;TPM3;TPPP;TPRN;TPT1;TRAF3IP1;TRAF4;TRAM2;TRAPPC11;TRAPPC13;TRAPPC2;TRIM28;TRIM36;TRIO;TRIP12;TRMT2B;TRMT61A;TSC1;TSPAN18;TSPAN31;TSR1;TTC32;TTC37;TTC5;TTC7B;TTLL7;TUBA1C;TUBB;TUBB2B;TUBB3;TUBB4B;TUBGCP2;TUFM;TULP4;TUSC2;TUT1;TWF1;TWISTNB;TXLNA;TXNDC15;TYMP;U2AF2;UBAC2;UBAP2L;UBE2H;UBE2R2;UBE2Z;UBE4B;UBR1;UBXN4;UCHL1;UCK2;UFSP2;UGDH;UHRF1BP1;UNC13B;UPF2;UQCRFS1;URB2;USP10;USP13;USP28;USP31;USP9X;UVRAG;VANGL2;VARS;VBP1;VCPIP1;VDAC2;VHLL;VMA21;VPS4B;VPS54;WASL;WDR1;WDR18;WDR19;WDR26;WDR37;WDR6;WDR81;WDR83OS;WEE1;WIPF2;WNT5A;WRNIP1;XKR7;XPO1;XPOT;XPR1;XRN1;XYLT2;YBX3;YIF1B;YIPF4;YIPF5;YWHAQ;ZADH2;ZBTB22;ZBTB34;ZBTB8B;ZC3H18;ZC3HAV1;ZC3HAV1L;ZCCHC2;ZDHHC21;ZDHHC24;ZDHHC5;ZFC3H1;ZFHX4;ZFP62;ZFYVE16;ZFYVE21;ZFYVE26;ZIC5;ZMYND8;ZNF134;ZNF157;ZNF17;ZNF174;ZNF224;ZNF24;ZNF264;ZNF267;ZNF276;ZNF277;ZNF282;ZNF317;ZNF354B;ZNF383;ZNF417;ZNF420;ZNF430;ZNF460;ZNF492;ZNF503;ZNF507;ZNF551;ZNF598;ZNF607;ZNF622;ZNF629;ZNF687;ZNF695;ZNF703;ZNF721;ZNF75A;ZNF772;ZNF776;ZNF785;ZNF84;ZNF850;ZNF98;ZNRF3;ZSCAN12;ZWINT
hsa-miR-18a-5p-478551_mir	-2.029	0.455	ABR;ACAP3;ACIN1;ACOD1;ACTL6A;ADAM15;ADGRF2;AFF4;AGFG1;AHI1;AHRR;ALG14;AP3S2;ARF6;ARVCF;AS3MT;ATM;ATP1A1;B4GALT1;BCL2;BCL2L10;BDH1;BVES;C15ORF38-AP3S2;CA12;CA13;CANX;CCND1;CCNL1;CCT6A;CDC20;CDCA5;CDK19;CIZ1;CKAP5;CLN8;CNTN1;COX7B;CRB1;CREBL2;CRIM1;CSRNP3;CTDSPL;CTGF;CUL4A;CXORF40B;DAAM2;DCAF4;DCAF7;DCAF8;DCTN2;DCTN5;DCUN1D3;DDAH1;DDX5;DIAPH1;DICER1;DNMT1;EEF1A1;EEF2;EFCAB14;EIF4EBP2;ERRFI1;ESR1;ETFA;ETS2;EXPH5;EZR;FAM104A;FAM120C;FAM173B;FAM3C;FAS;FBXL3;FCGR2B;FEM1C;GABPA;GATAD2A;GCH1;GDAP2;GIGYF1;GLRX5;GNAS;GNL1;HERC1;HIF1A;HMGCS1;HNRNPR;HOXA9;HSF2;ID4;IFNAR2;IGF2BP2;IGHMBP2;IRF2;IRGQ;IRS4;ISCU;ITGA2;ITM2C;JMY;KIAA0100;KIAA0907;KLHL3;LARGE1;LIN54;LMNB2;LRIG3;LRRC41;MAP7D1;MAPK13;MATR3;MBD1;MCM5;MCRIP2;MDC1;MEF2D;MID1;MOB4;MRPL35;MSL3;MXD3;MYLK;NACC1;NBEA;NCOA3;NCOA6;NEDD9;NEO1;NR1I2;NR3C1;ORAI3;PAFAH1B1;PAPSS2;PARD6B;PCDH9;PCNA;PCNP;PDE4D;PDIK1L;PELP1;PHLPP1;PI4KB;PIAS3;PKMYT1;PLA2G2F;PNISR;PNPO;POGK;PON2;PPIA;PPP1CB;PSAT1;PSMB5;PTCHD1;PTEN;RAB23;RAB5A;RAB5C;RABAC1;RACK1;RAD51AP1;RAP1A;RASSF3;RBM38;RBPJ;RDH10;RICTOR;RLIM;RNF138;RNF146;RNF157;RNF4;RNLS;RORA;RPL7A;RPL9;RPS6KA5;RTL8A;RUNX1;SAR1A;SASS6;SDC4;SEC24B;SEC61A1;SERBP1;SERTAD3;SLC25A28;SLC25A37;SLC36A1;SMAD2;SMAD3;SMAD4;SMCHD1;SON;SOX12;SP100;SPEN;SRSF6;STK4;STX12;SUPT7L;SYNM;SYPL1;TAOK1;TBPL1;TCEA2;TCP1;TGFBR2;THRA;TIMM50;TLE3;TMEM2;TMUB2;TNFAIP3;TNFSF11;TNPO1;TNRC6B;TOMM20;TP53;TRAPPC1;TRIM35;TSC22D3;TSR1;TUT1;TWF1;TXNDC15;TXNIP;UBC;UBE2G1;UBL5;UBQLN4;UBTD2;UTP14C;VCP;VMA21;VPS18;VPS37A;WAC;WDR82P1;WWTR1;XYLT2;YBX3;ZBTB47;ZC3H18;ZFAND5;ZFP36L2;ZMYM3;ZMYND8;ZNF132;ZNF33A;ZNF367;ZNF460;ZNF507;ZNF585B;ZNF670;ZNF678;ZNF703;ZNF708;ZNF711;ZNF770;ZSWIM6
hsa-miR-376a-3p-478240_mir	-0.569	0.458	ACVR1C;AGO2;AMOTL2;ATG2A;ATG4C;B3GALT5;BEND4;BTF3L4;C1ORF123;C1ORF216;C2CD4A;C4ORF19;CASP8;CASTOR2;CD164;CD2AP;CDK2;CNTROB;COPA;CXCR6;CYP27C1;DCAF17;DCTN5;E2F3;ERO1B;FGF2;FOXG1;FOXK1;FOXO1;GNAS;G

			NAT1;HCFC2;HNRNPA0;IAPP;IGF1R;IL7;INA;IQGAP1;ITGB8;KCND3;KIAA1671;KIF5C;KLF15;LEPROT;LRRC58;LRRC6;MAD2L1;MEPE;MRPL51;MYRF;MYSM1;NDUFS3;NIP7;PARD6B;PDS5A;PFKFB2;PIGM;PIK3R1;PIKFYVE;POTEG;POTEM;PPM1A;PPP3R1;PRKCH;RAB15;RAB21;RANBP6;RIMS2;RNF216;RPP14;RS1;SAMD8;SERINC3;SHC3;SLC16A1;SLC35G2;SMAD7;SNRPB;SORT1;SRRT;SRSF11;STON2;TMEM245;TNKS2;TTK;UBE2A;UBE2D2;WDR17;WIPI2;YAF2;ZFHX3;ZFP69B;ZNF169;ZNF180;ZNF253;ZNF266;ZNF281;ZNF426;ZNF439;ZNF440;ZNF487;ZNF567;ZNF594;ZNF667;ZNF669;ZNF699;ZNF736;ZNF780B;ZNF781;ZNF791;ZNF844;ZNRF3
hsa-miR-433-3p-478102_mir	5.982	0.465	AFMID;ALDOA;ATXN1;AZIN1;BBS2;BRWD1;C5ORF22;CFTR;CHD9;COX6B1;COX8A;CREB1;DCBLD2;DGKE;EDN1;ENTPD4;ERBIN;FAM126B;FAM3C;FGF20;FGF9;FKBP1A;FKBP1C;GABPAP;GBP2;GPR135;GRB2;HACD3;HAUS2;HIF1A;HIVEP1;HRH4;HSP90B1;ITIH5;KLHL4;KRAS;LEMD2;LONRF2;LONRF3;LRIG3;MAFK;MAPK8;MBNL1;MDM4;MET;MRPS25;MTMR9;NEK9;NINJ1;NSA2;NUBPL;PDLIM3;PLEKHA1;POTEG;POTEM;RAB5C;RPL24;RUNX2;SCAMP1;SCNN1G;SERBP1;SLC1A5;SLC28A1;SMU1;SPATS2L;SPRYD4;SSC5D;STARD7;STK38;TCF20;TFAP2A;TGIF2LX;TM4SF5;TMEM229B;TYMS;UGT2B4;ULBP2;WDR45B;YIPF6;ZNF584;ZNF780A
hsa-miR-342-3p-478043_mir	-2.093	0.465	ABL1;ACOT9;ACOX1;ACTG1;ACVR1B;AIFM2;AK4;ANKRD49;ANTXR2;APTX;ARF3;ARIH2;ASAH2;ASAH2B;ASB3;ATF4;ATG10;ATP6V0E1;ATP6V1G3;ATXN7;BHLHB9;BIRC6;BMP7;BPTF;C1QTNF6;C2ORF72;CALR;CAMK2N1;CANX;CAVIN1;CBX1;CCDC59;CCDC6;CCND2;CD3D;CDK6;CHD9;CLN8;CLSTN3;CPEB3;CPEB4;CRB2;CRIM1;CRLF1;CTBP2;CXADR;CYP4F11;DARS;DCAF12;DDX3X;DIAPH1;DNAJC9;DNMT1;DUSP6;E2F1;EEF1A2;EEF2;EGR3;EID1;EIF1AD;EMC7;EPB41;EPHB3;ERC1;ESCO1;EXOSC1;F11R;F9;FAM107A;FAM180B;FAM3C;FIBP;FIGN;FN3KRP;FNDC3B;FOSL2;FOXJ3;FPR1;FRMD3;G3BP1;GALNT10;GART;GEMIN4;GGCX;GID8;GJA1;GNL3L;GOT2;GPR75-ASB3;GRID1;GTF2H2C;HARS;HDAC9;HIPK3;HIST1H2BJ;HMBOX1;HNRNPC;HOXB3;HSP90B2P;HUNK;ID4;IDH3B;IDS;IFNAR2;IGF1R;IGF2R;IGFBP5;IKBKG;IKZF3;INO80D;INSIG1;IREB2;ISOC2;JUN;KCNK1;KCTD15;KCTD16;KIF13A;KIF3A;KLF17;KLF8;KLHL15;KPNA2;LETMD1;LHFPL3;LIPG;LMX1A;LONRF1;LRPAP1;LSAMP;LY6G5B;MAPKBP1;MBNL3;METTL2A;MLLT6;MLXIP;MMAB;MPIG6B;MRRF;MTDH;MTRF1L;MXI1;NABP2;NAPG;NBPF10;NBPF14;NCAN;NDRG1;NETO2;NEURL1B;NFASC;NGRN;NOP2;NUDT19;OR7D2;OSMR;P2RX3;PA2G4;PAGR1;PGPEP1;PHKG2;PKNOX1;PKP1;PLA2G4A;PLA2G7;PLD6;PLEKHA2;PLXNA4;POLR1B;POU2F3;PPIE;PPM1D;PPM1L;PRKCE;PRKCSH;PSAT1;PTGIS;PTPN14;PTPRN2;PUS10;PVRIG;PWP2;PXDN;QRFPR;RAB32;RABGAP1;RAD23B;RAD54L2;RALGAPB;RALGPS1;RBBP4;RCBTB1;RFTN2;RFX3;RGS4;RHOBTB3;RILPL1;RMND5A;RNF152;RPAP2;RPL15;RPL26;RPL27A;RPL37A;RPS3A;RPS9;RPSAP58;RRAGD;RRM2;SELPLG;SEPHS1;SETD7;SF3B2;SF3B3;SH3PXD2A;SHC3;SHOC2;SIGLEC15;SLC25A26;SLC30A9;SLC41A2;SLC6A12;SLITRK4;SNTN;SNX1;SOD2;SPAG7;SREBF1;SREBF2;STRN;STRN3;STX3;SYNPO2L;SYT5;TAB2;TAB3;TACC1;TAF8;TAL1;TCF3;TCP1;TCTE1;TESPA1;TFRC;TIAM1;TIMM10B;TJAP1;TLDC1;TM9SF4;TMED9;TMEM105;TMEM120B;TMEM236;TMEM33;TMEM98;TMLHE;TNFSF8;TNS3;TOMM70;TP53INP2;TRAPPC2L;TRERF1;TRMT61A;TTC9C;TUT1;TXNDC5;UBE2H;UBTF;UBXN7;UGDH;USP51;UTP4;VAPA;VAPB;VPS35;WAC;WDR73;XPOT;YME1L1;YWHAQ;ZBTB39;ZBTB46;ZC3H12B;ZC3H12C;ZDHHC20;ZDHHC22;ZFC3H1;ZNF175;ZNF268;ZNF280B;ZNF330;ZNF419;ZNF430;ZNF486;ZNF548;ZNF610;ZNF623;ZNF766;ZNF780A;ZSCAN29
hsa-miR-32-5p-478026_mir	0.523	0.473	AAED1;ABCF2;ACAA1;ACOD1;ACTC1;ADAM10;AEN;AGBL5;AGMAT;AGTPBP1;AKAP10;ALG14;AMD1;ANKIB1;ANO8;ANP32E;AP3S2;AP5Z1;APOBEC3F;APPL1;ARF1;ARFGEF2;ARGFX;ARGLU1;ARID1B;ARNTL2;ARRDC4;ASAP1;ASGR2;ATF7IP;ATOX1;ATP2A2;ATP2B4;ATP7A;ATXN1;AURKA;B4GALT7;BAK1;BAZ2B;BCAT1;BCAT2;BCL11A;BCL11B;BCL2L11;BICD2;BMP8A;BMPR1A;BMPR2;BPTF;BRMS1L;BTBD2;BTG2;C11ORF24;C15ORF38-AP3S2;C17ORF75;C1GALT1C1;C1ORF35;C21ORF91;C2ORF69;C5ORF24;C6ORF62;CAPZB;CARD6;CASD1;CASKIN1;CCDC113;CCDC171;CCDC186;CCNB1;CCNE2;CCSER2;CCT6A;CD180;CD226;CD2AP;CD69;CDC27;CDC5L;CDC6;CDK16;CDK5R1;CHST1;CIC;CIDEC;CLN8;CLTA;CNEP1R1;CNIH1;CNNM4;CNOT2;CNOT4;COG3;COX20;CPEB2;CPEB3;CPEB4;CPTP;CREB3L2;CREB5;CRKL;CTDSPL;CYP20A1;CYP2C19;CYTH2;DAB2IP;DAND5;DBT;DDI2;DDIT4;DDX3X;DENND2C;DENND4B;DNAJB12;DNAJB9;DNAJC27;DNAJC30;DOCK9;DSC2;DST;DSTYK;DUS2;DUSP10;DUSP5;DYNC1LI2;DYNLT3;E2F3;EARS2;EDEM1;EDRF1;EID2B;EIF1;EIF4EBP1;EIF4EBP2;EIF5A2;ELOA;ENTHD1;EPM2AIP1;ERBIN;ERGIC2;ESRP1;EVI5;EXOC5;F11R;FAM126B;FAM129A;FAM135A;FAM214A;FAM46A;FAM49A;FAM91A1;FAR1;FASLG;FBXO28;FBXW2;FBXW7;FCHO2;FGF2;FKBP14;FKBP1A;FKBP9;FLCN;FMN1;FNDC3B;FNIP1;FOPNL;FOXN2;FUT10;FUT11;FXR1;FZD6;G2E3;G3BP2;GAA;GALNT7;GAN;GATA6;GATAD2B;GCNT3;GEMIN2;GFPT2;GGCX;GID4;GIT2;GLYR1;GM2A;GNAQ;GOLGA3;GOLGA4;GOLGA8A;GOLGA8B;GOLGA8IP;GOLGA8J;GPBP1L1;GRAMD1B;GRAMD2B;GRAMD4;GTF2A1;GTF2E1;GUF1;GULP1;GXYLT1;H3F3B;H3F3C;HAT1;HBS1L;HECTD1;HIVEP1;HMGA2;HMGCR;HOXA13;HOXC8;HP1BP3;IBTK;ICAM1;IFITM1;IFT22;IKZF2;IKZF4;IL6ST;INCENP;INSIG1;IPP;IRGQ;ITGA6;ITGAV;ITGB8;ITM2B;ITPR1;JOSD1;KAT2B;KCNC4;KIAA0556;KIAA1109;KIAA1586;KIAA1958;KIF1B;KIF1BP;KIF

			20B;KIF5B;KLF4;KLHDC10;KLHL14;KLHL15;KLHL18;KLHL42;KMT5B;LAMP2;LAX1;LCOR;LETM1;LHFPL2;LILRA2;LIN54;LIN7C;LONRF3;MAN2A1;MAP1B;MAP2K4;MAST3;MBD2;MCF2L2;MCL1;MCOLN2;MDM2;MED19;MED29;MED7;MEF2D;MFF;MFHAS1;MFN1;MIA3;MIER3;MIS12;MKNK2;MOAP1;MORC3;MPP7;MRO;MRPL17;MRPL19;MRPS16;MRPS21;MRS2;MTF1;MTMR1;MTMR10;MUC21;MYCBP;MYLIP;MYO1D;MYO5A;MYZAP;NARF;NCAPG2;NECAP1;NEFL;NEMP1;NEO1;NF2;NFATC2IP;NFYB;NHSL1;NKAP;NLRP9;NOL4L;NPTN;NPY4R;NR3C1;NRAS;NSF;NUCKS1;NUDT19;NUFIP2;NUGGC;NUP43;NUS1;OPA3;OR2A4;ORAI2;OSBPL8;OSMR;OTUD3;OTUD7B;OTULIN;PAFAH1B1;PAIP1;PAPD5;PAPD7;PARD6B;PAWR;PAX9;PAXIP1;PBLD;PCBD2;PCMTD1;PCTP;PDZD8;PEAK1;PELP1;PER2;PGAM4;PGPEP1;PHLPP2;PHTF2;PIK3AP1;PIK3CD;PIP5K1C;PITPNA;PKNOX1;PLEKHA1;PLXNA3;PMEPA1;PNO1;POLK;PPCS;PPIC;PPP1R12C;PPP1R37;PPP1R3D;PRCP;PRMT5;PRPF40A;PRPS1;PRRC2B;PRRG4;PSMD5;PSME3;PTAR1;PTEN;PTGER4;PTGES2;PTPRJ;PURG;PUS7;QSER1;RAB3D;RAB7B;RAB8B;RAD21;RAD51;RANBP6;RBFOX2;RBL2;RBM27;RBM28;RBMS2;RBPJ;REL;REV3L;REXO1;RGS17;RHPN2;RLIM;RNF103;RNF141;RNF4;RNF44;RPE65;RPL23;RPL24;RPL9;RPLP1;RPRD2;RRN3;RSBN1;SAMD8;SASH1;SBNO2;SCAF11;SELENOT;SERTAD2;SERTAD3;SESN3;SETD5;SETD7;SGK3;SGPP1;SH2B3;SH3PXD2A;SHCBP1;SHE;SIK1;SLC10A7;SLC12A5;SLC25A16;SLC25A32;SLC25A36;SLC33A1;SLC37A3;SLC39A14;SLC39A6;SLC45A3;SLC7A1;SLC7A11;SLC9A1;SLX4;SMAD6;SMAD7;SMARCA5;SMG1;SMU1;SNN;SNRPD1;SNX10;SOX11;SOX4;SPATA2;SPATS2L;SPCS3;SPOCK2;SPRYD4;SRFBP1;SRPRA;SSFA2;STAT2;STYX;SUPT7L;SYBU;SYNJ1;SZRD1;TACC1;TAF8;TANK;TATDN3;TBC1D8;TECPR2;TEF;TESK1;TEX2;TIRAP;TLR3;TMEM184B;TMEM239;TMEM33;TMEM41A;TMEM44;TMF1;TNFRSF13C;TNPO1;TNRC6B;TOB1;TOB2;TOR1B;TOR4A;TPPP;TRAF3;TRAM2;TRIM36;TRIO;TRMT2B;TSC1;TSPAN31;TULP4;TWF1;TWIST1;TXLNA;TXNDC15;UBE2Q2;UBE2W;UBE2Z;UBXN4;UCK2;UGDH;UGP2;UHRF1BP1;UQCRFS1;USP21;USP28;USP45;UVRAG;VCPKMT;VDAC2;VHLL;VMA21;VPS4B;VPS54;WASL;WDR81;WRNIP1;XKR7;XPR1;XRN1;YIPF4;YWHAH;ZADH2;ZBTB34;ZBTB8B;ZC3HAV1L;ZDHHC21;ZDHHC24;ZDHHC5;ZFC3H1;ZFP62;ZFYVE21;ZHX1;ZIC5;ZNF134;ZNF157;ZNF17;ZNF224;ZNF24;ZNF264;ZNF267;ZNF277;ZNF317;ZNF354B;ZNF383;ZNF398;ZNF417;ZNF430;ZNF460;ZNF492;ZNF594;ZNF598;ZNF607;ZNF695;ZNF721;ZNF75A;ZNF772;ZNF850;ZNF98;ZNRF3;ZSCAN12;ZXDA
hsa-miR-214-3p-477974_mir	-1.374	0.480	ABCC12;ABCC6;ABLIM3;ADRA2B;AGO2;AHNAK2;AHSA1;ALPK2;AMER1;AP2B1;AP3B1;AP3M1;ARHGAP10;ARHGAP19-SLIT1;ARL2;ARL8B;ASF1B;ASH1L;ATF4;BANP;BAX;BCAM;BCL2L11;BCL2L13;BCL2L2;BCL2L2-PABPN1;BIRC5;BTBD19;C10ORF76;C16ORF58;C17ORF49;C9ORF3;C9ORF78;CADM1;CALU;CAPN5;CASKIN1;CCL5;CD180;CD274;CDC42SE1;CDK6;CLEC2D;CNIH1;CNNM2;CPD;CPEB4;CPNE7;CPSF2;CRKL;CS;CTNNB1;DAPK1;DKK3;DOCK9;ELFN2;ENTPD1;ERC1;ERGIC2;EZH2;FAM109A;FAM49B;FGFR1;FGFR4;FLOT1;FLOT2;FOXI2;FSCN1;FZR1;GABARAP;GALNT7;GDPD5;GMEB2;GNAL;GSR;HDGF;HEYL;HIGD1A;HOMER2;HSP90AB1;HSPD1;ING4;INSIG1;IPP;JAG1;JAG2;KCNK5;KCTD15;KIF1B;KLF16;KLHL15;KMT2B;KMT5A;LAMA4;LDB1;LDLRAD4;LIN7C;LRTM2;LTF;LUZP1;LZTS1;MAP2K3;MAP2K5;MAPK1;MAPK14;MAPK8;MED23;MEF2C;MIEF2;MINK1;MPDU1;MTHFD2;NAP1L4;NCAN;NCOA3;NDUFAF3;NFIC;NPTN;NRAS;NUFIP2;PABPN1;PAFAH1B2;PAPPA;PARS2;PCBD2;PCGF3;PDXK;PGAM4;PHLPP2;PIGQ;PIM1;PLXNB1;PNPLA6;POLK;POR;POU4F2;PPARGC1B;PPM1L;PPP6R3;PRR14L;PSMD10;PTEN;QKI;RAB15;RAB1B;RAB5B;RASA1;RASSF4;RETSAT;RLIM;RPL7L1;RUBCN;SCAMP4;SCN4B;SEMA4D;SFMBT2;SH3BP4;SHOC2;SIK1;SLC39A11;SLC7A5;SLIT3;SMDT1;SOCS5;SPEN;SRD5A3;SRGAP1;SRGAP2;STK10;STX6;SUFU;SUGT1;TAF1D;TBPL1;TFAP2A;TFAP2C;TMED9;TMEM189;TMEM189-UBE2V1;TMEM248;TNFSF15;TNFSF9;TP53;TPM3;TRAF1;TRAF7;TRIM29;TRPS1;TSEN54;TWF1;TWIST1;UBE2I;UBE2V1;VASP;VAV2;WASF2;WDR81;WSB2;XBP1;YWHAQ;ZBTB10;ZBTB33;ZBTB8B;ZNF417;ZNF641;ZNRF1
hsa-miR-499a-5p-478139_mir	-0.721	0.481	ADO;APOBEC3F;ARHGAP12;ASAP1;ASAP3;AXIN2;BCL2L11;C11ORF54;CDKN1A;CELF1;CFAP65;DGKG;EEF1E1;EIF2AK4;EIF4A2;ETS1;FGD4;FOXO4;GDE1;GXYLT1;HIST1H2AD;HIST1H3B;HNRNPC;INIP;IQGAP2;KPNA5;LEP;LIN54;MAPK10;MARCH6;MC2R;MDM2;METTL7A;MLANA;MTDH;NLN;NRIP1;PAQR5;PDCD4;PMAIP1;PPP6C;PSMB9;RAB5C;RPS4Y1;RTL8C;SIKE1;SLC25A12;SOX6;SSRP1;SUGT1;TFAM;TGOLN2;TMEM178B;TRAF3IP1;TYRP1;UBE2V2;UBN2;UTP18;VAV3;WASHC2C;WWC2;YTHDF1;ZNF107;ZNF844
hsa-miR-29b-3p-478369_mir	1.975	0.505	ABCE1;ADAM12;AGAP1;AKT2;AKT3;AMER1;AMFR;ANGPTL4;AQP4;ASXL2;B4GALT5;BACE1;BBC3;BCL2;BMP1;BMT2;BTG2;C1QTNF6;C21ORF91;C4ORF26;CALM3;CALU;CAND1;CASP8;CBX2;CBX6;CCDC117;CCNA2;CCND2;CCNT2;CCSAP;CD276;CDC23;CDC42;CDC42SE1;CDK6;CECR2;CIT;CLDN1;CNBP;COL10A1;COL15A1;COL1A1;COL2A1;COL3A1;COL4A1;COL4A2;COL4A5;COL4A6;COL5A1;COL5A2;COL5A3;COL6A3;COL7A1;COLEC10;COMMD2;COX7A2L;CRYBG1;CSGALNACT2;CSRNP2;CTC1;CTNNBIP1;DDX6;DENND6A;DNAJB11;DNMT1;DNMT3A;DNMT3B;DOT1L;DSC2;DUSP2;DYNLT1;EDC3;ELAVL1;ELMSAN1;EMP1;ENPP2;ENTPD1;EPHX2;EREG;ESR1;FAM102B;FAM193A;FAM53C;FAM71F2;FBN1;FBRS;FEM1B;FGA;FGB;FGG;F

			JX1;FMNL3;FOS;FRK;FSCN1;GAS2L3;GATA3;GLDN;GLRX3;GOLGA7;GRN;GSK3B;GTDC1;HDAC4;HDGF;HECW1;HMGA2;HMGCR;HP1BP3;ID3;IFIH1;IFNG;IFRD1;IL32;IMPDH1;INSIG1;ISG20L2;ITGA6;ITGB1;KCTD15;KDM2A;KDM6B;KIAA1671;KIF1B;KLHDC3;LAMA2;LAMC1;LAMC2;LASP1;LIMS1;LOX;LOXL2;LOXL4;LRP10;LRP6;MAP2K6;MAPKBP1;MAZ;MCL1;MDM2;MEN1;METTL15;MKI67;MMP15;MMP2;MMP24;MMP9;MORF4L1;MORF4L2;MRM3;MRPS35;MXD1;MYC;MYCN;NAA40;NASP;NCOA3;NEDD9;NID1;NKIRAS2;NNT;NOTCH2;NREP;NUS1;OTUD4;P3H1;PATZ1;PDGFA;PDGFB;PDGFC;PDGFRA;PDGFRB;PER1;PHACTR2;PIGN;PIGS;PIK3CG;PIK3R1;PLAG1;PPARD;PPIC;PPP1R13B;PPY;PRKAA1;PRY;PRY2;PTEN;PTP4A1;R3HDM4;RAB11FIP1;RAB40C;RACK1;RAET1L;RAX;REL;REST;RHBDD1;RIOK3;RNF138;RPL22;RPS4X;RSL24D1;RTL6;RUNDC3B;S100B;SCAF8;SEC31A;SERPINH1;SFPQ;SGK1;SH3GLB1;SLC16A1;SLC29A2;SLC2A14;SLC30A10;SLC7A5P2;SMARCC1;SNAI3;SNX24;SOX12;SP1;SPARC;SPRTN;STAT3;SURF2;TBX21;TCL1A;TDG;TESPA1;TET1;TET2;TET3;TGFB1;TGFB2;TGFB3;TGIF2;THBS2;TMEM237;TMTC3;TNFAIP3;TNRC18;TPD52L2;TPT1;TRAM2;TRIM72;TUBB2A;UBE2Q1;ULBP2;VEGFA;VHL;WDR26;WWTR1;YAE1D1;YY2;ZBTB34;ZBTB5;ZFP91;ZFPM1;ZNF286A;ZNF850
hsa-miR-199a-5p-478231_mir	-4.650	0.505	A2ML1;ABCC1;ACVR1B;AGTRAP;AKAP17A;APOE;ARHGAP12;ATF6;BECN1;C16ORF58;C1ORF226;C3ORF36;CAV1;CCNL1;CCR7;CD44;CDH1;CDH2;CDK9;CDKN1C;CENPO;CEP120;CHCHD4;CHRFAM7A;CLTC;COL19A1;COX15;CRIPT;CSGALNACT1;CSNK2A1;CTGF;CTSC;DDHD1;DDI2;DDR1;DDX19B;DDX3X;DNAJA4;DRAM1;DYNAP;E2F3;EDN1;ERBB2;ERBB3;ERN1;ETS1;ETS2;EXTL3;EZH2;FZD4;FZD6;GATA6;GM2A;GPR78;GSK3B;HIF1A;HK2;HSPA5;IKBKB;ITGA3;JAG1;JUNB;KL;KRAS;LAX1;LDLR;LIF;LIN7A;MAFB;MAP3K11;MAP3K9;MAP4K3;MECP2;MED6;NAA15;NAB2;NDUFS2;NECTIN1;NFKB1;OSCP1;OXSR1;PANK3;PAX8;PDE11A;PDE4D;PIAS3;PIK3CD;PIN1;PLEKHG2;PLGRKT;PLPP4;PLXND1;PODXL;POLA2;POLR2F;PSAPL1;PSG3;PSG9;PSMD9;PTCD2;PTGS2;QSOX1;RAB21;RCC1;RER1;RIC8A;RND1;RNF11;RNF115;SERPINH1;SESN2;SETD2;SIRT1;SLC16A10;SLC26A2;SLC27A1;SLC38A2;SLC8A1;SMAD3;SMAD4;SMARCA2;SNAI1;SNAP25;SNRNP48;SNTB1;SOX9;SULT1E1;TBC1D21;TFDP2;TGFB2;TGFBR1;TMEM54;TMOD2;TNFRSF13C;TRIM10;TSC22D1;TUBG1;UNG;VASP;VAV3;VEGFA;VPS53;WNK1;WNT2;XRRA1;ZBTB37;ZDHHC9;ZFP1;ZNF117;ZNF195;ZNF215;ZNF286B;ZNF394;ZNF415;ZNF440;ZNF468;ZNF525;ZNF544;ZNF584;ZNF611;ZNF625;ZNF669;ZNF772;ZNF791;ZNF844;ZNF846
hsa-miR-502-3p-478348_mir	2.712	0.509	AGBL5;ASXL2;B2M;B4GALT5;C8ORF33;CA12;CDC27;CDK6;COL23A1;COMMD4;CRK;CRYBG1;CSRP1;CYP4F11;DCTN2;EFCAB11;ELAVL2;EMP2;ENTHD1;FCN2;FRS2;FSCN1;GTPBP2;IBA57;IL21R;KCNJ6;KMO;LINC00632;LINC00955;LRRC58;LYSMD3;MANBAL;MGAT5;MRRF;MSI1;MTHFD2;NAA30;NCAPG2;NLGN4X;NPTXR;OIP5;PEG10;PITRM1;PLCB1;PLEKHB2;PSMG1;RBM3;RPL4;RPS27;RTFDC1;SESN1;SET;SIGLEC15;SLC2A12;SMIM13;SNRPD3;SOCS4;SOD2;SSTR1;STARD7;TBL1XR1;TLCD2;TMEM135;TNIP3;TOMM40L;TRDN;TRIM72;UGT2B10;WDR82P1;WSB1;ZBTB43;ZC3H12C;ZNF460;ZSWIM6
hsa-miR-518e-3p-479408_mir	0.443	0.510	C16ORF58;CADPS2;CSNK2A1;DMGDH;MINOS1;NCKAP5;PGM2L1;TYRO3;VSNL1;ZNF275
hsa-miR-744-5p-478200_mir	3.905	0.526	AARS;ABI3;ABL1;ACTB;ACTG1;ACTN4;ADAP2;ADCY9;ADRM1;AGO1;AGPAT2;AGPAT3;AIMP2;AKT2;ALDH18A1;ANAPC1;ANKRD45;ANKRD52;AP2A2;AP2B1;AP3D1;AP5Z1;ARHGAP5;ARHGDIA;ARHGEF2;ARL15;ARRDC2;ART5;ATF7IP;ATMIN;ATP11A;ATP5B;ATP5G3;ATP5S;ATXN2L;BAG6;BARX1;BCL2L12;BCR;BET1;BRAF;C1D;C1ORF229;C6ORF62;C8ORF33;CACNA1A;CAPN15;CAPNS1;CCDC106;CCDC71;CCNY;CD44;CDAN1;CDC25B;CDK12;CDK16;CEBPA;CGNL1;CHD5;CHERP;CHRAC1;CKB;CLUH;CNN2;CNOT1;COL5A1;COLGALT1;COPA;COTL1;CTDNEP1;CTNNA1;CTNNB1;CTNNBIP1;CXCL16;CYB5R3;CYTH2;DCXR;DDX11;DDX17;DDX23;DDX24;DDX54;DESI1;DHX57;DIDO1;DISC1;DPF1;DUSP18;DUT;DVL1;EDC3;EEF1A2;EIF3K;EIF3M;EIF4A1;EIF4A2;EIF4G1;EN2;ENO1;EPN1;ERP29;ESS2;F2R;FAAP100;FAM110A;FAM171A2;FAM212B;FAM53C;FAM83G;FANCG;FARSB;FASN;FBN2;FBXL19;FGFR1;FKBP4;FNDC3B;FOXO3;FOXP4;FOXRED2;FRAT1;FRZB;FZR1;G3BP1;GAK;GART;GATAD2A;GATAD2B;GDI2;GFER;GGA3;GIPC1;GMDS;GNB2;GPRASP2;GSK3A;GSK3B;GSR;HAAO;HINT1;HIST1H1E;HIST1H2BC;HIST1H2BD;HIST2H2AA3;HM13;HMGA1;HNRNPC;HOXC11;HOXD11;HPCAL4;HRNR;HSPA1B;HSPD1;HTRA2;IER5;IGSF8;ILK;ILKAP;INO80E;INTS3;IP6K1;IRAK1;IRF2BP1;IRGQ;IRS4;JCAD;KCTD11;KHSRP;KLHL15;KMT2D;KPNB1;L3MBTL2;LAIR1;LARP1;LARP6;LDLR;LDLRAD3;LGALS3;LIMS1;LIN37;LIPA;LMNB2;LNPEP;LRP3;LY6E;LYNX1;LYPLA2;MAFK;MAN2C1;MAPK7;MARCH3;MARVELD1;MATR3;MED15;MED28;MELTF;MEX3C;MEX3D;MGRN1;MICAL2;MIEF1;MIF;MINK1;MLF2;MNT;MRPL11;MRPL46;MRPS16;MRPS2;MRS2;MT-ATP6;MT-CO1;MT-CO2;MT-CO3;MT-ND1;MTHFD1;MTOR;MTSS1L;MYC;NACC1;NANOS1;NCEH1;NCKAP5L;NCL;NCOA3;NCS1;NDE1;NDUFB10;NECTIN1;NEUROD2;NFAT5;NFIX;NKD1;NMT1;NOB1;NOL9;NONO;NOP2;NOP9;NOTCH2;NR1D2;NRGN;NUDT2;NUFIP2;NUP153;NXPH4;OGFR;OPRD1;OSBPL5;OTUD4;PA2G4;PABPC1;PAGR1;PAK4;PAX2;PDE4A;PDPK1;PDXK;PELP1;PGGHG;PHC2;PHF8;PIAS4;PIGC;PIKFYVE;PIM3;PIN1;PIP5K1A;PKP2;PLEKHG2;PLEKHG5;PLEKHM2;PNPLA2;POFUT2;POLD3;POLR2A;POLR2L;POMGNT1;PPDPF;PPM1M;PPP1CA;PPTC7;PRDX5;PRKACA;PRR12;PRRC2A;PRX;PSMB2;PSMD11;P

			SMD4;PTGES3;PTGR2;PTMA;PTPMT1;PTPRF;PXK;PYGB;QARS;RAB17;RACK1;RAI1;RAN;RASSF1;RBM8A;RHEB;RNASEK;RNF207;RPL10;RPL13;RPL18;RPL18A;RPL3;RPL37;RPL4;RPL7A;RPLP1;RPS14;RPS6;RPS6KA5;RTL6;SALL1;SAMD4B;SAT1;SBF1;SCAF4;SCAMP2;SEC16A;SECISBP2;SENP2;SEPT2;SERP1;SETD1B;SF3A1;SFN;SFRP1;SGMS1;SH3BGRL3;SH3GL1;SKI;SLC25A1;SLC25A3;SLC25A46;SLC2A4RG;SLC35B4;SLC35D1;SLX4;SMARCD1;SMC1A;SMYD4;SNAPC4;SORBS3;SPECC1L;SRCAP;SRCIN1;SREBF1;SRGAP1;SRM;SRRM2;SRSF1;STAM2;STK16;STMN1;SURF4;SYMPK;SYPL1;TACC3;TAPBP;TBX1;TBX2;TCOF1;TDRKH;TECR;TGFA;TGFB1;THBS2;TIAM1;TJAP1;TLE3;TM9SF3;TMEM131L;TMEM14C;TMEM175;TMEM189;TNKS;TNRC6B;TOB2;TOR2A;TOR4A;TP53I13;TRAPPC12;TRIM28;TRIM33;TRIM59;TRMT2A;TSC2;TSC22D3;TSPAN17;TSPAN7;TSR1;TUB;TUBA1A;TUBA1B;UBA1;UBE2A;UBE2N;UBE2O;UBL5;UBQLN4;UFC1;ULK3;UNC13A;URB1;USP12;USP21;VDAC1;VGLL4;VPS18;VPS37C;VPS8;WBP2;WDR37;WDR45B;WIPF3;YBX1;ZCCHC14;ZCCHC24;ZMYM3;ZNF155;ZNF212;ZNF256;ZNF282;ZNF318;ZNF598;ZNF704;ZSCAN18
hsa-miR-339-5p-478040_mir	-1.358	0.540	AAR2;ABCC1;ACACA;ACAD8;ACOX1;ACTN4;AHCY;AHSA2;AKAP10;AMBRA1;AMOTL1;ANKRD52;ANP32E;APOC3;ARHGAP18;ATIC;ATP1B4;AWAT2;AXL;B4GALT7;BACE1;BAG2;BCL2L11;BCL6;BIVM;BLCAP;BORCS5;BRSK1;BTG2;C11ORF54;C17ORF105;C1GALT1;C7ORF49;CARD8;CBX5;CCNG1;CD3D;CDC6;CENPA;CENPL;CHAF1B;CHDH;CHMP1B;CHMP2A;CHPF2;CHRM3;CLN8;CRIPT;CTBP2;CTSD;CUL3;DCAF16;DHRS13;DLGAP5;DVL1;EBNA1BP2;EEF1A1;EFNB2;ELOVL7;EVC;EXOSC2;FAM110A;FAM118A;FAM208A;FAM219B;FAM241A;FBXO31;FEM1A;FGD4;FHL2;FRRS1;FSIP2;FUNDC2;FUT1;G3BP2;GDE1;GK5;GPATCH3;GPR45;GRIK4;GRK3;GTPBP10;H6PD;HDHD2;HHIP;HLA-E;HNRNPA1;HNRNPA1L2;HNRNPDL;HNRNPF;HOXD10;HSPA12A;HSPA1B;HSPA9;ICOSLG;IGSF9;INPP5A;IPMK;IPO9;IRGQ;KCNH1;KCNJ11;KCTD2;KIAA1143;KIAA1551;KIF6;KLHDC8A;KRTAP12-2;LBX2;LIMA1;LIMD1;LIX1L;LPIN3;MAP1S;MAP3K9;MAP4;MAPKAPK5;MARS;MBD3;MDM2;METTL27;METTL7A;MRPS14;NANOS1;NCBP3;NCSTN;NFIB;NFIC;NHLRC2;NOVA1;NRBP1;NSD2;NUDT4;ORC1;OTUD5;OTUD7B;PAFAH1B1;PAFAH2;PARP1;PAXIP1;PDK3;PGD;PHAX;PKMYT1;PLA2G2C;PLEKHG3;PNPO;POLR2E;PPFIBP1;PPT1;PRDX2;PSAP;PSD4;PTP4A1;PVR;RAB15;RAB32;RAN;RAPGEF1;RAPGEF2;RCAN1;RFC5;RFWD3;RILPL1;RNPEPL1;RPA1;RPS15A;RUNDC1;S1PR2;SEC63;SEL1L3;SIX3;SLC11A1;SLC11A2;SLC12A7;SLC24A4;SLC25A32;SLC25A43;SLC48A1;SLC5A5;SMC1A;SNX33;SOD2;SOX11;SPECC1L;SPI1;SRP19;SSR2;SSX2IP;SUCLG1;SULF2;SULT1B1;SUPT16H;SYNPO;SZT2;TCF15;TCTN3;TFPI;TIAL1;TIMM50;TMED4;TMEM170A;TPM3;TRA2B;TSKU;TTYH3;TUFM;UBXN7;UFM1;UGDH;UQCR10;URGCP;USF2;USP6NL;USP9X;VAPB;WDR13;WDR46;WDR77;XPNPEP3;ZBTB8A;ZCCHC9;ZNF280B;ZNF285;ZNF329;ZNF394;ZNF408;ZNF445;ZNF460;ZNF574;ZNF703;ZNF708;ZNF878;ZNHIT6;ZNRF3
hsa-miR-15b-5p-478313_mir	-2.200	0.540	ABCC6;ABHD2;ABL2;ACOX1;ACTR2;ACTR3B;ACVR2A;ADORA3;ADRA2B;AFDN;AFF4;AGO2;AGO4;AGPAT5;AHNAK2;AIFM2;AIG1;AKAP11;AKR1B10;AKT3;ALDH3B1;AMER1;AMMECR1L;AMOT;AMOTL1;ANAPC13;ANKMY1;ANKRD13B;ANKRD36;AP2B1;AP2M1;AP3M1;AP4S1;AP5S1;AP5Z1;APP;APRT;ARCN1;ARHGAP12;ARHGAP32;ARHGDIA;ARID2;ARIH1;ARMC12;ASCC1;ASGR2;ASH1L;ASXL1;ATAD5;ATG14;ATG9A;ATP13A3;ATP2A2;ATP5G3;ATP6V0E1;ATXN7L3B;AURKAIP1;AVL9;AXIN2;B3GNT2;B4GALT1;BAG4;BAMBI;BAX;BAZ2A;BCL2;BCL2L12;BCL7A;BEX1;BHLHE40;BRAT1;BSG;BSPRY;BTAF1;BTG2;BTN3A3;BTRC;BZW1;C11ORF24;C15ORF39;C16ORF58;C16ORF72;C1ORF21;C1ORF226;C1ORF43;C21ORF62;C2ORF42;C3ORF36;C5ORF15;C6ORF106;CA8;CACUL1;CALU;CAMSAP1;CANX;CAPZA2;CARD10;CARM1;CASK;CASKIN1;CBFA2T3;CBX2;CBX4;CBX6;CCDC80;CCDC83;CCDC88C;CCND1;CCND2;CCND3;CCNE1;CCNE2;CCNT1;CCNT2;CD180;CD274;CD2AP;CD44;CDADC1;CDC25A;CDC27;CDC37L1;CDC42SE2;CDCA4;CDK1;CDK17;CDK4;CDK6;CDKN1A;CDKN2AIPNL;CDS2;CDV3;CEP55;CHAC1;CHEK1;CHIC1;CHMP3;CHMP4B;CHMP7;CHPF;CKAP5;CLCN6;CLEC2D;CLIP4;CLSPN;CLU;CLUH;CMTM4;CNKSR3;COIL;CPEB2;CPEB3;CPNE1;CPOX;CPSF7;CREBL2;CREBRF;CREG1;CRIM1;CRK;CRKL;CSDE1;CSNK1E;CTDSPL;CUL2;CUL3;CYB561A3;CYBA;CYLD;CYP26B1;CYP51A1;DCAF17;DCTN5;DDX3X;DDX3Y;DDX5;DECR1;DENND6A;DENR;DHFR;DHX30;DHX37;DIAPH1;DICER1;DLGAP3;DMPK;DMRT2;DMTF1;DNAJA1;DNAJC10;DNAJC5;DNAJC9;DOCK11;DPP8;DSCR3;DSG2;DYNLL2;DYRK3;E2F3;E2F7;ECHS1;EDC3;EDC4;EEF1A1;EFNB2;EFTUD2;EIF1AX;EIF2AK2;EIF2B2;EIF4A1;EIF5B;ELK4;EN2;ENTPD1;ENTPD6;ENTPD7;EPM2AIP1;ETFRF1;ETNK1;EXT1;EZH1;FADD;FAM103A1;FAM122B;FAM214A;FAM229B;FANCC;FASN;FAXC;FBXL18;FBXL20;FBXO30;FCF1;FGF2;FGF5;FGFR4;FKBP1A;FLCN;FLOT2;FOXK1;FOXO1;FRYL;FURIN;FUT2;FZD6;FZD9;GABARAP;GABARAPL1;GABPA;GALNT1;GANAB;GATAD2A;GGA3;GLP2R;GNAL;GNAT1;GNB1;GNG12;GNL3L;GOSR1;GPATCH8;GPR180;GPR27;GPRC5A;GRAMD2B;GRB2;GSG1;GSK3B;HAUS3;HCFC2;HDGF;HEYL;HIGD1A;HIST2H2BE;HIST2H3A;HMBOX1;HMGA1;HNF1A;HNRNPA1;HNRNPA1L2;HNRNPA2B1;HNRNPDL;HNRNPH3;HNRNPK;HNRNPM;HOXA10;HOXA3;HOXC8;HSP90AB1;HSPA1B;HSPA4L;HSPA8;HSPE1-MOB4;HYOU1;IER2;IFIH1;IFNG;IFNGR2;IFT74;INSL6;INSR;IPPK;IRAK1BP1;IRF4;IT

			GA2;ITGA6;ITPR1;IVNS1ABP;JAK1;JARID2;JPT2;KANK1;KATNAL1;KDR;KIAA0895;KIAA1456;KIF20B;KIF23;KIF3B;KIF5B;KLC2;KLHDC10;KLHL15;KLHL40;KMT2C;KMT2D;KPNA1;KPNA3;KRT33B;L2HGDH;LAMC1;LAMP2;LANCL1;LIN28B;LITAF;LRIF1;LRIG2;LRPPRC;LRRC41;LRRC57;LRRFIP2;LSM11;LSM14A;LUC7L3;LURAP1L;LUZP1;LZTR1;MAFK;MAP2K3;MAP2K4;MAP3K7;MAP4K2;MAPK1;MAPKAPK2;MARCKS;MBD4;MCFD2;MCM3AP-AS1;MCPH1;MDH1;MED11;MESD;MIB1;MIGA1;MINK1;MKX;MLLT6;MLXIP;MMP9;MOB4;MPHOSPH9;MRPL40;MRPS11;MSANTD4;MSL1;MTFR1L;MTHFR;MTMR3;MTMR4;MTMR6;MTSS1;MYCBP2;MYO5A;N4BP1;NAA25;NAA30;NAB1;NAPG;NCKAP1;NCOR2;NDFIP1;NEGR1;NFIC;NNT;NOL4L;NOTCH2;NPM1;NR2C2;NR6A1;NRIP1;NUCKS1;NUFIP2;NUP160;NUP210;NUP214;NUP50;OCRL;ODF2L;OGT;OIP5;ORC4;OSBPL3;OSCAR;OTUB1;PAFAH1B1;PAFAH1B2;PAG1;PAGR1;PAK2;PANK1;PATJ;PCMT1;PDCD1;PDCD4;PDE4D;PDIA6;PDIK1L;PEBP4;PEX12;PEX13;PGD;PHC3;PHF19;PHKA1;PHLDB3;PHLPP2;PHYHIP;PI4K2B;PIK3C2B;PIK3R1;PIM1;PISD;PLAG1;PLEKHA1;PLEKHB2;PLPBP;PLPP3;PLRG1;PLSCR4;PNISR;PNPLA6;PNPO;PNRC2;POLDIP3;POLE4;POLR2E;POM121C;POU2AF1;POU2F1;PPIG;PPIL1;PPIL2;PPIP5K2;PPM1A;PPM1D;PPP1CB;PPP1CC;PPP1R11;PPP2R5C;PPP6C;PPP6R3;PPT1;PPTC7;PRDM4;PRDX3;PRICKLE2;PRKAA1;PRKAR2A;PRKCD;PRPS1;PRRC2C;PRSS21;PSAT1;PSKH1;PSMB5;PSMD7;PTAR1;PTBP1;PTPRD;PTPRJ;PURA;PXMP2;QARS;QRICH1;RAB11FIP2;RAB15;RAB1A;RAB1B;RAB23;RAB3IP;RAB40B;RACGAP1;RAD23B;RAE1;RALGAPB;RANBP10;RAP2C;RAPH1;RARB;RASEF;RASSF2;RASSF5;RBBP6;RBM27;RBPJ;RCAN3;RCOR1;RECK;REL;RELT;REXO1;RFK;RFWD2;RHOV;RIMS3;RLIM;RNASEH1P1;RNF138;RNF149;RNF168;RNF38;RNMT;RNPS1;RPL14;RPL27A;RPL36;RPLP0;RPRD1B;RPRD2;RPS3;RPS3A;RPS5;RPS6;RPS6KA3;RPS6KB1;RS1;RTN4;RUBCN;RUNX1T1;SALL1;SBNO1;SCAMP4;SCAMP5;SCCPDH;SEC24A;SEC61A1;SELENOI;SEPT2;SERBP1;SESTD1;SETD1B;SF3B3;SFT2D1;SH3BP4;SHOC2;SIDT2;SIK1;SIRT4;SKI;SLC1A5;SLC25A12;SLC25A22;SLC25A29;SLC29A1;SLC2A3;SLC35E2B;SLC39A14;SLC39A9;SLC7A5;SLC9A1;SLC9A6;SLCO3A1;SMAD2;SMAD3;SMAD7;SMDT1;SMURF1;SNCG;SNRPB2;SNTB2;SNX11;SNX16;SOCS3;SOCS5;SOWAHC;SPRED1;SPTLC1;SREK1;SRPK1;SRPRA;SRPRB;SRSF1;SSRP1;SSU72;STK38;STRADB;STT3B;STX17;STXBP1;STXBP3;SUN1;SUPT16H;SUPT3H;SYNJ1;SYNRG;SYPL1;SZRD1;TAB2;TADA2B;TAF13;TANGO6;TAOK1;TARBP2;TARDBP;TASP1;TBC1D14;TBC1D20;TBCCD1;TBL1XR1;TBPL1;TBR1;TBRG1;TBRG4;TCAF2;TCF3;TCP1;TECPR2;TERF2;TET3;TFAP2A;TFB1M;TGFB1;TGFBR3;TGOLN2;THRA;THRAP3;TIMM13;TKTL1;TLE4;TLK1;TLL1;TM4SF1;TM7SF3;TM9SF2;TM9SF3;TMC7;TMEM100;TMEM109;TMEM135;TMEM138;TMEM161B;TMEM189;TMEM189-UBE2V1;TMEM245;TMEM43;TMEM63B;TMEM69;TNFSF9;TNPO3;TNRC6B;TOB2;TPD52;TPM2;TPM3;TRAK1;TRAM1;TRAPPC1;TRIM14;TRIM29;TRIM35;TSC22D2;TSPAN3;TTC1;TTLL5;TUBB;TUBB2A;TXLNA;TXNIP;U2SURP;UACA;UBE2H;UBE2Q1;UBE2Q2;UBE2V1;UBE3B;UBE3C;UBE4A;UBN2;UBR3;UGT2B4;URB2;USP15;USP3;USP31;USP42;USP48;USP53;UVSSA;VAV2;VCL;VDAC2;VEGFA;VOPP1;VPS13A;VPS33B;VPS35;VPS4A;VSIR;WAC;WDR13;WEE1;WIPI2;WNK3;XKR7;XKR9;YIPF6;YRDC;YTHDC1;YWHAH;YWHAQ;ZBTB10;ZBTB16;ZBTB33;ZBTB34;ZBTB5;ZCCHC14;ZCCHC3;ZDHHC16;ZFAND5;ZFHX4;ZFP28;ZMAT3;ZNF217;ZNF267;ZNF275;ZNF284;ZNF367;ZNF391;ZNF449;ZNF460;ZNF585B;ZNF620;ZNF622;ZNF691;ZNF704;ZNF91;ZNRF1;ZNRF2;ZNRF3
hsa-miR-127-3p-477889_mir	-0.714	0.542	ACTR3B;ACTR3C;BAG5;BCL6;BOLA1;COTL1;DAGLA;GRK2;KCNA6;KMT5A;LGALS8;MAPK4;MGMT;MMP13;NBEA;PIK3CG;PRDM1;RGMA;SEC31A;SEPT7;SERPINB9;SFRP1;SKI;SLC29A1;TGM2;USP35;XBP1;XRCC3;ZWINT
hsa-miR-423-3p-478327_mir	-1.679	0.544	ABHD12;ADCY1;AGO1;AKIRIN2;AKT2;ALAS1;AMMECR1L;AP2M1;APP;APPL1;ARFGAP1;ARHGEF2;ATP6V0D1;ATP8B2;ATXN7;B4GALNT3;BAZ1B;BCL2L11;C1ORF56;C22ORF42;C6ORF106;C6ORF141;CALR;CCDC32;CCNT2;CDK7;CDKN1A;CENPM;CEP78;CHCHD3;CIP2A;CNOT1;COMMD9;COPA;CRK;CYFIP2;DDX46;DDX56;DGCR6L;DPYSL3;EAF1;EEF1A1;EIF3B;EIF3C;EIF4B;EIF5A;ESRRA;ETF1;EWSR1;F2;FAAP100;FADS2;FASN;FBRSL1;FBXL19;FEM1A;FLII;FLOT2;FOXJ2;FRMD4A;GCN1;GLDC;GPS1;H1F0;HDLBP;HGS;HIST1H3D;HLA-C;HNRNPA0;HNRNPUL1;HOXD13;HSPA1B;IER3IP1;IGF2R;INCENP;IRAK1;IRGQ;KDELR1;KDSR;KIAA0319L;KIAA1671;KIDINS220;KIF1A;KIF2C;KLHL15;LARP1B;LARP4;LPAR2;LRIG2;LRR1;LRRC8B;LSM4;LURAP1;LYPD1;LYPLA2;MAP3K1;MAP3K9;MAPK3;MCM2;MCMBP;METTL16;MFSD14A;MIB2;MICALL1;MOGS;MRFAP1;MRPL12;MRPS18A;MTA2;MTHFR;MYB;NAA10;NACC2;NARF;NAT14;NDE1;NOTO;NPEPPS;NPTX2;NSD2;NT5DC2;NTN1;NUBP1;NUDT2;NUP210;NUP58;PA2G4;PABPC1;PABPC3;PAPD7;PDCL;PELP1;PEPD;PFKL;PKM;PML;POLR2L;POLR3H;POM121;POTEG;POTEM;PPAN;PPP2R1B;PSMB5;PSMD1;PSMD8;PTCD2;PTMA;PTMS;PTPRK;PUM1;PUS3;QPRT;RAI14;RANBP2;RAP2C;RBBP4;RBM15B;RBSN;REEP2;RNF185;RNF213;ROBO1;RPH3AL;RPL26;RPL3;RPL4;RPLP1;RPP25L;RPS2;RRP1;RTCB;RXRA;SAC3D1;SCML2;SELENON;SET;SETD5;SF3B1;SF3B3;SF3B4;SFMBT2;SH2B1;SHMT2;SLC11A2;SLC25A15;SLC25A6;SLC30A6;SMARCA2;SMARCD2;SMC1A;SPEN;SPTBN1;SRPRA;SRSF1;STOML2;SUCLG1;SUZ12;SZT2;TAGLN2;TATDN2;TBC1D15;TBC1D22B;TBCE;TCEAL1;TLE1;TMEM9B;TNPO3;TRPM2;TXLNA;UBE2J2;UBE4B;UPF1;UQC

			RB;URB2;USMG5;VEGFA;WIZ;WNK1;XRCC6;YIPF5;YWHAQ;ZC3H4;ZFPM1;ZFYVE27;ZNF324;ZNF420;ZNF503;ZNF561
hsa-miR-194-5p-477956_mir	-1.369	0.569	AASDHPPT;ACBD7;ACVR2B;AGO1;AQP6;ARID2;ARRDC2;ATP6V1F;BCLAF1;BICD2;BMI1;BMP1;BMT2;BNIP2;BTF3L4;C11ORF54;C8ORF17;CAPZA1;CARD8;CAV1;CBY3;CD274;CDH2;CDKN1B;CELSR1;CHAC1;CHD1;COL4A3BP;DNAL1;DNMT3A;DUSP18;EIF4G2;EIF4H;ELF1;EP300;EPC2;FEM1A;FOXM1;FXR1;FZD6;GIGYF1;GIPC1;HBEGF;HIST2H3A;HMGA2;HNF4G;HNRNPA1;IGF1R;IL10;ITGA9;ITSN1;KDM5B;KIAA1210;KMT5A;KPNA1;NOTCH2NL;NUDT7;ONECUT3;OTUD3;PEX26;PTBP1;PTPN12;PTPN13;RAB11FIP1;RAC1;RBMS2;RBX1;RDH11;SETD5;SLAMF1;SLC16A9;SLC25A32;SLC6A5;SOCS2;SOX5;SPRED1;SPRY4;SPTY2D1;SRCAP;TJAP1;TJP1;TLN2;TMED5;TMEM120B;TMEM242;TRUB1;TWISTNB;TYMS;UBL3;UHMK1;ZC3H12B;ZNF280B;ZNF724
hsa-miR-31-5p-478015_mir	2.230	0.596	ABCB9;ACBD7;AFF1;AGO1;AKAP8L;AKNA;AKR1B1;ANKRD52;AP2B1;ARF1;ARID1A;ARPC5;ATP2A2;ATP5A1;BAHD1;BAP1;BCAS4;C15ORF52;C17ORF99;C19ORF12;C1QTNF9;C2CD5;CASKIN2;CASR;CCDC127;CCNT1;CDC42SE1;CDK1;CKAP2L;CNBP;CREG1;CXCL12;CYP27B1;DACT3;DDX19A;DKK1;DMD;DNAAF5;DNAJC5;DOCK1;DPM2;DPYSL5;E2F2;ECHDC1;EDC3;EFNB1;EMSY;ETS1;EXOC6;EXOSC5;FAM193A;FLNA;FOXC1;FOXD4;FOXD4L1;FOXD4L4;FOXD4L5;FOXJ3;FOXO3;FOXP3;FRK;FZD1;FZD3;GHITM;GIGYF1;GLI2;GNA13;GTF2E1;GUF1;GYG1;HIF1AN;HIST1H2BC;HIST1H2BJ;HIST1H2BK;HOXA7;HOXC13;HOXD3;ICAM1;IL25;IL5RA;ILF2;INTU;IPP;ITGA5;JARID2;JAZF1;KLF13;KLHDC10;KLHL15;KMT2B;LAPTM4A;LATS2;LILRA2;LIPG;LRRC59;MAGEA12;MAGEA3;MAGEA6;MAP4K4;MCM2;MCMBP;MED12;MET;MICA;MLH1;MMP16;MPRIP;MTSS1L;MXRA7;MYO1D;MZT1;NDFIP2;NF2;NFAT5;NFATC2IP;NFE2L1;NFIC;NOL9;NOP56;NPM1;NUDT3;NUMB;NUP188;OTUD4;PAPOLG;PARP1;PARP11;PCSK1N;PEX19;PHF12;PHLDA1;PLAGL2;PPIL2;PPM1L;PPP2R2A;PPP4R3B;PRKCE;PRRC2B;PTPRJ;PYURF;QSER1;RAB1B;RAB27A;RAB5B;RANGAP1;RASA1;RASA4;RDX;RET;RETREG3;REXO2;RHOA;RHOBTB1;RNF111;RPA1;RPL12;RPL27A;RPL35A;RPL37A;RPL7L1;RPS4Y1;RPS7;RSRC1;SATB2;SDC4;SELE;SERTAD2;SESN2;SFN;SFT2D2;SFXN1;SGPP2;SLC18B1;SLC1A2;SMAD4;SMG1;SNRNP27;SOX4;SP1;SP7;SPRED1;SPRED2;SPRTN;SPRY1;SPRY3;SPRY4;SRC;SRPX2;SRRM2;STK40;STMN1;STOML2;SYDE2;TAPBP;TBXA2R;TIAM1;TMEM109;TMEM182;TMEM9;TNRC6B;TOR1AIP1;TRIB3;TRRAP;TSPAN1;TXNDC5;UBA6;VPS26B;WASF3;XPO6;XRCC5;YWHAE;YY1;ZBTB39;ZC3H12C;ZC3H18;ZDHHC6;ZIK1;ZNF275;ZNF331;ZNF460;ZNF587;ZNF641;ZNF678;ZNF805
hsa-miR-374b-5p-478389_mir	-0.518	0.597	ABCF1;ABCG2;ABHD18;ACSL4;ACSM2A;ACSM2B;ACVR2B;ADD2;ADD3;AFF4;AFTPH;AKT1;ANGPTL3;ANKFY1;APOL6;ARHGAP6;ATF2;ATP6V1C1;ATXN1;AVPR1A;BACH2;BAMBI;BOLA3;BTBD3;C1ORF56;C8ORF33;C9ORF170;CA13;CA8;CAMSAP2;CCDC80;CCND1;CCNI;CDKN2B;CENPK;CMPK1;CNTNAP5;CXCL5;CYP4F11;CYSTM1;DDX52;DDX55;DEK;DLC1;DSN1;DUSP8;EFCAB14;EMC7;EPDR1;ETNK1;FAM126A;FAM229B;FAM76A;FAT3;FKBP1A;FOXC1;FXN;FYTTD1;FZD5;FZD6;FZD8;GABARAPL1;GABRG2;GALNT7;GHITM;GIMAP4;GNL3;GPR158;GSK3B;GSKIP;HHIP;HIST1H3B;HOXA11;HOXA5;HSBP1;HSPA13;IRS2;KBTBD8;KCNC4;KIAA1468;KLHL15;KLHL9;KLRC3;KYAT3;L1CAM;LAMP3;LAMTOR3;LHFPL2;LIN28B;LMAN2;LNPK;LRIG3;LUZP2;MAP3K8;MAPK1IP1L;MAPK7;MB21D2;MBNL2;MEX3C;MFAP5;MIER3;MINPP1;MLLT11;MLX;MPP5;MRPL18;MRPL19;MYBPC1;MYLIP;NAA16;NBPF11;NELL2;NKRF;NLN;NNT;NPLOC4;NPM3;NR2C2;NR3C1;NRXN3;NUFIP2;NUP153;NUS1;OCRL;OSTM1;PANK3;PAPD4;PARD6B;PARP15;PDE12;PELI1;PGS1;PHACTR2;PIGW;PITPNC1;PITX2;PLEKHA2;PMEPA1;POLR1B;PRDX3;PRKCD;PRKX;PTMA;RAB32;RAB8B;RAC1;RANBP6;RAP1B;RAPH1;RASSF5;RBMXL1;RC3H1;RECK;REEP3;RHOU;RORA;RPL24;RRS1;SCAF4;SDHD;SEC23B;SEMA3C;SESN3;SESTD1;SFT2D2;SHISA9;SHOC2;SIK3;SIM1;SLC11A2;SLC16A1;SLC25A24;SLC25A27;SLC38A1;SLC9A4;SNRNP27;SNRPA1;SOCS6;SON;SP1;SPC25;SPTLC1;SRSF7;STAU1;STRAP;STX16;STXBP2;SVIP;SYP;TACC1;TAF7;TFDP1;TMEM241;TMEM245;TMTC1;TNFSF9;TNRC6A;TOR2A;TPR;TPT1;TRA2A;TRIM2;TRIM35;TRMT112;TRPS1;TRUB1;TTC8;UBE3A;UST;VEGFA;VGLL2;WNT16;WWTR1;XKR4;XKR9;YEATS4;YOD1;YTHDF1;ZBED3;ZBTB41;ZBTB7A;ZCCHC2;ZCCHC9;ZDHHC5;ZER1;ZFP37;ZNF148;ZNF175;ZNF383;ZNF443;ZNF566;ZNF681;ZNF99;ZSWIM6
hsa-miR-26a-5p-477995_mir	0.398	0.616	ABCA1;ABCB7;ABCB9;ABHD2;ACBD5;ACSL3;ACTL8;ACTN4;ACVR1;ADAM17;ADGRG7;ADM;ADM2;AGO1;AGPAT5;AGTPBP1;AHR;AHRR;AKIRIN1;ALDH6A1;ALG1;ALG10B;AMACR;ANKRD52;ANO3;AP1S3;AQR;ARCN1;ARF1;ARHGEF1;ARHGEF39;ARL4C;ARNTL2;ARPP19;ASCC2;ASXL3;ATM;ATP1A1;ATP2B1;ATP5A1;ATP7B;AURKAIP1;B3GALT5;BAG4;BBX;BCL7B;BID;BLOC1S2;BMP2K;BMPR1A;BRCA1;BTBD3;BTBD9;C14ORF37;C16ORF70;C17ORF51;C1ORF50;C3;C6ORF47;C7ORF55-LUC7L2;CA2;CAMKV;CAPZA1;CCDC43;CCND2;CCNE1;CCNE2;CD55;CDC6;CDK6;CDK8;CDK9;CDV3;CEPT1;CES2;CFLAR;CHAC1;CHAF1A;CHD1;CHEK1;CHEK2;CHORDC1;CKS2;CLIC4;CNBP;CNTD2;COASY;COPB1;COX5A;CPEB2;CPEB3;CPEB4;CPSF2;CREBRF;CREBZF;CSNK1A1;CSTF2;CTBP1;CTC1;CTGF;CTNS;CTR9;DCAF10;DCAF7;DCP1A;DCTN4;DDB1;DDIT4;DDR2;DDX3X;DEPDC1;DHX15;DIP2A;DNMBP;DNMT1;DNMT3B;DST;DUSP4;DUSP5;DYRK1A;E2F2;E2F7;EDEM3;EEF1B2;EFCAB14;EHD1;EID2B;EIF4G2;EIF5;ELAVL2;ELOVL6;EP300;EP400;EPB41L3;EPG5;ESR1;E

			TF1;EZH2;FAM126B;FAM177A1;FAM20B;FASN;FAXC;FBXO11;FER;FGF9;FKTN;FOXO3;FPR1;FRAT2;FUT8;GALNT3;GDAP1;GGA2;GIT2;GLG1;GLTP;GPALPP1;GRK2;GSK3B;GTF2A1;HAUS8;HECTD3;HGF;HIST1H4E;HIST1H4J;HIST2H2AA3;HIST4H4;HMGA1;HMGA2;HNRNPA0;HNRNPU;HOXA5;HOXC8;HPSE;HSP90B2P;HSPA13;HSPA8;HSPD1;IFNB1;IGF1;IL6;INSL3;IRF4;ITGA2;ITGA5;JAG1;KAT14;KCTD5;KDELR1;KDM5C;KIAA0408;KIF3A;KLHDC10;KLHL15;KLHL42;KMT5A;KPNA2;KPNA6;LARP1;LIF;LIN28B;LINS1;LOXL2;LRP6;LRRC27;LRRTM4;LSM14A;LUC7L2;LYST;MAD2L1;MALT1;MAN2A1;MAP1B;MAP3K2;MAPK6;MAT2A;MCL1;MCM3AP;MCM8;MDM2;METAP2;METTL2B;MFHAS1;MFSD14A;MICA;MINDY1;MRPL51;MSH6;MSL3;MT-CO1;MT-CO3;MTA3;MTDH;MTMR12;MTRF1;MTRF1L;MYC;MYH10;MYO10;MYOZ3;NABP1;NAMPT;NANOS2;NIP7;NKX2-5;NRAS;NRP1;NSA2;NUAK1;NUCKS1;NUDT19;NUFIP2;NUP205;NUPL2;NUS1;NWD1;OCIAD2;OGT;OLA1;ORC6;OTUD1;OTUD4;PCK1;PCYT1A;PDCD10;PDE12;PDE4B;PDE4C;PDE4DIP;PHAX;PHB;PHLDA1;PIGT;PIK3C2A;PIK3CG;PIK3R4;PIKFYVE;PIM1;PLAC8;PLAG1;PLOD2;PMAIP1;PNMA2;PNPLA3;PNRC1;POLR2E;POLR3G;PPIA;PPP1CC;PPP1R15B;PPP2R5D;PPP4R3A;PRDX3;PRKAA1;PRKCD;PRKX;PSAT1;PSMA3;PTEN;PTGS2;PTPN13;PTTG1;RAB18;RABGAP1L;RABL3;RASA1;RASGRP3;RB1;RBM39;RBM48;RCBTB1;RCC1L;RCOR1;REEP4;RETREG2;RGS17;RHCG;RHOBTB1;RHOQ;RND2;RNF11;RNF216;RNF6;RPL13;RPL13A;RPL37;RPS24;RPS26;RPS27A;RRM2;RRP36;RTF1;SACS;SARS;SASS6;SCRN1;SDCBP;SDE2;SEC31A;SEPT14;SERBP1;SERP1;SET;SETD2;SFMBT1;SFPQ;SFXN1;SHC2;SLC25A30;SLC25A36;SLC25A5;SLC26A2;SLC31A1;SLC35A4;SLC35B3;SLC35B4;SLC38A2;SLC5A3;SLC7A1;SLFN13;SMAD1;SMAD4;SMIM9;SOGA3;SPTBN1;SSBP2;SSH2;SSR1;SSR3;SSX2IP;ST3GAL6;ST8SIA4;STRADB;TAF13;TBC1D13;TBC1D16;TCP1;TDG;TET2;TET3;TFAM;TIMM29;TMCC1;TMCC3;TMEM154;TMEM19;TMEM2;TMEM254;TMEM30A;TMTC3;TNFAIP8L1;TNFSF10;TNRC6A;TNRC6B;TOB1;TP53INP1;TPGS2;TPMT;TRPC6;TSC22D2;TSHZ1;TTK;TTLL12;TTN;TUB;TUBG1;TUBGCP5;TUT1;TXLNG;TXNDC11;TXNL1;TYW1;UBA2;UBE2A;UBE2H;UBN2;UBR3;UBR4;UBTF;UGGT1;UQCR11;USB1;USP3;USP7;VANGL2;WDR92;WEE1;WNK1;WNT5A;WWP2;YWHAE;ZBTB18;ZBTB24;ZBTB45;ZCCHC11;ZDHHC18;ZKSCAN1;ZNF134;ZNF207;ZNF233;ZNF254;ZNF347;ZNF410;ZNF426;ZNF451;ZNF506;ZNF566;ZNF608;ZNF664;ZNF669;ZNF680;ZNF703;ZNF772;ZNF814;ZNF860;ZSWIM6
hsa-miR-653-5p-479134_mir	-0.585	0.617	AASS;ADPRHL1;AK2;ALDOA;ANKRD6;AQP3;ATG10;ATP5G3;BACE1;BAG4;BBX;C1GALT1C1;C9ORF40;CALM2;CC2D1B;CHCHD4;CLIP1;DCAF5;DCBLD2;DOK6;DZIP1L;EIF1AX;ELMOD2;ETF1;FBXL18;FLCN;GJB7;GLUD1;GLUD2;GNAQ;HOXA10;HOXD8;IGFBP5;IL6R;KLF6;LUZP2;LVRN;MAPKAPK5;MECP2;MOB1B;MRPL22;MTDH;MTRNR2L10;MTRNR2L8;NR5A2;PAPOLG;PAWR;PAX6;PCNP;RABAC1;RBM25;RDH10;REPS1;RNF38;RPS27A;SEM1;SEPHS1;SERINC3;SERP1;SH3BP4;SMAD9;STAT1;STYK1;TBX18;TFPI;TPBG;TRIM56;TSN;TUB;UBC;UBE2D1;UBE2V2;YIPF4;YWHAE;ZNF813;ZSCAN12
hsa-miR-93-5p-478210_mir	-0.855	0.672	A1CF;AAK1;ABCA1;ABCG8;ABHD15;ABHD18;ABHD2;ABI2;ACADSB;ACAP2;ACBD5;ACER2;ACIN1;ACOT9;ACOX1;ACSL4;ACTR2;ACVR1B;ADAR;ADARB1;ADAT2;ADD1;AFF1;AGFG2;AGMAT;AGO1;AGO3;AKAP11;AKR7A2;AKTIP;ALDH9A1;ANAPC5;ANG;ANKFY1;ANKH;ANKIB1;ANKRD12;ANKRD13C;ANKRD29;ANKRD33B;ANKRD50;ANKRD52;ANKS4B;AP1AR;AP1G1;AP2A2;APBB2;APLP2;APOH;ARAP2;ARCN1;ARHGAP1;ARHGAP12;ARHGAP32;ARHGAP35;ARHGEF18;ARHGEF7;ARID4B;ARIH2;ARL1;ARL9;ARMT1;ARPC2;ARSJ;ASB1;ASB16;ASF1A;ATAD2;ATAT1;ATG14;ATG16L1;ATG2A;ATG2B;ATL3;ATP1B3;ATP2B1;ATP5B;ATP6V0E1;ATP6V1G2-DDX39B;ATXN1;ATXN7L3B;B2M;BAG2;BAGE5;BAZ2A;BBX;BCAS4;BCL2L11;BCL2L2;BHMT2;BICD2;BIRC5;BLOC1S3;BMP2;BMP8B;BMPR2;BMT2;BNIP2;BRI3BP;BRMS1L;BSCL2;BTBD10;BTBD7;BTF3L4;BTG2;BTG3;BTN3A1;BTN3A2;BTN3A3;BZW1;C11ORF54;C12ORF65;C14ORF119;C14ORF28;C15ORF40;C15ORF41;C16ORF52;C16ORF70;C17ORF75;C18ORF32;C1ORF50;C2ORF69;C3ORF38;C5ORF22;C6ORF120;C6ORF62;C7ORF43;C9ORF40;CABLES1;CADM2;CALCOCO2;CAMK2N2;CAMTA1;CAP1;CAPN15;CAPRIN2;CAPZA2;CASP2;CAV1;CAV2;CAVIN1;CBX1;CBX3;CBX5;CBX8;CCDC125;CCDC137;CCDC198;CCDC30;CCDC47;CCDC6;CCDC71L;CCDC88C;CCL1;CCL5;CCND1;CCNG2;CCP110;CCSER2;CCT8;CD164;CD28;CD46;CD47;CDC16;CDC37L1;CDHR1;CDIPT;CDKN1A;CDKN2AIPNL;CENPQ;CEP104;CEP170;CEP57;CEP72;CEP97;CERCAM;CERS2;CFL2;CHAF1A;CHD9;CHIC1;CHSY1;CHURC1;CIT;CKAP2;CLEC12B;CLIC4;CLIP4;CLN8;CLOCK;CMPK1;CMTR2;CNKSR3;CNOT4;CNOT6L;CNOT7;CNTN1;COIL;COQ2;COX19;COX6B1;CPOX;CPPED1;CPS1;CPT1A;CRCP;CREB1;CREBRF;CRELD2;CRISPLD2;CRK;CROT;CRTC3;CRY2;CSDE1;CSNK1A1;CSNK1G1;CTC1;CTSS;CXCL8;CXORF38;CYB5A;CYBRD1;CYCS;CYCSP5;CYLD;DAB2;DAPK3;DCAF8;DCBLD2;DCTN1;DCTN4;DCTN5;DCTN6;DDHD1;DDI2;DDX39B;DDX46;DDX5;DEGS1;DENND5B;DHODH;DIS3L;DMAC2;DMKN;DNAJB13;DNAJB4;DNAJB6;DNAJB9;DNAJC10;DNAJC27;DNAJC28;DNAL1;DNM1L;DNMT3B;DNTTIP2;DPP8;DPP9;DPYSL2;DRAXIN;DSPP;DSTYK;DUSP18;DUSP2;DUSP8;DVL3;DYNC1LI2;DYRK2;DZIP3;E2F1;E2F2;E2F3;E2F5;EAPP;EEA1;EEF1A1;EFCAB11;EFCAB14;EGLN3;EGR2;EIF2B2;EIF2S1;EIF4A2;EIF4G2;EIF4H;EIF5A2;ELAC2;ELAVL1;ELAVL2;ELK4;ELMS

			AN1;ELOC;EMC1;EMSY;ENPP5;ENTPD4;ENTPD7;EPHA4;EPHA7;EPS15L1;ERAP1;EREG;ERGIC2;ERLIN2;ESR2;ETF1;EXO5;EZH1;EZH2;F2R;F2RL1;F2RL3;FAAP24;FAF2;FAH;FAHD1;FAM102A;FAM117B;FAM126B;FAM129A;FAM160B1;FAM210A;FAM213A;FAM241A;FAM3C;FAM46C;FAM57A;FAM83D;FAM89A;FASN;FAXC;FBXL3;FBXL5;FBXL7;FBXO10;FBXO21;FBXO31;FBXO48;FCHO2;FEM1A;FEM1B;FEM1C;FEZ2;FGFR1OP;FHDC1;FICD;FIGNL1;FJX1;FKBP14;FKBP7;FMNL2;FMNL3;FNBP1L;FOXA1;FOXC1;FOXJ2;FOXJ3;FOXK1;FOXK2;FOXO3;FOXQ1;FOXRED2;FRMD6;FRS2;FUT10;FXYD5;FYCO1;FZD9;GAB1;GABBR1;GABPAP;GAK;GAPDH;GARS;GATA6;GATAD1;GBF1;GBP3;GDF11;GDF5OS;GEMIN8;GID4;GIGYF1;GINS4;GLI3;GLO1;GNA12;GNAS;GNB4;GNB5;GNPTAB;GNS;GOLGA1;GOLGA2;GPAM;GPATCH2;GPN3;GPR137B;GPR137C;GPR155;GPR157;GPR183;GPRIN3;GPS1;GRAMD1A;GRB2;GRID2IP;GRK3;GRK7;GRPEL2;GRSF1;GTF2H2C;GTF2H3;GTF2IRD2;GTF2IRD2B;H1F0;HARS;HAS2;HAUS8;HBP1;HCCS;HDDC2;HDGF;HECA;HEG1;HERPUD1;HIF1A;HIF1AN;HIP1;HIST1H2BD;HIST1H2BG;HIST1H2BJ;HIST1H3B;HIST2H3A;HIST2H4B;HIST3H2A;HK1;HLA-F;HMBOX1;HMGB1;HMGB2;HMGB3;HNRNPM;HNRNPR;HOOK1;HOOK3;HOXD11;HPS1;HS3ST1;HSPA4L;HSPA8;HUWE1;HYPK;IBA57;ICA1L;ICAM1;ICAM4;ICMT;IER3;IFNAR1;IFNAR2;IGF2;IKZF4;INPP5F;INSL6;IP6K1;IPO5;IPP;IQSEC1;IRAK4;IRF1;ISCA2;ISOC1;ISY1;ITCH;ITGA2;ITGB1;ITGB8;ITPA;ITPKB;JAK1;JUN;KAT2B;KATNAL1;KCNA7;KCNB1;KCND3;KCNJ8;KCNK6;KCNMB1;KDM3B;KDM6B;KIAA0232;KIAA0513;KIAA1147;KIAA1191;KIAA1551;KIAA1841;KIF1BP;KIF23;KIF6;KLB;KLF10;KLF11;KLF3;KLF6;KLF9;KLHDC2;KLHL15;KLHL20;KLHL28;KLHL36;KLRD1;KMT2B;KMT2D;KMT5B;KPNA2;KPNA6;KRT10;LAMC1;LAMTOR1;LAPTM4A;LAPTM4B;LASP1;LATS2;LDHD;LDLR;LEPROT;LGSN;LHFPL2;LIAS;LIMA1;LIMK1;LINC00598;LIX1L;LLPH;LNPK;LOXL1;LPAR2;LPGAT1;LRIF1;LRP12;LRP3;LRPAP1;LRRC58;LRRD1;LSM3;LUZP1;LUZP2;LY6G5B;LYSMD3;LYST;LZIC;M6PR;MAD2L1;MAK16;MAN1C1;MAN2B2;MANEAL;MAP3K13;MAP3K14;MAP3K2;MAP3K3;MAP3K4;MAP7;MAPK1;MAPK9;MAPKAPK5;MAPRE3;MARCH6;MARK2;MASTL;MAVS;MCC;MCL1;MCM5;MDM2;MECP2;MED12L;MED13L;MED16;MED17;MED18;MED21;MELK;METTL8;MFN1;MFN2;MFSD2A;MFSD8;MGLL;MICALL1;MICB;MIDN;MINK1;MINOS1-NBL1;MIXL1;MKNK2;MKRN1;MLLT1;MLLT6;MLXIP;MMP3;MOB1A;MORC1;MORF4L1;MPG;MPPE1;MRPS10;MSANTD4;MSH3;MSMO1;MT-ATP6;MTF1;MTMR3;MTMR9;MTPAP;MTSS1L;MXD1;MXI1;MYC;MYH9;MYLIP;MYLK3;MYO19;MYO1D;MYO1E;MYO1F;MYO9B;MYPN;MZT1;N4BP1;N4BP2L2;NAA30;NAA50;NABP1;NACC2;NAGK;NARS;NBEAL2;NBL1;NBPF15;NCAPD2;NCOA3;NDEL1;NEK8;NETO2;NFAT5;NFATC2IP;NFIB;NFKBIA;NHLRC3;NIN;NIPA1;NKIRAS1;NME6;NOB1;NONO;NPAS2;NPAT;NPEPPS;NPM1;NPNT;NR2C2;NR2C2AP;NR2F6;NR3C1;NR4A1;NRBP1;NRIP3;NSD2;NTHL1;NUAK1;NUBP1;NUDT3;NUFIP2;NUGGC;NUP107;NUP153;NUP188;NUP205;NUP35;NUP58;NUP98;OCIAD1;OCRL;OLAH;ORAI1;ORAI2;ORMDL3;OSR1;OSTM1;OTUD4;OXR1;PAFAH1B1;PAK6;PANK3;PAPD5;PAPD7;PAPOLA;PARD3;PARD6B;PBRM1;PBX3;PBXIP1;PCBP2;PCID2;PCLAF;PCMTD1;PCNX2;PDCD4;PDE3B;PDE4C;PDGFB;PDHB;PDPK1;PDRG1;PDZD11;PEA15;PEAK1;PFAS;PFKFB2;PFKP;PGAM1;PGM2L1;PGP;PHC3;PHF10;PHF6;PHLPP2;PHTF2;PI4KA;PIGO;PIK3R2;PIP4K2A;PIP4K2C;PITPNA;PIWIL2;PKD2;PKMYT1;PKNOX1;PLAGL2;PLEKHA1;PLEKHM1;PLEKHO2;PLRG1;PLS1;PLXNA1;PMAIP1;PNPLA4;PNRC1;POFUT1;POGK;POLE;POLK;POLM;POLR1B;POLR2A;POLR3A;POLR3F;POLR3G;PPAN;PPM1H;PPP1R12B;PPP1R15B;PPP1R3B;PPP3R1;PPP6C;PPP6R2;PPP6R3;PPTC7;PRICKLE4;PRIM1;PRKACB;PRKAR1A;PRKCB;PRKCI;PRNP;PRPF4;PRPF8;PRR14L;PRRG1;PRRG4;PRUNE2;PSMD11;PSMD4;PTEN;PTENP1;PTGER4;PTGES3;PTGFRN;PTGIS;PTP4A1;PTPDC1;PTPN4;PURA;PURB;PVR;PXK;PXYLP1;PYGL;QKI;QRFPR;QSOX1;RAB10;RAB11FIP1;RAB12;RAB22A;RAB30;RAB3IP;RAB42;RAB5B;RAB8B;RABEP1;RABGAP1L;RACGAP1;RAN;RANGAP1;RAP2C;RAPGEF4;RB1;RBBP7;RBM12B;RBM20;RBM23;RBM41;RCCD1;REEP3;REEP5;REST;REV1;REV3L;RFC3;RFK;RFXANK;RFXAP;RGMB;RHEBL1;RHOC;RLIM;RMDN3;RMND1;RMND5B;RNASEH1;RND3;RNF115;RNF145;RNF19B;RNF216;RNF220;RNF34;RNF44;RORA;RPA2;RPF2;RPL14;RPL17;RPL17-C18ORF32;RPL27;RPL30;RPL7A;RPL9;RPRD2;RPS27A;RPS6KA4;RPS6KA5;RRAGD;RRAS2;RRM2;RRN3;RSRP1;RTN2;RUFY2;RUNDC1;RUNX3;RYBP;SACS;SALL3;SAMD12;SAMD4B;SAMD8;SAMD9L;SASH1;SCAMP2;SCAMP5;SCD;SEC16A;SEC23A;SEC23IP;SEMA4B;SEMA7A;SENP1;SEPT2;SERF1A;SERF1B;SERF2;SERINC1;SERTAD2;SESN1;SESN2;SESN3;SET;SF3B3;SFXN5;SGMS1;SGPL1;SGTB;SH3BP4;SH3BP5;SH3GLB1;SH3PXD2A;SHOC2;SIK1;SIKE1;SKI;SKIL;SLAIN2;SLC12A6;SLC16A9;SLC19A1;SLC1A5;SLC22A23;SLC25A33;SLC25A44;SLC25A46;SLC28A1;SLC29A2;SLC2A4;SLC30A1;SLC30A7;SLC35F5;SLC35F6;SLC4A7;SLC5A3;SLC6A4;SLC7A11;SLC9A6;SLCO5A1;SLK;SMAD4;SMAD5;SMAD6;SMAD7;SMG6;SMG7;SMIM13;SMOC1;SNAP47;SNAPIN;SND1;SNTB2;SNX16;SNX9;SOCS4;SOCS5;SOD2;SON;SORCS2;SOWAHC;SOX4;SP2;SP4;SPATA2;SPCS1;SPIB;SPOPL;SPRED1;SPTBN1;SPTLC2;SQSTM1;SRCAP;SREK1IP1;SRPK1;SRSF2;SSH2;SSX2IP;STAC2;STAT3;STC2;STK11;STK11IP;STK17B;STX16;STX4;STX6;STYX;SUCO;SUGP1;SUSD6;SYBU;SYNJ2BP;SYNPO2L;TAD

			A2B;TAF4;TAF6;TAF8;TANC1;TAX1BP1;TBC1D17;TBC1D20;TBCC;TBL1XR1;TCF4;TCF7L2;TELO2;TET3;TFAM;TGFB1;TGFBR2;TGOLN2;THEM4;TIMM17A;TLE3;TLR7;TM4SF5;TMBIM6;TMEM100;TMEM123;TMEM127;TMEM131L;TMEM133;TMEM134;TMEM138;TMEM167A;TMEM168;TMEM196;TMEM200C;TMEM242;TMEM245;TMEM267;TMEM38A;TMEM64;TMEM67;TMEM9B;TMOD3;TMX3;TNFAIP1;TNFAIP8L1;TNFRSF10B;TNFRSF21;TNIP3;TNKS1BP1;TNKS2;TNRC6A;TNRC6B;TOLLIP;TOPORS;TP53INP1;TPK1;TPM4;TPRG1L;TRAF3IP2;TRAK1;TRAM1;TRAPPC10;TRAPPC2;TRIM32;TRIM37;TRIM65;TRIM8;TRIOBP;TRIP10;TRPC4AP;TSG101;TSKU;TSN;TSPAN6;TSR1;TTC9;TTPAL;TUSC2;TUT1;TWF1;TXK;TXLNA;TXNIP;TYSND1;TYW5;U2SURP;UBAP2L;UBC;UBE2J1;UBE2L3;UBE2M;UBE2O;UBE2Q2;UBE2V2;UBE3B;UBFD1;UBN1;UBOX5;UBR1;UBR5;UBXN2A;UGCG;ULK1;UNC45A;UNK;UNKL;UPF1;UQCRC1;USP16;USP21;USP28;USP3;USP32;USP48;UXS1;VAMP7;VAT1;VCPKMT;VDAC1;VEGFA;VLDLR;VMA21;VPS13C;VPS26A;VPS50;VPS53;VTI1A;WAC;WASL;WDR1;WDR26;WDR33;WDR37;WDR53;WDR73;WDR89;WDR92;WEE1;WIPF2;WIZ;WNK3;WNT2B;WSB1;WWC1;XBP1;XIAP;XIRP2;XYLT2;YIPF4;YOD1;YTHDC1;YWHAQ;YWHAZ;YY1;ZADH2;ZBED1;ZBTB18;ZBTB21;ZBTB25;ZBTB3;ZBTB33;ZBTB37;ZBTB4;ZBTB41;ZBTB5;ZBTB6;ZBTB7A;ZBTB9;ZC3H12C;ZC3H7A;ZCCHC14;ZDHHC20;ZFP91;ZFYVE21;ZFYVE26;ZFYVE9;ZIC2;ZIC5;ZMAT3;ZMIZ1;ZMYM1;ZNF107;ZNF12;ZNF143;ZNF148;ZNF174;ZNF180;ZNF202;ZNF217;ZNF264;ZNF280B;ZNF280C;ZNF347;ZNF35;ZNF354B;ZNF384;ZNF385A;ZNF417;ZNF426;ZNF446;ZNF454;ZNF514;ZNF532;ZNF578;ZNF597;ZNF652;ZNF665;ZNF681;ZNF682;ZNF7;ZNF70;ZNF706;ZNF76;ZNF770;ZNF780A;ZNF785;ZNF786;ZNF800;ZNF805;ZNF813;ZNF93;ZNFX1;ZNRF3;ZRANB1;ZSWIM1;ZYG11A
hsa-miR-186-5p-477940_mir	0.330	0.686	AASDHPPT;ABCB1;ABCF2;ABHD2;ABRAXAS2;ABT1;ACAA2;ACER3;ACOD1;ACOT12;ACSL4;ACTL8;ACVR1;ACVR2A;ADIPOR2;AEN;AGBL3;AGO1;AGO2;AGPAT5;AHCTF1;AIMP1;AKAP11;AKAP12;AKR1A1;ALDH16A1;ALDH1B1;ALG10B;AMBRA1;AMD1;AMZ1;ANKRD11;ANKRD17;ANKRD26;ANKRD42;ANKZF1;ANO3;ANTXR2;AP1G1;APC;APEH;ARHGAP39;ARL14;ARL5B;ARL6IP4;ARRB1;ATL2;ATL3;ATP1B3;ATP2A2;ATP6V1B2;ATXN1;AVL9;AZIN1;B2M;BAAT;BBS10;BCL11B;BDNF-AS;BDP1;BEND3;BEND5;BHLHE41;BICD1;BLMH;BLOC1S6;BMT2;BRCA1;BRD1;BTF3;BTF3L4;BTG2;BTLA;BTN3A3;BUB1;C11ORF24;C11ORF74;C18ORF32;C1ORF21;C2CD5;CACNB1;CAMK2B;CAMSAP3;CAND1;CBR1;CBX1;CBX2;CBX3;CC2D2A;CCAR1;CCDC80;CCSAP;CCT4;CCT6A;CD164;CD2AP;CD3EAP;CD46;CDC73;CDK5R1;CDT1;CEBPG;CENPCP1;CEP192;CEPT1;CGGBP1;CGN;CHERP;CHMP2A;CHST11;CHTOP;CKAP5;CLDN23;CLDN9;CLEC12B;CLK1;CLPTM1;CMTM4;CNBP;CNOT4;CNOT6;CNOT6L;COG2;COG8;COX17;COX6A1;COX6A1P2;CRAMP1;CREBL2;CREBRF;CRK;CRNKL1;CSDE1;CSNK1A1;CSNK2A1;CTPS1;CUEDC2;CXCL5;DAGLB;DARS2;DBN1;DCAF12;DCAF16;DCBLD2;DDB1;DDX21;DDX42;DEK;DEPDC1;DHPS;DIEXF;DLC1;DLG3;DLGAP5;DNAJB9;DNAJC21;DNAJC27;DNAJC8;DNASE2;DOCK3;DPYSL5;DSEL;DSP;DUS3L;DUSP8;DYRK3;ECH1;ECHS1;EEF1A1;EEF1D;EEF2;EIF1;EIF2A;EIF3C;EIF3G;EIF4G2;EIF5;ELK1;EMC2;EPHA2;ERCC3;ERCC8;ERI1;ERI2;ERN1;EXOC5;EXT1;EZR;FAAP100;FAHD2A;FAM117B;FAM177A1;FAM192A;FAM217B;FAM57A;FASN;FBXL3;FBXL5;FBXO42;FBXO47;FEM1B;FGF2;FGFR2;FIBP;FIGN;FIZ1;FNIP1;FNIP2;FOCAD;FOXK1;FOXM1;FOXN2;FOXO1;FOXP4;FOXRED2;FPGT;FRK;FRS2;FRY;FUNDC2;FUT11;G2E3;G3BP1;GAB2;GAREM1;GATAD2B;GBP2;GCC2;GCSAML;GGPS1;GINS1;GJA1;GLUL;GMFB;GNAI1;GNG12;GNL3L;GOLGA4;GOLGA7;GOLIM4;GPC5;GRIK5;GUCY1B3;H1F0;HAT1;HAUS6;HDGF;HELZ;HEMK1;HHIP;HIF1A;HIST1H1C;HIST1H1E;HIST1H2BJ;HIST1H2BK;HIST1H2BO;HIST2H3A;HMGCS1;HMGXB4;HMOX2;HNRNPA0;HNRNPA1;HNRNPC;HNRNPL;HNRNPU;HPSE;HSP90AA1;HSP90AB1;HSP90B2P;HUWE1;ID4;IGF2BP3;IGFBP5;IKBIP;ILF3;INPP5F;INSL6;INTU;IRF2BP2;ITGB1;ITGB8;ITM2B;ITPKB;ITSN2;JCAD;JOSD1;JPT2;JUND;KATNAL1;KCTD12;KCTD15;KDELR1;KDM3B;KDM6B;KHSRP;KIAA0895;KIAA1456;KIAA1522;KIF11;KIF14;KIF21A;KIF5B;KIF6;KIT;KLRD1;KMT2C;KMT2D;KMT5A;KNSTRN;KPNA1;KRT3;KRTAP5-6;LAPTM4A;LBP;LBR;LCLAT1;LDHA;LDHD;LIMA1;LINC00346;LINC00598;LLGL1;LPCAT1;LRRC3C;LRRC41;LRRFIP1;LUZP1;LZIC;MAP3K2;MAP3K21;MAP7D1;MAT2A;MATR3;MAVS;MCL1;MDH1;MDN1;MED15;MED4;MED8;MESD;METTL21A;MFF;MIER3;MINK1;MLF2;MOB1A;MOB3A;MRFAP1;MRPL42;MRPL45;MRPS15;MRPS16;MSANTD4;MSL3;MT-ATP6;MT-CO1;MT-CO3;MT-ND5;MTA2;MTHFD2;MTSS1L;MTX3;MYC;MYLIP;MYO19;MYSM1;MZT2B;NAA10;NAA15;NASP;NCAPD2;NCAPD3;NCSTN;NDUFA2;NDUFA4;NDUFA7;NDUFB6;NEK2;NENF;NGLY1;NID1;NKAP;NMT1;NOL8;NRP1;NSD1;NSRP1;NUAK1;NUDT13;NUFIP2;NUP50;NUP62;NUPL2;NVL;NXN;ORAI1;ORC5;P2RX7;PABPC4;PAFAH1B2;PAK5;PANK3;PAPD5;PCDH10;PCGF1;PCNP;PDAP1;PDCD10;PDCD5;PEG10;PER2;PGAM1;PGK1;PHC3;PHEX;PHIP;PHTF2;PIGG;PIP4K2B;PKP2;PLA2G4D;PLEKHH2;PLEKHM3;PLIN3;PLPP4;PLS1;PLS3;PLXNA1;PLXNB2;PMAIP1;PNP;PODXL;POLD3;POLR1B;POLR2D;PON2;POTED;PPARGC1B;PPCS;PPIG;PPL;PPM1B;PPM1F;PPP1R15B;PPP4R2;PPRC1;PRKAB2;PROCA1;PRPF8;PRPSAP1;PRR3;PSAT1;PSMA2;PSMD13;PSMD2;PSMD3;PSMD4;PSMD8;PSMD9;PSME1;PTGES3;PTP4A1;PTPMT1;PTPN13;PTPN6;PTPN9;PTTG1;PUM1;PUM3;PURG;PVT1;QRICH1;RAB11A;RAB12;RAB2A;RAB3GAP1;RA

			B6A;RACK1;RAF1;RAG1;RALA;RALGDS;RAN;RANBP1;RANBP10;RANBP2;RAPH1;RASSF1;RBFOX2;RBM10;RBM12B;RBM27;RBM28;RBM5;RBM8A;RBPJ;RCAN3;RCC1L;RELA;RETREG2;RFK;RGR;RHOB;RIMS3;RIMS4;RLIM;RNASEH1;RNF111;RNF113A;RNF146;RNF149;RNF167;RNF2;RNF40;RNF6;RNPEP;RORA;RPL14;RPL18A;RPL27;RPL29;RPL32;RPL36;RPL4;RPL5;RPL7L1;RPL9;RPLP1;RPS2;RPS21;RPS26;RPS4X;RPS7;RRM2;RRP1B;RSRC2;SAMD15;SASH1;SAT1;SAT2;SCFD1;SCGB2A2;SCRIB;SDE2;SEC24A;SEC24B;SEPT11;SEPT8;SEPT9;SERP1;SERPINA12;SETD2;SETD6;SETD7;SF1;SFT2D2;SHC2;SHMT2;SIGLEC14;SIGLEC9;SIN3B;SLC15A4;SLC1A1;SLC25A13;SLC28A1;SLC29A1;SLC30A1;SLC30A4;SLC30A7;SLC39A10;SLC44A1;SLC4A1AP;SLC6A6;SLC7A5;SLTM;SMAD1;SMAD4;SMAD5;SMARCA2;SMARCA5;SMARCC2;SMN2;SNRNP27;SNRNP35;SNRNP40;SNX6;SOAT1;SON;SP1;SPATA5;SPECC1;SPECC1L;SPEN;SPIRE1;SPRED2;SPRTN;SPTLC1;SPTY2D1;SREK1IP1;SRGAP2;SRP19;SRPK1;SRRM2;SRSF11;SRSF2;SRSF3;SRSF6;SSR2;ST13;ST3GAL5;STAG2;STARD8;STK24;STK25;STK35;STOX2;SUGT1;SUMO2;SUPT16H;SYNJ2BP;TADA3;TARDBP;TARSL2;TBC1D19;TBCA;TBK1;TCP1;TEF;TERF2IP;TES;TEX15;TEX261;TEX35;TGFBR3;TGIF1;TGOLN2;THAP2;THOC1;THOC5;TLE4;TLN1;TLX3;TM9SF3;TMBIM6;TMED2;TMEM11;TMEM135;TMEM183A;TMEM2;TMEM245;TMEM64;TNFRSF10A;TNPO2;TOP1;TOP2A;TRAPPC10;TRIM2;TRIM59;TRIP10;TSC22D2;TSEN15;TSPYL4;TSR1;TTC30A;TTI2;TTYH1;TUBB;TUBB2B;TUBB4B;TUFM;TULP4;TWIST1;TXN2;TXNRD1;UBE2E2;UBE2E3;UBE2K;UBE2Q1;UBL3;UBXN6;UHMK1;ULK1;UNK;UPF1;USP19;USP9X;UTP11;VAV3;VCAM1;VEGFA;VKORC1L1;VPS13B;VPS13C;VPS36;VPS45;WDR33;WDR62;WEE1;WLS;WNK1;WNT5A;XIAP;XPO7;XRCC1;XRCC2;XRN2;YARS;YES1;YRDC;YTHDF1;YWHAE;YWHAH;YY1;ZBTB21;ZBTB33;ZBTB34;ZBTB4;ZC3H15;ZC3H8;ZDHHC6;ZFHX3;ZFP36L2;ZFP90;ZFP91;ZIC5;ZMAT2;ZNF148;ZNF214;ZNF256;ZNF324;ZNF324B;ZNF333;ZNF385A;ZNF426;ZNF512B;ZNF516;ZNF583;ZNF629;ZNF652;ZNF654;ZNF699;ZNF700;ZNF770;ZNF793;ZNF80;ZNF805;ZSCAN16;ZWINT
hsa-miR-99b-5p-478343_mir	-0.858	0.702	ACTG1;ACTR1B;AHSA1;ALDH1B1;ARID3A;CAMSAP3;CHEK1;CNTNAP2;DDHD2;DDX39A;DHFR;DYRK1A;EDC3;EDF1;EEF2;EIF4A3;ENO2;ETV6;FAM171A2;FAM8A1;GRHL1;H2AFZ;HIST1H1C;HK1;HOXA3;IFIT3;IGF1R;IGF2BP1;KBTBD8;MCM3;MFN2;MTOR;NOX4;NPDC1;OAT;PIP4K2B;PLA2G4F;PPP1CB;RAVER2;RNF213;RPS10;RPS2;SMARCA5;SMPDL3B;SP1;TANC2;TMEM127;TMEM30A;TRIB1;TRPS1;UBL7;WDR48
hsa-miR-128-3p-477892_mir	0.494	0.705	A2M;ABCA1;ABCG1;ABHD17C;ACADM;ACSL5;ADAMTS4;ADAMTS5;ADD1;ADO;ADORA2B;AFF4;AGTRAP;AJUBA;AKIRIN1;AKR7A2;ALDH4A1;ALDH9A1;ALG14;ANKFY1;ANKRD40;ANP32E;AP3D1;AP3S2;APEX1;APPBP2;ARF6;ARHGAP12;ARHGAP5;ARID5B;ARL10;ARL5B;ARMT1;ATP5A1;ATP6V1C1;ATP8A1;ATPAF1;AZGP1;B4GALT1;BAG2;BAX;BCL11B;BCL2L15;BEX3;BLOC1S2;BMI1;BMPR2;BOC;BPGM;BPIFB3;BRCA2;BRWD1;BYSL;C11ORF84;C15ORF38-AP3S2;C17ORF75;C19ORF47;C2ORF72;C6ORF120;C8ORF4;CABLES1;CANX;CASC3;CASP3;CASQ2;CCDC117;CCDC36;CCDC59;CCDC6;CCL17;CCNK;CCNT1;CCR4;CD2AP;CD300A;CDH5;CDH6;CDK19;CDY2B;CERK;CETN1;CHEK2;CISD1;CLCA4;CLDN5;CLPP;CLSPN;CMTM6;CNNM2;CNNM4;CNOT1;COASY;COX18;CPEB4;CREB5;CSF1;CSRP2;CTDSP1;CTSZ;CYP20A1;CYP2C9;DACT2;DARS;DAZAP2;DBR1;DCAF12;DCAF7;DCK;DCP2;DCX;DDAH1;DDI2;DDX18;DDX6;DNAJB8;DNAJC27;DNAJC8;DSC2;DYNC2LI1;DYNLL2;DYRK3;E2F3;E2F7;EFEMP2;EGFR;EIF2S2;EIF5;EIF5A2;ELL2;EMC3;EN2;EPB41L1;EPHB1;EPHB2;ESCO2;ETF1;EYA4;FADD;FAM213B;FAM217B;FAM49B;FAM84B;FAM89A;FAM9B;FBLN5;FBXO6;FBXW7;FKBP10;FLCN;FNBP1;FOXN2;FOXP4;FRA10AC1;FRYL;FUBP3;FXYD2;FYN;FZD9;G6PC3;GAS7;GATA6;GBA;GCC2;GCNT2;GDF15;GDNF;GFPT2;GLRX;GNAQ;GNB5;GNG12;GOLGA8B;GOLPH3L;GPAM;GPATCH11;GPI;GPR83;H3F3B;H3F3C;HACD3;HAL;HEY2;HINT1;HIP1R;HLA-DRA;HMGA1;HMOX1;HNRNPF;HOXA10;HOXB3;HOXC6;HRASLS5;HSP90B1;HSPB8;HTRA4;IFITM1;IGF1;IGF2R;ILK;IMP4;INA;INO80D;INTS2;INTS8;ISL1;ITCH;ITPR1;ITSN2;JAK1;JMY;KAT7;KBTBD11;KCNJ6;KCTD14;KDM3B;KIAA0319L;KIAA1191;KIF5B;KIN;KLF4;KLF5;KLHL23;KMT2C;KMT5B;KNG1;KPNB1;KREMEN1;KRTAP13-2;LARP1;LBR;LDLR;LGALS3;LIFR;LILRB5;LIN28A;LIN54;LITAF;LMNB1;LNPEP;LONRF1;LOXL2;LRRC40;LSM1;LSM3;LYSMD3;MACC1;MAP2K1;MAPK14;MAPK6;MAPKAPK5;MARCKSL1;MBL2;MBTD1;MCRIP2;MCTP1;METTL25;METTL8;MFSD14B;MID2;MIER3;MKLN1;MKNK2;MMS22L;MOB1A;MOB1B;MOB3A;MOB3B;MPI;MPP2;MPRIP;MRPL44;MRPS14;MRPS27;MTOR;MUC13;MYO1F;MYO9A;MYPN;NAA50;NANOG;NBEA;NCAPD2;NCK2;NDST1;NDUFC2;NDUFC2-KCTD14;NECTIN4;NEK2;NEURL1B;NLN;NOP2;NOP53;NR1D2;NR2F2;NTRK3;NUFIP2;NUP93;NUS1;NXT1;OR7D2;PAGR1;PAIP2;PARP1;PCTP;PDE3A;PDE3B;PDHX;PDIA5;PDK1;PDPK1;PDS5A;PDZRN4;PEG10;PFKL;PHF14;PI4K2A;PIK3C2B;PIK3R1;PITHD1;PKD1;PLA2G5;PLAG1;PLAGL2;PMEPA1;PNKD;POLR1B;POP4;POPDC2;POU5F1;PPIF;PPP1R14C;PRKAA1;PROSER3;PROX2;PRR11;PRRG4;PSPC1;PTCHD1;PTCHD3;PTEN;PTGS2;PYGO1;R3HDM2;RAB21;RAB22A;RAB8B;RABGAP1;RAP1B;RASSF6;RBBP4;RBBP5;RBM24;RELN;RET;RETREG2;RFX3;RGPD4;RGS14;RGS6;RICTOR;RILPL1;RMND5A;RND1;RNF144B;RNF152;RNF182;RNF187;ROPN1L;RPL21;RPL28;RPS24;RPS6KA5;RREB1;RUNX1;RXRA;S1PR2;SDE2;SDK2;SEC16A;SEC61A1;SERPING

			1;SERTAD2;SERTAD4;SETD3;SETD7;SFXN1;SFXN2;SFXN4;SGPL1;SGPP1;SH3BGRL;SH3D19;SH3RF1;SHOC2;SIRT1;SLC16A1;SLC17A4;SLC1A4;SLC22A14;SLC26A2;SLC35F4;SLC39A11;SLC39A7;SLC46A3;SLC5A3;SLC6A17;SLC7A11;SMAD2;SMCHD1;SNAI1;SNAI2;SNRK;SNRNP27;SNX11;SOS1;SOX11;SOX2;SP1;SPTAN1;SREBF1;SREBF2;SRPRA;SRSF1;SSFA2;SSR1;SSX2;SSX2B;STARD7;STK3;STK35;SUCO;SUMF1;SUPT7L;SUZ12;TAGLN;TAGLN2;TAOK1;TBC1D24;TBC1D2B;TBX3;TGFBR1;TGFBR3;TGOLN2;TIGD2;TIMM10;TLNRD1;TM4SF1;TMBIM6;TMED5;TMEM120B;TMEM167A;TMEM170B;TMEM192;TMEM30A;TMEM68;TMEM91;TMTC3;TNFSF15;TNNI1;TNPO1;TOMM40L;TOPBP1;TOR2A;TP53INP1;TP53INP2;TPPP;TRIM36;TRMT112;TROVE2;TSEN54;TSPAN13;TSPAN6;TSPYL1;TUB;TUBB4B;TUBBP1;TUSC3;TUSC5;TXNIP;UBE2D1;UBE2G2;UBE2N;UBE2W;UBR4;UGCG;UNC13C;USP25;USP46;VEGFC;VPS13C;VPS13D;VPS26B;VPS4B;WASL;WDCP;WDR55;WDR81;WDTC1;WEE1;WNT3A;WRN;XRCC6;YIPF5;YPEL1;YWHAZ;ZBED6CL;ZBTB18;ZBTB20;ZBTB37;ZFHX3;ZFHX4;ZFP1;ZFP36L1;ZIC5;ZNF148;ZNF236;ZNF329;ZNF385A;ZNF394;ZNF490;ZNF552;ZNF562;ZNF573;ZNF614;ZNF652;ZNF800;ZYG11B
hsa-miR-452-5p-478109_mir	0.305	0.706	ADORA2B;AKR1B10;ATP5G3;BAG4;BEND6;BMI1;BMPR2;CALHM5;CASD1;CCDC137;CCDC39;CDK6;CDKN1B;CLASP1;CYP1A1;DCSTAMP;DOT1L;DPYSL2;DSTN;EGLN1;EIPR1;EPM2AIP1;GFRA1;GOT1;HACE1;HARS;HEYL;HOOK3;IL3;IRS1;ITGA9;ITPRIPL1;KRAS;LAMC1;LEF1;LIPA;LNPK;MAP3K1;MED4;MMP2;MRPL19;MTDH;MVK;NOTCH2NL;PDK3;PIGP;RAB3B;RAI1;REST;RHOBTB3;RHOU;RPL7;RPS16;RTP4;SEMA6D;SHPK;SKI;SLC25A37;SMAD4;SMU1;TADA2A;TBK1;TCF4;THRB;TMCC1;TMED2;TNF;TNFAIP8;TXNDC16;UBE2D1;UGGT1;WNK1;ZNF621;ZNF788;ZNF799
hsa-miR-324-5p-478024_mir	-0.673	0.723	AARS;ABCC4;ACIN1;ACTB;ACTN4;ADGRB2;ADORA3;AGAP1;AGO1;AHNAK;AK3;ALDOA;AMZ1;ANAPC13;ANXA6;ANXA7;APOF;ARF1;ARFGAP2;ARHGEF10L;ARID1A;ARID1B;ARID5A;ARID5B;ARNT;ARPP19;ASF1B;ASS1;ATP5B;ATP5H;ATXN2L;BIVM;BOP1;BTBD2;BTN3A1;C8ORF33;CACHD1;CBS;CCND3;CD274;CDC123;CDS1;CELF2;CENPB;CFL1;CHCHD10;CHIC1;CKB;CLMP;CLN6;CLPB;CLPTM1;COL14A1;COL6A1;COPS3;COX5A;CRTC1;CRY1;CS;CTTNBP2NL;CUEDC2;CYP20A1;DAP3;DAZAP2;DBT;DDX11;DDX20;DEAF1;DEF8;DHX8;DNAJC11;DNAL1;DYRK1A;DYRK1B;EDC3;ELOVL5;ESD;ETS1;FAM43B;FAM83H;FBL;FLNA;FOXC1;FOXO1;GANAB;GEMIN5;GID8;GIGYF1;GLI1;GMNN;GNB1;GNB2;GTF3C1;GYS1;H3F3B;HIST2H3A;HMGXB3;HNRNPA1;HNRNPH2;HNRNPL;HOXA9;HSD17B4;HSP90B2P;HSPA1B;HSPD1;HTT;IGDCC3;IPO5;ITM2C;ITPR2;JCAD;KCNG1;KIAA0408;KIAA0907;KIF3B;KLF7;KLHDC3;LACC1;LARS2;LDHA;LDHB;LRRC47;MAPKAPK2;MAZ;MDC1;MEA1;MEMO1;METAP2;MGAT5B;MID2;MKLN1;MPI;MRPL14;MSH6;MTFR1;MTHFD1;MTMR12;MYADM;MYO1C;MYOD1;MZT2A;NANS;NDUFS1;NEBL;NELFB;NEUROD2;NFATC1;NFIX;NKX2-5;NME3;NSD2;NSMCE1;NSMF;NSUN3;NUP160;NUP188;NUP85;OPRL1;OSTC;PA2G4;PACS1;PAK4;PALM;PARM1;PAX3;PCDH11X;PCDH11Y;PDRG1;PDXP;PFAS;PGAM1;PGK1;PIAS4;PIN1;PISD;PKD1;PMS2;POLD1;POLE;POLM;POLR2A;POLR3H;POTEG;POTEM;PPIA;PPIAL4G;PPM1F;PPP1R18;PRPF8;PSMA4;PSMC5;PSMD11;PSMD2;PSMD4;PSME1;PSME3;PTK7;PUM1;PXDN;RAB11FIP1;RAB1B;RAB6A;RACK1;RAD51D;RAN;RAP2B;RAPH1;RARS;RBBP4;RBM23;REEP4;RELA;RER1;RNF26;RPL27;RPL36AL;RPL37;RPL41;RPP30;RPS2;RUSC2;SAP18;SAP30BP;SARS2;SCARB1;SCD;SCD5;SEC23IP;SEC61B;SEPT8;SERTAD2;SF3B2;SGO1;SLC25A3;SLC26A2;SLC39A10;SLITRK4;SMAD2;SMARCD2;SMO;SNX7;SOBP;SP1;SPEN;SPOPL;SRRM1;STXBP4;SYNGR2;TACC3;TAX1BP3;TBC1D10B;TBCB;TCF7L2;TCOF1;TIMELESS;TIMM50;TMCC1;TMEM129;TMEM63B;TMEM8B;TNKS;TOM1;TP53;TRAFD1;TRERF1;TRIM23;TRPC4AP;TUBA1A;TUBA1B;TUBA1C;TUBB;TUBGCP6;TUFM;TUT1;UBA1;UBE2I;UBE2S;UBL5;UBXN11;UGCG;UGT8;UNKL;USP16;USPL1;VANGL1;VMAC;VPS51;WASHC5;WBP2;WIZ;XPO6;YOD1;YWHAH;ZBED1;ZIC5;ZMYND11;ZNF747
hsa-miR-20a-5p-478586_mir	-0.858	0.741	A1CF;AAK1;ABCA1;ABCA3;ABCG8;ABHD15;ABHD18;ABHD2;ABI2;ABL2;ACADSB;ACAP2;ACBD5;ACER2;ACOT9;ACOX1;ACSL4;ACTR2;ACVR1B;ADAR;ADAT2;ADD1;ADGRE2;ADGRL3;ADSS;AFF1;AGFG2;AGMAT;AGO1;AGO3;AGO4;AKAP11;AKR7A2;AKTIP;ALDH18A1;ALDH9A1;ANKFY1;ANKH;ANKIB1;ANKRD12;ANKRD13C;ANKRD33B;ANKRD50;ANKRD52;ANKS4B;AP1G1;AP3D1;AP3S2;APOH;APP;ARAP2;ARCN1;ARFGEF2;ARHGAP1;ARHGAP12;ARHGAP35;ARHGEF18;ARHGEF7;ARID4B;ARL1;ARL9;ARMT1;ARPC2;ARSJ;ASB1;ASB16;ATAD2;ATAT1;ATG14;ATG16L1;ATG2A;ATG2B;ATL3;ATP1B3;ATP2B1;ATP6V0E1;ATP8B2;ATXN1;ATXN7L3B;B2M;B4GALT2;BACH1;BAGE5;BAMBI;BBX;BCAS4;BCL2;BCL2L11;BCL2L2;BHMT2;BICD2;BLOC1S3;BMP2;BMP8B;BMPR2;BMT2;BNIP2;BRI3BP;BRMS1L;BSCL2;BTBD2;BTBD7;BTF3L4;BTG2;BTG3;BTN3A1;BTN3A2;BTN3A3;BZW1;C11ORF54;C11ORF68;C12ORF65;C14ORF119;C14ORF28;C15ORF40;C15ORF41;C16ORF52;C16ORF70;C17ORF75;C18ORF32;C1ORF50;C2ORF69;C3ORF38;C6ORF120;C7ORF43;C9ORF40;C9ORF78;CABLES1;CADM2;CAMK2N2;CAMTA1;CAPN15;CAPRIN2;CAPZA2;CASP2;CAV1;CAVIN1;CBX1;CBX5;CBX8;CCDC125;CCDC137;CCDC198;CCDC30;CCDC47;CCDC6;CCDC71L;CCDC88C;CCL1;CCL5;CCNB1;CCND1;CCND2;CCP110;CCSER2;CD28;CD47;CDIPT;CDK16;CDK19;CDKN1A;CDKN2AIPNL;CDT1;CENPQ;CEP104;CEP120;CEP170;CEP57;CEP72;CEP97;CERCAM;CERS2;CFL2;CHAF1A;CHD9;CHIC1;CHSY1;CHURC1;CIT;CKAP2;CLEC12B;CLIC4;CLIP4;CLOCK;CMPK1;CMTR2;CNKSR3;CN

			OT4;CNOT6L;CNOT7;CNTN1;COIL;COX19;COX5A;COX6B1;CPOX;CPS1;CPT1A;CRCP;CREB1;CRIM1;CRISPLD2;CRK;CROT;CRTC3;CRY2;CSDE1;CSNK1A1;CTR9;CTSA;CTSS;CXORF38;CYB5A;CYBRD1;CYCS;CYCSP5;CYLD;DAPK3;DCAF8;DCBLD2;DCTN5;DCTN6;DDHD1;DDI2;DDX5;DEGS1;DENND5B;DHODH;DIAPH2;DIS3L;DLC1;DLG5;DNAJB13;DNAJB4;DNAJB6;DNAJB9;DNAJC10;DNAJC27;DNAJC28;DNAL1;DNM1L;DNMT1;DNTTIP2;DPP9;DPY19L4;DPYSL2;DRAXIN;DSPP;DSTYK;DTX2;DUSP18;DUSP2;DYNC1LI2;DYRK2;E2F1;E2F2;E2F3;E2F5;ECI1;EEA1;EFCAB11;EFCAB14;EGLN3;EGR2;EIF2B2;EIF2S1;EIF4A2;EIF4G2;EIF4H;EIF5A2;ELAVL2;ELK4;ELMSAN1;ELOC;EMC1;EMSY;ENPP5;ENTPD4;ENTPD7;EPAS1;EPHA4;EPS15L1;ERAP1;EREG;ERGIC2;ESR2;ETF1;ETV1;EXO5;EZH1;F2R;F2RL1;F2RL3;FAAP24;FAF2;FAHD1;FAM102A;FAM117B;FAM126B;FAM129A;FAM160B1;FAM210A;FAM213A;FAM241A;FAM46C;FAM57A;FAM83D;FAM89A;FAXC;FBL;FBXL5;FBXL7;FBXO10;FBXO21;FBXO3;FBXO31;FBXO48;FCHO2;FEM1A;FEM1B;FEM1C;FEZ2;FGF7;FGFR1OP;FHDC1;FHL3;FICD;FJX1;FKBP14;FLNA;FMNL2;FMNL3;FNBP1L;FOXC1;FOXJ2;FOXJ3;FOXK1;FOXK2;FOXQ1;FOXRED2;FRMD6;FRS2;FUT10;FXYD5;FYCO1;FZD9;GAB1;GABBR1;GABPAP;GAK;GATA6;GATAD1;GBF1;GBP3;GDF11;GDF5OS;GDI2;GEMIN8;GID4;GIGYF1;GINS4;GJA1;GLO1;GNAS;GNB5;GNPTAB;GNS;GOLGA1;GOLGA2;GPAM;GPATCH11;GPN2;GPR137B;GPR155;GPR157;GPR183;GPRIN3;GRAMD1A;GRK3;GRK7;GRPEL2;GTF2H2C;GTF2H3;GTF2IRD2;GTF2IRD2B;HAS2;HAUS2;HAUS8;HBP1;HECA;HEXIM1;HIF1A;HIF1AN;HIP1;HIST1H2BD;HIST1H2BG;HIST1H2BJ;HMBOX1;HMG20A;HMGB1;HMGB2;HMGB3;HNRNPR;HOOK3;HOXD11;HS3ST1;HSPA4L;HSPA8;HYPK;ICA1L;ICMT;IER3;IFNAR1;IFNAR2;IKZF5;IL17RC;INPP5F;INSIG1;INTS3;IPP;IQSEC1;IRAK4;IRF2;ISCA2;ISOC1;ISY1;ITCH;ITGA2;ITGB1;ITGB8;ITPKB;JAK1;KATNAL1;KCNA7;KCNB1;KCND3;KCNJ8;KCNK6;KCNMB1;KDM4D;KDM6B;KIAA0100;KIAA0232;KIAA0513;KIAA1147;KIAA1191;KIAA1551;KIAA1841;KIF23;KIF26B;KIF2C;KIF6;KIT;KLF10;KLF3;KLF6;KLHL15;KLHL20;KLHL28;KLHL36;KLRD1;KMT2B;KMT5B;KPNA2;KPNA6;KRT10;L2HGDH;LAMC1;LAMTOR1;LAPTM4A;LASP1;LDHB;LDHD;LDLR;LEPROT;LGSN;LIAS;LIMA1;LIMK1;LINC00598;LLPH;LPAR2;LPGAT1;LRIF1;LRP12;LRPAP1;LRRC58;LRRD1;LSM3;LUZP2;LY6G5B;LYPD6;LYSMD3;LZIC;M6PR;MAD1L1;MAGOHB;MAK16;MAN1C1;MAN2B2;MANEAL;MAP2K3;MAP3K12;MAP3K14;MAP3K2;MAP3K3;MAP3K5;MAP7;MAPK1;MAPK9;MAPKAPK5;MAPRE3;MARCH6;MASTL;MAVS;MCC;MCL1;MDM2;MECP2;MED16;MED17;MED18;MEF2D;MELK;METTL22;METTL8;MFN1;MFSD2A;MFSD8;MICB;MIDN;MINK1;MINOS1-NBL1;MIXL1;MKNK2;MKRN1;MLF2;MLLT1;MLXIP;MORC1;MORF4L1;MORF4L2;MPHOSPH8;MPPE1;MRPL13;MRPS10;MRS2;MSH3;MSMO1;MT-ATP6;MTF1;MTMR3;MTMR9;MTPAP;MUC17;MXI1;MYC;MYH9;MYLIP;MYLK3;MYO1D;MYO1F;MYPN;N4BP1;N4BP2L2;NAA50;NABP1;NACC2;NAGK;NAP1L1;NARS;NAXE;NBL1;NCAPD2;NCOA3;NCOR2;NEK8;NETO2;NFAT5;NFATC2IP;NFIB;NFKBIB;NHLRC3;NIN;NIPA1;NKIRAS1;NME6;NPAT;NPNT;NR2C2;NR2F6;NR3C1;NRAS;NRBP1;NRIP3;NSD2;NUDT21;NUDT3;NUFIP2;NUGGC;NUP188;NUP214;NUP35;NUP98;OCIAD1;OCRL;OLAH;ORAI1;ORAI2;ORMDL3;OSTM1;OTUD4;OXR1;P3H4;PAFAH1B1;PAIP1;PAK6;PANK3;PAPD5;PAQR5;PARD3;PARD6B;PBXIP1;PCBP2;PCLAF;PCMTD1;PCNX2;PCNX4;PDE4C;PDGFB;PDHB;PDPK1;PDRG1;PDZD11;PEA15;PEAK1;PFKFB2;PFKP;PGK1;PGM2L1;PHC1;PHF6;PHF8;PHLPP2;PHTF2;PHYH;PIGO;PIP4K2A;PIP4K2C;PITPNA;PIWIL2;PKD1;PKMYT1;PKNOX1;PLAGL2;PLEKHM1;PLEKHM3;PLEKHO2;PLRG1;PLS1;PLXNA1;PMAIP1;PNPLA4;PNRC1;POFUT1;POGZ;POLK;POLM;POLR1B;POLR3A;POLR3F;POLR3G;PPAN;PPARG;PPP1R12B;PPP1R15B;PPP1R3B;PPP2R1A;PPP2R2A;PPP3R1;PPP6C;PPP6R3;PRICKLE4;PRIM1;PRKACB;PRKAR1A;PRKCB;PRKD3;PRKG1;PRNP;PRPF4;PRR14L;PRRC2C;PRRG1;PRRG4;PSD3;PSMD2;PTEN;PTENP1;PTGER4;PTGES3;PTGFRN;PTGIS;PTP4A1;PTPDC1;PTPN23;PTPN4;PTPRO;PTPRS;PURA;PURB;PVR;PXK;PYGB;QKI;QRFPR;QSOX1;RAB10;RAB11FIP1;RAB12;RAB22A;RAB30;RAB3IP;RAB42;RAB5B;RABEP1;RABGAP1L;RACGAP1;RAN;RANGAP1;RAP2C;RAPGEF4;RB1;RB1CC1;RBBP7;RBL1;RBL2;RBM10;RBM12B;RBM20;RBM41;RCCD1;REEP3;REEP5;REST;REV1;RFC3;RFK;RFXANK;RFXAP;RGMB;RGS5;RHOC;RLIM;RMND1;RNASEH1;RNF115;RNF19B;RNF216;RNF34;RNGTT;RORA;RPA2;RPF2;RPL14;RPL17;RPL17-C18ORF32;RPL18A;RPL21;RPL30;RPL31;RPRD1A;RPRD2;RPS10;RPS27;RPS27A;RPS6KA5;RRAGD;RRAS2;RRM2;RRN3;RSRP1;RTFDC1;RTN2;RUFY2;RUNDC1;RUNX1;RUNX3;SACS;SALL3;SAMD12;SAMD8;SAMD9L;SARAF;SCAMP2;SCAMP5;SCD;SEC16A;SEC23A;SELENBP1;SEMA4B;SEMA7A;SENP1;SEPT2;SERF1A;SERF1B;SERF2;SERINC1;SESN1;SESN2;SESN3;SF3B3;SGMS1;SGPL1;SGTB;SH3BP5;SH3GLB1;SHOC2;SIK1;SIKE1;SIRPA;SKI;SKIL;SLAIN2;SLC12A6;SLC16A9;SLC1A5;SLC22A23;SLC25A28;SLC25A33;SLC25A44;SLC25A46;SLC28A1;SLC30A1;SLC30A7;SLC35F5;SLC35F6;SLC4A7;SLC5A3;SLC6A4;SLC7A11;SLCO5A1;SLK;SMAD4;SMAD5;SMAD6;SMAD7;SMIM13;SMOC1;SNAP47;SNTB2;SOCS4;SOCS5;SOD2;SON;SORCS2;SOX4;SP2;SP4;SPCS1;SPIB;SPOPL;SPRED1;SPTLC2;SQSTM1;SRCAP;SREK1IP1;SRSF2;SSH2;SSRP1;SSX2IP;STAC2;STAT3;STIL;STK11IP;STK17B;STX4;STX6;SUCO;SUGP1;SUSD6;SYNJ2BP;SYNPO2L;TADA2B;TANC1;TAX1BP1;TBC1D15;TBC1D17;TBL1XR1;TCEA1;TCEAL1;TCF4;TCF7L2;TDRD3;TET3;TFAM;TGFBR1;TGFBR2;TGOLN2;THBS1;

			THEM4;TIMM17A;TIMP2;TLR7;TM4SF5;TMBIM6;TMEM100;TMEM123;TMEM127;TMEM131L;TMEM133;TMEM134;TMEM138;TMEM167A;TMEM196;TMEM200C;TMEM242;TMEM245;TMEM267;TMEM38A;TMEM64;TMEM67;TMEM97;TMEM9B;TMOD3;TMX3;TMX4;TNFAIP1;TNFAIP8L1;TNFRSF10B;TNFRSF21;TNIP3;TNKS2;TNRC6A;TNRC6B;TOMM20;TOPORS;TP53;TP53INP1;TPK1;TPM4;TPRG1L;TRAF3IP2;TRAPPC10;TRAPPC2;TRIM32;TRIM37;TRIM65;TRIM8;TRIOBP;TRIP10;TSG101;TSKU;TSPAN6;TSR1;TTC9;TTPAL;TUBB;TWF1;TXK;TXLNA;TXNIP;U2SURP;UBC;UBE2C;UBE2Q2;UBE2V2;UBFD1;UBOX5;UBR5;UBXN2A;UEVLD;UGCG;ULK1;UNK;UQCRC1;USP10;USP16;USP28;USP3;USP32;USP48;UXS1;VCPKMT;VDAC1;VEGFA;VEZF1;VPS13C;VPS26A;VPS50;VPS53;VTI1A;WAC;WASL;WBP4;WDR1;WDR37;WDR53;WDR73;WDR89;WDR92;WEE1;WIPF2;WNK3;WSB1;WWC1;XIAP;XIRP2;XYLT2;YBX1;YIPF4;YOD1;YTHDC1;YWHAZ;ZBED1;ZBTB18;ZBTB25;ZBTB33;ZBTB37;ZBTB4;ZBTB5;ZBTB6;ZBTB7A;ZBTB9;ZC3H12C;ZDHHC20;ZFYVE21;ZFYVE26;ZFYVE9;ZMAT3;ZMYM1;ZNF107;ZNF12;ZNF174;ZNF180;ZNF202;ZNF264;ZNF280B;ZNF280C;ZNF331;ZNF347;ZNF35;ZNF354B;ZNF385A;ZNF398;ZNF417;ZNF426;ZNF446;ZNF454;ZNF514;ZNF532;ZNF578;ZNF597;ZNF598;ZNF652;ZNF665;ZNF681;ZNF682;ZNF7;ZNF70;ZNF706;ZNF770;ZNF780A;ZNF785;ZNF786;ZNF800;ZNF805;ZNF93;ZNFX1;ZRANB1;ZYG11A
hsa-miR-660-5p-478192_mir	0.827	0.742	ABCA12;ADM;ARHGEF12;ARL4C;ATP13A4;BZW1;C18ORF25;C1ORF56;C2CD4B;CCDC47;CDK2;CERS4;COL4A3BP;CRIPT;DCBLD2;DYNLL2;E2F3;EIF1;ENPP5;FAM221B;FBXO28;GALNT7;HNRNPA1;HUWE1;LANCL3;MECP2;MED10;MED28;MYH11;MYOCD;NAP1L1;NUP50;PCSK2;PMAIP1;SEC22C;SMN1;SMN2;STARD7;TFCP2;TMLHE;UBA6;UBE2K;UGCG;WNK3;XKR7;XRRA1;YTHDF1;YWHAH;ZFYVE26;ZHX1
hsa-miR-424-5p-478092_mir	-1.278	0.750	ABCC6;ABHD2;ABL2;ACTR2;ACTR3B;ACVR2A;ADORA3;ADRA2B;AGO2;AGO4;AHNAK2;AKR1B10;AKT3;ALDH3B1;AMOT;AMOTL1;ANAPC13;ANKHD1;ANKMY1;ANKRD17;ANKRD36;ANLN;AP2B1;AP5Z1;APBB2;APP;ARHGAP12;ARHGAP32;ARHGDIA;ARIH1;ARMC12;ASCC1;ASGR2;ASH1L;ATAD5;ATF6;ATG14;ATG9A;ATP5G3;ATXN7L3B;AURKAIP1;AVL9;AXIN2;B3GNT2;BAG4;BAZ2A;BAZ2B;BCL2L12;BSPRY;BTN3A3;BZW1;C11ORF24;C16ORF58;C16ORF72;C1ORF21;C1ORF226;C21ORF62;C3ORF36;CA8;CACUL1;CALU;CAMSAP1;CANX;CAPZA2;CARD10;CARM1;CASK;CASKIN1;CBX2;CBX4;CBX6;CCDC80;CCDC83;CCDC88C;CCIN;CCND1;CCND2;CCND3;CCNE1;CCNE2;CCNF;CCNT1;CD180;CD274;CD2AP;CDADC1;CDC14A;CDC25A;CDC37L1;CDC42SE2;CDCA4;CDK1;CDK17;CDK6;CDX2;CEP55;CHAC1;CHEK1;CHIC1;CHMP4B;CLEC2D;CLIP4;CLSPN;CLU;CLUH;CMTM4;CNIH4;CNKSR3;CPEB3;CPSF7;CREBL2;CREBRF;CRIM1;CRK;CRKL;CSNK1E;CTDSPL;CUL2;CUL3;CYP26B1;DCAF17;DCTN5;DDX3X;DECR1;DENND6A;DIAPH1;DLGAP3;DMPK;DMRT2;DMTF1;DNAJA1;DNAJC10;DOCK11;DYNLL2;DYRK3;E2F7;EFNB2;EFTUD2;EIF1AX;EIF2B2;EIF4G2;ELK4;EN2;ENTPD1;EPM2AIP1;EXT1;FAM103A1;FAM122B;FAM229B;FASN;FBXL18;FBXL20;FCF1;FGF2;FGFR1;FGFR4;FKBP1A;FLCN;FLOT2;FOXK1;FURIN;FZD6;FZD9;GABARAPL1;GABPA;GALNT1;GATAD2A;GLP2R;GNAL;GNAT1;GNB1;GNG12;GOSR1;GPATCH8;GPR180;GPR27;GPRC5A;GRAMD2B;GRB2;GSG1;GTF2A1;HAUS3;HCFC2;HDGF;HEYL;HIF1A;HIGD1A;HIST1H2BK;HIST2H2AA3;HIST2H2BE;HNRNPA1;HNRNPA1L2;HNRNPA2B1;HNRNPDL;HOXA10;HOXA3;HOXC8;HSP90B2P;HSPA1B;HSPA4L;HSPA8;HSPE1-MOB4;IARS;IER2;IFIH1;INSL6;IPPK;ITGA2;ITPR1;IVNS1ABP;JARID2;JPT2;KANK1;KDM5B;KIAA0895;KIAA1456;KIF23;KIF5B;KLHL15;KLHL40;KMT2D;KPNA1;KPNA3;KRT33B;L2HGDH;LAMC1;LAMP2;LAMTOR4;LANCL1;LRIG2;LRRFIP2;LSM11;LUC7L3;LURAP1L;LUZP1;MAFK;MAGED1;MAP2K1;MAP2K3;MAP3K7;MAP4K2;MBD4;MCFD2;MINK1;MKX;MOB4;MSANTD4;MSL1;MTFR1L;MTHFR;MTMR3;MYB;MYO5A;N4BP1;NAA25;NAPG;NCKAP1;NEGR1;NFIA;NFIC;NNT;NOTCH2;NR6A1;NUCKS1;NUFIP2;NUP50;OCRL;ODF2L;OGT;ORC4;OSBPL3;OSCAR;PAFAH1B2;PAG1;PAGR1;PAK2;PDCD1;PDE4D;PDIA6;PHC3;PHF19;PHKA1;PHLPP2;PHYHIP;PI4K2B;PIAS1;PIK3R1;PIM1;PISD;PLAG1;PLEKHA1;PLEKHB2;PLPBP;PLPP3;PLRG1;PNISR;PNPLA6;PNRC2;POLDIP3;POLR2E;POM121C;PPIG;PPIL1;PPIP5K2;PPM1A;PPP1R11;PPP2R5C;PPP6R3;PRDM4;PRICKLE2;PRKAA1;PRKAR2A;PRSS21;PSAT1;PTCH1;PTPRD;RAB15;RAB23;RAB3IP;RACGAP1;RAD23B;RALGAPB;RAPH1;RARB;RASEF;RASSF2;RBBP6;RBM4B;RBPJ;RCAN3;RECK;REL;RFK;RIMS3;RNF149;RNF168;RNPS1;RPL14;RPRD1B;RPRD2;RPS6KB1;RS1;RTN4;RUBCN;RUNX1T1;SACS;SALL1;SBNO1;SCAMP4;SCOC;SEC24A;SEC61A1;SERBP1;SESTD1;SETD1B;SH3BP4;SHOC2;SIAH1;SIDT2;SIK1;SKI;SLC25A12;SLC29A1;SLC2A3;SLC35E2B;SLC39A9;SLCO3A1;SMAD3;SMAD7;SMARCA5;SMDT1;SMURF1;SNCG;SNRPB2;SNTB2;SNX16;SOCS2;SOCS5;SOCS6;SOWAHC;SPI1;SPRED1;SREK1;SRPRA;SRPRB;SRSF1;SSRP1;SSU72;STK38;STRADB;SYNRG;SYPL1;SZRD1;TAF13;TAOK1;TARBP2;TBPL1;TBRG1;TFAP2A;TGFBR3;THRAP3;TLK1;TLL1;TM4SF1;TM7SF3;TMEM100;TMEM161B;TMEM189;TMEM189-UBE2V1;TMEM245;TMF1;TNFSF9;TNRC6B;TOB2;TPM2;TPM3;TRAK1;TRIM35;TSC22D2;TTC39A;TTLL5;TUBB2A;TXNIP;UBE2H;UBE2Q1;UBE2V1;UBN2;UBR3;UGT2B4;USP15;USP42;USP48;USP53;VAV2;VEGFA;VOPP1;VPS4A;VSIR;WDR13;WEE1;WIPI2;WNK3;XKR7;YIPF6;YRDC;YTHDC1;YWHAH;YWHAQ;ZBTB10;ZBTB16;ZBTB3

			4;ZCCHC3;ZDHHC16;ZFHX4;ZFP28;ZMAT3;ZNF267;ZNF275;ZNF284;ZNF367;ZNF391;ZNF449;ZNF460;ZNF585B;ZNF620;ZNF622;ZNF691;ZNF704;ZNRF1;ZNRF2;ZNRF3
hsa-miR-223-3p-477983_mir	-0.100	0.761	ABCB1;ARL8B;ARTN;ATM;BAG2;C9ORF40;CACNG8;CAPRIN1;CARM1;CCL3;CDC27;CDK2;CDS1;CFTR;CHMP2B;CHUK;CNTN1;CXCL2;CYB5A;E2F1;ECT2;EPB41L3;FABP7;FBXW7;FOXO1;FOXO3;GPATCH8;HAX1;HEXIM1;HSP90B1;IGF1R;IL6;IL6ST;INSL6;ITGB1;LATS2;LGALS8;LIF;LMO2;MAFB;MDM2;MEF2C;MKNK2;MSMO1;MTRF1L;MYL9;NAMPT;NFIA;NFIX;NLRP3;NMNAT2;NOVA2;NSUN3;PARP1;PAX4;PAX6;PDZD8;PHF19;POLR3G;POTEG;POTEM;PRDM1;PRRC2C;PTBP2;RANGAP1;RHOB;RRAS2;SCARB1;SECISBP2L;SEMA3A;SEPT2;SESN3;SINHCAF;SLC12A7;SLC2A4;SLC7A5;SMARCD1;SNX24;SP1;SP3;SPPL2A;STAT1;STAT3;STAT5A;STMN1;SYNGR2;TAL1;TMEM64;TMEM67;TOX;TP53;TRPV2;TWF1;WASL;ZBTB18;ZEB1;ZNF365;ZNF460
hsa-let-7b-5p-478576_mir	-0.617	0.775	AARSD1;AATF;ABCB10;ABCB8;ABCC1;ABCF1;ABHD17C;ABL1;ABT1;ACACA;ACER2;ACOT9;ACPP;ACSL1;ACTA1;ACTB;ACTG1;ACTN4;ACVR1;ADCK2;ADCY1;ADGRG1;ADGRL2;ADH5;ADIPOR2;ADNP;ADRM1;AFF4;AFG1L;AGFG2;AGL;AGO1;AGO2;AGO3;AHCTF1;AHCYL1;AHCYL2;AHR;AIDA;AK4;AKAP8;AKR1A1;AKR1B1;AKT2;ALDH7A1;ALG3;AMD1;AMPH;ANAPC1;ANKRD17;ANKRD46;ANKRD52;ANKZF1;ANP32E;ANTXR1;ANXA8;ANXA8L1;AP1S1;AP3M1;APPL1;APRT;AQP6;ARCN1;AREL1;ARFIP2;ARHGAP17;ARHGAP26;ARHGEF5;ARID3A;ARID3B;ARIH1;ARL15;ARL6IP1;ARL8B;ARPP19;ASCC3;ASIC1;ASNA1;ASPA;ASPSCR1;ATAD3B;ATE1;ATG12;ATG4B;ATG9A;ATOX1;ATP1A1;ATP2A2;ATP6V0A1;ATP6V1F;ATP6V1G1;ATXN1L;ATXN2;ATXN2L;ATXN7L3;ATXN7L3B;AUP1;AURKA;AURKB;BACH1;BAG5;BAG6;BAHD1;BAZ1A;BAZ1B;BAZ2A;BBS7;BCAS4;BCAT1;BCL7A;BCOR;BCORL1;BEND4;BFSP1;BGLAP;BIRC5;BIRC6;BMP7;BNIP3L;BRD2;BRI3BP;BRPF1;BRPF3;BTBD9;BTG2;BZW1;BZW2;C11ORF52;C11ORF57;C12ORF4;C12ORF49;C19ORF47;C19ORF53;C1GALT1;C1ORF21;C1ORF210;C1ORF27;C1RL;C5ORF15;C5ORF24;C5ORF51;C6ORF62;CA12;CACNG8;CALCOCO2;CALU;CAPG;CARHSP1;CASTOR2;CBFB;CBX5;CBX6;CCDC115;CCDC134;CCDC186;CCDC71;CCNA1;CCNA2;CCNB1;CCNB2;CCND1;CCND2;CCND3;CCNF;CCNG1;CCNJ;CCNK;CCNT2;CCNY;CD151;CD2BP2;CD59;CD81;CD99;CDC25A;CDC34;CDCA7;CDCA8;CDIPT;CDK6;CDKAL1;CDKN1A;CDKN1B;CDV3;CELF1;CENPB;CENPV;CEP120;CEP126;CEP135;CHAF1A;CHD1;CHD3;CHD4;CHD7;CHMP2A;CHMP3;CHPF2;CHRAC1;CHTOP;CIAO1;CIZ1;CKAP2;CKB;CKS2;CLCA2;CLDN12;CLINT1;CLPX;CLUH;CMC1;CNDP2;CNNM3;CNOT1;CNOT2;COIL;COL3A1;COL8A1;COLEC12;COMMD9;COPG1;COPS2;COX6B1;COX7B;CPA4;CPEB1;CPEB3;CPEB4;CPED1;CPSF1;CPTP;CRKL;CRX;CRY2;CS;CSNK1D;CSNK2A1;CTBP2;CTCF;CTHRC1;CTPS1;CTR9;CUL1;CUL2;CUL3;CUX1;CXCL8;CYP1A2;CYP2J2;CYSTM1;DAB2IP;DBNDD2;DBNL;DCAF7;DCD;DCTD;DCTPP1;DDX10;DDX17;DDX18;DDX20;DDX21;DDX28;DDX41;DDX49;DEDD;DENND4B;DENR;DFFA;DHTKD1;DHX16;DHX33;DHX57;DHX9;DIABLO;DIAPH1;DICER1;DISC1;DLAT;DLC1;DMD;DNA2;DNAAF5;DNAH9;DNAJC1;DNAJC11;DNAJC28;DNAJC8;DNAL1;DNM3;DNMBP;DOCK5;DPF2;DPYSL5;DRAXIN;DRG2;DSG2;DSP;DTX3L;DUSP1;DUSP12;DUSP23;DVL3;DYNC1H1;DYRK3;DZIP1;E2F2;E2F3;E2F5;E2F6;E2F7;EAF1;ECHDC1;EDEM3;EDN1;EEF1A1;EEF1E1;EEF2;EFCAB14;EFHD2;EHD4;EIF2AK1;EIF2B3;EIF3C;EIF3D;EIF4A1;EIF4A2;EIF4A3;EIF4G2;ELK4;ELMOD2;ELOVL1;EMC6;EMILIN2;ENG;ENTPD4;ENTPD6;EP300;EPB41L3;EPHA4;ERAP2;ERC1;ERCC1;ERGIC2;ERGIC3;ERO1A;ESPL1;ETFA;EZH2;F2;FADS2;FAM104A;FAM105A;FAM126A;FAM131A;FAM136A;FAM213A;FAM222B;FAM43A;FAM49B;FAM57A;FAM83G;FAM84B;FAM96A;FANCD2;FARP1;FBXL20;FBXW2;FEN1;FGFRL1;FIGN;FLAD1;FLII;FLNA;FMNL3;FMO4;FNDC3A;FNDC9;FOXK1;FOXRED1;FOXRED2;FPR1;FSTL1;FTO;FUT10;FXN;FXR2;FZD9;GABPAP;GABPB1;GALNT11;GALNT2;GAPDH;GAPVD1;GATA6;GATC;GATM;GBF1;GCN1;GDE1;GDF11;GDPD5;GEMIN5;GEMIN7;GFM2;GGA3;GGCT;GGPS1;GLB1;GLO1;GNAS;GNB1;GNG5;GOLGA4;GPAT4;GPATCH1;GPATCH4;GPHN;GPI;GPM6B;GPX7;GRPEL2;GSK3A;GSPT1;GSR;GTF2I;GTF3C1;GTF3C4;GTPBP3;GYG1;GYS1;GYS2;GZF1;HADHA;HAND1;HARS;HASPIN;HAUS6;HCFC1;HELLS;HERPUD1;HES1;HGH1;HGS;HIF1A;HIPK1;HIST1H1C;HIST1H2BD;HIST1H2BK;HIST1H3B;HK1;HMGA1;HMGA2;HMGB1;HMGCS1;HNRNPDL;HNRNPF;HNRNPL;HNRNPUL1;HOXD11;HRAS;HS2ST1;HSF2;HSP90AA1;HSPA1B;HSPA8;HTT;HUWE1;IARS2;IBA57;ICOSLG;IDI1;IFIT5;IFNB1;IFNLR1;IFRD1;IGDCC4;IGF1R;IGF2BP1;IGF2BP2;IGF2BP3;IGHMBP2;IGSF3;IGSF8;IKZF3;IL17RC;IL6R;IMPDH1;IMPDH2;INPPL1;INTS1;INTS11;INTS14;INTS5;INTS7;IP6K1;IPCEF1;IPO11;IPO4;IPO7;IPO8;IPO9;IRF2BP2;IRS2;IRS4;ISOC2;ITGA3;ITGB5;JAG1;KCNC4;KCTD12;KCTD21;KDELR1;KDM4A;KHSRP;KIAA0141;KIAA0391;KIAA0930;KIAA1143;KIAA1328;KIAA1549;KIAA1586;KIDINS220;KIF1C;KIF27;KIF2A;KIFC1;KLHDC8B;KLHL11;KMT2D;KPNA5;KPNA6;KREMEN1;KXD1;KYAT3;L3MBTL4;LACTB2;LARP1;LBR;LDLR;LEFTY1;LGR4;LIMD2;LIN28A;LIN28B;LLPH;LMAN2;LPCAT3;LPGAT1;LPL;LRIG1;LRIG3;LRRC20;LRRC40;LRRC41;LRRC47;LRRC8A;LSG1;LSM6;LTA4H;LYN;LZIC;MAB21L1;MAGEA12;MAGEA3;MAGEA6;MANSC1;MAP2K2;MAP2K7;MAP4;MAP7;MAP7D1;MAPK1;MAPK6;MARCH6;MARCKSL1;MARS;MARS2;MAT2B;MBD2;MBD6;MCAT;MCF2L2;MCM4;MCM7;MDM4;MED13;MED14;MED25;MED28;MEF2C;MEF2D;MEIS3P1;MFSD3;MFSD8;MGME1;MIB1;MIDN;MIEF1;MIEN1;MIER2;MIOS;MIPEP;MKRN2;MLLT1;MLLT10;MMACHC;MMS22L;MOB1A

			;MOB1B;MOV10;MPG;MPND;MRM1;MRM3;MRPL12;MRPL37;MRPL47;MRPS11;MRPS24;MRPS33;MSANTD2;MSI1;MSI2;MSN;MT-ATP6;MT-CO1;MT-CO2;MT-CO3;MT-ND1;MT-ND2;MT-ND4;MT-ND4L;MT-ND5;MTFP1;MTPN;MTRR;MTSS1L;MTUS1;MTX3;MXD1;MYC;MYCBP;MYO1C;MYO1E;NAA10;NAA15;NAA20;NAA25;NAA30;NAA40;NAA50;NABP2;NACA;NAF1;NAP1L1;NARS2;NAT8L;NBPF15;NCAPD2;NCAPG2;NCKIPSD;NCOA3;NCOA5;NCOR1;NDRG1;NDUFA10;NDUFA4P1;NDUFAF7;NEDD4;NEK9;NFATC1;NFATC3;NFKBIA;NFU1;NHLRC2;NHLRC3;NHSL1;NIPBL;NLE1;NME4;NME6;NOA1;NOL6;NOLC1;NOM1;NOMO3;NOP14;NOP53;NPTN;NR2E1;NR6A1;NRAS;NRDC;NSA2;NSD1;NSMCE4A;NSUN2;NT5DC2;NUBP2;NUCB2;NUCKS1;NUDT19;NUP153;NUP155;NUP214;NUP35;NUSAP1;NVL;NXN;NXT2;OCLN;ONECUT2;OPA3;OPRL1;ORC4;OSBPL10;OSTM1;OTUB1;OXA1L;OXNAD1;OXR1;PA2G4;PAAF1;PABPC1;PAFAH1B3;PAFAH2;PAK1;PALD1;PAPOLA;PAPSS1;PARP16;PARVB;PAX3;PBX2;PCBP2;PCCB;PCGF3;PCYT1A;PCYT1B;PCYT2;PDCD10;PDCD11;PDCL3;PDE12;PDGFB;PDGFRA;PDHX;PDK1;PDLIM5;PDP2;PDPR;PDS5A;PDZD8;PEG10;PER1;PEX11B;PEX6;PFKM;PFN1;PGM1;PGM2L1;PGM3;PGRMC1;PHACTR4;PHKA1;PIGG;PIGU;PIH1D1;PITHD1;PLAGL2;PLCB3;PLCG2;PLD3;PLEKHA3;PLEKHO1;PLK1;PLXDC2;PLXNB2;PLXND1;PM20D2;PMAIP1;PMF1;PMPCA;POLD2;POLL;POLR1A;POLR1B;POLR2A;POLR2C;POLR2D;POLR2H;POLR2L;POLR3B;POLR3D;POLR3G;POM121;POM121C;POP1;POTEG;POTEM;PPARGC1A;PPID;PPM1G;PPP1R12C;PPP1R15B;PPP1R16B;PPP1R7;PPP2R2A;PPP2R5E;PPRC1;PRAF2;PRDM1;PRDM4;PRIM1;PRIM2;PRKAA2;PRKAR2A;PRKD1;PRPF19;PRPF8;PRPS1;PRR5-ARHGAP8;PRRC2A;PRRC2B;PRSS22;PRTG;PSD3;PSMD9;PSME3;PSMG4;PTCD1;PTGES2;PTGFRN;PTGS2;PTP4A2;PTPN23;PTTG1;PUM1;PUS1;PVR;PXDN;PYCR3;QARS;QDPR;QKI;QRSL1;RAB10;RAB11FIP4;RAB19;RAB38;RAB3GAP2;RAB40C;RABL2A;RABL2B;RAD18;RAI1;RALB;RALGAPB;RANBP6;RAP2C;RBBP6;RBFOX1;RBFOX2;RBM12;RBM12B;RBM19;RBM22;RBM34;RBPJ;RCAN1;RCC1L;RDH10;RDX;REEP4;REPS1;RFC2;RFFL;RHBDF2;RHD;RHOB;RHOG;RIOK2;RIOK3;RNASE10;RNF115;RNF144B;RNF40;RNF44;RNFT1;RNMT;RPAP1;RPIA;RPL12;RPL18;RPL18A;RPP38;RPS24;RPS4X;RPSA;RRAD;RRBP1;RRM1;RRM2;RRP1B;RRP7A;RRP8;RTCA;RWDD1;RXRB;SAFB;SALL2;SALL3;SAR1A;SC5D;SCAF4;SCAF8;SCAMP1;SCAMP3;SCD;SCML2;SCRN1;SCYL1;SDAD1;SDR42E1;SEC11A;SEC16A;SEC23B;SEMA4C;SEMG2;SEPT4;SERBP1;SETD5;SF3B1;SFMBT1;SH3GLB1;SH3PXD2A;SIGMAR1;SIKE1;SKI;SLC10A3;SLC10A7;SLC11A2;SLC12A7;SLC16A9;SLC19A3;SLC1A4;SLC20A1;SLC25A1;SLC25A12;SLC25A13;SLC25A19;SLC25A24;SLC25A32;SLC25A4;SLC27A2;SLC30A1;SLC30A7;SLC35F1;SLC35F6;SLC38A1;SLC38A2;SLC38A5;SLC38A7;SLC5A6;SLC9A3R1;SLF2;SMARCA1;SMARCA4;SMARCAD1;SMARCB1;SMARCC1;SMARCC2;SMARCD1;SMC1A;SMCR8;SMG1;SMG7;SNAP23;SNRPA;SNRPE;SNX12;SNX17;SOCS1;SOCS4;SOD2;SON;SOX13;SOX9;SP1;SP100;SPATA12;SPCS3;SPN;SPR;SPRYD4;SPRYD7;SPTBN2;SRCAP;SSR1;ST13;STAT2;STEAP3;STIM1;STIP1;STK38;STK4;STRN;STX3;SUMO1;SUMO2;SUOX;SUPT16H;SUPT20H;SURF4;SYNE1;SYNE2;SYNGR2;SYNJ2BP;SYT1;SZRD1;TAB2;TAF9;TAF9B;TBC1D13;TBC1D15;TBC1D19;TBC1D20;TBRG4;TCOF1;TENM3;TERF2IP;TES;TGFBR1;TGFBR3;TGOLN2;THBS1;THEM6;THOC5;THYN1;TIAF1;TIAM1;TIMM23;TIMM50;TIMM8A;TIMM9;TJP1;TLN1;TLNRD1;TLR4;TMED4;TMED5;TMEM115;TMEM167A;TMEM2;TMEM201;TMEM33;TMEM63B;TMEM65;TMTC3;TNFRSF10B;TNFSF12;TNFSF9;TNPO1;TNRC6B;TOE1;TOMM40L;TOR4A;TOX3;TPBG;TPD52L2;TPM4;TPP2;TPT1;TRABD;TRAPPC1;TRAPPC10;TRIM24;TRIM28;TRIM71;TRIP12;TRMO;TRMT1;TRNT1;TROVE2;TRUB2;TSC22D2;TSEN15;TSPAN3;TST;TTC9C;TTLL12;TUBA1B;TUBA1C;TUBB;TUBB2A;TUBB4A;TUBGCP2;TUBGCP3;TUT1;TXLNA;TXLNG;TXNL4A;TYMS;UBA1;UBA3;UBAP2L;UBE2A;UBE2D2;UBE2D3;UBE2I;UBE2M;UBE2Q1;UBXN2B;UBXN8;UCK1;UCK2;UGT8;UHRF1;UNC13B;UNC13D;USO1;USP10;USP14;USP15;USP22;USP38;USP47;USP54;UTP15;UTP6;UTRN;VAMP3;VCL;VIRMA;VPS28;VPS39;VPS41;VPS51;VWA8;WARS2;WASF1;WASL;WBP11;WDCP;WDR25;WDR26;WDR3;WDR33;WDR4;WDR55;WDR74;WDR75;WLS;WNK1;XPO5;XPO7;XRN1;XYLT2;YAE1D1;YAP1;YEATS2;YIPF3;YLPM1;YOD1;YTHDC1;YWHAE;YWHAZ;ZADH2;ZBTB37;ZBTB5;ZBTB8OS;ZC3H11A;ZC3HAV1;ZC3HAV1L;ZCCHC11;ZCCHC3;ZCCHC9;ZER1;ZFAND4;ZFHX4;ZMYM2;ZNF106;ZNF148;ZNF200;ZNF207;ZNF264;ZNF28;ZNF3;ZNF317;ZNF417;ZNF426;ZNF443;ZNF460;ZNF507;ZNF556;ZNF566;ZNF578;ZNF581;ZNF584;ZNF587;ZNF606;ZNF609;ZNF611;ZNF629;ZNF644;ZNF652;ZNF687;ZNF738;ZNF774;ZNF799;ZNF8;ZNF805;ZNF841
hsa-miR-382-5p-478078_mir	0.235	0.779	ADM;ADO;APCDD1;ARL4A;ARMCX3;ATG10;ATXN1;ATXN3;B4GALT7;BBS4;CALM1;CASP3;CCND2;CLTC;COPS4;CTTN;DAD1;DDOST;DGAT2L6;DICER1;DRD1;DSN1;DYNC1H1;DYRK2;EIF1AX;EXOSC2;FAM105A;FAM199X;FAM46A;FNIP1;FRAXA;G3BP2;GNL3L;GPR176;GSKIP;HERC2;HIST1H3F;HIST1H3G;KIF5C;KLHDC10;KLHL3;LUZP6;MRPS18C;MSANTD3;MTCL1;MTHFD2;MTPN;MTRNR2L1;MTRNR2L2;MTRNR2L8;MXD1;NETO2;NEXMIF;NFIA;NHLRC3;NHS;NYAP2;PARM1;PCBP1;PCNX2;PFKFB2;PLEKHA8;PLSCR4;PNMA2;PNPO;PPM1A;PRNP;PTEN;PTGDR;PTP4A2;RAB6A;RAPH1;RBM39;RPAP2;RPLP0;SAP30;SAR1B;SCAMP4;SERGEF;SF3B1;SLC10A7;SLC25A46;SLC3A1;SLC6A8;SMC3;SMURF2;SPIC;STT3A;SYNJ2;SYT13;TBL1XR1;T

			M4SF1;TMEM209;TRPV2;TSPYL1;UBB;UBBP4;UFL1;VAMP3;VEZF1;XPO1;XPR1;YAF2;YBX1;ZCCHC14;ZFP36L1;ZKSCAN5;ZNF264;ZNF318;ZNF652;ZNF860
hsa-miR-210-3p-477970_mir	-1.247	0.787	ABCB9;ACTR1A;ACVR1B;AIFM3;ALDH5A1;APC;ARHGAP35;ASB11;ATG7;ATP11C;BDNF;BNIP3;BTK;CASP8AP2;CBX1;CDK10;CHD9;CLASP2;CNTNAP5;COL4A2;CPEB2;CSNK1E;DDAH1;DEAF1;DENND6A;DIMT1;DOCK7;DYRK2;E2F3;EFNA3;EHD2;ELK3;ERP27;ESCO2;ESPL1;FCHSD2;FGFRL1;FOXN3;FOXP3;GIT2;GPD1L;GTDC1;HECTD1;HIF1A;HIF3A;HMGCS1;HOXA1;HOXA3;HOXA9;HSD17B1;ICMT;IGFBP3;INPP5A;INSIG1;ISCU;KCMF1;KCNB1;KCNJ6;KCNK10;LDHA;LDHB;MAF;MCM3;MDGA1;MEF2D;MGRN1;MIB1;MID1IP1;MITF;MNT;MRE11;NCAM1;NDUFA4;NFIC;NIPBL;NPTX1;P4HB;PFDN2;PIM1;PKIA;PLK1;POU2AF1;PPP1R2;PSAP;PTAR1;PTBP3;PTPN1;PTPN2;RAD52;RCC2;RPL22;RUNX1T1;SCN1B;SDHD;SEH1L;SERTAD2;SERTM1;SH3BGRL;SIN3A;SIPA1L3;SLCO3A1;SMCHD1;SSX2;SSX2B;STMN1;TBC1D16;TFRC;THSD7A;TNPO1;TNPO3;TNRC6B;TP53I11;TWIST1;U2AF2;UBA1;UBQLN1;VAMP4;VMP1;XIST;XPA;ZNF607;ZNF618
hsa-miR-323a-3p-477853_mir	-0.320	0.805	AADAC;ACVR1C;AGO3;BZW1;CAMK1D;CANX;CBR1;CDKN1B;CDKN2AIP;CHEK1;CLCC1;CNBP;CNEP1R1;DEPDC1;ENPP5;EREG;F5;FAM20B;FNDC3A;GALNT1;GPR50;GPSM2;HMGXB4;HNRNPA1;IFFO2;INO80D;KCTD10;KIAA2026;LHFPL6;LIN52;MAP3K1;METTL15;MYC;MYLIP;NLGN4X;NUP205;P2RY11;PDZD8;PELP1;PGM2L1;PHC3;PLA2G7;PLAG1;PMAIP1;PNISR;PPP1CB;PPP1CC;PRDM4;PRKAR1A;RAB3GAP2;RAP2C;RMDN1;SDE2;SEMA7A;SESN3;SLC17A5;SLC35C2;SMAD2;SMAD3;SOCS5;STAT3;STMN1;TCP1;TNFRSF10B;TRPS1;TRUB1;TSNAX;TSPAN3;TXNIP;UNC5C;UTP18;WASL;WDR45B;ZNF566;ZXDA
hsa-miR-486-5p-478128_mir	0.096	0.835	ABCF2;ARF6;ARHGAP5;BAG2;BASP1;BTF3L4;CADM1;CCDC14;CD40;CDK4;CENPN;CIT;CLDN10;CMSS1;CRIPT;DCTN4;DENND5B;DOCK3;EPGN;FAM217B;FAM46A;FBN1;FOXO1;FOXP1;FPR1;G3BP2;H3F3B;HAT1;HMGA1;HPGD;ID4;IGF1R;IGSF3;LTBP2;MACROD2;MARCH4;MBD4;METTL27;NELL2;OLFM4;PCCA;PIK3R1;PIM1;PTEN;RBM12B;RBM22;RCOR3;SAPCD2;SEC23IP;SEL1L3;SERPINE1;SMAD2;SNAI1;SP4;SPRTN;TM4SF20;TMED1;TTC8;UBASH3B;UBE2S;UTP4;YAE1D1;ZDHHC20;ZIC5;ZNF286B;ZNF460;ZNRF2
hsa-miR-15a-5p-477858_mir	-0.111	0.847	ABCC6;ABHD2;ABL2;ACOX1;ACTR1A;ACTR2;ACTR3B;ACVR1B;ACVR2A;ADORA3;ADRA2B;AFF4;AGO2;AGO4;AHNAK2;AKAP11;AKR1B10;AKT3;ALDH3B1;AMER1;AMOT;AMOTL1;ANAPC13;ANAPC16;ANKMY1;ANKRD13B;ANKRD36;AP2B1;AP3M1;AP5Z1;APP;ARCN1;ARHGAP12;ARHGAP32;ARHGDIA;ARIH1;ARMC12;ASCC1;ASGR2;ASH1L;ASNSD1;ASXL1;ASXL2;ATAD5;ATF2;ATG14;ATG9A;ATP13A3;ATP5G3;ATP6AP1;ATXN7L3B;AURKAIP1;AVL9;AXIN2;B3GNT2;B4GALT1;BACE1;BAG4;BAZ2A;BCL2;BCL2L12;BCL7A;BDNF;BHLHE40;BMI1;BRCA1;BRWD1;BSG;BSPRY;BTG2;BTN3A3;BTRC;BZW1;C11ORF24;C15ORF39;C16ORF58;C16ORF72;C17ORF80;C1ORF21;C1ORF226;C21ORF62;C2ORF42;C2ORF74;C3ORF36;C6ORF106;CA8;CABIN1;CACUL1;CADM1;CALD1;CALU;CAMSAP1;CANX;CAPZA2;CARD10;CARD8;CARM1;CASK;CASKIN1;CBFA2T3;CBX2;CBX4;CBX6;CCDC80;CCDC83;CCDC88C;CCND1;CCND2;CCNE1;CCNE2;CCNT1;CCNT2;CCNYL1;CCT6B;CD180;CD274;CD2AP;CDADC1;CDC14B;CDC25A;CDC27;CDC37L1;CDC42SE2;CDCA4;CDK1;CDK17;CDK6;CDKN1A;CDKN2AIPNL;CDKN2B;CDS2;CDV3;CENPJ;CEP55;CEP63;CHAC1;CHD4;CHEK1;CHIC1;CHMP3;CHMP4B;CHUK;CLCC1;CLCN3;CLEC2D;CLIP4;CLSPN;CLU;CLUH;CMTM4;CNKSR3;CNN3;CPEB2;CPEB3;CPNE1;CPSF7;CREBL2;CREBRF;CREG1;CRIM1;CRK;CRKL;CSDE1;CSNK1E;CTDSPL;CUL2;CUL3;CXCL10;CYB561A3;CYLD;CYP26B1;DCAF17;DCTN5;DDX3X;DDX3Y;DECR1;DENND6A;DIAPH1;DICER1;DLGAP3;DLK1;DMPK;DMRT2;DMTF1;DNAJA1;DNAJC10;DNAJC9;DOCK11;DPP8;DSCR3;DSTYK;DYNLL2;DYRK3;E2F3;E2F7;ECHDC1;EDC3;EFNB2;EFTUD2;EIF1AX;EIF2B2;ELK4;EN2;ENTPD1;ENTPD6;ENTPD7;EPM2AIP1;ETFRF1;ETNK1;EXT1;EZH1;FAM103A1;FAM122B;FAM122C;FAM229B;FAM69A;FASN;FBXL18;FBXL20;FBXO3;FCF1;FGF2;FGF7;FGFR4;FKBP1A;FLCN;FLOT2;FOXK1;FOXO1;FRYL;FURIN;FZD6;FZD9;GABARAP;GABARAPL1;GABPA;GALNT1;GANAB;GATAD2A;GCLM;GDI2;GGA3;GLP2R;GNAL;GNAT1;GNB1;GNG12;GOLGA5;GOLPH3L;GOSR1;GPATCH8;GPR180;GPR27;GPRC5A;GRAMD2B;GRB2;GSG1;GSK3B;GTF2H1;H3F3B;HACE1;HAUS3;HCFC2;HDGF;HDHD2;HERC6;HEYL;HIGD1A;HIST1H2BK;HIST2H2BE;HMBOX1;HMGA1;HMGA2;HNRNPA1;HNRNPA1L2;HNRNPA2B1;HNRNPDL;HOXA10;HOXA3;HOXC8;HPF1;HSDL2;HSP90B1;HSPA1A;HSPA1B;HSPA4L;HSPA8;HSPE1-MOB4;HYOU1;IER2;IFNG;IFT74;IKBKG;IL10RA;INSL6;IPPK;IRAK1BP1;IRF4;ITGA2;ITPR1;IVNS1ABP;JARID2;JPT2;JUN;KANK1;KATNAL1;KIAA0895;KIAA1456;KIF1A;KIF23;KIF3B;KIF5B;KLC2;KLF4;KLF6;KLHDC10;KLHL15;KLHL40;KMT2D;KPNA1;KPNA3;KRT33B;L2HGDH;LAMC1;LAMP2;LANCL1;LDAH;LITAF;LRIF1;LRIG2;LRPPRC;LRRC57;LRRFIP2;LSM11;LUC7L3;LURAP1L;LUZP1;MAFK;MAP2K3;MAP3K7;MAP4K2;MAPK6;MAPKAPK2;MBD4;MCFD2;MCL1;MCM3AP-AS1;MED11;MIB1;MIGA1;MINK1;MKX;MLLT6;MLXIP;MN1;MOB4;MRPL40;MSANTD4;MSH2;MSL1;MTFR1L;MTHFR;MTMR3;MTMR4;MYB;MYBL1;MYO5A;N4BP1;NAA25;NAB1;NAPG;NCKAP1;NCOR2;NEGR1;NFIC;NFKB1;NIPAL2;NNT;NOL4L;NOP2;NOTCH2;NR2C2;NR6A1;NT5DC1;NUCKS1;NUFIP2;NUP50;OCRL;ODC1;ODF2L;OGT;OMA1;ORC4;OSBPL3;OSCAR;OSGEPL1;OTUB1;PAFAH1B1;PAFAH1B2;PAG1;PAGR1;PAK2;PANK1;PCF11;PCMT1;PDCD1;PDCD4;PDCD6IP;PDE4D;PDHX;PDIA6;P

			DIK1L;PEX12;PEX13;PGD;PHC3;PHF19;PHKA1;PHKB;PHLPP1;PHLPP2;PHYHIP;PI4K2B;PIK3R1;PIM1;PISD;PLA2G2D;PLAG1;PLEKHA1;PLEKHB2;PLPBP;PLPP3;PLRG1;PMS1;PNISR;PNN;PNPLA6;PNPO;PNRC2;POLDIP3;POLE4;POLR2E;POM121C;POU2AF1;PPIG;PPIL1;PPIP5K2;PPM1A;PPP1R11;PPP2R5C;PPP6C;PPP6R3;PRDM4;PRDX3;PREB;PRICKLE2;PRIM1;PRIMPOL;PRKAA1;PRKAR2A;PRKCD;PRRC2C;PRSS21;PSAT1;PSKH1;PSMB5;PSMC4;PTAR1;PTPRD;PTPRJ;PURA;PWWP2A;RAB11FIP2;RAB15;RAB21;RAB23;RAB3IP;RAB40B;RAB9B;RACGAP1;RAD23B;RAD51C;RALGAPB;RAP2C;RAPH1;RARB;RASEF;RASSF2;RASSF5;RBBP6;RBPJ;RCAN3;RCOR1;RECK;REL;RELT;REPIN1;RET;REXO1;RFK;RFWD2;RGPD5;RHOT1;RHOV;RIDA;RIMS3;RNASEH1P1;RNASEL;RNF138;RNF149;RNF168;RNF38;RNMT;RNPS1;RPL14;RPL36;RPP30;RPRD1B;RPRD2;RPS5;RPS6KA3;RPS6KB1;RS1;RTN4;RUBCN;RUNX1T1;SALL1;SBNO1;SCAMP4;SCAMP5;SEC24A;SEC61A1;SEC63;SELENOI;SEPT2;SERBP1;SESTD1;SETD1B;SH3BP4;SHOC2;SIDT2;SIK1;SIRT4;SKAP2;SKI;SLC25A12;SLC25A22;SLC25A29;SLC29A1;SLC2A3;SLC35A1;SLC35B3;SLC35E2B;SLC39A9;SLC7A5;SLC9A1;SLC9A6;SLCO3A1;SMAD3;SMAD7;SMDT1;SMURF1;SNCG;SNRPB2;SNTB2;SNX11;SNX16;SNX6;SOCS5;SOWAHC;SOX5;SPRED1;SPTLC1;SREK1;SRPK1;SRPRA;SRPRB;SRSF1;SSRP1;SSU72;STK38;STRADB;STX17;STXBP3;SUN1;SUPT16H;SYNRG;SYPL1;SZRD1;TADA2B;TAF13;TAOK1;TARBP2;TASP1;TBC1D14;TBC1D20;TBCCD1;TBL1XR1;TBPL1;TBRG1;TCF3;TECPR2;TET3;TFAP2A;TFB1M;TGFBR3;THRAP3;TIA1;TIMM13;TKTL1;TLE4;TLK1;TLL1;TM4SF1;TM7SF3;TM9SF2;TMEM100;TMEM109;TMEM135;TMEM138;TMEM161B;TMEM184B;TMEM189;TMEM189-UBE2V1;TMEM214;TMEM245;TMEM251;TMEM69;TNFAIP1;TNFSF9;TNRC6B;TOB2;TP53;TPI1;TPM2;TPM3;TRAK1;TRAM1;TRIM28;TRIM35;TRMT13;TSC22D2;TSPYL2;TTC1;TTLL5;TUBB;TUBB2A;TXNIP;U2SURP;UBE2H;UBE2Q1;UBE2Q2;UBE2V1;UBE3C;UBE4A;UBN2;UBR3;UCP2;UGDH;UGP2;UGT2B4;USP15;USP3;USP31;USP42;USP48;USP53;VAV2;VCL;VEGFA;VOPP1;VPS33B;VPS45;VPS4A;VSIR;WDR13;WEE1;WIPF1;WIPI2;WNK3;WNT3A;WT1;XKR7;YAP1;YIPF6;YRDC;YTHDC1;YWHAH;YWHAQ;ZBTB10;ZBTB16;ZBTB33;ZBTB34;ZBTB5;ZCCHC3;ZDHHC16;ZFHX4;ZFP28;ZMAT3;ZNF267;ZNF275;ZNF284;ZNF367;ZNF391;ZNF449;ZNF460;ZNF559;ZNF585B;ZNF620;ZNF622;ZNF691;ZNF704;ZNF91;ZNRF1;ZNRF2;ZNRF3
hsa-miR-495-3p-478136_mir	0.146	0.849	ABCB1;ACBD7;ACTA1;ACTC1;ADAMTS9;AGO2;AKT1;ALPK3;ANKRD40;AREL1;ARL10;ARPP19;ATP7A;B4GALT1;BMI1;BTF3L4;C16ORF52;C4ORF46;C5ORF24;C6ORF106;CALHM5;CASP16P;CASP8;CBX4;CCDC141;CCL2;CD164;CD9;CDC5L;CDC73;CDCA2;CDK1;CEP19;CNBP;COIL;COL4A1;COPS7B;CRLF3;CRLS1;CXCL5;DDIT4;DGKB;DLC1;DNAJC21;EEF2K;EIF5A2;EIF5AL1;EMB;FGF2;FNDC3B;FOXC1;GALNT8;GEMIN6;HEXIM1;HMBOX1;HMGA2;HMGN1;HNRNPC;HOXC8;HOXD12;HSP90AA1;HSPA1B;HSPA5;IL15;IL6R;INHBA;KDM5B;KIAA1549L;KLHL15;KPNA2;LDLR;LMAN1;LRP6;LRRC4;LYPLA1;MARCKS;MAT1A;MCL1;MED13;MEIS1;MFAP3;MIEF1;MLEC;MLLT10;MOCS2;MPRIP;MRPL35;MRRF;MSANTD3-TMEFF1;MTA3;MTAP;MTMR9;MTRNR2L1;MTRNR2L10;MTRNR2L11;MTRNR2L3;MTRNR2L7;MYADM;MYO10;NCAM2;NCK2;NDUFB6;NGDN;NHLRC3;NPM3;NUP62;OCRL;PBX3;PCDH1;PCMT1;PCNX1;PER2;PFDN2;PHF13;PHLPP1;PNISR;PRNP;PRRT2;PTP4A3;QSER1;RAB10;RAB31;RFK;RNF138;RNF141;RNF41;RUNX3;SAMD8;SC5D;SCIMP;SCO1;SDC2;SF1;SFT2D2;SIGLEC14;SIX3;SLC25A46;SMOC1;SMR3B;SNX24;SOX9;SPNS1;SREBF1;SREK1IP1;STX4;TAB2;TBC1D9;TGFBR2;TGIF1;TIMM10;TMEFF1;TMEM170B;TMEM2;TMEM68;TNPO1;TNPO2;TOP2A;TOR1AIP1;TP53INP1;TRIM37;TRIM67;TTYH3;TXNIP;UBE2Z;UHMK1;UTP4;VEGFA;VGLL4;WASL;WTIP;XRCC2;ZBTB18;ZBTB37;ZBTB47;ZBTB8B;ZNF324B;ZNF354C;ZNF431;ZNF460;ZNF573;ZNF703;ZNF724;ZNF740;ZZZ3
hsa-miR-485-3p-478125_mir	-0.182	0.852	ABHD15;AGO3;ANKRD33B;ANKRD62;ARID1A;C11ORF74;CLTC;DST;EAF1;FASLG;FLNB;FOCAD;G6PC;GAS1;GBF1;GID4;GRHL1;HNRNPC;HOMER2;HOXA13;HS3ST3B1;IDS;IFNLR1;IL5RA;INTS3;KANSL1;KPNA2;LINC00598;LNPEP;MAML3;MAT1A;MGAT5;MKLN1;MRI1;MTF2;MTRNR2L2;MTRNR2L8;NAPEPLD;NBEA;NFE2L1;NFYB;NGDN;NIPAL2;NTRK3;PANK3;PBRM1;PCGF3;PJA2;PPARGC1A;QSOX2;RAD50;RCAN2;RORA;RPS4X;RPS6KA5;RREB1;RTL6;RTP4;SETX;SKIL;SLC40A1;SMIM13;SRCAP;SRSF2;SS18;TAS2R30;TCF7L2;TESK2;TMED10;UCHL5;WDR17;WSCD2;YWHAG
hsa-miR-576-3p-478164_mir	-0.152	0.859	AASDHPPT;AGFG1;AIRE;AMD1;AP1G1;ARID5B;ATXN1;BCL7B;BLOC1S6;C12ORF65;C5ORF51;CAPZA2;CCDC6;CCND1;CHCHD5;DDTL;DLC1;DYNLL2;EMB;ERGIC2;FAM102A;FAM102B;FUT9;GEMIN2;GNG5;GSPT1;HIF1A;HIGD2A;HINFP;HIST1H2BD;KCNK6;KLHDC10;KPNA5;LETMD1;LIN28B;LRIF1;LRRC58;MAML3;MAN2A1;MNT;MRPS27;MTHFD2;MYH2;MYLK3;NAXD;NFIC;NQO2;OCLN;ORAI2;PGAM4;PHF12;PMAIP1;POLK;POLR3F;PPP2R5E;PQLC2;PRSS8;RABGAP1L;RACGAP1;RBM23;RBM27;RNF217;RSL1D1;SAMD8;SCARA3;SDK2;SESN3;SGK1;SH2B3;SLC2A14;SNRPD1;SOX4;SRSF11;STON2;STOX2;TAF1D;TNFRSF11A;TRAM1;TRPC3;TSR1;UGCG;XPO6;YTHDF1;YWHAQ;ZBTB18;ZFP91;ZNF101;ZNF24;ZNF543;ZNF566;ZNF708
hsa-miR-423-5p-478090_mir	0.049	0.921	AAR2;ABCC5;ABL1;ACTG1;ADAMTS14;ADIRF;AHDC1;AJAP1;AK2;AKAP13;ALDOC;ANKRD13B;ANKRD42;ANKRD52;AP2S1;AP3M1;APEX1;APOBEC3C;ARHGAP32;ARHGDIA;ARHGEF2;ARID5B;ARL6IP1;ARL8A;ARPC1A;ARRB2;ASCL2;ASPH;ATG9A;ATN1;ATP2B4;ATP5B;ATP6V1E1;B4GALNT4;BAK1;BANP;BARHL1;BCCIP;BCL

			AF1;BICDL1;BRSK1;C10ORF55;C12ORF10;C15ORF39;C20ORF27;C22ORF39;C3ORF36;C6ORF106;CALM3;CAPN15;CASZ1;CBX6;CBX8;CCDC106;CCND2;CCNF;CCNI;CD81;CDK15;CDKN2A;CEACAM6;CEBPB;CHD8;CHTF8;CLCN7;CLSPN;CLSTN1;COPS7A;COPZ1;COQ6;CREB1;CSDE1;CSTB;CTDNEP1;DAG1;DDX21;DDX24;DDX54;DIP2A;DLX5;DRAP1;E2F3;EFHD2;EIF1;EIF5AL1;EN1;EPB41;EPM2AIP1;EPN1;ERRFI1;EVI5L;FA2H;FAM110B;FAM129B;FAM189B;FAM3A;FAM83H;FAM98A;FHOD1;FKBP4;FOXK1;GAPDH;GDE1;GDF11;GGA3;GJB1;GLUL;GNG12;GPAT4;GPRIN1;GRID1;GTF2F1;GTPBP1;HDGF;HES4;HIPK2;HIST1H1C;HIST1H1E;HIST1H2BK;HIST2H2BE;HIST2H2BF;HLA-DOA;HNRNPUL1;HOXA7;IGDCC4;ITPRIPL2;KCNK3;KCTD15;KCTD2;KDM6B;KIF1A;KIF3B;KLHL38;KMT2A;KMT2B;KMT5A;LARP1;LASP1;LIMS2;LMNB2;LRCH4;MAP3K9;MAPK8IP3;MCM5;MCM7;MCRIP1;MDM4;MED28;MEX3A;MFGE8;MICALL1;MIDN;MIER2;MINK1;MKNK2;MLF2;MLN;MMP17;MNT;MRPL34;MSANTD3;MT-ATP6;MT-CO1;MT-ND5;MTA1;MTCH2;MTMR3;MTRF1L;MUL1;MYBL2;MYC;NACC1;NANOS1;NAV1;NCEH1;NCS1;NDE1;NDRG1;NECAP1;NECTIN1;NEDD4L;NF2;NFASC;NFIX;NFKB2;NID1;NKX6-2;NMD3;NME2;NOLC1;NOS1AP;NPLOC4;NPTXR;NSD1;NUTM1;OLR1;PA2G4;PABPC1;PAK1;PAX2;PBX2;PCBP1;PCP4L1;PDP2;PDPK1;PEA15;PFKFB3;PGAM1;PGAM4;PHACTR4;PKM;PLAGL2;PLCB1;PLXND1;PMPCA;PNMA8B;PNRC1;POLL;POLR3H;PPARGC1B;PPIA;PPIAL4G;PPIB;PPP1R11;PRPF38B;PRRC2A;PRRC2B;PSMD11;PSMD6;PTMA;PTTG1;RABAC1;RAD18;RBPJL;RC3H1;RCE1;REEP3;REPS1;RGPD2;RIN3;RNF139;RNF165;RNF187;RPL10;RPL18A;RPL23A;RPL7L1;RPS10;RPS15A;RPS2;RPS6KA4;RPS6KL1;RPS9;RRM2;RTL8C;RUNX1;SALL1;SAR1B;SBK1;SCAMP2;SEC22C;SELENON;SEPT8;SEPT9;SF3B3;SHANK3;SHMT1;SLC2A5;SLC39A11;SLC48A1;SLC5A6;SLC6A6;SLC7A2;SLC9A3R1;SLITRK5;SNX8;SOX12;SP1;SPATA2;SPEF1;SPNS1;SPRY4;SQSTM1;SRM;SRP54;SRSF11;STAT5B;STIM1;STRIP2;STRN4;SYNGR1;SYNGR2;TAB2;TACR3;TAGLN;TDRKH;TEX2;TFF1;TIMP3;TLNRD1;TLR5;TMBIM6;TMEM11;TMEM127;TMEM184B;TMUB1;TPGS2;TPST2;TRAF7;TRIM28;TRIM41;TSPYL1;TSPYL6;TTLL12;TUBB;TXNIP;U2AF2;UBE2D3;UBE2L3;UBFD1;ULK1;UNK;URM1;USP6;UST;VAC14;WASF2;WBP2;WDR45B;WFIKKN2;WIPF2;YARS;YWHAE;YWHAZ;ZBTB34;ZBTB46;ZFP36L1;ZMAT5;ZNF609;ZNF655;ZNF787;ZNF805;ZYX
hsa-miR-629-5p-478183_mir	0.244	0.932	AAK1;AGAP1;AKTIP;ANO8;ATP5G2;CARD8;CFLAR;CMBL;CSNK1E;DHFR2;DMXL1;DYRK2;EIF1AD;ELF3;ERAP2;ERGIC2;GPR107;GPR82;HIST1H1C;HIST1H2AC;HIST1H2BB;HIST1H2BE;HIST1H2BH;HIST1H3B;HIST1H3D;HNF4A;HOXB13;HOXC8;IRF2BP2;MIEF1;MTRF1L;NCDN;NFYA;ONECUT3;OR2A4;PAPD7;PARD3;PERP;PTPN14;PTPRB;RAB12;RAB3B;RAB3IP;RASSF8;RIOK3;RSRC2;RTKN;SDCBP;SGCD;SIX1;SYNGAP1;TFDP1;TNFRSF10D;TP53INP2;TRIM33;TXNIP;ZBTB18;ZBTB47;ZBTB7A;ZCCHC6;ZEB1;ZNF175
hsa-miR-191-5p-477952_mir	0.060	0.940	AACS;ACSL1;ACSS1;AGL;AMMECR1L;ATF6;BASP1;BCOR;BICD2;BRAF;BZW2;CASTOR2;CCDC88C;CCND2;CDK6;CDK9;CEBPB;CTDSP2;CYTH2;DCAF15;DCUN1D2;DHCR24;EGR1;EIF3I;EMX2;ENAH;FN3KRP;GBA;GOLGA7;HNRNPA2B1;HNRNPM;HNRNPUL1;HSD17B12;IL1A;IRS4;KNL1;LPP;LRRC8A;MCFD2;MDM4;MFAP3;MPST;MTSS1L;NDST1;NOTCH2;NPLOC4;PDHA1;PI4KB;PISD;PPIAL4G;PPP1R14C;PSAP;PSMA2;PURB;RABL6;RAD21;RCC2;RGS2;RPIA;RPL37;RPS6KA3;RRN3;SATB1;SLC16A2;SLC7A1;SOCS4;SOX4;SPATA2;SRGAP2;TARS;TJAP1;TLE1;TMC7;TMEM33;TNPO2;UHRF1;USP37;YBX3
hsa-miR-221-3p-477981_mir	-0.029	0.961	ABHD3;ABLIM1;ACIN1;ACSL3;ACTB;ACTG1;ACVR2B;ADAM1A;ADAMTS6;ADD1;ADGRL2;AGAP1;AGO2;AKT3;AMMECR1L;AMOT;ANKRD28;ANXA1;AP2A1;AP3B1;APAF1;APOL2;ARF4;ARHGAP42;ARHGEF18;ARID1A;ARIH2;ARL6IP1;ARNT;ASXL2;ASXL3;ASZ1;ATL2;ATP2A2;ATP6V1E1;ATXN1;B3GALNT2;B4GALT2;BAG3;BBC3;BCL2L11;BECN1;BMF;BNIP3;BNIP3L;BRAP;BRD1;BRWD1;C15ORF40;C19ORF12;C5ORF51;CABYR;CAMKK1;CASP3;CCDC142;CCSAP;CCT3;CDC25C;CDC27;CDK6;CDKN1B;CDKN1C;CENPT;CERS2;CHCHD2;CHORDC1;CHSY1;CIAPIN1;CLIC1;CNOT1;CNRIP1;COG5;CORO1A;CPSF6;CREBZF;CROT;CSTF2T;CTCF;CTNNB1;CXCL12;CXORF38;CYP1B1;DDAH1;DDIT4;DDX3Y;DDX6;DENR;DERL2;DHX15;DICER1;DIRAS3;DKK2;DNAJB14;DVL2;DYNC1H1;DYRK3;E2F3;EEF1A1;EIF2AK1;EIF2D;EIF2S3;EIF4G3;ELAVL2;ERBB4;ERC1;ESR1;ETS1;EVL;EXO5;EXOC8;FAM35A;FAM84A;FARSA;FBN3;FBXL18;FBXO28;FICD;FLNA;FMR1;FOS;FOXO3;FSCN1;FUS;GALNT3;GATAD2B;GCN1;GID8;GJA1;GLYR1;GPR107;GRB10;GTF2E1;HECTD1;HECTD2;HIST1H2AC;HIST1H2AE;HIST1H3D;HIST2H2AC;HIST2H3D;HIVEP1;HMBOX1;HMGXB4;HNRNPA0;HNRNPD;HOXB5;HOXC10;HSPA1B;ICAM1;IQCE;KHSRP;KIF16B;KIT;KLF9;KLHDC10;KLHL8;KPNA2;KPNA6;LAMTOR5;LDHB;LHFPL2;LIMS1;LRP6;LYN;LYSMD1;MAP3K2;MAPK10;MAT2A;MBD2;MBNL1;MDFIC;MDM2;MEOX2;MFN2;MGMT;MIDN;MIEN1;MKI67;MKKS;MMP2;MTSS1L;MYBL1;MYLIP;NABP2;NAIP;NCL;NDFIP1;NDUFB5;NDUFS1;NFYA;NFYC;NHSL1;NME2;NOP58;NR2C2AP;NT5DC2;NUF2;NUFIP2;NUP205;NUP210;OIP5;PAFAH1B2;PAIP2;PAK1;PALB2;PANK3;PCDHA1;PCDHA10;PCDHA11;PCDHA12;PCDHA13;PCDHA2;PCDHA3;PCDHA4;PCDHA5;PCDHA6;PCDHA7;PCDHA8;PCDHAC1;PCDHAC2;PDGFA;PDIK1L;PEG10;PELO;PEX1;PEX19;PGPEP1;PHF12;PHF21A;PIK3R1;PITPNM1;PKM;PLOD2;PLP2;PNRC2;POGZ;PO

			LG;POU3F2;PPP1R14C;PPP1R15B;PPP2R2A;PPP6C;PRDM16;PRPS1L1;PSMB5;PSMD4;PTBP3;PTEN;PTPRF;PXN;RAB1A;RAB5C;RACGAP1;RAD51;RB1;RBM33;RBM39;RBM6;RBMS2;RECK;RHOA;RNF20;RNF4;RNF44;RNPS1;RPL15;RPL21;RPLP0;RPS24;RPS7;RUNDC3B;RUNX1;SAPCD2;SELE;SEPHS1;SERPINH1;SF1;SF3B3;SGTA;SIRT1;SKI;SLC10A7;SLC19A3;SLC25A36;SLC30A7;SLC6A9;SMC2;SMCHD1;SNX4;SOCS1;SOCS3;SOX11;SPAG5;SPRYD3;SPTSSA;SRP68;SRSF2;SSX2IP;STAMBP;STAT5A;STMN1;STN1;TBK1;TCEAL1;TDRP;TFAP2A;TIAM1;TICAM1;TIMP3;TIPARP;TLE4;TMCC1;TMED7;TMEM132B;TMEM168;TMEM183A;TMEM2;TMEM245;TMEM248;TMEM64;TNFSF10;TNIP1;TNKS2;TOB2;TOMM20;TP53;TRAF4;TRAT1;TRIM28;TRPC3;TRPS1;TSC22D2;TSN;TSPAN13;TUB;TUBA1C;UBC;UBE2J1;UBE2N;UBN2;UHRF1;UNC13B;UQCR10;USP18;USP28;UTP14A;VANGL1;VPS53;WDR34;WDR61;WEE1;XRCC6;YOD1;YWHAB;YWHAE;YY1;ZBTB37;ZBTB5;ZEB2;ZFP1;ZFP30;ZKSCAN8;ZNF236;ZNF275;ZNF35;ZNF571;ZNF652;ZNF805;ZYX
hsa-miR-483-5p-478432_mir	0.223	0.966	ABHD15;AKAP6;ALCAM;ALG10B;API5;APOBEC3F;ARGFX;ART4;ATP5G1;BACE2;BARHL1;BORCS7;C19ORF47;C9ORF64;CACNG8;CAPZB;CBS;CCS;CKB;CLSPN;DENND6A;DPP8;EMC8;ETV7;FAHD1;FAM160B2;FAM83H;FLYWCH2;FN3K;FOXC1;FOXJ2;G3BP1;GDE1;GDF5OS;GFRA1;GMEB1;GNE;GPC6;GPR156;GRK3;GUCA1B;HAND2;HEATR5A;HIC2;HINFP;IFNAR1;IGDCC3;IL21R;ILDR1;IPO9;IPP;KANK2;KIAA1456;KIAA1549;KIR3DX1;KREMEN1;LARS;LAYN;LMOD3;LYPLA1;MACC1;MAP4K2;MAPK3;MAPKAPK2;MAVS;MCRIP2;MINDY1;MIOX;MLLT1;MLLT6;MLXIP;MUC3A;NAV1;NCBP2;NDUFV3;NEK8;NFIX;NOTCH3;NTMT1;NUCB1;ORMDL2;P4HB;PCSK9;PDGFRA;PEX26;PGAM1;PGAM4;PHF20;PLEKHG2;PLIN3;POLR3A;PPDPF;PRKN;PRRT2;PSPH;PTMA;PTPN14;PURB;RASSF9;RHOA;RHOBTB3;RNF19B;RNF20;RRP36;RSBN1L;SLC26A2;SLC35B2;SMARCD1;SPON2;SPPL3;SRF;SRSF10;STX6;SUSD1;SYT7;TAOK1;THAP1;TIMM22;TIMM29;TNFAIP8;TRAF1;TRAT1;TROVE2;UTP6;VHL;WDPCP;XPC;YIPF4;ZFAND4;ZMYM1;ZNF101;ZNF276;ZNF384;ZNF556;ZNF662;ZNF677;ZNF70;ZNF708;ZNF747;ZNF793;ZNF813;ZYG11A;ZYG11B
hsa-miR-22-3p-477985_mir	-0.007	0.989	ACLY;ACVR1C;AKT1;ALMS1;ARHGEF26;ARID5B;ARPC5;BDNF;BMP6;BMP7;BMPR1B;BRWD3;BSG;BTF3;BTG1;BTN3A3;BUB1B;C15ORF40;C1ORF87;C5ORF24;CAMK2N1;CCNA2;CCNT2;CD151;CDK6;CDKN1A;CHD9;CLPTM1L;CSF1R;CSNK2A1;CTC1;CXCR2;CYCS;CYR61;DAD1;DCAF16;DDIT4;DDX6;DNHD1;E2F2;EDC3;EFR3B;ELP5;ERBB2;ERBB3;ESR1;FKBP5;FOXK1;FOXP1;FRAT2;FUBP1;GINS2;GLIS2;GRB2;H3F3B;HDAC4;HDAC6;HIF1A;HMGB1;HNRNPA3;HSPA1B;HTR2C;IBA57;IFT140;INSIG1;IRF5;KCTD10;KCTD12;LBP;LEMD3;LGALS1;LGALS9;LIN7C;LONP2;LRRC1;LRRC20;MALAT1;MAOA;MAX;MECOM;MIS18BP1;MMP14;MTA1;MTDH;MTHFR;MYCBP;MYO6;NCOA1;NET1;NR3C1;NTRK2;NUP214;PDHA1;PDIK1L;PEX5;PHACTR4;PIGP;PIK3C2A;PLK1;PPARA;PPM1K;PRELID2;PRKACA;PTEN;PTMS;RAB44;RAB5B;RAP2B;RBL1;RBM39;RBSN;RCC2;RCOR1;RGS2;RMND5A;RPL24;RPL35A;RPS2;RPS4X;RPSA;SCD;SERBP1;SFXN1;SIRT1;SLC2A1;SLC7A5;SNAI1;SOGA1;SP1;SPG11;SRPK1;SRSF7;STX4;TACC1;TBC1D12;TBX3;TCEAL1;TCF7;TET2;TFRC;TIAM1;TMED4;TMEM120B;TMEM178B;TMEM201;TNFRSF10D;TPD52L2;TRAF3IP1;TSC22D4;TTC33;UBR5;VAPB;VASN;VSNL1;WNT1;WWC1;YWHAZ;ZMAT5;ZNF217;ZNF431;ZNF460;ZNF646;ZNF662

Supplementary Table S5: Comprehensive mapping of analyzed miRNAs to their validated gene targets. The table lists all miRNAs for which validated targets could be identified in the miRTarBase database. For each miRNA, the corresponding differential expression statistics (log2FC and p-value) are provided, along with a semicolon-separated list of its validated target gene symbols. This table forms the basis for the Gene Set Enrichment Analysis (GSEA).
