# Supplementary material for: Systemic downregulation of EV-associated MiRNAs following remote ischemic preconditioning
Source: Sci Rep. 2025 Dec 11;15:43657. doi: 10.1038/s41598-025-31356-9 (PMC12701078; doi:10.1038/s41598-025-31356-9)
Supplement: Supplementary file 2 — Supplementary Material 2 [file 41598_2025_31356_MOESM2_ESM.pdf]

Supplementary Figures for *Systemic Downregulation of EV-Associated miRNAs Following Remote Ischemic Preconditioning*

Marius Drysch,[1] Alexander Fiedler[1], Sonja Verena Schmidt,[1] Felix Reinkemeier,[1] Flemming Puscz[1], Tabea Kurbacher[2], Ulrich Frey[3], Crista Ochsenfarth[3], Marcus Lehnhardt,[1] Christoph Wallner,[1#] Alexander Sogorski[1#]

[1] Department of Plastic Surgery, BG University Hospital Bergmannsheil, Ruhr University Bochum, Bürkle-de-la-Camp Platz 1, 44789 Bochum, Germany

[2] Department of Gynecology and Obstetrics, St. Elisabeth-Hospital, Ruhr University Bochum, Bleichstraße 15, 44789 Bochum, Germany

[3] Department of Anesthesia, Intensive Care, Pain and Palliative Medicine, Ruhr-University Bochum, Marien Hospital Herne, 44625, Herne, Germany

# These authors contributed equally

Corresponding author:
Marius Drysch, MD, MHBA
Bürkle-de-la-Camp-Platz 1
44789 Bochum, Germany
+49 234 302 0
Email: marius.drysch@rub.de

Keywords: Extracellular vesicles, miRNA, Ischemia-Reperfusion Injury, Free Flap surgery, Remote Ischemic Preconditioning, EV-miRNA profiling, GSEA

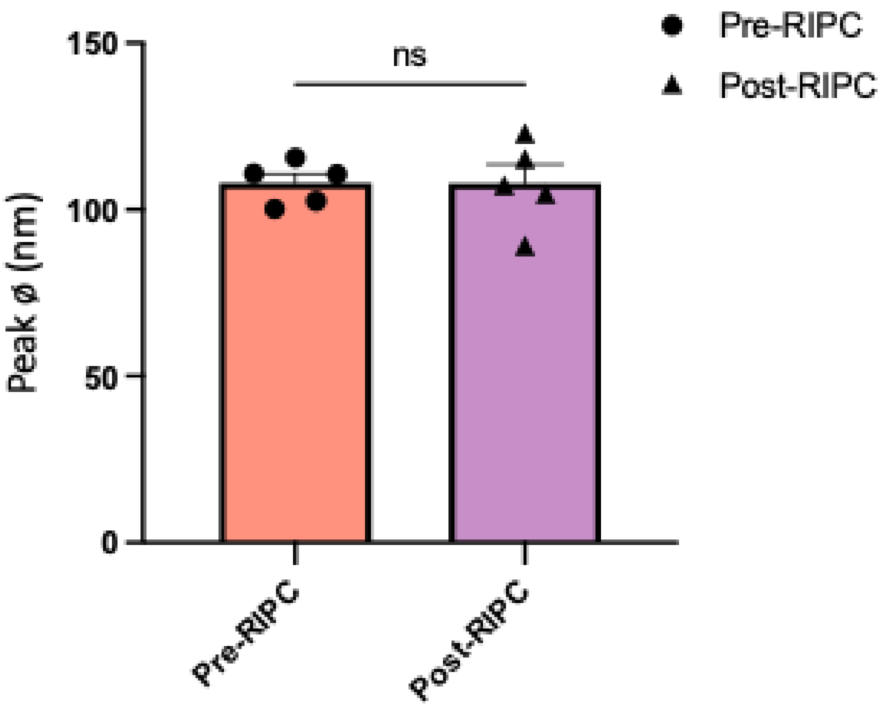

Supplementary Figure S1. Nanoparticle Tracking Analysis (NTA) of Extracellular Vesicle Size. Comparison of the peak particle diameter (nm) of extracellular vesicles (EVs) isolated from patient plasma immediately before (Pre-RIPC) and 30 minutes after (Post-RIPC) RIPC. Bars represent mean ± SEM and dots represent individual patient values. Statistical analysis revealed no significant difference (ns) in EV size between the two time points, indicating that the RIPC stimulus does not alter the modal size of the circulating vesicle population.

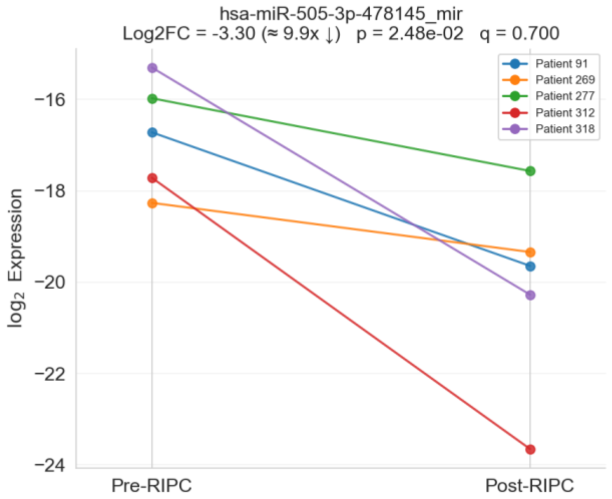
hsa-miR-505-3p-478145_mir
Log2FC = -3.30 (≈ 9.9x ↓) p = 2.48e-02 q = 0.700
Patient 91
Patient 269
Patient 277
Patient 312
Patient 318
log2 Expression
−16
−18
−20
−22
−24
Pre-RIPC
Post-RIPC

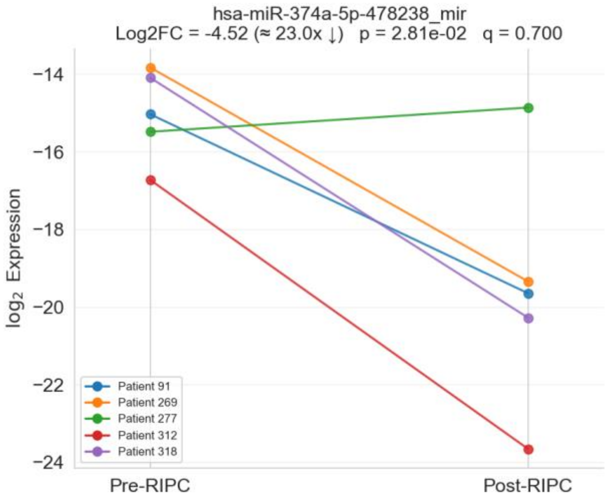
hsa-miR-374a-5p-478238_mir
Log2FC = -4.52 (≈ 23.0x ↓) p = 2.81e-02 q = 0.700
Patient 91
Patient 269
Patient 277
Patient 312
Patient 318
log2 Expression
−14
−16
−18
−20
−22
−24
Pre-RIPC
Post-RIPC

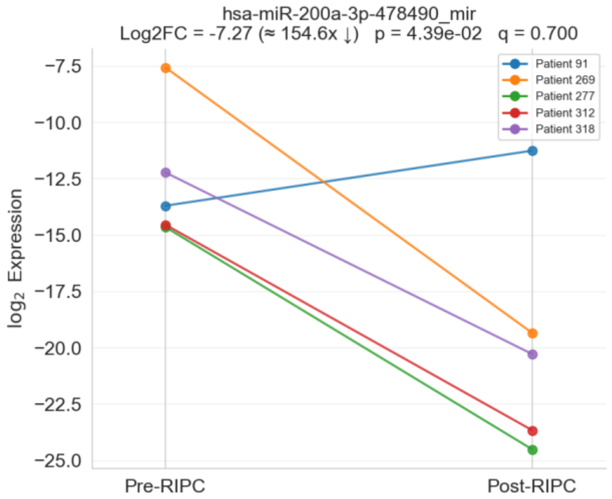
hsa-miR-200a-3p-478490_mir
Log2FC = -7.27 (≈ 154.6x ↓) p = 4.39e-02 q = 0.700
Patient 91
Patient 269
Patient 277
Patient 312
Patient 318
log2 Expression
−7.5
−10.0
−12.5
−15.0
−17.5
−20.0
−22.5
−25.0
Pre-RIPC
Post-RIPC

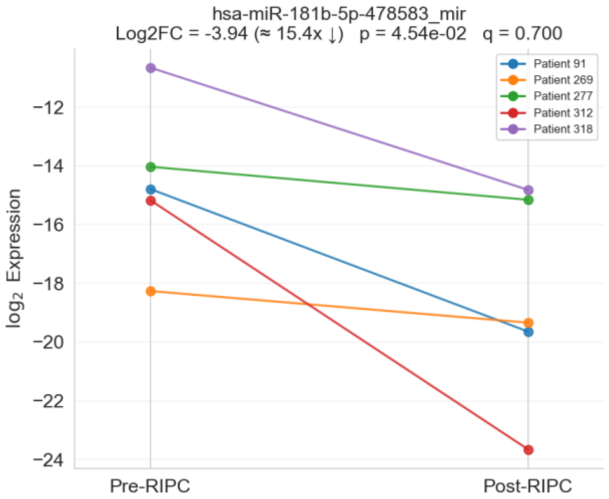
hsa-miR-181b-5p-478583_mir
Log2FC = -3.94 (≈ 15.4x ↓) p = 4.54e-02 q = 0.700
Patient 91
Patient 269
Patient 277
Patient 312
Patient 318
log2 Expression
−12
−14
−16
−18
−20
−22
−24
Pre-RIPC
Post-RIPC

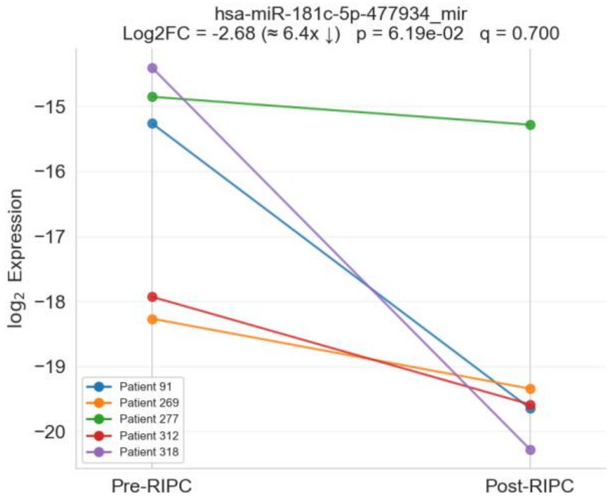
hsa-miR-181c-5p-477934_mir
Log2FC = -2.68 (≈ 6.4x ↓) p = 6.19e-02 q = 0.700
Patient 91
Patient 269
Patient 277
Patient 312
Patient 318
log2 Expression
−15
−16
−17
−18
−19
−20
Pre-RIPC
Post-RIPC

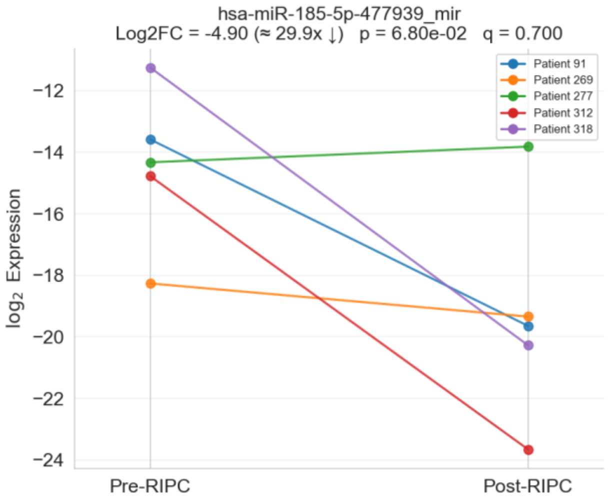
hsa-miR-185-5p-477939_mir
Log2FC = -4.90 (≈ 29.9x ↓) p = 6.80e-02 q = 0.700
Patient 91
Patient 269
Patient 277
Patient 312
Patient 318
log2 Expression
−12
−14
−16
−18
−20
−22
−24
Pre-RIPC
Post-RIPC

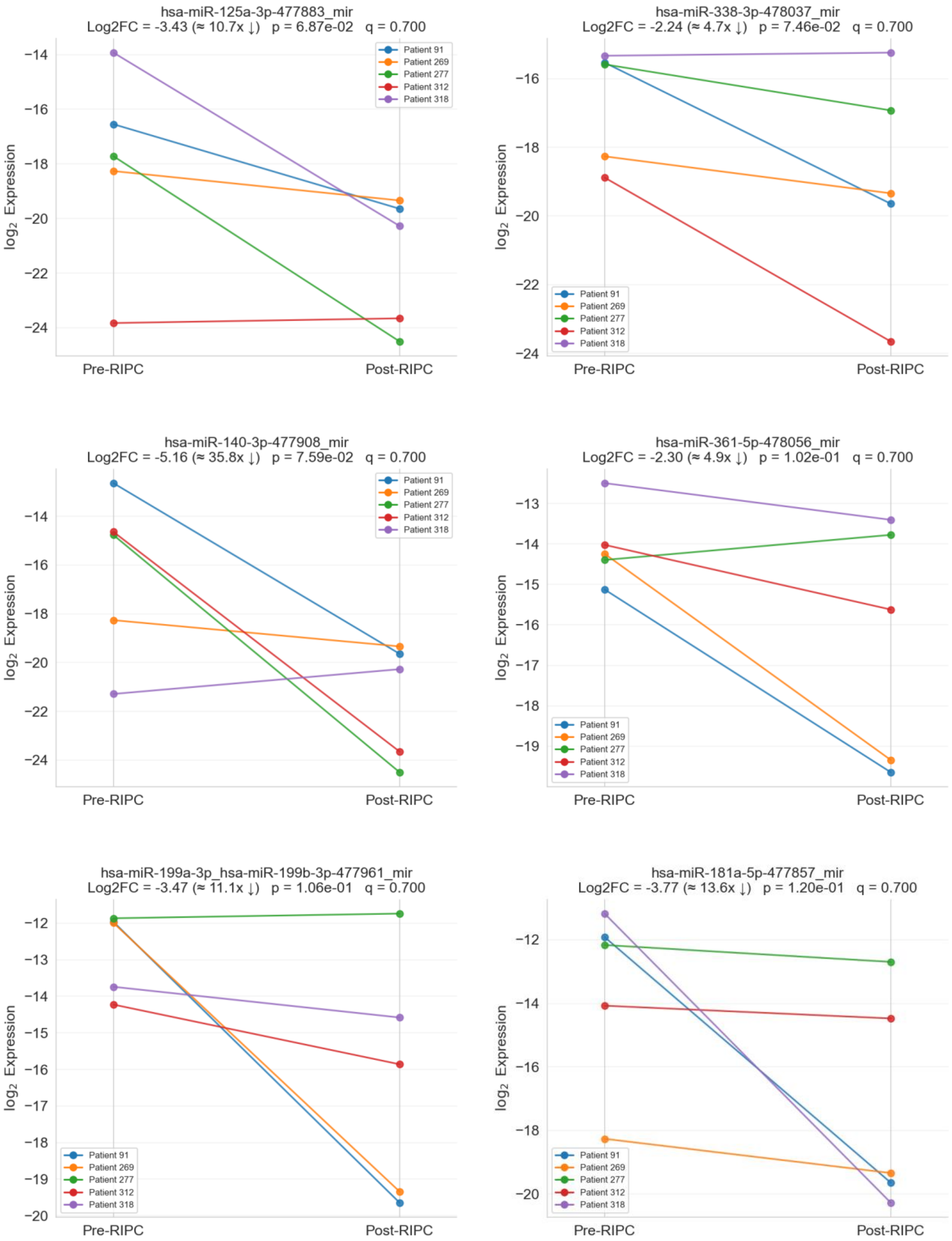
hsa-miR-125a-3p-477883_mir
Log2FC = -3.43 (≈ 10.7x ↓)   p = 6.87e-02   q = 0.700
hsa-miR-338-3p-478037_mir
Log2FC = -2.24 (≈ 4.7x ↓)   p = 7.46e-02   q = 0.700
hsa-miR-140-3p-477908_mir
Log2FC = -5.16 (≈ 35.8x ↓)   p = 7.59e-02   q = 0.700
hsa-miR-361-5p-478056_mir
Log2FC = -2.30 (≈ 4.9x ↓)   p = 1.02e-01   q = 0.700
hsa-miR-199a-3p_hsa-miR-199b-3p-477961_mir
Log2FC = -3.47 (≈ 11.1x ↓)   p = 1.06e-01   q = 0.700
hsa-miR-181a-5p-477857_mir
Log2FC = -3.77 (≈ 13.6x ↓)   p = 1.20e-01   q = 0.700
$\log_2$ Expression
Pre-RIPC
Post-RIPC
Patient 91
Patient 269
Patient 277
Patient 312
Patient 318

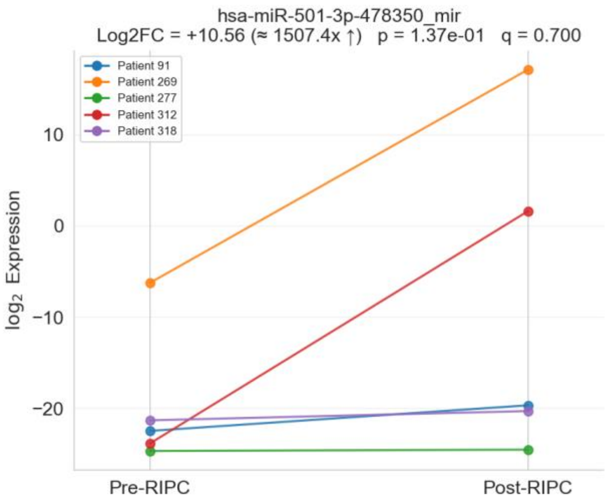
hsa-miR-501-3p-478350_mir
Log2FC = +10.56 (≈ 1507.4x ↑)   p = 1.37e-01   q = 0.700
Patient 91
Patient 269
Patient 277
Patient 312
Patient 318
log2 Expression
10
0
−10
−20
Pre-RIPC
Post-RIPC

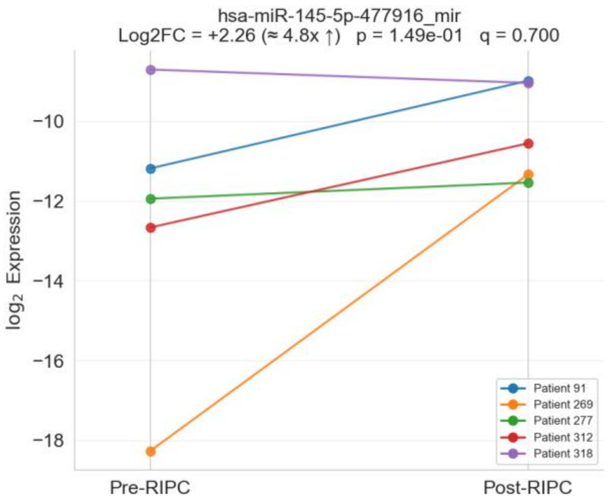
hsa-miR-145-5p-477916_mir
Log2FC = +2.26 (≈ 4.8x ↑)   p = 1.49e-01   q = 0.700
Patient 91
Patient 269
Patient 277
Patient 312
Patient 318
log2 Expression
−10
−12
−14
−16
−18
Pre-RIPC
Post-RIPC

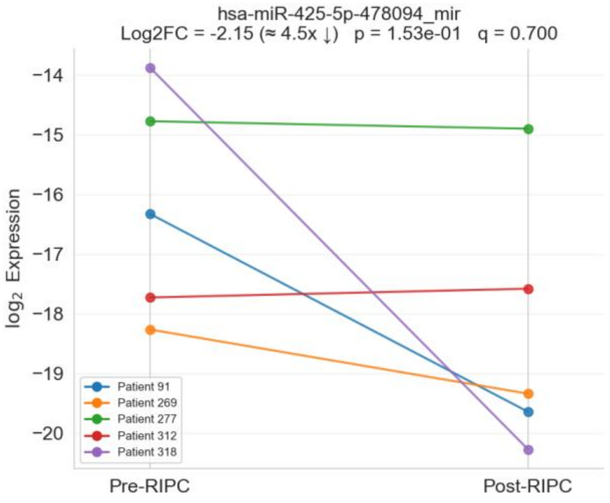
hsa-miR-425-5p-478094_mir
Log2FC = -2.15 (≈ 4.5x ↓)   p = 1.53e-01   q = 0.700
Patient 91
Patient 269
Patient 277
Patient 312
Patient 318
log2 Expression
−14
−15
−16
−17
−18
−19
−20
Pre-RIPC
Post-RIPC

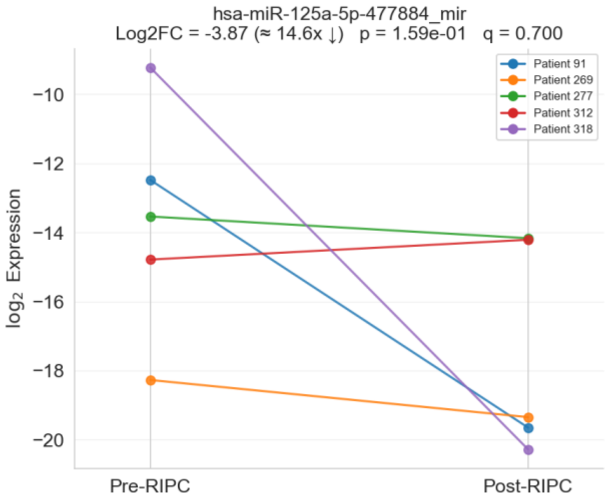
hsa-miR-125a-5p-477884_mir
Log2FC = -3.87 (≈ 14.6x ↓)   p = 1.59e-01   q = 0.700
Patient 91
Patient 269
Patient 277
Patient 312
Patient 318
log2 Expression
−10
−12
−14
−16
−18
−20
Pre-RIPC
Post-RIPC

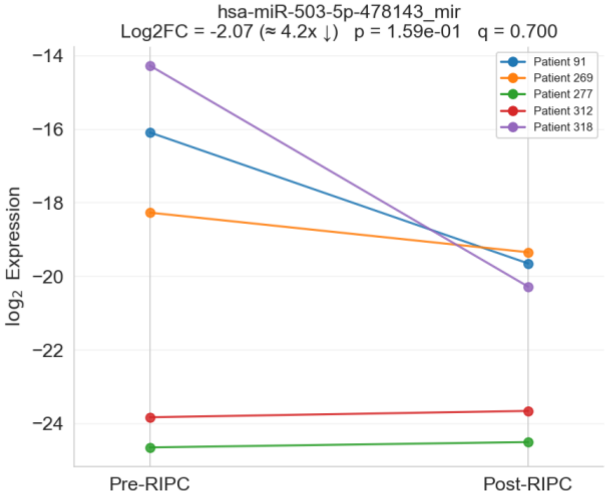
hsa-miR-503-5p-478143_mir
Log2FC = -2.07 (≈ 4.2x ↓)   p = 1.59e-01   q = 0.700
Patient 91
Patient 269
Patient 277
Patient 312
Patient 318
log2 Expression
−14
−16
−18
−20
−22
−24
Pre-RIPC
Post-RIPC

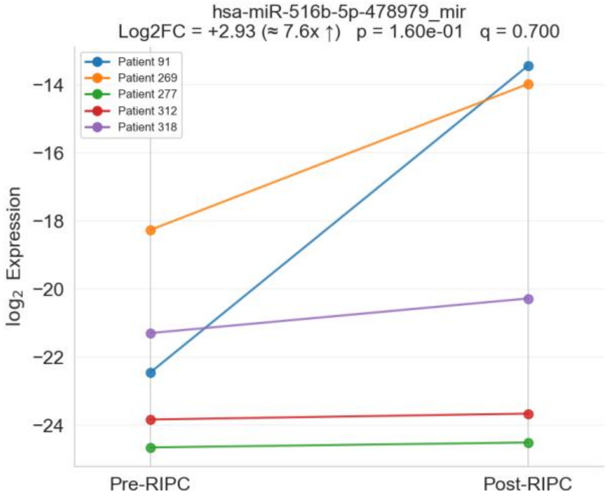
hsa-miR-516b-5p-478979_mir
Log2FC = +2.93 (≈ 7.6x ↑)   p = 1.60e-01   q = 0.700
Patient 91
Patient 269
Patient 277
Patient 312
Patient 318
log2 Expression
−14
−16
−18
−20
−22
−24
Pre-RIPC
Post-RIPC

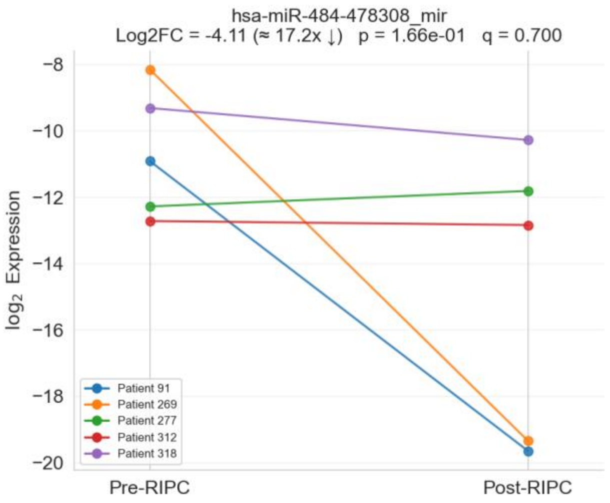
hsa-miR-484-478308_mir
Log2FC = -4.11 (≈ 17.2x ↓)   p = 1.66e-01   q = 0.700
log2 Expression
−8
−10
−12
−14
−16
−18
−20
Pre-RIPC
Post-RIPC
Patient 91
Patient 269
Patient 277
Patient 312
Patient 318

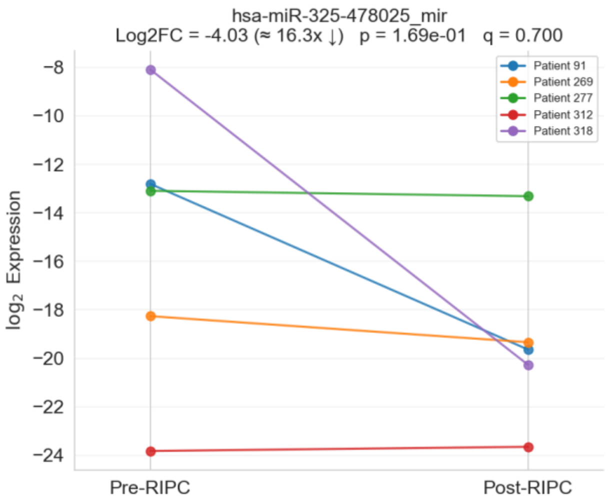
hsa-miR-325-478025_mir
Log2FC = -4.03 (≈ 16.3x ↓)   p = 1.69e-01   q = 0.700
log2 Expression
−8
−10
−12
−14
−16
−18
−20
−22
−24
Pre-RIPC
Post-RIPC
Patient 91
Patient 269
Patient 277
Patient 312
Patient 318

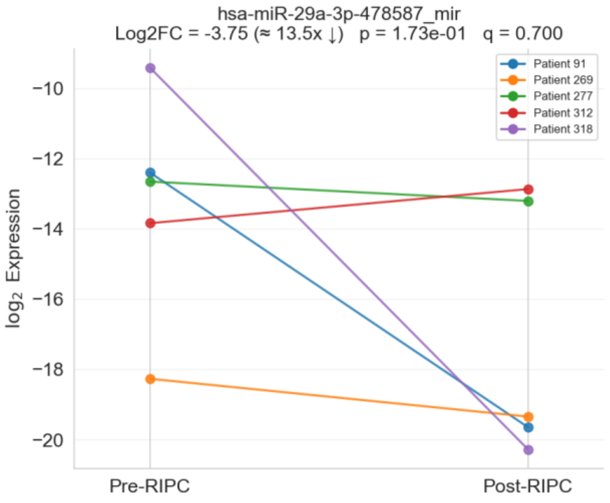
hsa-miR-29a-3p-478587_mir
Log2FC = -3.75 (≈ 13.5x ↓)   p = 1.73e-01   q = 0.700
log2 Expression
−10
−12
−14
−16
−18
−20
Pre-RIPC
Post-RIPC
Patient 91
Patient 269
Patient 277
Patient 312
Patient 318

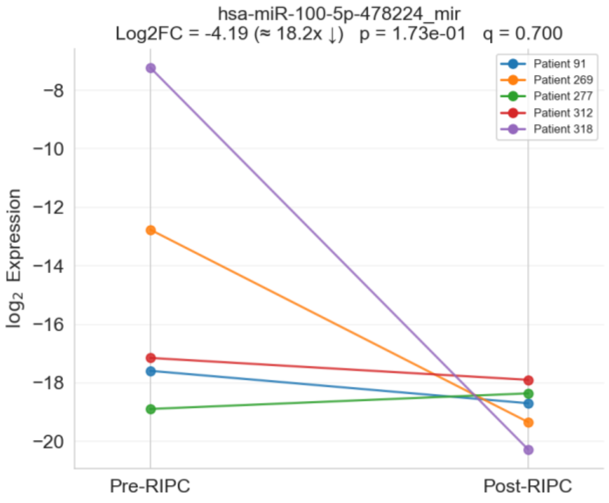
hsa-miR-100-5p-478224_mir
Log2FC = -4.19 (≈ 18.2x ↓)   p = 1.73e-01   q = 0.700
log2 Expression
−8
−10
−12
−14
−16
−18
−20
Pre-RIPC
Post-RIPC
Patient 91
Patient 269
Patient 277
Patient 312
Patient 318

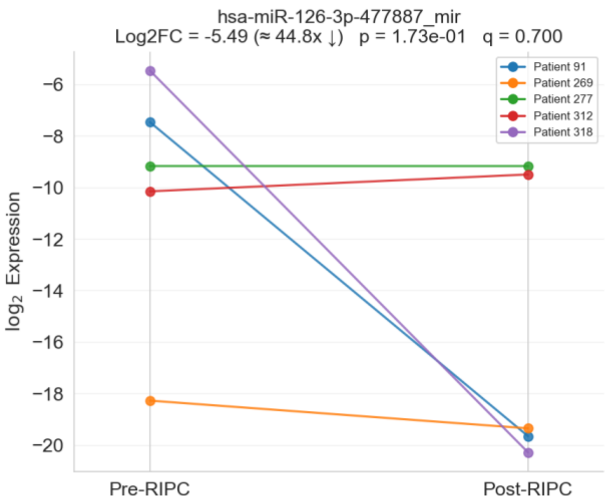
hsa-miR-126-3p-477887_mir
Log2FC = -5.49 (≈ 44.8x ↓)   p = 1.73e-01   q = 0.700
log2 Expression
−6
−8
−10
−12
−14
−16
−18
−20
Pre-RIPC
Post-RIPC
Patient 91
Patient 269
Patient 277
Patient 312
Patient 318

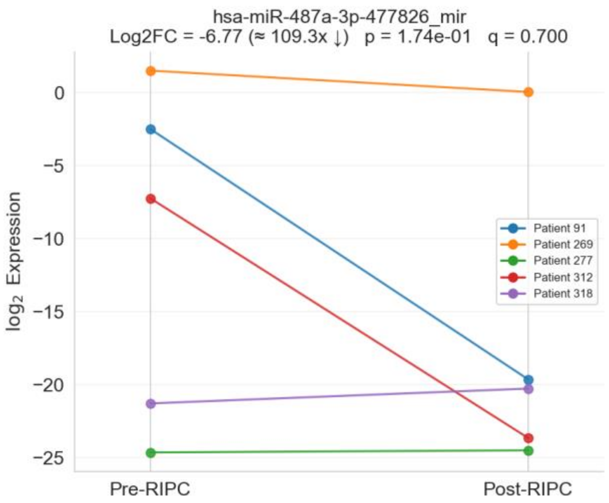
hsa-miR-487a-3p-477826_mir
Log2FC = -6.77 (≈ 109.3x ↓)   p = 1.74e-01   q = 0.700
log2 Expression
0
−5
−10
−15
−20
−25
Pre-RIPC
Post-RIPC
Patient 91
Patient 269
Patient 277
Patient 312
Patient 318

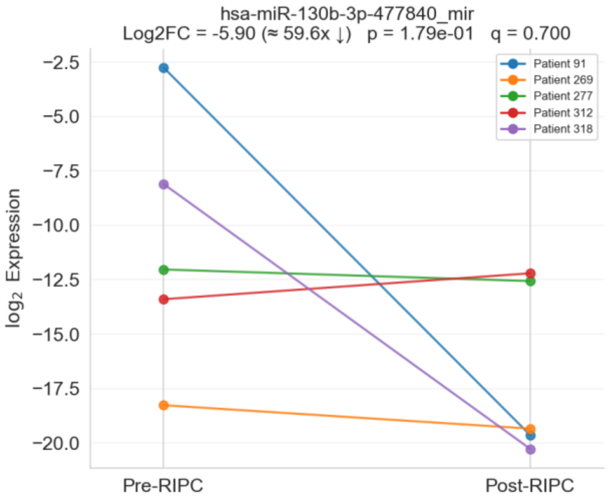

Supplementary Figure S2. Individual Patient Trajectories of Top Differentially Expressed miRNAs. Paired top 25 miRNAs ranked by p-value. Each plot represents a single miRNA from the top 25 candidates identified in the differential expression analysis. The y-axis displays the expression level. Lines connect the Pre-RIPC and Post-RIPC samples for each of the five patients (color-coded). The header for each plot includes the miRNA identifier, the log2 fold change (Log2FC), the fold change magnitude, the unadjusted p-value, and the False Discovery Rate q-value.

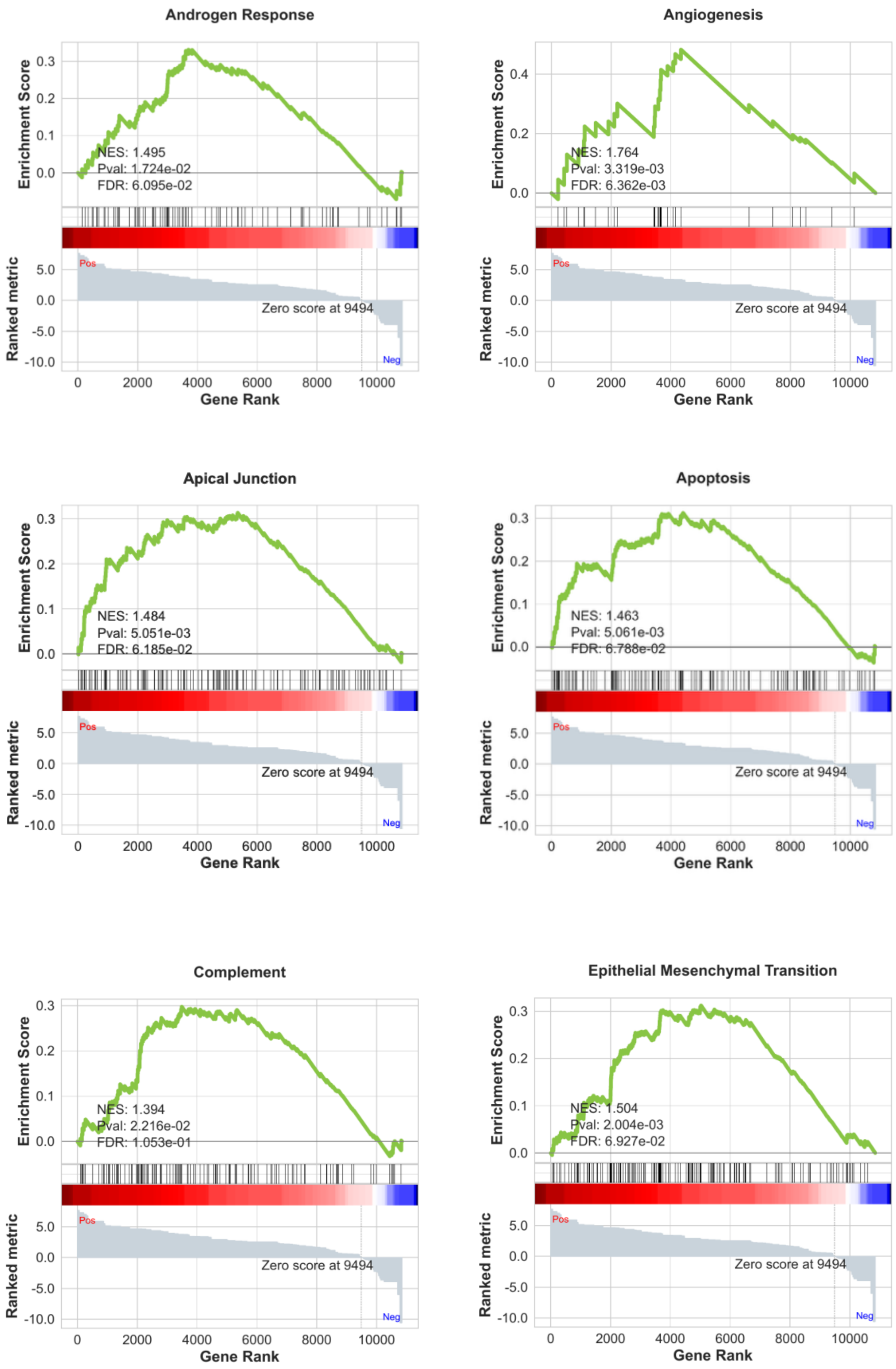
Androgen Response
NES: 1.495
Pval: 1.724e-02
FDR: 6.095e-02
Angiogenesis
NES: 1.764
Pval: 3.319e-03
FDR: 6.362e-03
Apical Junction
NES: 1.484
Pval: 5.051e-03
FDR: 6.185e-02
Apoptosis
NES: 1.463
Pval: 5.061e-03
FDR: 6.788e-02
Complement
NES: 1.394
Pval: 2.216e-02
FDR: 1.053e-01
Epithelial Mesenchymal Transition
NES: 1.504
Pval: 2.004e-03
FDR: 6.927e-02
Enrichment Score
Ranked metric
Pos
Neg
Zero score at 9494
Gene Rank

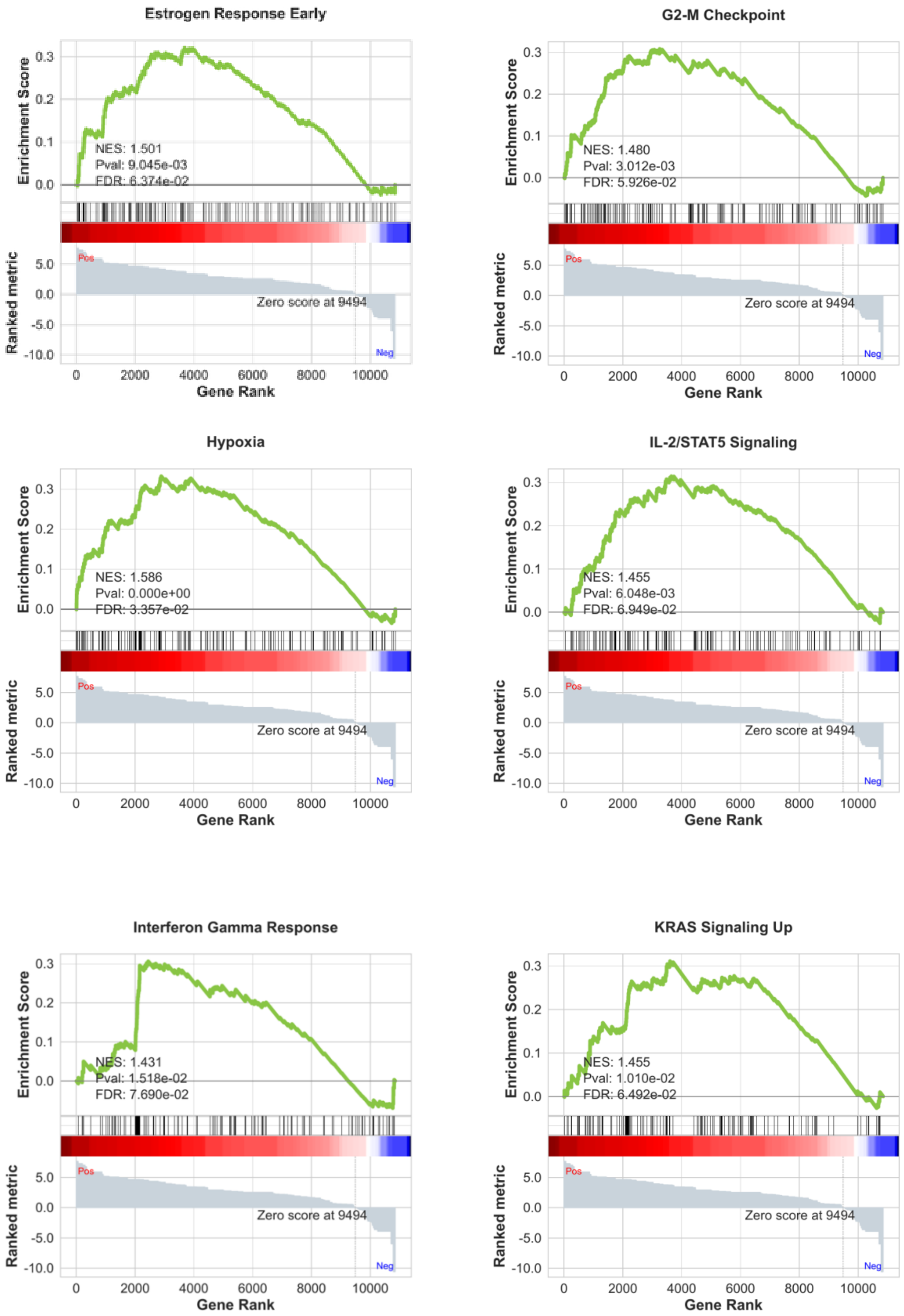

Estrogen Response Early
NES: 1.501
Pval: 9.045e-03
FDR: 6.374e-02
Zero score at 9494
G2-M Checkpoint
NES: 1.480
Pval: 3.012e-03
FDR: 5.926e-02
Zero score at 9494
Hypoxia
NES: 1.586
Pval: 0.000e+00
FDR: 3.357e-02
Zero score at 9494
IL-2/STAT5 Signaling
NES: 1.455
Pval: 6.048e-03
FDR: 6.949e-02
Zero score at 9494
Interferon Gamma Response
NES: 1.431
Pval: 1.518e-02
FDR: 7.690e-02
Zero score at 9494
KRAS Signaling Up
NES: 1.455
Pval: 1.010e-02
FDR: 6.492e-02
Zero score at 9494
Enrichment Score
Ranked metric
Gene Rank
Pos
Neg

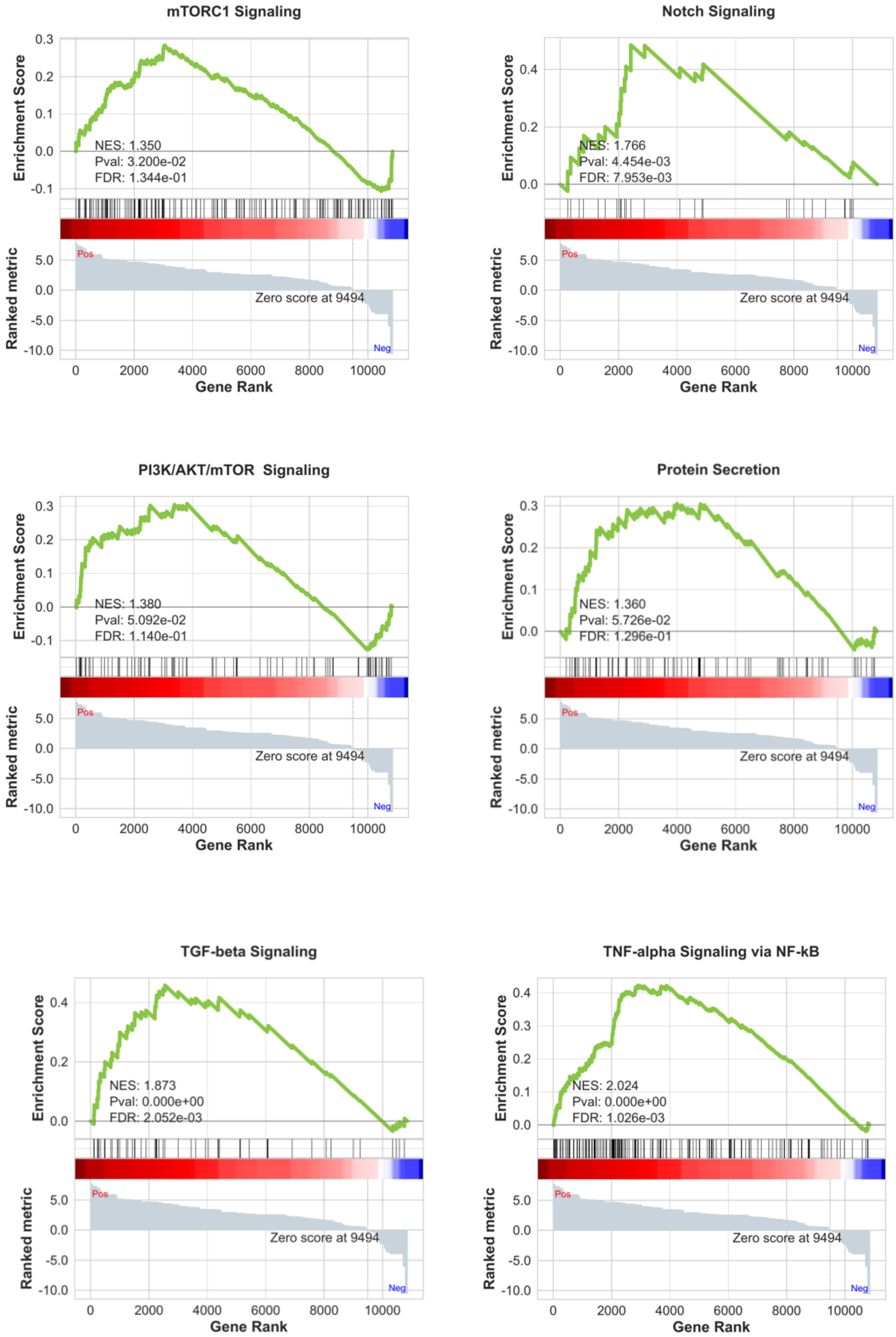
mTORC1 Signaling
NES: 1.350
Pval: 3.200e-02
FDR: 1.344e-01
Enrichment Score
Ranked metric
Pos
Neg
Zero score at 9494
Gene Rank
Notch Signaling
NES: 1.766
Pval: 4.454e-03
FDR: 7.953e-03
Enrichment Score
Ranked metric
Pos
Neg
Zero score at 9494
Gene Rank
PI3K/AKT/mTOR Signaling
NES: 1.380
Pval: 5.092e-02
FDR: 1.140e-01
Enrichment Score
Ranked metric
Pos
Neg
Zero score at 9494
Gene Rank
Protein Secretion
NES: 1.360
Pval: 5.726e-02
FDR: 1.296e-01
Enrichment Score
Ranked metric
Pos
Neg
Zero score at 9494
Gene Rank
TGF-beta Signaling
NES: 1.873
Pval: 0.000e+00
FDR: 2.052e-03
Enrichment Score
Ranked metric
Pos
Neg
Zero score at 9494
Gene Rank
TNF-alpha Signaling via NF-kB
NES: 2.024
Pval: 0.000e+00
FDR: 1.026e-03
Enrichment Score
Ranked metric
Pos
Neg
Zero score at 9494
Gene Rank

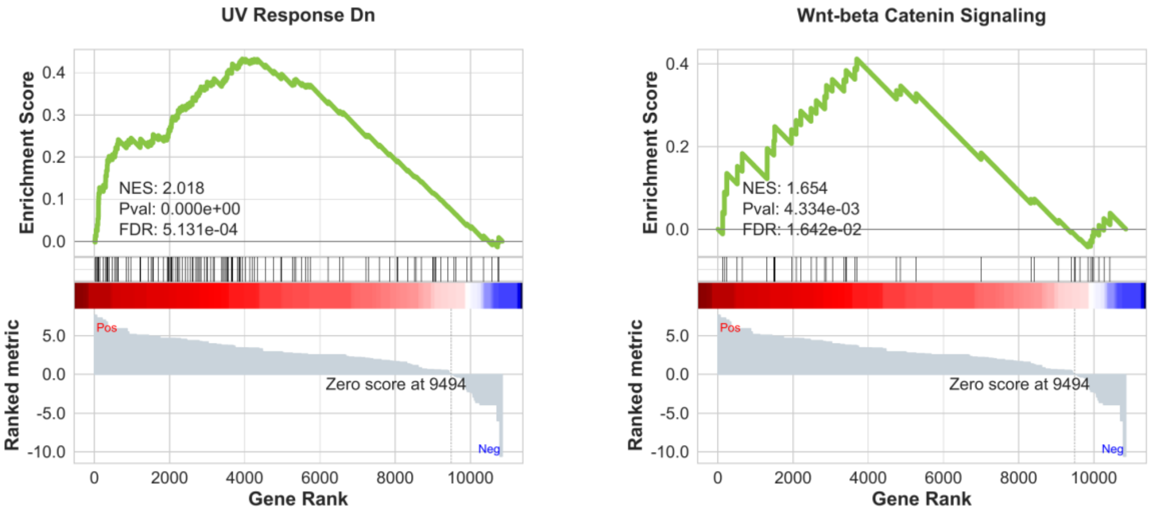

Supplementary Figure S3. Enrichment plots for significant Hallmark pathways. Detailed GSEA plots for pathways enriched within the target genes of the RIPC-modulated miRNAs. The graphs visualize the running enrichment score (green line) across the gene list, which is ranked by the negative log2 fold change of the targeting miRNAs. A positive Enrichment Score (peaks to the left) indicates that the gene set is overrepresented among the targets of miRNAs downregulated by RIPC, suggesting a functional derepression of these pathways.
